# Residue-Level Affinity Decomposition via Quantum Electron Density: A Multivariable Framework Applied to HIV‑1 Protease Inhibitors

**DOI:** 10.1021/acs.jpcb.5c08041

**Published:** 2026-04-02

**Authors:** Jorge Gutiérrez-Flores, Gerardo Padilla-Bernal, César Sánchez-Juárez, Dulce M. Méndez-Orduña, Javier Serrano Medina, Ponciano García-Gutiérrez, Rafael A. Zubillaga, Rubicelia Vargas

**Affiliations:** Departamento de Química, División de Ciencias Básicas e Ingeniería, 428230 Universidad Autónoma Metropolitana Iztapalapa, San Rafael Atlixco 186, Col. Vicentina, C.P., 09340 Iztapalapa CDMX, México

## Abstract

Understanding the molecular determinants that govern protein–ligand binding remains a central challenge in computational chemistry and rational drug design. Here, we introduce a quantum-informed, residue-level affinity decomposition framework that integrates experimental thermodynamic data with multivariable modeling of electron-density descriptors. Using a unified computational workflow combining molecular dynamics simulations, semiempirical hydrogen refinement, Density Functional Theory single-point calculations, and QTAIM analysis, we quantified noncovalent interactions between HIV-1 protease and six clinically approved inhibitors. Although the total electron density at bond critical points showed no direct correlation with experimental binding enthalpies, residue-specific multivariate models revealed that affinity is primarily governed by the nature of individual contacts rather than by their overall number or density. This statistical decomposition is further supported by Hessian-based electron-density descriptors and NCI index, which provide a physical interpretation of stabilizing and destabilizing residue-level interactions. The models clearly distinguished between stabilizing (cooperative) and destabilizing (anticooperative) residue contributions, highlighting the mechanistic influence of structured water. Comparison with MM-PBSA per-residue energies further supported the predictive value of the quantum descriptors. Importantly, the analysis is independent of the structural relatedness of the ligands, underscoring its applicability to chemically diverse scaffolds. Overall, this study presents a transferable, quantum-topological framework for mechanistic protein–ligand affinity analysis, offering a generalizable strategy for structure-guided optimization of inhibitors targeting a common binding site.

## Graphical abstract

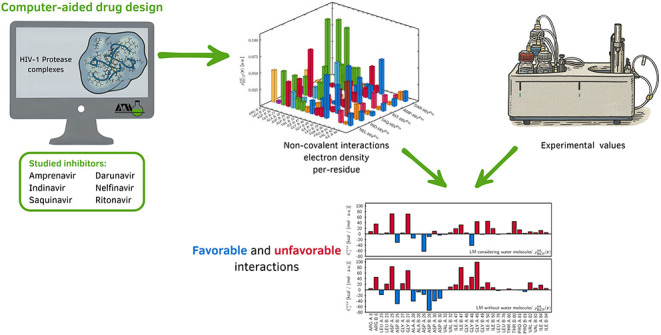

## 1 Introduction

Drug design plays a key role in medicinal chemistry, enabling the development of effective therapies for a wide range of diseases.^1^ A fundamental objective of rational drug design is to improve therapeutic efficacy while minimizing adverse effects by understanding, at the molecular level, how drug molecules interact with their biological targets. In this context, computer-aided drug design (CADD) has become an indispensable strategy.^2,3^ By employing computational tools to model ligand-target interactions and predict molecular behavior, CADD significantly accelerates the discovery and optimization of drug candidates, particularly when integrated with experimental structural and thermodynamic data.^4^

A key strategy in CADD involves identifying and optimizing the noncovalent interactions that govern protein–ligand recognition and binding affinity.^5^ Classical approaches, including molecular docking and molecular dynamics (MD) simulations, are widely employed to investigate the structural and energetic properties of these interactions. Although these methods are computationally efficient and suitable for large biomolecular systems, they rely on empirical force fields whose accuracy is limited by the constraints of their parametrization, among other limitations.^6^ As a result, they often provide an approximate representation of the noncovalent landscape and may overlook weak or noncanonical interactions that critically influence molecular recognition. Semiempirical methods, while less computationally demanding than *ab initio* techniques, also depend on fitted parameters and thus share similar limitations in predictive accuracy.^7^

To overcome these drawbacks, quantum chemical approaches, particularly Density Functional Theory (DFT),^8,9^ have been increasingly adopted. Unlike classical or semiempirical techniques, DFT provides a more accurate description of the electronic structure, which is essential for understanding the nature and strength of noncovalent interactions. Furthermore, DFT grants access to the electron density, a fundamental quantity that can be analyzed via the Quantum Theory of Atoms in Molecules (QTAIM).^10^ Since the electron density is a quantum-mechanical observable, accessible both experimentally and theoretically, which in principle encodes all ground-state properties of a molecular system, QTAIM provides a physically well-founded framework for the statistical modeling and as well as for the rigorous characterization and interpretation of intermolecular interactions that govern binding specificity and strength.^11,12^ In this context, density-based descriptors derived from QTAIM have already been successfully employed within quantitative structure–activity relationship (QSAR) models in CADD, enabling physically interpretable correlations between electronic structure and biological activity.^11^

Several recent studies have demonstrated the advantages of integrating classical,^13–17^ semiempirical,^18–21^ and quantum chemical methods^22–27^ to gain a better understanding of drug–protein interactions. For example, Rojas et al.^28^ combined MD simulations and QTAIM analysis to investigate the binding of ligands to biological targets, revealing weak interactions that are often undetected by conventional force-field-based approaches. Their study reported a strong correlation between topological descriptors, such as the electron density at bond critical points (BCPs) associated with noncovalent interactions, and inhibitory activity (p*K_i_*), highlighting the relevance of QTAIM in rational drug design. In a related and complementary effort, Lefrancois-Gagnon and Mawhinney developed a series of studies establishing QTAIM-based atomic and group descriptors as transferable and physically meaningful representations of substituent effects. Through a systematic progression involving the assessment of descriptor transferability, their correlation with classical empirical substituent proxies, and the development of interpretable multivariate and machine-learning models, these works demonstrated that experimentally derived substituent constants can be reinterpreted as emergent manifestations of underlying electron-density properties.^29–31^ Despite the widespread use of inhibition constants (*K*_*i*_) derived from the Cheng-Prusoff equation^32^ or graphical methods,^33^ these values are sensitive to experimental conditions and do not explicitly account for the underlying intermolecular forces. A thermodynamic perspective, particularly through binding enthalpy (Δ_int_ *H*), offers a more direct assessment of the energetic contributions associated with ligand binding. Notably, Freire emphasized that for HIV-1 protease inhibitors, enhanced potency across successive drug generations correlates with both increasingly favorable enthalpic and entropic contributions.^34^ However, they particularly underscored the importance of enthalpic effects, as these are directly linked to structural features and, therefore, to the specific noncovalent interactions between ligand and target.

In light of these considerations, we present a systematic quantum topological analysis of the noncovalent interactions between HIV-1 protease and six clinically approved inhibitors: amprenavir, darunavir, indinavir, nelfinavir, ritonavir, and saquinavir. Using a unified computational workflow, we modeled these inhibitors within the wild-type protease binding site through molecular dynamics simulations, followed by the extraction of representative fragments and hydrogen position optimization using semiempirical methods. We then performed single-point DFT energy calculations and QTAIM analysis to identify and quantify the electron density at bond critical points associated with noncovalent contacts. Our findings indicate that binding affinity is not solely dictated by the number of noncovalent interactions but rather by the specific nature and quality of residue-level contacts. To unravel these contributions, we employed a multivariate regression model that distinguishes cooperative from anticooperative interactions at the residue level. By integrating quantum topological descriptors with statistical modeling, this strategy enables a residue-level decomposition of favorable and unfavorable contributions, providing mechanistic insights and a rational basis for optimizing both structurally related and chemically diverse inhibitors targeting a common binding site. While multivariate modeling based on QTAIM descriptors has been previously explored in QSAR contexts, to the best of our knowledge, this is the first application that systematically breaks down protein–ligand binding affinity at the residue level, identifying stabilizing and destabilizing contributions within a biologically relevant system. By doing so, this study establishes a comprehensive and physically interpretable framework for structure-based drug design and molecular recognition. This approach has broad implications for understanding the structure–activity relationships in protein–ligand systems.

## 2 Computational Details

### 2.1 Molecular Dynamics Simulations and MM-PBSA Calculations

Each inhibitor-HIV-1 protease (Inh-HIV^Pro^) complex was modeled using AlphaFold 3,^35^ with Inh representing one of the following inhibitors: amprenavir (AMP), darunavir (DAR), indinavir (IND), nelfinavir (NEL), ritonavir (RIT), or saquinavir (SAQ), based on the HIV^Pro^ subtype B sequence. The p*K*_a_ values of acidic and basic residues were estimated using the PropKa^36,37^ algorithm implemented in the PDB2PQR server (https://server.poissonboltzmann.org/pdb2pqr), and protonation states were assigned to both the protein and the inhibitors at pH 5.0.

All-atom MD simulations were performed for each Inh-HIV^Pro^ complex and the free HIV^Pro^ using the AMBER-19SB force field.^38,39^ System parametrization was carried out via CHARMM-GUI (https://www.charmm-gui.org),^40,41^ and each system was solvated in a dodecahedral box containing TIP4P-Ewald water molecules,^42^ with NaCl added to neutralize the system and reach an ionic strength of 1 M. Energy minimization was conducted using the steepest descent algorithm with a force convergence criterion of *F*_max_ = 1000 kJ mol^–1^ nm^–1^. Equilibration was performed under NVT and NPT ensembles at 298.15 K and 1 bar, respectively, with harmonic restraints (1000 kJ mol^–1^ nm^–1^) applied to heavy protein atoms. The V-rescale thermostat^43^ and C-rescale barostat^44^ were used for temperature and pressure control, respectively.

Production MD simulations were run using five independent replicates per system, with a 2 fs integration time step under periodic boundary conditions. From each trajectory, 500 frames were extracted for binding free energy calculations using the Molecular Mechanics Poisson–Boltzmann surface area (MM-PBSA) method^45^ (version 1.6.2). The ff99SB^46^ and GAFF^47,48^ force fields were employed at a simulation temperature of 298.15 K. MM-PBSA parameters included interaction_entropy = 1,^49^ c2_entropy = 1,^50^ and no quasi-harmonic approximation. The linearized Poisson–Boltzmann model was used with the following settings: ipb = 2, inp = 2; an internal dielectric constant (ε_internal) of 15.85 (estimated using the Neumann fluctuation formula^51^); an external dielectric constant (ε_external) of 80.0; ionic strength of 1 M; optimized atomic radii; and a probe radius of 1.4 Å. Grid spacing was set to 0.5 Å. Per-residue energy decomposition (idecomp = 1) was limited to residues within 3.5 Å of the inhibitor. All MD simulations and MM-PBSA analyses were carried out using GROMACS version 2023.^52^

### 2.2 Reduced Models (Protein Cavities)

For each Inh-HIV^Pro^ complex, the five MD simulation replicates were concatenated and subjected to principal component analysis (PCA). The free energy landscape (FEL) was constructed using the first two principal components (PC1 and PC2), and the frame corresponding to the center of the global minimum was selected using tools from MDAnalysis and GROMACS.^53–55^ This representative structure was then reduced for quantum mechanical analysis by retaining all protein residues within 4.5 Å of the inhibitor, along with nearby water molecules. To maintain chemical integrity, hydrogen atoms were added to cap the terminal amino and carboxyl groups of cleaved peptide bonds. The number of atoms and net charges of the resulting models are summarized in Table 1.

**Table 1:** General Characteristics of the Studied Systems^a^

system	*n*_T_	*n*_LIG_	*n*_PRT_	*n*_ASP_	*n*_H_2_O_	*q*_T_	*q*_LIG_	Δ_int_ *H*
DAR-HIV^Pro^	763	75	676	4	4	–2	0	–12.7
AMP-HIV^Pro^	754	70	675	5	3	–3	0	–6.9
RIT-HIV^Pro^	787	98	677	3	4	–1	0	–4.3
SAQ-HIV^Pro^	797	100	676	4	7	–1	1	1.2
IND-HIV^Pro^	776	93	677	3	2	0	1	1.8
NEL-HIV^Pro^	770	86	675	5	3	–2	1	3.1

a *n*_T_ represents the total number of atoms, while *n*_LIG_, *n*_PRT_, and *n*_ASP_ correspond to the number of atoms in the ligands, protein cavities, and charged aspartate residues, respectively. *n*_H_2_O_ denotes the number of water molecules in each system. The total charge of the system (*q*_*T*_) and the charge of the ligands (*q*_LIG_) are also reported. Δ_int_ *H* represents the interaction enthalpy, obtained from refs 34,72,73 and is reported in kcal/mol.

Hydrogen positions were optimized using the PM7 semiempirical method,^56^ as implemented in MOPAC,^57^ employing the COSMO implicit solvation model with a dielectric constant of ε = 4.0.^58^ This value is commonly used to approximate the electrostatic environment of protein interiors,^59,60^ ensuring consistency across the workflow.

### 2.3 DFT Calculations and Electron Density Analysis

Single-point (SP) energy calculations were performed on reduced models both including explicit water molecules and excluding them (preserving the same protein–ligand geometry), at the PBE0 ^61^/BSIP-3–21G­(d)^62–64^ level of theory, incorporating Grimme’s D3 dispersion correction^65^ and the COSMO implicit solvation model^66^ (ε = 4.0). These calculations were carried out using the TeraChem software package,^67,68^ with double-precision settings and integration grids of approximately 80,000 points per atom. Although DFT calculations on large biomolecular systems are traditionally considered computationally demanding, the use of GPU-accelerated quantum chemistry platforms such as TeraChem makes such evaluations tractable, even for systems with hundreds of atoms.

The topological analysis of the electron density was performed within the framework of QTAIM,^10^ using the GPUAM code.^69,70^ This GPU-accelerated implementation enables efficient QTAIM analysis of large biomolecular systems by handling the evaluation of electron density and its derivatives on dense integration grids. In QTAIM, two atoms are considered to interact when a (3,-1) bond critical point (BCP) is identified between them, which is connected by a bond path (BP) representing the line of maximum electron density. BCPs are classified as shared-shell (SS) or closed-shell (CS) interactions based on the sign of the Laplacian of the electron density: negative values indicate SS (typically covalent), while positive values denote CS interactions, which include hydrogen bonds, H–H bonds, heteroatom interactions, and other noncovalent contacts.

From this perspective, a physically well-justified approach to modeling protein–ligand interaction energies is to use descriptors derived from bond critical points associated with intermolecular closed-shell interactions. These descriptors directly capture the local electronic features governing noncovalent contacts within the binding site and have been shown to yield robust and physically interpretable QSAR relationships.^11^ In addition, BCP-based QTAIM descriptors offer practical advantages over integrated electron (de)­localization measures, such as localization and delocalization indices, as they are computationally less demanding and avoid complexities associated with matrix-based representations of electron sharing.^11^ Accordingly, in this study, only intermolecular closed-shell BCPs corresponding to protein–ligand noncovalent contacts were considered for further analysis.

Finally, to complement the QTAIM analysis and to provide a spatial and physically intuitive visualization of noncovalent interactions, the Noncovalent Interaction index (NCI) was employed. NCI is based on the reduced density gradient (*s*(**r**)), together with the electron density (ρ­(**r**)) and the sign of the second eigenvalue of the electron density Hessian (λ_2_).^71^ Within this framework, isosurfaces of *s*(**r**) are colored according to the values of sign­(λ_2_)­ρ­(**r**), allowing different interaction regimes to be distinguished: large negative values (blue regions) correspond to strongly attractive interactions, values close to zero (green regions) indicate weak and dispersion-dominated interactions, and large positive values (red regions) are associated with nonattractive or sterically repulsive interactions. NCI analysis was performed using the GPUAM code, ensuring consistency with the QTAIM calculations and enabling an integrated interpretation of directional and nondirectional noncovalent interactions within the protein–ligand binding site.

## 3 Results and Discussion

### 3.1 General Features of the Reduced Systems and Global Distribution of Noncovalent Interactions

Table 1 summarizes the general characteristics of the reduced systems analyzed, including the number of atoms in the ligands (*n*_LIG_) and protein cavities (*n*_PRT_), the number of deprotonated aspartic acid residues (*n*_ASP_), the number of retained water molecules (*n*_H_2_O_), and the total charges of both the systems (*n*_T_) and the ligands (*q*_LIG_). Experimentally reported interaction enthalpies (Δ_int_ *H*) are also provided and serve as reference values for the subsequent analyses.^34,72,73^ The inhibitors are listed in order of increasing interaction enthalpy, from the most favorable (more negative) to the least favorable (more positive).

Figure 1 presents the chemical structures of the six inhibitors, while Figure 2 (left) illustrates, as an example, the reduced model used for the DAR-HIV^Pro^ complex. PDB structures and graphical representations of all reduced systems (Figure S1) are provided in the Supporting Information (SI). To ensure consistency across all systems, the same amino acid sequence was employed to define each reduced protein cavity; however, only residues for which noncovalent interactions with the inhibitor were identified via QTAIM analysis (residues in green in Figure 2) were retained for further analysis. This strategy is further justified by an earlier QTAIM study demonstrating the transferability and relative invariance of atomic and bond properties in genetically encoded amino acids, particularly for main-chain groups and chemically related side chains. Matta et al. showed that such topological descriptors enable meaningful correlations between residue-level electronic properties and experimental thermodynamic data across different chemical environments.^74^ The protonation states of the aspartic acid residues were carefully assigned based on a pH of 5.0, in accordance with experimental estimates.^72,73^ As indicated in Table 1, the number of retained water molecules varies among systems. In most cases, these molecules act as bridges between the protein and the ligand, modulating the overall network of noncovalent interactions.

**Figure 1:**
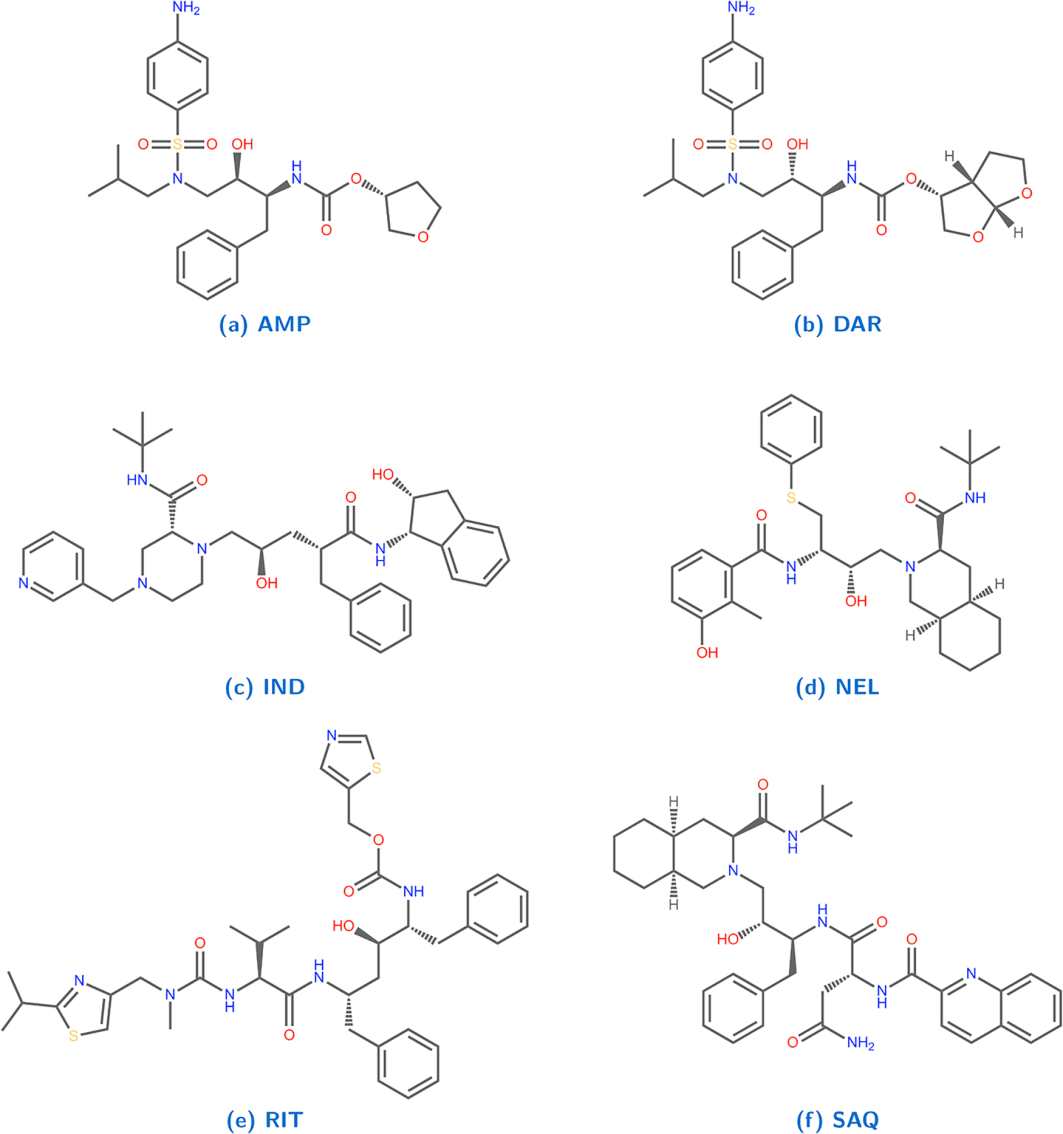
Chemical structures of the six HIV-1 protease inhibitors studied in this work: (a) amprenavir (AMP), (b) darunavir (DAR), (c) indinavir (IND), (d) nelfinavir (NEL), (e) ritonavir (RIT), and (f) saquinavir (SAQ).

**Figure 2:**
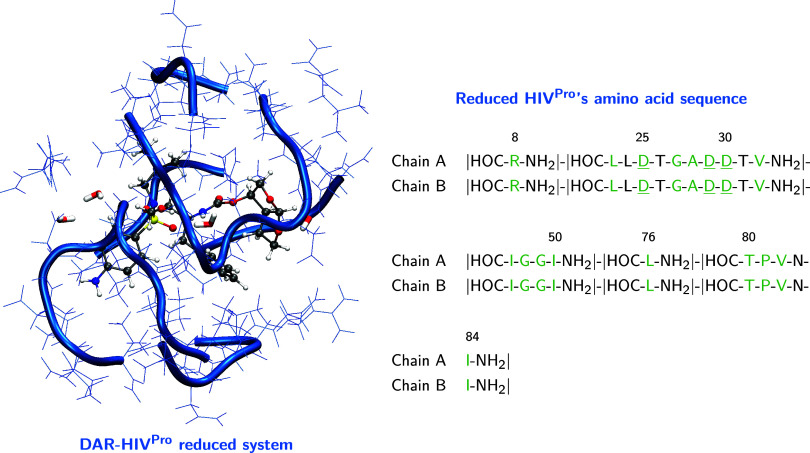
Representative structure of the reduced DAR-HIV^Pro^ complex (left). Equivalent reduced models for the remaining inhibitors are provided in Figure S1 in the Supporting Information (SI). The amino acid sequence used to define the reduced protein cavity is shown on the right and was applied uniformly across all complexes to ensure structural consistency. Residues in green correspond to positions where noncovalent interactions with the inhibitors were identified via QTAIM analysis and were therefore retained for residue-level electronic analysis. Residues with variable protonation states across different inhibitor-bound systems are underlined. In the structural representation, the inhibitor is shown in CPK, water molecules in Licorice, and the protease in Cartoon with residues depicted as blue lines. Atom colors: gray (C), red (O), blue (N), yellow (S), white (H).

Figure 3 shows the total electron density accumulated at BCPs associated with intermolecular noncovalent interactions (ρ_BCP_^int^(**r**)), as well as the percentage contribution of each interaction type (see Table S1 in the SI for numerical values). In all systems, conventional hydrogen bonds (HBs) are the predominant contributors, followed by nonconventional HBs, H–H contacts, and, to a lesser extent, heteroatom-heteroatom (HA) interactions. The ranking obtained from the total accumulated densities is as follows: SAQ > NEL > DAR > AMP > IND > RIT. However, this order does not match the experimentally determined interaction enthalpies, which follow the sequence DAR < AMP < RIT < SAQ < IND < NEL. This discrepancy suggests that the global ρ_BCP_^int^(**r**), although informative, does not directly correlate with experimental affinity.

**Figure 3:**
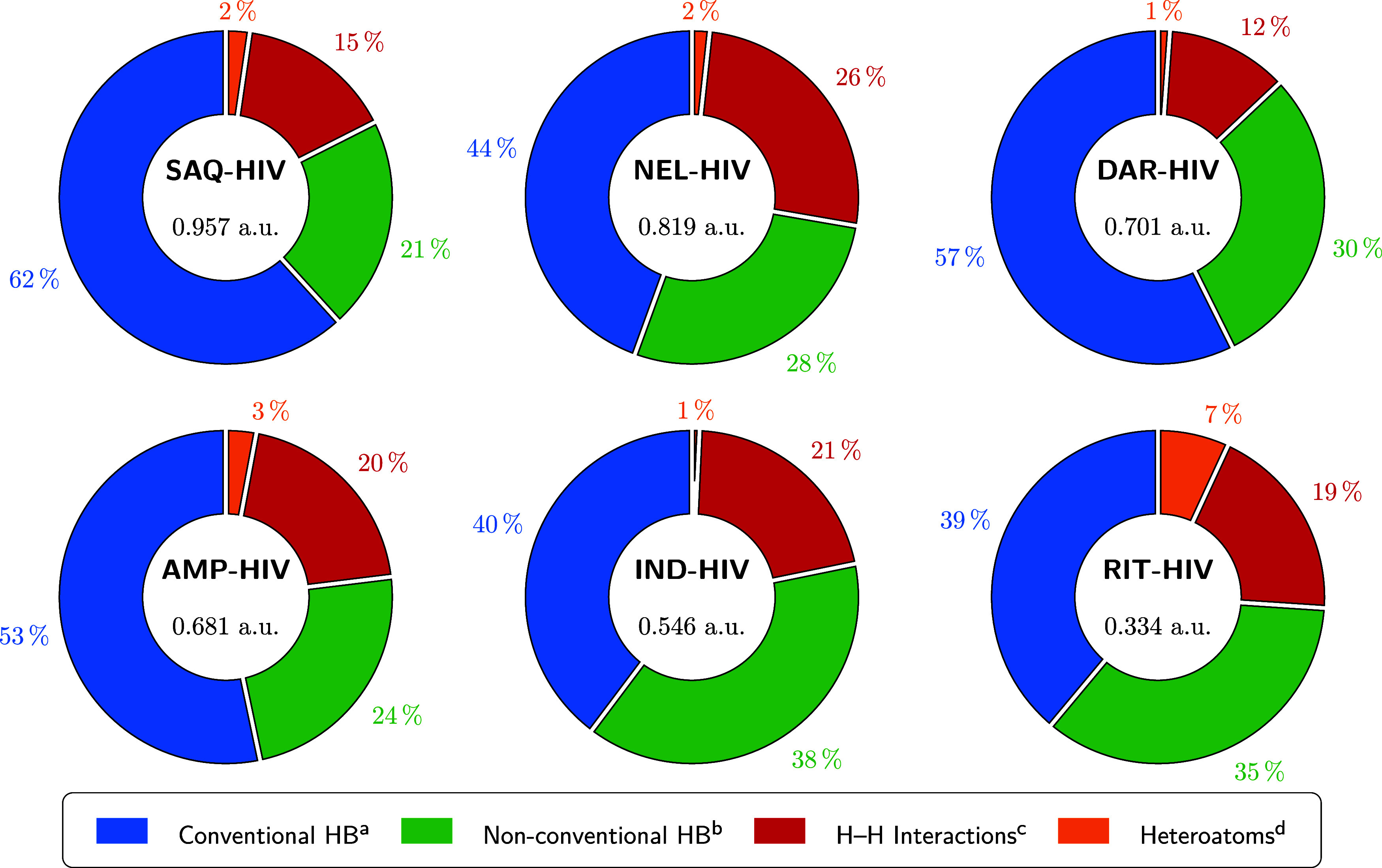
Distribution of ρ_BCP_^int^(**r**) identified in each studied system (values shown at the center of each ring). Interactions involving water molecules are included. Contributions are categorized by interaction type. For simplicity, the superscript “Pro” has been omitted from the inhibitor labels. ^a^ Conventional hydrogen bonds (HB) include N-H···N, N–H···O, O–H···N, and O–H···O interactions. ^b^ Nonconventional hydrogen bonds (HB) include C–H···π, C–H···C, C–H···N, C–H···O, C–H···S, N–H···C, and O–H···C interactions. ^c^ H-H interactions involve C–H···H–C, C–H···H–N, C–H···H–O, and N–H···H–O contacts. ^d^ Heteroatom interactions include C···C, C···O, C···N, N···O, O···O, and O···S interactions.

A consistent trend is observed for amprenavir and darunavir, which are two structurally related inhibitors that differ only by the presence of a tetrahydrofuran (AMP) or a hexahydrofuro­[2,3-*b*]­furan (DAR) moiety. In this case, DAR exhibits both a higher ρ_BCP_^int^(**r**) and a more favorable Δ_int_ *H*. This result indicates that subtle structural modifications in a key anchoring region can expand the noncovalent interaction network and enhance binding affinity.

To further assess the influence of the solvent, Figure 4 decomposes the total accumulated ρ_BCP_^int^(**r**) into protein–ligand, protein-water, and ligand-water contributions (see Table S2 in the SI for full values). The contribution from water molecules follows the trend SAQ (0.604 a.u.) > DAR (0.315 a.u.) > NEL (0.300 a.u.) > AMP (0.279 a.u.) > IND (0.229 a.u.) > RIT (0.172 a.u.), which generally correlates with the number of retained water molecules reported in Table 1. An exception is observed for ritonavir, where, despite the presence of four water molecules, the associated BCP densities are low (0.082 and 0.090 a.u. for protein-water and ligand-water, respectively), indicating weak contacts with a limited stabilizing effect.

**Figure 4:**
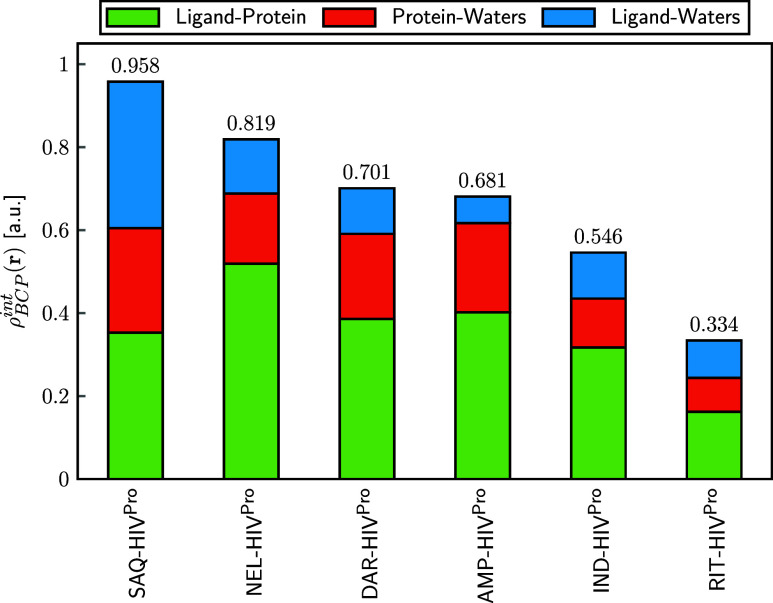
ρ_BCP_^int^(**r**) associated with noncovalent interactions in the studied Inh-HIV^Pro^ complexes. Contributions are categorized by interaction type: protein–ligand (green), protein-water (red-orange), and ligand-water (cyan). Values are reported in atomic units (a.u.), and correspond to the sum of all BCP densities identified in each interaction class.

When water-mediated contributions are excluded, the ranking shifts to NEL (0.519 a.u.) > AMP (0.402 a.u.) > DAR (0.386 a.u.) > SAQ (0.353 a.u.) > IND (0.317 a.u.) > RIT (0.162 a.u.). This inversion, particularly between AMP and DAR, highlights the relevance of structured water in modulating inhibitor affinity. Overall, these results indicate that water molecules not only complement direct protein–ligand interactions but can also play a decisive role in shaping experimental binding trends.

### 3.2 Residue-Level Contributions and Multivariate Regression Analysis

Given the lack of direct correlation between the total ρ_BCP_^int^(**r**) and the experimental interaction enthalpy, a more detailed analysis was performed at the residue level. Figures 5 and 6 show the electron density accumulated at BCPs associated with ligand-residue contacts for chains A and B of HIV^Pro^, respectively (numerical values are provided in Tables S3 and S4 in the SI). HIV^Pro^ is a dimeric aspartic protease in which the catalytic site is composed of two ASP 25 residues,^75^ one from each chain.^76^ Along with THR 26 and GLY 27, these residues define the active site and are known to play a critical role in inhibitor binding.^76,77^ Although THR 26 was included in the reduced models, no noncovalent ligand-residue interactions involving this residue were identified for any of the studied inhibitors, and it was therefore not considered in the residue-level QTAIM analysis. In contrast, all six inhibitors displayed measurable ρ_BCP_^int^(**r**) at ASP 25 in both chains and at GLY 27 in chain A. Additional recurrent contacts were identified with ALA 28, GLY 48, GLY 49, ILE 50, and ILE 84 in chain A, and with ALA 28, VAL 32, GLY 48, GLY 49, ILE 50, PRO 81, VAL 82, and ILE 84 in chain B. The accumulated density values suggest that inhibitors tend to interact more extensively with chain B. The residues showing the strongest contacts varied depending on the inhibitor: ILE 50 in AMP and RIT, ASP 29 in DAR, GLY 49 in IND, ASP 25 in NEL, and ASP 30 in SAQ in chain A; and ASP 25 (AMP, DAR), THR 80 (IND), ILE 50 (NEL, RIT), and GLY 27 (SAQ) in chain B.

**Figure 5:**
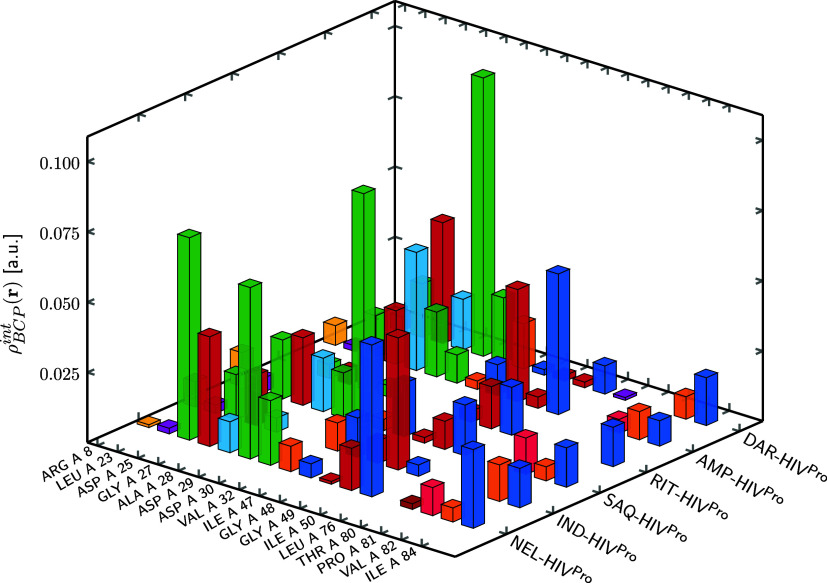
3D plot of the electron density at bond critical points associated with noncovalent interactions between the ligands and the residues forming the protein cavity in chain A of HIV^Pro^. Each bar represents the total ρ_BCP_^int^(**r**) value for a given ligand-residue pair. Values are expressed in atomic units (a.u.). Residues of the same type are shown using the same color.

**Figure 6:**
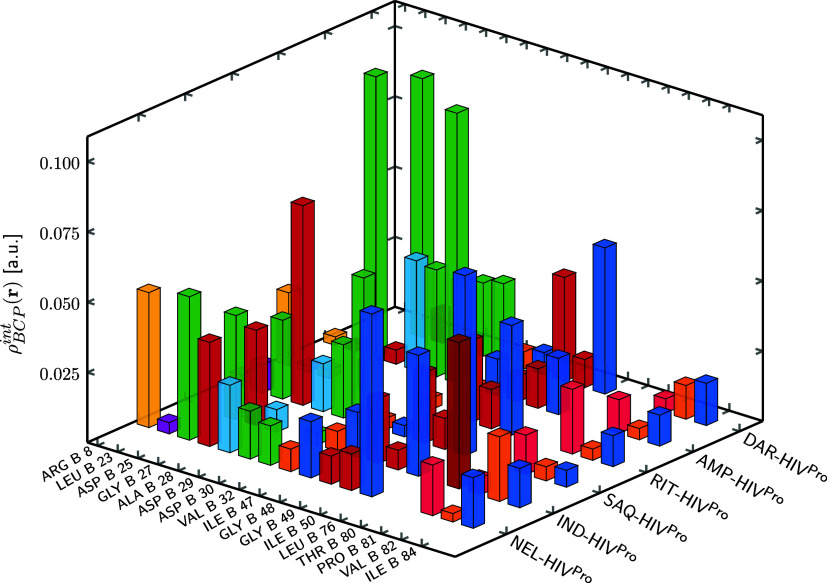
3D plot of the electron density at bond critical points associated with noncovalent interactions between the ligands and the residues forming the protein cavity in chain B of HIV^Pro^. Each bar represents the total ρ_BCP_^int^(**r**) value for a given ligand-residue pair. Values are expressed in atomic units (a.u.). Residues of the same type are shown using the same color.

To further investigate the role of water molecules, Figures 7 and 8 present heat maps of ρ_BCP_^int^(**r**) associated with water-residue contacts. White cells indicate the absence of interaction, while increasing shades of blue denote higher densities. These results show that water molecules generally establish more contacts with chain B than with chain A. In chain A (Figure 7), the most significant contributions are associated with aspartate and glycine residues. A similar pattern is observed in chain B (Figure 8), where water contacts are again mainly localized on aspartate and glycine residues. Notably, ILE 50 consistently engages in water-mediated contacts in both chains, except for DAR in chain B and DAR and RIT in chain A, underscoring its structural role as a hydration hotspot within the binding cavity.

**Figure 7:**
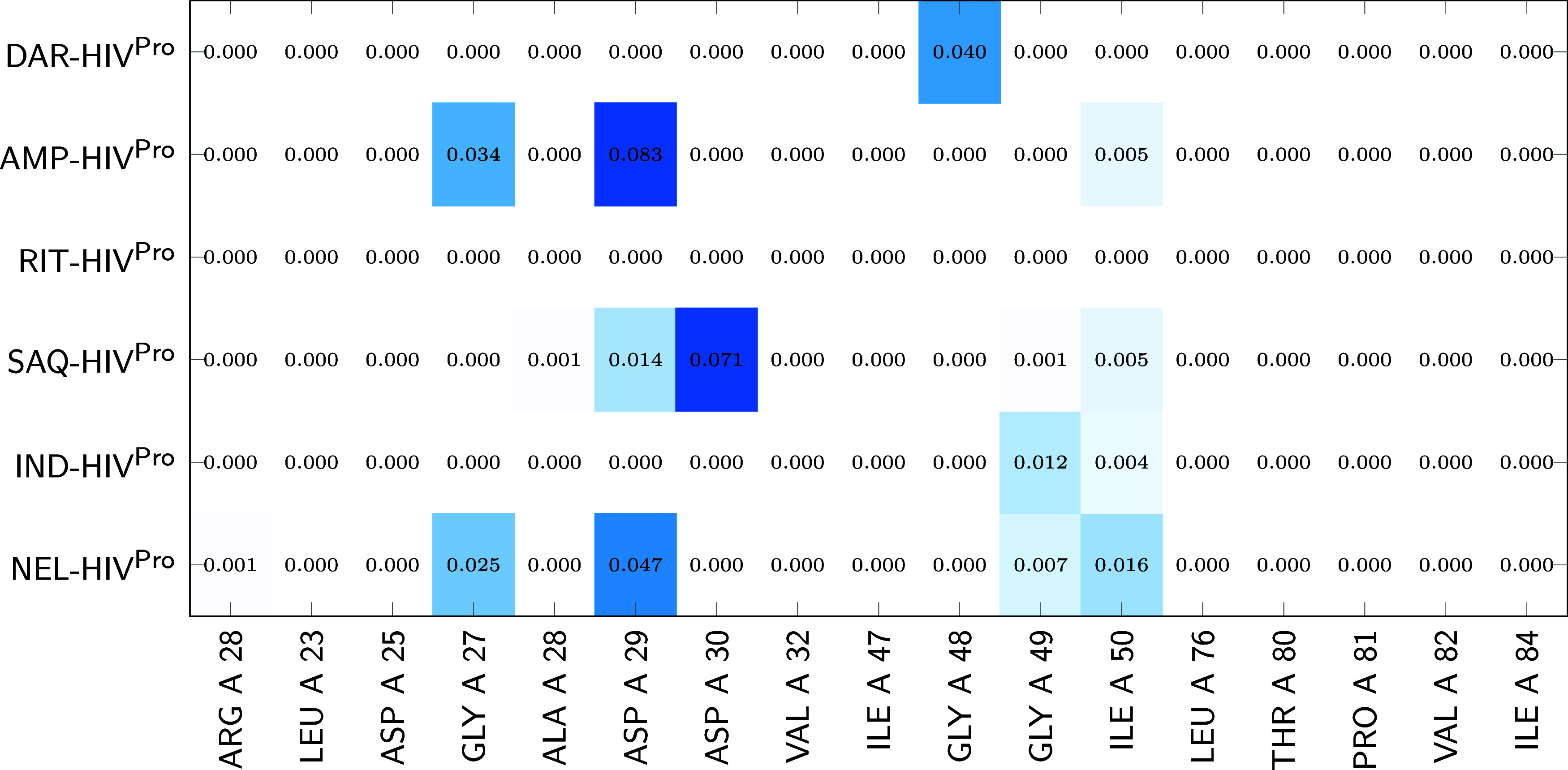
Heat map of ρ_BCP_^int^(**r**) associated with noncovalent interactions between water molecules and the residues forming the protein cavity in chain A of each reduced Inh-HIV^Pro^ complex. Color intensity ranges from 0 (white) to the global maximum of 0.083 a.u. (dark blue). Residues are shown along the *x*-axis, inhibitors along the *y*-axis, and values are reported in atomic units (a.u.).

**Figure 8:**
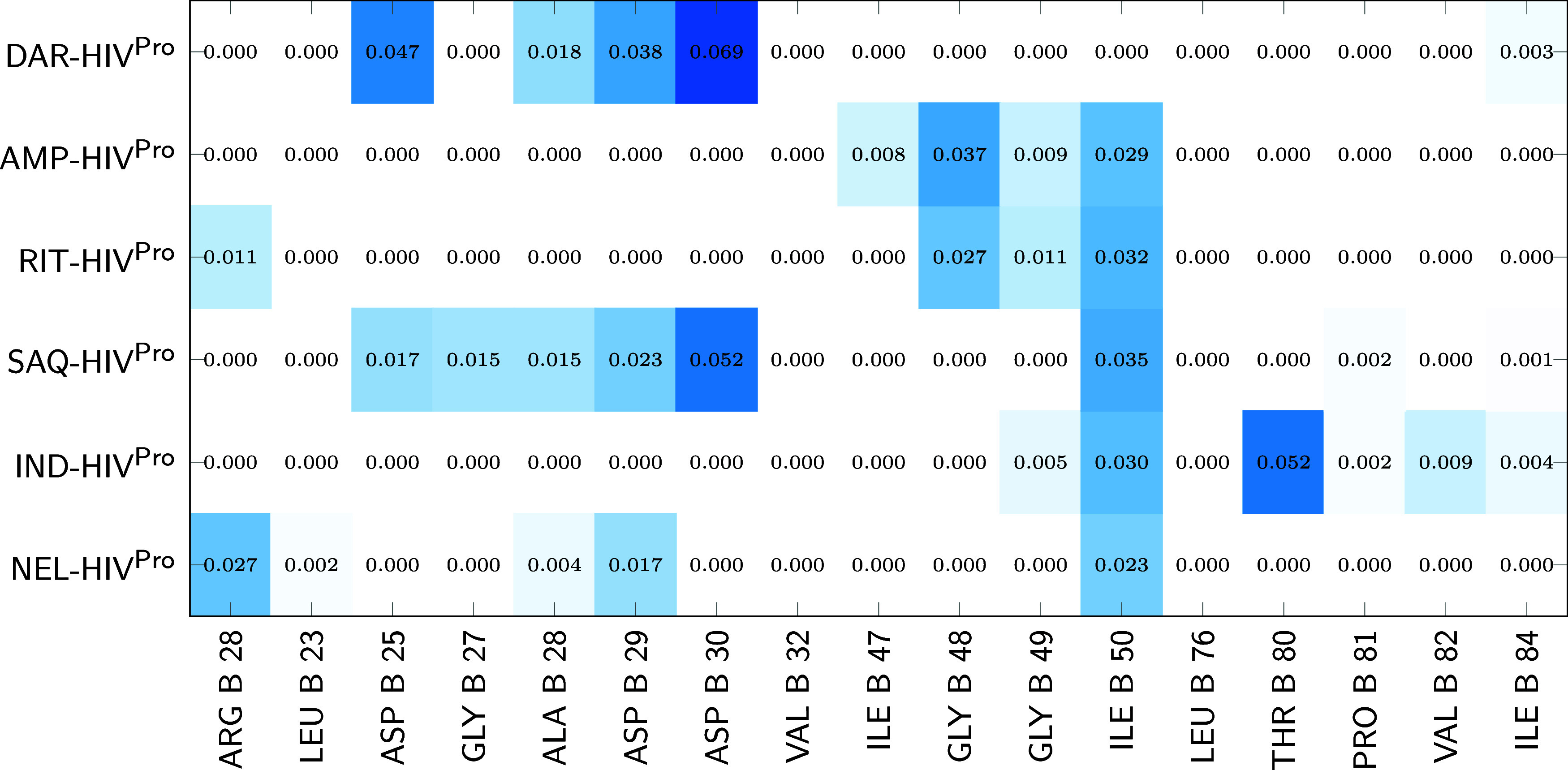
Heat map of ρ_BCP_^int^(**r**) associated with noncovalent interactions between water molecules and the residues forming the protein cavity in chain B of each reduced Inh-HIV^Pro^ complex. Color intensity ranges from 0 (white) to the global maximum of 0.069 a.u. (dark blue). Residues are shown along the *x*-axis, inhibitors along the *y*-axis, and values are reported in atomic units (a.u.).

Although neither the total accumulated ρ_BCP_^int^(**r**) nor the water-residue contributions exhibit a direct correlation with the experimental Δ_int_ *H*, their combined effects are implicitly reflected in the observed trends in interaction enthalpy. This behavior contrasts with previous QTAIM-based studies, such as that of Rojas et al.,^28^ who reported a strong correlation between ρ_BCP_^int^(**r**) and biological activity (*pK*_*i*_) for ligands targeting the dopamine D2 receptor. A key methodological difference lies in the construction of the reduced models. In addition to a geometric cutoff similar to that employed here, Rojas et al. applied an explicit energetic filter, retaining only those residues that significantly favor binding according to MM-PBSA calculations. As a result, their global descriptor inherently emphasizes attractive contributions and promotes additivity. In contrast, the present approach includes all residues within a defined geometric range, without energetic preselection, thereby allowing both favorable and unfavorable contributions to be identified and analyzed. To quantitatively explore these relationships, multivariate linear regression models were constructed using the experimental Δ_int_ *H* as the dependent variable and the residue-level ρ_BCP_^int^(**r**) as independent variables. No residues were excluded, based on the assumption that all contacts, even weak ones, could contribute to the total interaction energy. Due to the limited number of experimental data points in relation to the number of variables, the regression analysis is inherently exploratory and may be prone to overfitting. Nevertheless, it proposes a potentially transferable predictive model that allows for the identification and interpretation of cooperative and anticooperative residue-level contributions within a consistent electronic and structural framework.

Figure 9 presents the regression coefficients, where positive values (red) denote destabilizing contributions and negative values (blue) represent stabilizing effects. In this context, residues are classified as stabilizing or destabilizing solely on the basis of the sign and magnitude of their regression coefficients as defined by the multivariate model. The regression equations are provided in eqs (1) and (2) in the SI. Both models achieved *R* ^2^ = 1.00, an outcome attributed to overfitting from the small sample size. Nonetheless, the models remain useful for qualitative assessments and the identification of potential cooperative or anticooperative residue effects. The upper panel corresponds to the model including water-mediated contacts, while the lower panel corresponds to the model excluding them (removing water molecules but preserving the same protein–ligand geometry). Notable changes in regression coefficients were observed between the two models. For example, residues such as LEU B 23, GLY A 27, ALA B 28, ASP A 29, ILE B 47, GLY A 48, GLY B 48, and GLY A 49 exhibited reduced coefficients upon inclusion of water, consistent with cooperative effects. Conversely, residues including LEU A 23, ASP B 25, ALA A 28, ASP B 29, ASP A 30, ASP B 30, ILE A 50, ILE B 50, and THR B 80 showed increased coefficients, indicative of anticooperative effects. Interestingly, several of these residues do not establish strong direct water contacts (as shown in Figures 7 and 8), suggesting long-range effects and collective contributions from the hydration network.

**Figure 9:**
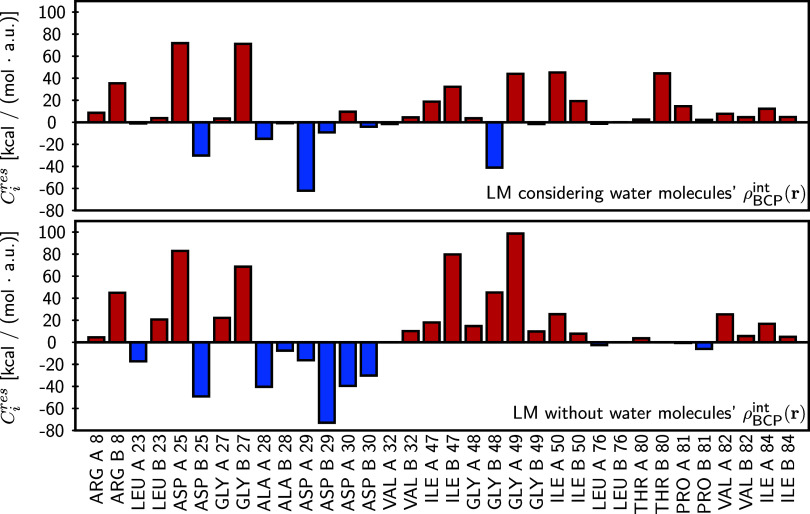
Regression coefficients obtained from the multivariable linear models (LM) correlating ρ_BCP_^int^(**r**) with the experimentally reported protein–ligand interaction enthalpy. The top panel shows the results considering BCPs associated with interactions involving water molecules, while the bottom panel excludes their contribution. LM expressions are in eq (1) and (2) of the SI. Residues along the protein cavity are shown on the *x*-axis. Positive coefficients (red bars) indicate a destabilizing contribution to the interaction enthalpy, whereas negative coefficients (blue bars) represent stabilizing effects.

Focusing on the model that includes water-mediated interactions (top panel of Figure 9), the residues with the most stabilizing contributions (|*C*_*i^res^*_| > 15 kcal/mol, where *C*_*i^res^*_ represents the residues’ coefficients of the multilinear model defined in eq (1) of the SI) are ASP B 25, ALA A 28, ASP A 29, and GLY B 48. In contrast, residues such as ARG B 8, ASP A 25, GLY B 27, ILE A 47, ILE B 47, GLY A 49, ILE A 50, ILE B 50, and THR B 80 contribute unfavorably. A comparison with the experimental order of affinities reveals a clear pattern. The most effective inhibitors, DAR and AMP, display relatively low electron density at residues identified by the regression model as destabilizing, such as ARG B 8, ASP A 25, ILE A 50, and ILE B 50. In contrast, they show stronger electron density at residues that contribute to stability, including GLY A 27, ASP B 25, and ASP B 29. On the other hand, inhibitors with lower affinities, like IND and NEL, exhibit significant electron density at the destabilizing residues identified by the regression model, which include ARG B 8, ASP A 25, GLY A 49, ILE A 50, ILE B 50, and THR B 80. Their density at stabilizing residues is comparatively lower. The intermediates, RIT and SAQ, present a mixed profile, exhibiting a balance between stabilizing and destabilizing contributions.

To further rationalize the stabilizing and destabilizing residue-level contributions identified by the multivariate model, we analyzed additional QTAIM-based descriptors that capture the local curvature and energetic balance of the electron density at intermolecular bond critical points. In particular, the second eigenvalue of the electron density Hessian, λ_2_, has been employed to classify noncovalent interactions, as its sign and magnitude reflect local electron density concentration or depletion perpendicular to the bond path.^78^ When combined with the electron density at the BCP, the product λ_2_ · ρ_BCP_ provides a physically meaningful metric to assess the attractive or nonattractive character of a contact, in close analogy to the color-mapping scheme used in the NCI index.^71^ In addition, the bond degree, defined as *H*/ρ_CP_,^79^ where *H* and ρ_*CP*_ are the total energy density and the electron density at the BCP, respectively, provides complementary insight into the interaction’s stabilizing or destabilizing nature, with more negative values indicating stronger stabilizing contributions.

Figure 10 illustrates the distribution of λ_2_ · ρ_BCP_ values for the noncovalent contacts associated with residues exhibiting the most significant regression coefficients (|*C*_*i^res^*_| ≥ 15 kcal/mol). The numerical values are provided in Tables S5–10 in the SI. For the most potent inhibitors, DAR and AMP, the strongest attractive interactions (more negative λ_2_ · ρ_BCP_ and bond degree values) are predominantly associated with residues that contribute favorably to the interaction enthalpy, such as ASP B 25 and ASP A 29. Notably, DAR shows a stronger interaction with ASP A 29 than AMP does, providing a physical rationale for the more favorable binding enthalpy of DAR despite their structural similarity. In contrast, for inhibitors with lower affinity, such as IND and NEL, the most pronounced interactions are frequently associated with residues identified by the regression model as enthalpically unfavorable.

**Figure 10:**
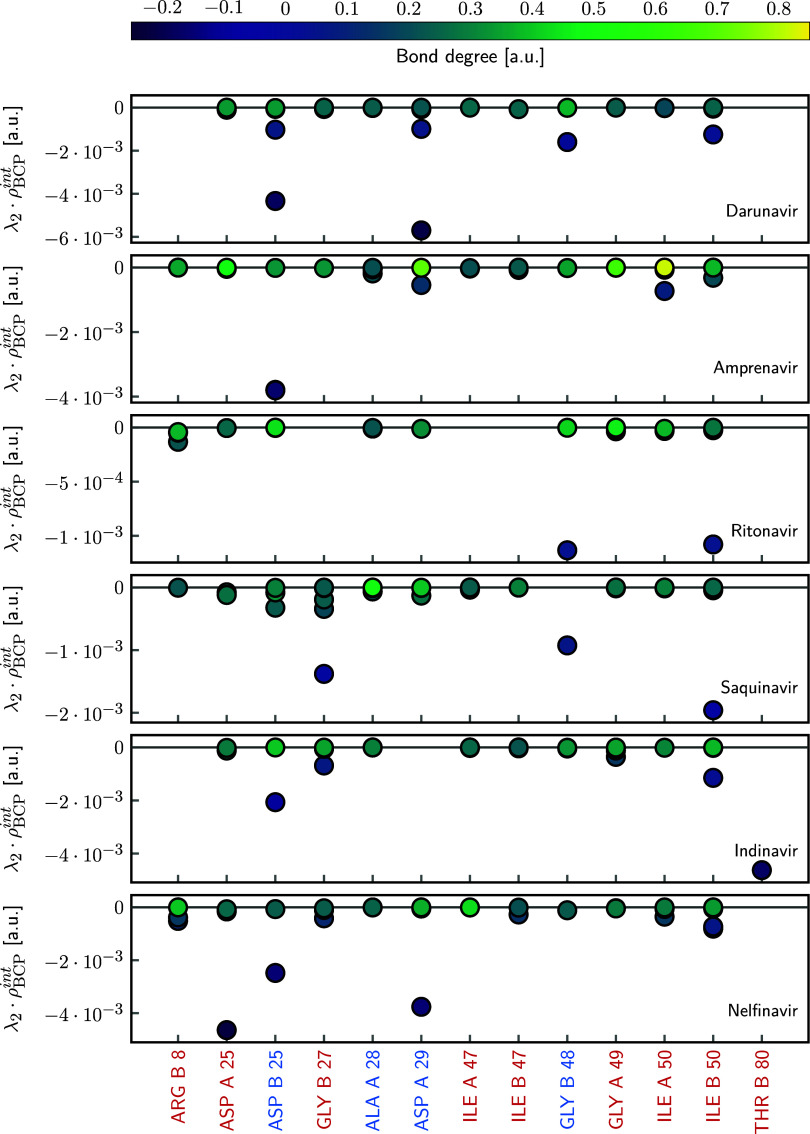
Product of λ_2_ and ρ_BCP_^int^(**r**) for noncovalent interactions in the Inh-HIV^Pro^ complexes. Each panel corresponds to a different inhibitor, ordered top to bottom by increasing experimental reaction enthalpy. Only residues with regression coefficients |*C*_*i^res^*_| > 15 kcal/mol (from the multivariable model, Figure 9) are shown. Residue labels are colored by their enthalpic contribution: blue for stabilizing, red for destabilizing. Data points are colored by bond degree.

This interpretation is further supported by NCI analysis of the DAR-HIV^Pro^ and NEL-HIV^Pro^ complexes (Figure 11). Although both inhibitors exhibit strong attractive interactions with ASP B 25, the NEL complex displays a partially nonattractive (red) NCI surface component in the same region. This feature is consistent with the presence of a strong interaction between NEL and ASP A 25 (as discussed before), which the multivariate model identifies as enthalpically unfavorable. Together, these observations suggest that residues contributing unfavorably to the interaction enthalpy may induce noncooperative effects on otherwise favorable contacts, thereby modulating the overall binding energetics through long-range electronic and geometric constraints.

**Figure 11:**
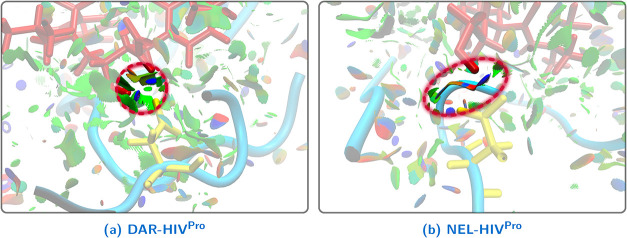
NCI surfaces (isovalue = 0.5 a.u.) centered on residue ASP B 25 in the (a) DAR-HIV^Pro^ and (b) NEL-HIV^Pro^ complexes. Inhibitors are shown in Licorice representation (red), residue ASP B 25 in Licorice (yellow), and the rest of the protein in NewCartoon (cyan). The NCI surface associated with the interaction between ASP B 25 and the corresponding inhibitor is highlighted and enclosed in red. The color scale ranges from −2.00 a.u. (blue, strongly attractive interactions) to 2.00 a.u. (red, strongly nonattractive interactions), with green indicating weak attractive interactions.

These results highlight that the quality of residue-specific contacts, whether they contribute favorably (cooperatively) or unfavorably (anticooperatively) to binding, is more determinant than the total number of noncovalent interactions. This perspective provides a conceptual framework for rational drug design: rather than maximizing the overall density of interactions, future inhibitors should be engineered to strengthen contacts with residues that contribute favorably while avoiding those with destabilizing effects, thereby enhancing binding affinity and specificity.

### 3.3 Comparison with MM-PBSA Residue Decomposition and Implications for Drug Design

To complement the findings from the multivariate regression model, we compared its results with the residue-level interaction free energies obtained from MM-PBSA calculations. Unlike MM-PBSA, which provides residue-level free energy decompositions derived from classical force-field terms, the present approach extracts residue-specific information directly from the electronic density. This quantum-topological framework captures both the directionality and cooperative nature of inter-residue interactions, enabling the explicit identification of stabilizing and destabilizing electronic effects that are averaged out in conventional energetic decompositions and therefore remain inaccessible to purely classical schemes. Given the MM-PBSA protocol used in this study, which incorporates implicit solvation and estimates entropy based on conformational fluctuations rather than complete configurational sampling, it can be assumed that the computed interaction free energies are predominantly enthalpic. Under this assumption, the regression coefficients from the multilinear model can be meaningfully compared with the per-residue MM-PBSA energy contributions. Furthermore, since MM-PBSA does not explicitly include solvent molecules, the regression model that excludes water-mediated BCPs (bottom panel of Figure 9) offers the most consistent basis for comparison. Differences in the sign or magnitude of specific contributions may arise from two sources: (1) the approximate treatment of entropy in MM-PBSA, and (2) the fact that QTAIM resolves all directional noncovalent contacts encoded in the electron density, including weak or nonclassical interactions that are not explicitly captured by classical force-field terms.

Figure 12 presents the per-residue decomposition of MM-PBSA interaction energies, with corresponding numerical values reported in Table S11 in the SI. The systems are arranged according to their experimental enthalpies, from most favorable (DAR) to least favorable (NEL). A coherent trend is observed: residues ARG A 8 and ARG B 8 generally exhibit unfavorable contributions to the interaction energy, consistent with the results from the regression model. Notably, DAR shows minimal interaction with these destabilizing residues (low absolute energy values), while NEL displays the strongest unfavorable contacts with both. In contrast, inhibitors with higher affinity, such as DAR and AMP, establish strong stabilizing interactions with key residues, including ASP B 25, ASP A 30, and ASP B 29, in agreement with the regression analysis. For other residues identified as unfavorable in the regression model, MM-PBSA yields only modest positive contributions, suggesting that entropic components may be more significant in those cases.

**Figure 12:**
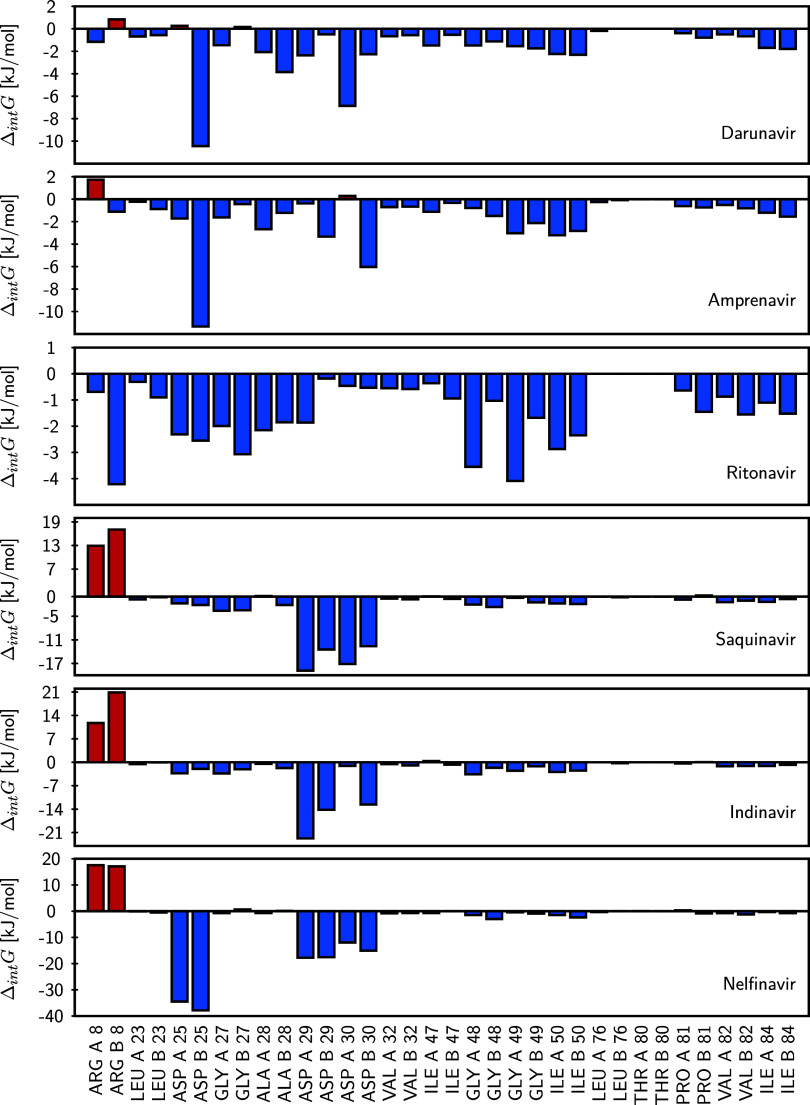
Per-residue free energy decomposition of protein–ligand interactions obtained from MM-PBSA analysis for each studied inhibitor. Bars represent the contribution of each residue forming the modeled cavity to the interaction free energy (Δ_int_ *G*) with the corresponding ligand. Stabilizing contributions are shown in blue, while destabilizing contributions are shown in red. The inhibitors are arranged from top to bottom in order of increasing experimental reaction enthalpy (from lowest to highest).

These findings emphasize that the affinity of HIV^Pro^ inhibitors is governed primarily by the nature and quality of residue-specific interactions, rather than by the total number of noncovalent contacts. Beyond confirming trends consistent with experimental enthalpies, our approach provides a mechanistically grounded framework for rational drug design. By combining quantum topological descriptors with statistical modeling, it becomes possible to identify which residues contribute favorably or unfavorably to ligand binding. This information can be exploited to optimize molecular recognition by selectively reinforcing interactions with stabilizing residues while minimizing contacts with destabilizing ones. Importantly, this strategy is not limited to structurally related compounds but may also apply to chemically diverse ligands targeting the same active site. In practice, the multivariate model is not intended as a standalone predictive tool trained on a limited data set, but rather as an interpretable framework that exploits physically grounded electron-density descriptors to identify stabilizing and destabilizing residue-level interactions, thereby enabling mechanistically informed ligand optimization. Moreover, this framework can be naturally extended as experimental data become available and are incorporated into the model, allowing integration with larger data sets or more advanced statistical and machine-learning approaches while preserving physical interpretability. This study, therefore, introduces a novel conceptual and computational strategy that explicitly distinguishes stabilizing from destabilizing effects at the residue level and can be integrated with existing energetic protocols in structure-guided ligand design.

## 4 Conclusions

Understanding the molecular determinants that govern ligand binding to protein targets remains a central challenge in rational drug design. In this study, we systematically characterized noncovalent interactions between HIV^Pro^ and six clinically approved inhibitors by combining experimental thermodynamic data with quantum-based atomistic descriptors. Our computational workflow integrated molecular dynamics simulations, semiempirical hydrogen-refinement, DFT single-point energy calculations, and topological analysis via QTAIM to quantify electron density at BCPs associated with protein–ligand contacts. This multiscale approach enabled a quantitative link between electronic structure and macroscopic thermodynamic behavior, providing mechanistic insight into the energetic basis of molecular recognition.

While the total accumulation of BCP electron density did not correlate directly with experimental binding enthalpies, a residue-resolved analysis revealed that affinity is governed not simply by the number or strength of noncovalent interactions, but by the qualitative nature of specific residue contributions. Multivariate regression models identified residues that cooperatively enhance or antagonize ligand binding, and the inclusion of water-mediated contacts further underscored the role of structured solvent in modulating these effects. Notably, these residue-level trends are physically rationalized by analyzing electron-density curvature, bond degree, and NCI index, which elucidate the attractive or nonattractive nature of the underlying contacts. Comparison with MM-PBSA per-residue free energies provided consistent trends, reinforcing the relevance of the quantum topological descriptors. Together, these results delineate a coherent energetic landscape that captures how local electronic features translate into global binding behavior.

Our findings establish a comprehensive framework for interpreting structure–activity relationships that goes beyond simply quantifying noncovalent interactions and their densities. By pinpointing stabilizing (cooperative) and destabilizing (anticooperative) interactions at the residue level, this approach provides actionable insights for the rational design of ligands with enhanced affinity and selectivity. Importantly, this analysis is independent of the structural similarities among the compounds studied, demonstrating that the identified electronic and energetic principles can be applied to chemically diverse ligands targeting the same binding site. Although our study focuses on HIV-1 protease, this methodology could be applicable to other protein–ligand systems as well, representing a versatile paradigm that connects electronic topology, thermodynamics, and biological function. Overall, this research demonstrates how integrating quantum chemical analysis, statistical modeling, and energetic data can advance structure-guided drug design by enriching our understanding of molecular recognition mechanisms. This framework may complement existing free-energy and decomposition techniques by incorporating residue-level electronic information unavailable to classical methods.

## Supplementary Material



## Notes

Tables reporting total electron density values at all intermolecular BCPs, protein–ligand and protein–water decompositions, residue-level QTAIM data, and MM-PBSA free-energy contributions; figures illustrating reduced protein–ligand models; multivariable models’ equations; and hydrogen-refined PDB structures of all reduced systems (PDF)
