# Supplementary material for: Residue-Level Affinity Decomposition via Quantum Electron Density: A Multivariable Framework Applied to HIV‑1 Protease Inhibitors
Source: J Phys Chem B. 2026 Apr 2;130(15):4080–93. doi: 10.1021/acs.jpcb.5c08041 (PMC13093483; doi:10.1021/acs.jpcb.5c08041)
Supplement: Supplementary file 1 [file jp5c08041_si_001.pdf]

Residue-Level Affinity Decomposition via Quantum Electron Density: A Multivariable Framework Applied to HIV-1 Protease Inhibitors

Supporting Information

Jorge Gutiérrez-Flores, Gerardo Padilla-Bernal, César Sánchez-Juárez, Dulce M. Méndez-Orduña, Javier Serrano Medina, Ponciano García-Gutiérrez, Rafael A. Zubillaga, Rubicelia Vargas*

Departamento de Química, División de Ciencias Básicas e Ingeniería, Universidad Autónoma Metropolitana Iztapalapa, San Rafael Atlixco 186, Col. Vicentina, C.P. 09340 Iztapalapa, CDMX, México; rvargas@izt.uam.mx

Contents

1 General features of the reduced systems and global distribution of non-covalent interactions **S2**

2 Residue-Level Contributions and Multivariate Regression Analysis **S4**

3 Comparison with MM-PBSA Residue Decomposition and Implications for Drug Design **S16**

4 Reduced Systems: Hydrogen-Refined Structures **S17**

4.1 AMP-HIV^{Pro} S17

4.2 DAR-HIV^{Pro} S37

4.3 IND-HIV^{Pro} S57

4.4 NEL-HIV^{Pro} S78

4.5 RIT-HIV^{Pro} S99

4.6 SAQ-HIV^{Pro} S120

1 General features of the reduced systems and global distribution of non-covalent interactions

Table S1. Electron density values at BCPs associated with non-covalent interactions ($\rho_{BCP}^{int}(\mathbf{r})$) identified in the reduced systems of the Inh-HIV^{Pro} complexes. The data include contributions from interactions involving water molecules present in each cavity. Electron densities are grouped by interaction type: conventional hydrogen bonds (HB), non-conventional HB, H–H interactions, and heteroatom-heteroatom (HA) interactions. The bottom row reports the total electron density ($\rho_{BCP}^{tot}(\mathbf{r})$) associated with all non-covalent interactions in each system. All values are expressed in atomic units (a.u.).

	AMP-HIV^{Pro}	DAR-HIV^{Pro}	IND-HIV^{Pro}	NEL-HIV^{Pro}	RIT-HIV^{Pro}	SAQ-HIV^{Pro}
Conventional HB[a]	0.363	0.402	0.217	0.364	0.130	0.591
Non-conventional HB[b]	0.161	0.208	0.210	0.228	0.117	0.198
H–H interactions[c]	0.137	0.083	0.115	0.213	0.064	0.146
HA interactions[d]	0.020	0.008	0.004	0.014	0.023	0.022
$\rho_{BCP}^{tot}(\mathbf{r})$	0.681	0.701	0.546	0.819	0.334	0.957

[a]Conventional HB include N–H$\cdots$N, N–H$\cdots$O, O–H$\cdots$N, and O–H$\cdots$O interactions.

[b]Non-conventional HB include C–H$\cdots\pi$, C–H$\cdots$C, C–H$\cdots$N, C–H$\cdots$O, C–H$\cdots$S, N–H$\cdots$C, and O–H$\cdots$C interactions.

[c]H–H interactions involve C–H$\cdots$H–C, C–H$\cdots$H–N, C–H$\cdots$H–O, and N–H$\cdots$H–O contacts.

[d]HA interactions include C$\cdots$C, C$\cdots$O, C$\cdots$N, N$\cdots$O, O$\cdots$O, and O$\cdots$S interactions.

Table S2. Total electron density at BCPs associated with non-covalent interactions in the studied Inh-HIV^{Pro} complexes. Values are classified into ligand-protein (LIG-PRT), protein-water (PRT-HOH), and ligand-water (LIG-HOH) contributions. All values are reported in atomic units (a.u.).

	AMP-HIV^{Pro}	DAR-HIV^{Pro}	IND-HIV^{Pro}	NEL-HIV^{Pro}	RIT-HIV^{Pro}	SAQ-HIV^{Pro}
LIG-PRT	0.402	0.386	0.317	0.519	0.162	0.353
PRT-HOH	0.215	0.205	0.118	0.169	0.082	0.252
LIG-HOH	0.064	0.110	0.111	0.131	0.090	0.352
$\rho_{BCP}^{tot}(\mathbf{r})$	0.681	0.701	0.546	0.819	0.334	0.957

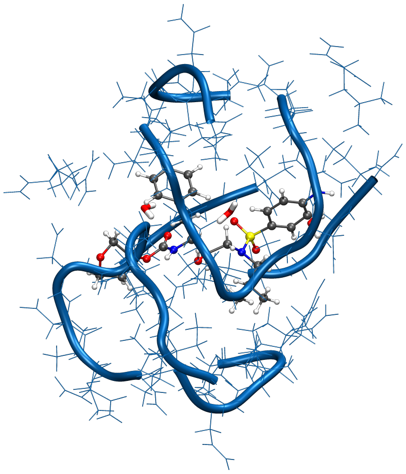

AMP-HIV^{Pro} reduced system

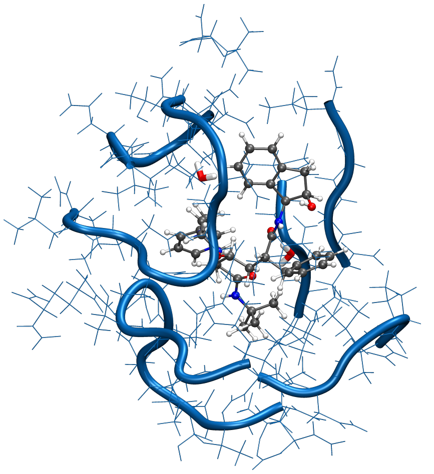

IND-HIV^{Pro} reduced system

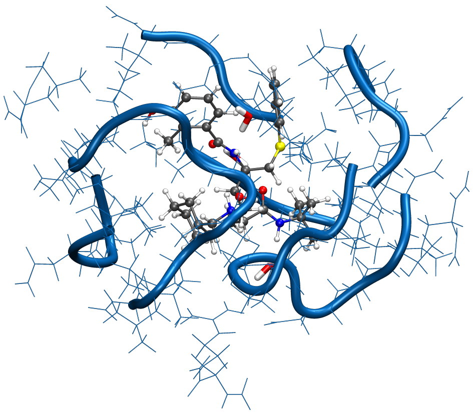

NEL-HIV^{Pro} reduced system

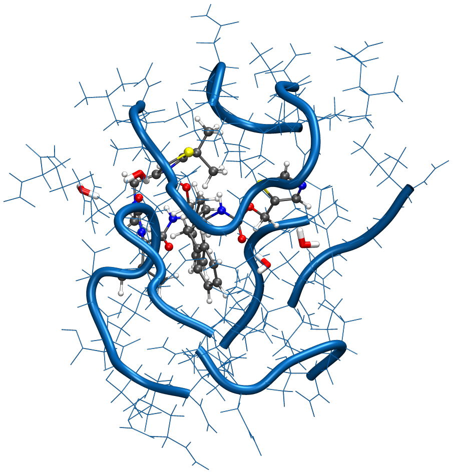

RIT-HIV^{Pro} reduced system

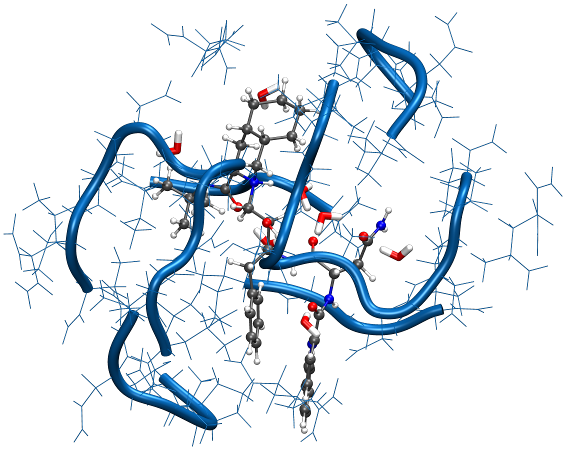

SAQ-HIV^{Pro} reduced system

Figure S1. Reduced systems of the HIV^{Pro} complexes with amprenavir (AMP), indinavir (IND), nelfinavir (NEL), ritonavir (RIT), and saquinavir (SAQ). All systems share the same reduced amino acid sequence for the protease. Inhibitors are shown in CPK representation, water molecules in Licorice, and the protease in Cartoon style with residues as blue lines. Atom colors: gray (C), red (O), blue (N), yellow (S), and white (H).

2 Residue-Level Contributions and Multivariate Regression Analysis

Table S3. $\rho_{BCP}^{int}(\mathbf{r})$ of non-covalent interactions between the ligands and individual residues forming the protein cavity in chain A of HIV^{Pro}. Values are reported in atomic units (a.u.).

	DAR-HIV^{Pro}	AMP-HIV^{Pro}	RIT-HIV^{Pro}	SAQ-HIV^{Pro}	IND-HIV^{Pro}	NEL-HIV^{Pro}
ARG A 8	0.000	0.007	0.000	0.012	0.009	0.001
LEU A 23	0.005	0.002	0.000	0.005	0.003	0.002
ASP A 25	0.019	0.015	0.005	0.021	0.016	0.072
GLY A 27	0.043	0.019	0.005	0.024	0.018	0.039
ALA A 28	0.018	0.042	0.014	0.019	0.005	0.011
ASP A 29	0.099	0.023	0.004	0.016	0.000	0.061
ASP A 30	0.023	0.010	0.000	0.082	0.000	0.023
VAL A 32	0.016	0.003	0.000	0.004	0.010	0.009
ILE A 47	0.002	0.011	0.000	0.019	0.014	0.005
GLY A 48	0.002	0.040	0.004	0.002	0.008	0.001
GLY A 49	0.002	0.004	0.015	0.010	0.047	0.015
ILE A 50	0.010	0.050	0.017	0.018	0.004	0.054
LEU A 76	0.001	0.000	0.000	0.000	0.000	0.000
THR A 80	0.000	0.000	0.000	0.000	0.000	0.002
PRO A 81	0.000	0.005	0.000	0.013	0.000	0.010
VAL A 82	0.008	0.010	0.000	0.005	0.013	0.005
ILE A 84	0.017	0.009	0.014	0.014	0.014	0.028

Table S4. $\rho^{int}_{BCP}(\mathbf{r})$ of non-covalent interactions between the ligands and individual residues forming the protein cavity in chain B of HIV^{Pro}. Values are reported in atomic units (a.u.).

	DAR-HIV^{Pro}	AMP-HIV^{Pro}	RIT-HIV^{Pro}	SAQ-HIV^{Pro}	IND-HIV^{Pro}	NEL-HIV^{Pro}
ARG B 8	0.000	0.003	0.026	0.004	0.000	0.048
LEU B 23	0.000	0.002	0.002	0.010	0.000	0.004
ASP B 25	0.092	0.100	0.003	0.028	0.037	0.051
GLY B 27	0.011	0.005	0.000	0.071	0.034	0.037
ALA B 28	0.024	0.039	0.010	0.017	0.008	0.024
ASP B 29	0.026	0.038	0.000	0.026	0.000	0.017
ASP B 30	0.028	0.096	0.000	0.052	0.004	0.014
VAL B 32	0.006	0.001	0.004	0.004	0.007	0.008
ILE B 47	0.008	0.013	0.000	0.004	0.016	0.020
GLY B 48	0.037	0.009	0.029	0.025	0.023	0.010
GLY B 49	0.010	0.014	0.014	0.011	0.007	0.013
ILE B 50	0.052	0.020	0.039	0.064	0.043	0.065
LEU B 76	0.000	0.000	0.000	0.000	0.000	0.000
THR B 80	0.000	0.000	0.000	0.000	0.052	0.000
PRO B 81	0.005	0.012	0.023	0.014	0.006	0.018
VAL B 82	0.012	0.004	0.004	0.005	0.023	0.003
ILE B 84	0.015	0.011	0.011	0.006	0.014	0.018

LM considering water molecules' $\rho^{\text{int}}_{\text{BCP}}(\mathbf{r})$:

$$\begin{aligned}\Delta_{int}H = {} & 8.60\rho^{\text{int}}_{\text{ARG A 8}} + 35.37\rho^{\text{int}}_{\text{ARG B 8}} - 1.01\rho^{\text{int}}_{\text{LEU A 23}} + 3.84\rho^{\text{int}}_{\text{LEU B 23}} + 71.80\rho^{\text{int}}_{\text{ASP A 25}} \\ & - 30.15\rho^{\text{int}}_{\text{ASP B 25}} + 3.42\rho^{\text{int}}_{\text{GLY A 27}} + 71.20\rho^{\text{int}}_{\text{GLY B 27}} - 14.94\rho^{\text{int}}_{\text{ALA A 28}} - 0.79\rho^{\text{int}}_{\text{ALA B 28}} \\ & - 62.04\rho^{\text{int}}_{\text{ASP A 29}} - 9.16\rho^{\text{int}}_{\text{ASP B 29}} + 9.60\rho^{\text{int}}_{\text{ASP A 30}} - 4.05\rho^{\text{int}}_{\text{ASP B 30}} - 1.53\rho^{\text{int}}_{\text{VAL A 32}} \\ & + 4.48\rho^{\text{int}}_{\text{VAL B 32}} + 18.08\rho^{\text{int}}_{\text{ILE A 47}} + 28.25\rho^{\text{int}}_{\text{ILE B 47}} + 3.68\rho^{\text{int}}_{\text{GLY A 48}} - 41.09\rho^{\text{int}}_{\text{GLY B 48}} \\ & + 44.02\rho^{\text{int}}_{\text{GLY A 49}} - 1.58\rho^{\text{int}}_{\text{GLY B 49}} + 45.24\rho^{\text{int}}_{\text{ILE A 50}} + 19.19\rho^{\text{int}}_{\text{ILE B 50}} - 1.38\rho^{\text{int}}_{\text{LEU A 76}} \\ & + 0.00\rho^{\text{int}}_{\text{LEU B 76}} + 2.44\rho^{\text{int}}_{\text{THR A 80}} + 44.37\rho^{\text{int}}_{\text{THR B 80}} + 14.64\rho^{\text{int}}_{\text{PRO A 81}} + 2.17\rho^{\text{int}}_{\text{PRO B 81}} \\ & + 7.68\rho^{\text{int}}_{\text{VAL A 82}} + 4.62\rho^{\text{int}}_{\text{VAL B 82}} + 12.32\rho^{\text{int}}_{\text{ILE A 84}} + 4.84\rho^{\text{int}}_{\text{ILE B 84}} - 6.34\end{aligned} \tag{1}$$

LM without water molecules' $\rho^{\text{int}}_{\text{BCP}}(\mathbf{r})$:

$$\begin{aligned}\Delta_{int}H = {} & 12.68\rho^{\text{int}}_{\text{ARG A 8}} + 12.13\rho^{\text{int}}_{\text{ARG B 8}} - 7.04\rho^{\text{int}}_{\text{LEU A 23}} - 10.45\rho^{\text{int}}_{\text{LEU B 23}} + 87.90\rho^{\text{int}}_{\text{ASP A 25}} \\ & - 52.42\rho^{\text{int}}_{\text{ASP B 25}} + 21.13\rho^{\text{int}}_{\text{GLY A 27}} + 61.74\rho^{\text{int}}_{\text{GLY B 27}} - 43.73\rho^{\text{int}}_{\text{ALA A 28}} - 15.46\rho^{\text{int}}_{\text{ALA B 28}} \\ & - 18.17\rho^{\text{int}}_{\text{ASP A 29}} - 77.76\rho^{\text{int}}_{\text{ASP B 29}} - 34.94\rho^{\text{int}}_{\text{ASP A 30}} - 33.82\rho^{\text{int}}_{\text{ASP B 30}} - 2.90\rho^{\text{int}}_{\text{VAL A 32}} \\ & + 9.37\rho^{\text{int}}_{\text{VAL B 32}} + 24.68\rho^{\text{int}}_{\text{ILE A 47}} + 68.26\rho^{\text{int}}_{\text{ILE B 47}} + 13.72\rho^{\text{int}}_{\text{GLY A 48}} + 48.24\rho^{\text{int}}_{\text{GLY B 48}} \\ & + 94.20\rho^{\text{int}}_{\text{GLY A 49}} + 18.01\rho^{\text{int}}_{\text{GLY B 49}} + 25.39\rho^{\text{int}}_{\text{ILE A 50}} + 10.43\rho^{\text{int}}_{\text{ILE B 50}} - 2.64\rho^{\text{int}}_{\text{LEU A 76}} \\ & + 0.00\rho^{\text{int}}_{\text{LEU B 76}} + 3.26\rho^{\text{int}}_{\text{THR A 80}} + 0.00\rho^{\text{int}}_{\text{THR B 80}} + 1.19\rho^{\text{int}}_{\text{PRO A 81}} - 4.36\rho^{\text{int}}_{\text{PRO B 81}} \\ & + 20.59\rho^{\text{int}}_{\text{VAL A 82}} + 5.06\rho^{\text{int}}_{\text{VAL B 82}} + 16.58\rho^{\text{int}}_{\text{ILE A 84}} + 3.55\rho^{\text{int}}_{\text{ILE B 84}} - 6.34\end{aligned} \tag{2}$$

Residues that lower interaction enthalpy ($\Delta_{int}H$) are marked in blue, while those that raise $\Delta_{int}H$ are marked in red.

Table S5. Topological and bonding descriptors of non-covalent interactions for residues with $|c_i| > 15$ kcal/mol in Equation 1, corresponding to the DAR-HIV^{Pro} system. For each BCP, the electron density ($\rho_{BCP}^{int}(\mathbf{r})$), the second eigenvalue of the Hessian (λ_2), their product ($\rho_{BCP}^{int}(\mathbf{r}) \cdot \lambda_2$), and the bond degree are reported. Values are expressed in atomic units (a.u.).

	$\rho_{BCP}^{int}(\mathbf{r})$	λ_2	$\rho_{BCP}^{int}(\mathbf{r}) \cdot \lambda_2$	Bond degree
ASP A 25	0.010	-0.010	-0.000100	0.23
	0.007	-0.005	-0.000035	0.31
	0.002	-0.001	-0.000002	0.34
ASP B 25	0.051	-0.085	-0.004335	-0.18
	0.027	-0.038	-0.001026	0.06
	0.009	-0.007	-0.000063	0.28
	0.005	-0.004	-0.000020	0.34
GLY B 27	0.008	-0.008	-0.000064	0.28
	0.003	-0.001	-0.000003	0.25
ALA A 28	0.010	-0.002	-0.000020	0.12
	0.003	-0.002	-0.000006	0.23
	0.003	-0.001	-0.000003	0.24
	0.002	-0.001	-0.000002	0.24
ASP A 29	0.057	-0.100	-0.005700	-0.21
	0.026	-0.038	-0.000988	0.05
	0.009	-0.008	-0.000072	0.26
	0.004	-0.003	-0.000012	0.35
	0.003	-0.002	-0.000006	0.22
ILE A 47	0.002	-0.001	-0.000002	0.26
ILE B 47	0.008	-0.009	-0.000072	0.25
GLY B 48	0.032	-0.050	-0.001600	-0.01
	0.005	-0.002	-0.000010	0.38
GLY A 49	0.002	-0.001	-0.000002	0.24
ILE A 50	0.005	-0.005	-0.000025	0.30
	0.005	-0.003	-0.000015	0.19

Continue on the next page

Table S5. (Continued.)

ILE B 50	0.029	-0.043	-0.001247	0.02
	0.007	-0.007	-0.000049	0.31
	0.006	-0.005	-0.000030	0.21
	0.005	-0.003	-0.000015	0.33
	0.003	-0.001	-0.000003	0.22
	0.002	-0.001	-0.000002	0.25

Table S6. Topological and bonding descriptors of non-covalent interactions for residues with $|c_i| > 15$ kcal/mol in Equation 1, corresponding to the AMP-HIV^{Pro} system. For each BCP, the electron density ($\rho_{BCP}^{int}(\mathbf{r})$), the second eigenvalue of the Hessian (λ_2), their product ($\rho_{BCP}^{int}(\mathbf{r}) \cdot \lambda_2$), and the bond degree are reported. Values are expressed in atomic units (a.u.).

	$\rho_{BCP}^{int}(\mathbf{r})$	λ_2	$\rho_{BCP}^{int}(\mathbf{r}) \cdot \lambda_2$	Bond degree
ARG B 8	0.001	-0.001	-0.000001	0.41
	0.001	-0.001	-0.000001	0.36
	0.001	0.000	0.000000	0.36
ASP A 25	0.010	-0.004	-0.000040	0.36
	0.005	-0.001	-0.000005	0.49
ASP B 25	0.048	-0.079	-0.003792	-0.16
	0.047	-0.081	-0.003807	-0.16
	0.005	-0.002	-0.000010	0.33
GLY B 27	0.005	-0.003	-0.000015	0.33
ALA A 28	0.013	-0.014	-0.000182	0.21
	0.008	-0.007	-0.000056	0.28
	0.008	-0.006	-0.000048	0.30
	0.005	-0.003	-0.000015	0.26
	0.004	-0.003	-0.000012	0.33
	0.004	-0.002	-0.000008	0.21
ASP A 29	0.020	-0.027	-0.000540	0.13
	0.003	-0.002	-0.000006	0.71

Continue on the next page

Table S6. (Continued.)

ILE A 47	0.006	-0.005	-0.000030	0.20
	0.005	-0.003	-0.000015	0.21
ILE B 47	0.009	-0.008	-0.000072	0.18
	0.002	-0.002	-0.000004	0.28
	0.002	-0.001	-0.000002	0.23
GLY B 48	0.005	-0.003	-0.000015	0.32
	0.004	-0.003	-0.000012	0.35
GLY A 49	0.003	-0.002	-0.000006	0.22
	0.001	0.000	0.000000	0.66
ILE A 50	0.022	-0.033	-0.000726	0.08
	0.011	-0.006	-0.000066	0.12
	0.007	-0.004	-0.000028	0.13
	0.004	-0.003	-0.000012	0.23
	0.003	-0.001	-0.000003	0.23
	0.002	-0.001	-0.000002	0.49
	0.001	-0.001	-0.000001	0.82
ILE B 50	0.015	-0.021	-0.000315	0.20
	0.002	-0.001	-0.000002	0.24
	0.001	-0.001	-0.000001	0.43
	0.001	-0.001	-0.000001	0.29
	0.001	0.000	0.000000	0.38

Table S7. Topological and bonding descriptors of non-covalent interactions for residues with $|c_i| > 15$ kcal/mol in Equation 1, corresponding to the RIT-HIV^{Pro} system. For each BCP, the electron density ($\rho_{BCP}^{int}(\mathbf{r})$), the second eigenvalue of the Hessian (λ_2), their product ($\rho_{BCP}^{int}(\mathbf{r}) \cdot \lambda_2$), and the bond degree are reported. Values are expressed in atomic units (a.u.).

	$\rho_{BCP}^{int}(\mathbf{r})$	λ_2	$\rho_{BCP}^{int}(\mathbf{r}) \cdot \lambda_2$	Bond degree
ARG B 8	0.011	-0.012	-0.000132	0.22
	0.008	-0.006	-0.000048	0.20

Continue on the next page

Table S7. (Continued.)

	0.007	-0.006	-0.000042	0.38
ASP A 25	0.005	-0.001	-0.000005	0.26
ALA A 28	0.002	-0.002	-0.000004	0.40
	0.001	-0.001	-0.000001	0.44
ALA A 28	0.005	-0.002	-0.000010	0.17
	0.005	-0.002	-0.000010	0.26
	0.004	-0.001	-0.000004	0.22
ASP A 29	0.004	-0.003	-0.000012	0.32
GLY B 48	0.027	-0.042	-0.001134	0.04
	0.002	-0.001	-0.000002	0.42
GLY A 49	0.007	-0.005	-0.000035	0.22
	0.006	-0.003	-0.000018	0.22
	0.001	0.000	0.000000	0.29
	0.001	-0.001	-0.000001	0.46
ILE A 50	0.008	-0.004	-0.000032	0.18
	0.005	-0.003	-0.000015	0.20
	0.004	-0.003	-0.000012	0.38
ILE B 50	0.027	-0.040	-0.001080	0.04
	0.005	-0.005	-0.000025	0.22
	0.005	-0.003	-0.000015	0.29
	0.002	-0.001	-0.000002	0.28

Table S8. Topological and bonding descriptors of non-covalent interactions for residues with $|c_i| > 15$ kcal/mol in Equation 1, corresponding to the SAQ-HIV^{Pro} system. For each BCP, the electron density ($\rho_{BCP}^{int}(\mathbf{r})$), the second eigenvalue of the Hessian (λ_2), their product ($\rho_{BCP}^{int}(\mathbf{r}) \cdot \lambda_2$), and the bond degree are reported. Values are expressed in atomic units (a.u.).

	$\rho_{BCP}^{int}(\mathbf{r})$	λ_2	$\rho_{BCP}^{int}(\mathbf{r}) \cdot \lambda_2$	Bond degree
ARG B 8	0.004	-0.001	-0.000004	0.22
ASP A 25	0.011	-0.007	-0.000077	0.24

Continue on the next page

Table S8. (Continued.)

	0.010	-0.012	-0.000120	0.27
ASP B 25	0.017	-0.019	-0.000323	0.23
	0.008	-0.010	-0.000080	0.33
	0.003	-0.002	-0.000006	0.30
GLY B 27	0.030	-0.046	-0.001380	-0.02
	0.019	-0.018	-0.000342	0.19
	0.012	-0.016	-0.000192	0.24
	0.005	-0.002	-0.000010	0.33
	0.003	-0.002	-0.000006	0.35
	0.002	-0.001	-0.000002	0.23
ALA A 28	0.008	-0.008	-0.000064	0.20
	0.006	-0.002	-0.000012	0.23
	0.004	-0.002	-0.000008	0.21
	0.001	-0.001	-0.000001	0.50
ASP A 29	0.010	-0.013	-0.000130	0.27
	0.004	-0.002	-0.000008	0.32
	0.002	-0.002	-0.000004	0.40
ILE A 47	0.007	-0.005	-0.000035	0.19
	0.004	-0.002	-0.000008	0.20
	0.003	-0.002	-0.000006	0.23
	0.003	-0.002	-0.000006	0.22
	0.002	0.000	0.000000	0.24
ILE B 47	0.003	-0.001	-0.000003	0.32
	0.001	0.000	0.000000	0.30
GLY B 48	0.025	-0.037	-0.000925	0.06
GLY A 49	0.007	-0.002	-0.000014	0.20
	0.002	-0.001	-0.000002	0.38
	0.001	0.000	0.000000	0.27
ILE A 50	0.005	-0.003	-0.000015	0.26
	0.004	-0.003	-0.000012	0.36

Continue on the next page

Table S8. (Continued.)

	0.004	-0.003	-0.000012	0.29
	0.004	-0.003	-0.000012	0.20
	0.001	-0.001	-0.000001	0.30
ILE B 50	0.035	-0.056	-0.001960	-0.05
	0.009	-0.005	-0.000045	0.15
	0.005	-0.001	-0.000005	0.18
	0.004	-0.002	-0.000008	0.20
	0.003	-0.002	-0.000006	0.22
	0.003	-0.002	-0.000006	0.22
	0.003	-0.001	-0.000003	0.24
	0.002	-0.001	-0.000002	0.26

Table S9. Topological and bonding descriptors of non-covalent interactions for residues with $|c_i| > 15$ kcal/mol in Equation 1, corresponding to the IND-HIV^{Pro} system. For each BCP, the electron density ($\rho_{BCP}^{int}(\mathbf{r})$), the second eigenvalue of the Hessian (λ_2), their product ($\rho_{BCP}^{int}(\mathbf{r}) \cdot \lambda_2$), and the bond degree are reported. Values are expressed in atomic units (a.u.).

	$\rho_{BCP}^{int}(\mathbf{r})$	λ_2	$\rho_{BCP}^{int}(\mathbf{r}) \cdot \lambda_2$	Bond degree
ASP A 25	0.010	-0.012	-0.000120	0.27
	0.006	-0.003	-0.000018	0.29
ASP B 25	0.035	-0.059	-0.002065	-0.09
	0.002	-0.001	-0.000002	0.40
GLY B 27	0.022	-0.031	-0.000682	0.07
	0.008	-0.007	-0.000056	0.26
	0.004	-0.002	-0.000008	0.35
ALA A 28	0.003	-0.002	-0.000006	0.22
	0.001	-0.001	-0.000001	0.26
	0.001	0.000	0.000000	0.30
ILE A 47	0.005	-0.004	-0.000020	0.22
	0.005	-0.004	-0.000020	0.21

Continue on the next page

Table S9. (Continued.)

	0.002	-0.001	-0.000002	0.22
	0.002	-0.001	-0.000002	0.26
ILE B 47	0.006	-0.006	-0.000036	0.19
	0.004	-0.002	-0.000008	0.20
	0.003	0.000	0.000000	0.25
	0.003	-0.001	-0.000003	0.22
GLY B 48	0.007	-0.006	-0.000042	0.26
	0.006	-0.002	-0.000012	0.29
	0.005	-0.004	-0.000020	0.33
	0.005	-0.003	-0.000015	0.33
GLY A 49	0.017	-0.021	-0.000357	0.16
	0.012	-0.010	-0.000120	0.22
	0.007	-0.006	-0.000042	0.28
	0.004	-0.002	-0.000008	0.20
	0.004	-0.002	-0.000008	0.35
	0.003	-0.002	-0.000006	0.35
ILE A 50	0.004	-0.003	-0.000012	0.31
ILE B 50	0.028	-0.041	-0.001148	0.03
	0.006	-0.003	-0.000018	0.18
	0.004	-0.003	-0.000012	0.21
	0.003	-0.001	-0.000003	0.22
	0.002	-0.001	-0.000002	0.38
THR B 80	0.052	-0.089	-0.004628	-0.17

Table S10. Topological and bonding descriptors of non-covalent interactions for residues with $|c_i| > 15$ kcal/mol in Equation 1, corresponding to the NEL-HIV^{Pro} system. For each BCP, the electron density ($\rho^{int}_{BCP}(\mathbf{r})$), the second eigenvalue of the Hessian (λ_2), their product ($\rho^{int}_{BCP}(\mathbf{r}) \cdot \lambda_2$), and the bond degree are reported. Values are expressed in atomic units (a.u.).

	$\rho^{int}_{BCP}(\mathbf{r})$	λ_2	$\rho^{int}_{BCP}(\mathbf{r}) \cdot \lambda_2$	Bond degree
ARG B 8	0.027	-0.019	-0.000513	0.11
	0.020	-0.019	-0.000380	0.16
	0.001	0.000	0.000000	0.40
ASP A 25	0.051	-0.091	-0.004641	-0.22
	0.012	-0.013	-0.000156	0.24
	0.009	-0.008	-0.000072	0.26
ASP B 25	0.040	-0.062	-0.002480	-0.15
	0.011	-0.006	-0.000066	0.24
GLY B 27	0.018	-0.023	-0.000414	0.12
	0.010	-0.010	-0.000100	0.25
	0.009	-0.004	-0.000036	0.24
ALA A 28	0.005	-0.001	-0.000005	0.20
	0.003	-0.001	-0.000003	0.23
	0.003	-0.001	-0.000003	0.25
ASP A 29	0.047	-0.080	-0.003760	-0.15
	0.009	-0.005	-0.000045	0.16
	0.004	-0.003	-0.000012	0.20
	0.001	0.000	0.000000	0.37
ILE A 47	0.003	-0.002	-0.000006	0.20
	0.002	-0.001	-0.000002	0.43
ILE B 47	0.015	-0.018	-0.000270	0.14
	0.005	-0.002	-0.000010	0.21
GLY B 48	0.010	-0.011	-0.000110	0.23
GLY A 49	0.008	-0.004	-0.000032	0.17
	0.007	-0.006	-0.000042	0.29

Continue on the next page

Table S10. (Continued.)

ILE A 50	0.016	-0.022	-0.000352	0.16
	0.009	-0.007	-0.000063	0.14
	0.007	-0.004	-0.000028	0.24
	0.005	-0.003	-0.000015	0.20
	0.005	-0.003	-0.000015	0.21
	0.005	-0.003	-0.000015	0.19
	0.005	-0.001	-0.000005	0.20
	0.002	-0.001	-0.000002	0.29
ILE B 50	0.023	-0.035	-0.000805	0.07
	0.022	-0.033	-0.000726	0.06
	0.010	-0.005	-0.000050	0.16
	0.006	-0.002	-0.000012	0.21
	0.002	-0.001	-0.000002	0.24
	0.002	0.000	0.000000	0.34

3 Comparison with MM-PBSA Residue Decomposition and Implications for Drug Design

Table S11. Per-residue free energy decomposition (in kJ/mol) obtained from MM-PBSA analysis of the Inh-HIV^{Pro} complexes. Values correspond to the energetic contribution of each residue to the ligand-protein interaction, including both chains A and B.

	DAR-HIV^{Pro}	AMP-HIV^{Pro}	RIT-HIV^{Pro}	SAQ-HIV^{Pro}	IND-HIV^{Pro}	NEL-HIV^{Pro}
ARG A 8	-1.16	1.74	-4.21	17.1	20.87	17.58
ARG B 8	0.84	-1.11	-0.69	13.0	11.74	17.09
LEU A 23	-0.68	-0.87	-0.90	-0.7	-0.56	-0.48
LEU B 23	-0.56	-0.22	-0.31	-0.2	-0.02	-0.09
ASP A 25	0.27	-11.33	-2.55	-2.1	-3.30	-37.85
ASP B 25	-10.44	-1.72	-2.31	-1.7	-1.97	-34.48
GLY A 27	-1.46	-1.62	-3.07	-3.6	-3.34	-0.72
GLY B 27	-0.16	-0.43	-1.99	-3.5	-2.11	0.66
ALA A 28	-2.07	-2.67	-2.15	-2.1	-1.71	-0.70
ALA B 28	-3.85	-1.22	-1.85	0.2	-0.46	0.09
ASP A 29	-2.36	-3.33	-1.86	-18.8	-22.73	-17.78
ASP B 29	-0.49	-0.38	-0.18	-13.4	-14.21	-17.55
ASP A 30	-6.86	-6.03	-0.53	-17.1	-12.61	-15.07
ASP B 30	-2.26	0.29	-0.46	-12.6	-1.08	-11.94
VAL A 32	-0.66	-0.70	-0.58	-0.7	-0.94	-0.80
VAL B 32	-0.56	-0.66	-0.55	-0.5	-0.57	-0.69
ILE A 47	-1.49	-1.12	-0.94	-0.6	-0.71	-0.71
ILE B 47	-0.53	-0.33	-0.36	0.1	0.35	-0.06
GLY A 48	-1.48	-1.49	-3.55	-2.7	-3.57	-2.98
GLY B 48	-1.13	-0.79	-1.03	-2.0	-1.64	-1.46
GLY A 49	-1.54	-3.03	-4.09	-1.5	-2.52	-0.91
GLY B 49	-1.74	-2.12	-1.68	-0.3	-1.20	-0.47
ILE A 50	-2.24	-3.21	-2.87	-1.9	-2.88	-2.37

Continue on the next page

Table S11. (Continued.)

ILE B 50	-2.31	-2.83	-2.35	-1.7	-2.49	-1.47
LEU A 76	-0.19	-0.25	0.00	0.0	0.00	-0.34
LEU B 76	0.00	-0.08	-0.06	-0.2	-0.27	0.00
THR A 80	0.00	0.00	0.00	0.0	0.00	0.00
THR B 80	0.00	0.00	0.00	0.0	0.00	0.00
PRO A 81	-0.39	-0.74	-1.45	-0.8	-0.35	-0.85
PRO B 81	-0.79	-0.62	-0.64	0.3	0.03	0.30
VAL A 82	-0.50	-0.81	-1.55	-1.4	-1.18	-1.25
VAL B 82	-0.67	-0.51	-0.87	-1.0	-1.10	-0.78
ILE A 84	-1.69	-1.55	-1.52	-1.3	-1.09	-0.74
ILE B 84	-1.79	-1.20	-1.10	-0.6	-0.75	-0.30

4 Reduced Systems: Hydrogen-Refined Structures

4.1 AMP-HIV^{Pro}

```
HEADER  data-set: HIV_AMP_full_wH2O_OPT
REMARK  MOPAC, Version: 23.1.2
REMARK  99
REMARK  99 MOE v2014.09 (Chemical Computing Group Inc.)
ATOM      1  C   ACE A   7      64.926  50.280  34.227  1.0   4.40      PROT C
ATOM      2  O   ACE A   7      64.926  51.010  33.213  1.0  -6.07      PROT O
ATOM      3  HC  ACE A   7      65.763  50.387  34.951  1.0   1.43      PROT H
ATOM      4  N   ARG A   8      64.010  49.420  34.500  1.0  -4.92      PROT N
ATOM      5  CA  ARG A   8      62.890  49.220  33.650  1.0  -0.28      PROT C
ATOM      6  CB  ARG A   8      63.280  49.000  32.170  1.0  -2.78      PROT C
ATOM      7  CG  ARG A   8      63.470  50.210  31.240  1.0  -2.91      PROT C
ATOM      8  CD  ARG A   8      63.580  49.610  29.850  1.0  -0.60      PROT C
ATOM      9  NE  ARG A   8      64.090  50.750  29.040  1.0  -5.18      PROT N
ATOM     10  CZ  ARG A   8      63.980  50.850  27.760  1.0   6.30      PROT C
ATOM     11  NH1 ARG A   8      63.460  49.890  27.000  1.0  -6.56      PROT N
ATOM     12  NH2 ARG A   8      64.390  51.910  27.180  1.0  -6.43      PROT N
ATOM     13  C   ARG A   8      61.900  48.150  34.140  1.0   5.52      PROT C
ATOM     14  O   ARG A   8      62.280  47.090  34.730  1.0  -6.72      PROT O
```

ATOM	15	H	ARG	A	8	64.081	48.770	35.294	1.0	3.33	PROT	H
ATOM	16	HA	ARG	A	8	62.313	50.219	33.650	1.0	1.95	PROT	H
ATOM	17	HB2	ARG	A	8	62.510	48.335	31.717	1.0	1.40	PROT	H
ATOM	18	HB3	ARG	A	8	64.218	48.399	32.153	1.0	1.59	PROT	H
ATOM	19	HG2	ARG	A	8	64.368	50.822	31.489	1.0	2.21	PROT	H
ATOM	20	HG3	ARG	A	8	62.627	50.930	31.315	1.0	1.85	PROT	H
ATOM	21	HD2	ARG	A	8	62.586	49.269	29.478	1.0	1.53	PROT	H
ATOM	22	HD3	ARG	A	8	64.300	48.766	29.788	1.0	1.42	PROT	H
ATOM	23	HE	ARG	A	8	64.482	51.565	29.615	1.0	4.10	PROT	H
ATOM	24	HH11	ARG	A	8	62.928	49.129	27.396	1.0	3.24	PROT	H
ATOM	25	HH12	ARG	A	8	63.325	50.012	26.007	1.0	3.29	PROT	H
ATOM	26	HH21	ARG	A	8	64.861	52.720	27.727	1.0	4.12	PROT	H
ATOM	27	HH22	ARG	A	8	64.457	52.025	26.176	1.0	3.40	PROT	H
ATOM	28	N	NME	A	9	60.629	48.399	33.910	1.0	-5.86	PROT	N
ATOM	29	H1	NME	A	9	60.295	49.256	33.490	1.0	3.28	PROT	H
ATOM	30	H2	NME	A	9	59.900	47.763	34.204	1.0	3.26	PROT	H
ATOM	31	C	ACE	A	10	58.179	44.387	28.654	1.0	4.86	PROT	C
ATOM	32	O	ACE	A	10	59.334	44.625	29.064	1.0	-6.70	PROT	O
ATOM	33	HC	ACE	A	10	58.046	43.573	27.907	1.0	1.48	PROT	H
ATOM	34	N	LEU	A	23	57.130	45.030	29.030	1.0	-4.94	PROT	N
ATOM	35	CA	LEU	A	23	57.200	46.140	30.010	1.0	-0.10	PROT	C
ATOM	36	CB	LEU	A	23	58.040	47.280	29.520	1.0	-3.16	PROT	C
ATOM	37	CG	LEU	A	23	59.580	47.020	29.190	1.0	-0.73	PROT	C
ATOM	38	CD1	LEU	A	23	60.230	48.000	28.240	1.0	-4.66	PROT	C
ATOM	39	CD2	LEU	A	23	60.400	46.900	30.490	1.0	-4.37	PROT	C
ATOM	40	C	LEU	A	23	55.810	46.730	30.280	1.0	5.73	PROT	C
ATOM	41	O	LEU	A	23	55.110	47.120	29.330	1.0	-6.39	PROT	O
ATOM	42	H	LEU	A	23	56.258	44.998	28.490	1.0	3.36	PROT	H
ATOM	43	HA	LEU	A	23	57.648	45.704	30.952	1.0	1.73	PROT	H
ATOM	44	HB2	LEU	A	23	58.013	48.118	30.246	1.0	1.53	PROT	H
ATOM	45	HB3	LEU	A	23	57.599	47.683	28.578	1.0	1.68	PROT	H
ATOM	46	HG	LEU	A	23	59.683	46.103	28.569	1.0	1.92	PROT	H
ATOM	47	HD11	LEU	A	23	59.653	48.096	27.310	1.0	1.43	PROT	H
ATOM	48	HD12	LEU	A	23	60.320	49.012	28.669	1.0	1.70	PROT	H
ATOM	49	HD13	LEU	A	23	61.235	47.662	27.967	1.0	1.30	PROT	H
ATOM	50	HD21	LEU	A	23	60.358	47.824	31.072	1.0	1.36	PROT	H
ATOM	51	HD22	LEU	A	23	60.019	46.079	31.105	1.0	1.37	PROT	H
ATOM	52	HD23	LEU	A	23	61.446	46.670	30.269	1.0	1.38	PROT	H

ATOM	53	N	LEU	A	24	55.490	47.010	31.560	1.0	-5.39	PROT	N
ATOM	54	CA	LEU	A	24	54.280	47.750	31.960	1.0	0.20	PROT	C
ATOM	55	CB	LEU	A	24	54.030	47.520	33.460	1.0	-3.23	PROT	C
ATOM	56	CG	LEU	A	24	53.870	46.070	33.920	1.0	-0.53	PROT	C
ATOM	57	CD1	LEU	A	24	53.380	46.250	35.410	1.0	-4.63	PROT	C
ATOM	58	CD2	LEU	A	24	52.900	45.200	33.020	1.0	-4.53	PROT	C
ATOM	59	C	LEU	A	24	54.500	49.220	31.600	1.0	5.75	PROT	C
ATOM	60	O	LEU	A	24	55.460	49.830	32.070	1.0	-6.41	PROT	O
ATOM	61	H	LEU	A	24	56.099	46.733	32.327	1.0	3.32	PROT	H
ATOM	62	HA	LEU	A	24	53.404	47.334	31.377	1.0	1.80	PROT	H
ATOM	63	HB2	LEU	A	24	53.115	48.097	33.730	1.0	1.75	PROT	H
ATOM	64	HB3	LEU	A	24	54.849	48.001	34.042	1.0	1.69	PROT	H
ATOM	65	HG	LEU	A	24	54.849	45.548	33.934	1.0	1.28	PROT	H
ATOM	66	HD11	LEU	A	24	53.288	45.278	35.901	1.0	1.40	PROT	H
ATOM	67	HD12	LEU	A	24	54.090	46.849	35.983	1.0	1.48	PROT	H
ATOM	68	HD13	LEU	A	24	52.400	46.731	35.444	1.0	1.53	PROT	H
ATOM	69	HD21	LEU	A	24	53.351	45.021	32.042	1.0	1.48	PROT	H
ATOM	70	HD22	LEU	A	24	52.715	44.237	33.498	1.0	1.41	PROT	H
ATOM	71	HD23	LEU	A	24	51.942	45.706	32.884	1.0	1.54	PROT	H
ATOM	72	N	ASH	A	25	53.780	49.760	30.630	1.0	-4.88	PROT	N
ATOM	73	CA	ASH	A	25	53.980	51.040	30.020	1.0	-0.12	PROT	C
ATOM	74	CB	ASH	A	25	54.510	50.840	28.670	1.0	-3.70	PROT	C
ATOM	75	CG	ASH	A	25	54.980	52.180	28.040	1.0	6.75	PROT	C
ATOM	76	OD1	ASH	A	25	54.140	52.990	27.540	1.0	-5.66	PROT	O
ATOM	77	OD2	ASH	A	25	56.340	52.450	28.170	1.0	-5.42	PROT	O
ATOM	78	C	ASH	A	25	52.750	51.970	30.150	1.0	5.88	PROT	C
ATOM	79	O	ASH	A	25	51.660	51.470	30.290	1.0	-5.94	PROT	O
ATOM	80	H	ASH	A	25	52.942	49.269	30.276	1.0	3.40	PROT	H
ATOM	81	HA	ASH	A	25	54.801	51.577	30.638	1.0	1.99	PROT	H
ATOM	82	HB2	ASH	A	25	53.747	50.404	27.975	1.0	2.04	PROT	H
ATOM	83	HB3	ASH	A	25	55.360	50.115	28.660	1.0	1.96	PROT	H
ATOM	84	HD2	ASH	A	25	56.502	53.438	27.949	1.0	4.10	PROT	H
ATOM	85	N	THR	A	26	52.920	53.300	30.320	1.0	-5.46	PROT	N
ATOM	86	CA	THR	A	26	51.850	54.250	30.560	1.0	-0.62	PROT	C
ATOM	87	CB	THR	A	26	51.910	54.740	31.970	1.0	1.85	PROT	C
ATOM	88	CG2	THR	A	26	51.890	53.650	33.030	1.0	-4.85	PROT	C
ATOM	89	OG1	THR	A	26	53.040	55.510	32.220	1.0	-6.37	PROT	O
ATOM	90	C	THR	A	26	51.780	55.360	29.500	1.0	5.70	PROT	C

ATOM	91	O	THR A	26	50.820	56.140	29.410	1.0	-5.93	PROT	O
ATOM	92	H	THR A	26	53.865	53.705	30.362	1.0	3.40	PROT	H
ATOM	93	HA	THR A	26	50.853	53.690	30.433	1.0	2.16	PROT	H
ATOM	94	HB	THR A	26	51.011	55.410	32.134	1.0	1.27	PROT	H
ATOM	95	HG21	THR A	26	51.185	52.847	32.786	1.0	1.63	PROT	H
ATOM	96	HG22	THR A	26	52.890	53.194	33.134	1.0	1.77	PROT	H
ATOM	97	HG23	THR A	26	51.629	54.057	34.011	1.0	1.44	PROT	H
ATOM	98	HG1	THR A	26	52.936	56.469	32.012	1.0	3.69	PROT	H
ATOM	99	N	GLY A	27	52.850	55.450	28.720	1.0	-5.36	PROT	N
ATOM	100	CA	GLY A	27	52.930	56.220	27.460	1.0	-1.55	PROT	C
ATOM	101	C	GLY A	27	52.160	55.550	26.310	1.0	5.84	PROT	C
ATOM	102	O	GLY A	27	51.490	56.250	25.610	1.0	-5.95	PROT	O
ATOM	103	H	GLY A	27	53.623	54.764	28.773	1.0	3.54	PROT	H
ATOM	104	HA2	GLY A	27	54.002	56.352	27.188	1.0	1.69	PROT	H
ATOM	105	HA3	GLY A	27	52.484	57.235	27.616	1.0	1.87	PROT	H
ATOM	106	N	ALA A	28	52.220	54.240	26.140	1.0	-5.74	PROT	N
ATOM	107	CA	ALA A	28	51.420	53.390	25.180	1.0	0.61	PROT	C
ATOM	108	CB	ALA A	28	52.130	52.100	24.840	1.0	-4.73	PROT	C
ATOM	109	C	ALA A	28	50.020	53.070	25.800	1.0	5.47	PROT	C
ATOM	110	O	ALA A	28	49.950	52.380	26.830	1.0	-6.26	PROT	O
ATOM	111	H	ALA A	28	52.731	53.670	26.808	1.0	3.37	PROT	H
ATOM	112	HA	ALA A	28	51.294	54.011	24.241	1.0	1.91	PROT	H
ATOM	113	HB1	ALA A	28	53.023	52.277	24.227	1.0	1.57	PROT	H
ATOM	114	HB2	ALA A	28	52.442	51.551	25.740	1.0	1.61	PROT	H
ATOM	115	HB3	ALA A	28	51.474	51.417	24.271	1.0	1.88	PROT	H
ATOM	116	N	ASP A	29	48.970	53.500	25.140	1.0	-5.38	PROT	N
ATOM	117	CA	ASP A	29	47.610	53.290	25.410	1.0	-0.01	PROT	C
ATOM	118	CB	ASP A	29	46.710	54.170	24.480	1.0	-3.99	PROT	C
ATOM	119	CG	ASP A	29	47.040	55.660	24.360	1.0	7.53	PROT	C
ATOM	120	OD1	ASP A	29	46.670	56.430	25.230	1.0	-7.84	PROT	O
ATOM	121	OD2	ASP A	29	47.430	56.090	23.220	1.0	-8.47	PROT	O
ATOM	122	C	ASP A	29	47.260	51.840	25.300	1.0	6.12	PROT	C
ATOM	123	O	ASP A	29	46.400	51.440	26.000	1.0	-6.12	PROT	O
ATOM	124	H	ASP A	29	49.151	54.117	24.312	1.0	3.52	PROT	H
ATOM	125	HA	ASP A	29	47.376	53.614	26.482	1.0	1.94	PROT	H
ATOM	126	HB2	ASP A	29	45.663	54.091	24.840	1.0	1.86	PROT	H
ATOM	127	HB3	ASP A	29	46.711	53.741	23.453	1.0	1.88	PROT	H
ATOM	128	N	ASP A	30	47.870	51.210	24.310	1.0	-5.14	PROT	N

ATOM	129	CA	ASP	A	30	47.630	49.880	23.890	1.0	0.23	PROT	C
ATOM	130	CB	ASP	A	30	46.780	49.710	22.580	1.0	-4.18	PROT	C
ATOM	131	CG	ASP	A	30	45.650	50.760	22.250	1.0	7.67	PROT	C
ATOM	132	OD1	ASP	A	30	45.770	51.400	21.210	1.0	-7.84	PROT	O
ATOM	133	OD2	ASP	A	30	44.540	50.840	22.890	1.0	-8.49	PROT	O
ATOM	134	C	ASP	A	30	48.890	48.990	23.790	1.0	5.28	PROT	C
ATOM	135	O	ASP	A	30	50.010	49.500	23.460	1.0	-6.21	PROT	O
ATOM	136	H	ASP	A	30	48.619	51.680	23.783	1.0	3.29	PROT	H
ATOM	137	HA	ASP	A	30	46.969	49.406	24.701	1.0	1.79	PROT	H
ATOM	138	HB2	ASP	A	30	46.289	48.726	22.597	1.0	1.53	PROT	H
ATOM	139	HB3	ASP	A	30	47.458	49.720	21.704	1.0	1.74	PROT	H
ATOM	140	N	THR	A	31	48.760	47.730	24.100	1.0	-4.83	PROT	N
ATOM	141	CA	THR	A	31	49.760	46.710	24.160	1.0	-0.46	PROT	C
ATOM	142	CB	THR	A	31	49.190	45.480	24.880	1.0	1.46	PROT	C
ATOM	143	CG2	THR	A	31	49.760	43.980	24.700	1.0	-4.82	PROT	C
ATOM	144	OG1	THR	A	31	49.010	45.790	26.230	1.0	-5.86	PROT	O
ATOM	145	C	THR	A	31	50.350	46.350	22.810	1.0	5.73	PROT	C
ATOM	146	O	THR	A	31	49.510	46.080	21.920	1.0	-6.42	PROT	O
ATOM	147	H	THR	A	31	47.805	47.358	24.264	1.0	3.27	PROT	H
ATOM	148	HA	THR	A	31	50.614	47.112	24.812	1.0	1.89	PROT	H
ATOM	149	HB	THR	A	31	48.108	45.363	24.557	1.0	1.58	PROT	H
ATOM	150	HG21	THR	A	31	49.168	43.309	25.318	1.0	1.50	PROT	H
ATOM	151	HG22	THR	A	31	49.689	43.688	23.652	1.0	1.64	PROT	H
ATOM	152	HG23	THR	A	31	50.804	43.928	25.010	1.0	1.54	PROT	H
ATOM	153	HG1	THR	A	31	49.872	45.922	26.681	1.0	3.37	PROT	H
ATOM	154	N	VAL	A	32	51.630	46.350	22.600	1.0	-5.25	PROT	N
ATOM	155	CA	VAL	A	32	52.470	46.310	21.370	1.0	-0.14	PROT	C
ATOM	156	CB	VAL	A	32	53.160	47.670	21.020	1.0	-0.76	PROT	C
ATOM	157	CG1	VAL	A	32	53.660	47.760	19.590	1.0	-4.58	PROT	C
ATOM	158	CG2	VAL	A	32	52.180	48.760	21.250	1.0	-4.64	PROT	C
ATOM	159	C	VAL	A	32	53.550	45.220	21.480	1.0	5.98	PROT	C
ATOM	160	O	VAL	A	32	54.290	45.140	22.430	1.0	-6.61	PROT	O
ATOM	161	H	VAL	A	32	52.259	46.461	23.424	1.0	3.38	PROT	H
ATOM	162	HA	VAL	A	32	51.766	46.065	20.513	1.0	1.94	PROT	H
ATOM	163	HB	VAL	A	32	54.042	47.781	21.710	1.0	1.47	PROT	H
ATOM	164	HG11	VAL	A	32	54.226	48.685	19.426	1.0	1.55	PROT	H
ATOM	165	HG12	VAL	A	32	54.328	46.928	19.329	1.0	1.47	PROT	H
ATOM	166	HG13	VAL	A	32	52.839	47.731	18.862	1.0	1.53	PROT	H

```
ATOM    167 HG21 VAL A  32      51.902  48.884  22.313  1.0   1.81      PROT H
ATOM    168 HG22 VAL A  32      52.557  49.740  20.927  1.0   1.46      PROT H
ATOM    169 HG23 VAL A  32      51.226  48.594  20.724  1.0   1.63      PROT H
ATOM    170  N   NME A  33      53.617  44.378  20.472  1.0  -6.32      PROT N
ATOM    171  H1  NME A  33      53.011  44.401  19.661  1.0   3.44      PROT H
ATOM    172  H2  NME A  33      54.325  43.655  20.457  1.0   3.14      PROT H
ATOM    173  C   ACE A  34      50.320  52.583  14.035  1.0   4.50      PROT C
ATOM    174  O   ACE A  34      49.378  52.500  14.851  1.0  -6.53      PROT O
ATOM    175  HC  ACE A  34      50.202  52.079  13.051  1.0   1.39      PROT H
ATOM    176  N   ILE A  47      51.420  53.210  14.260  1.0  -5.04      PROT N
ATOM    177  CA  ILE A  47      51.670  53.890  15.510  1.0  -0.58      PROT C
ATOM    178  CB  ILE A  47      51.550  52.940  16.760  1.0  -0.70      PROT C
ATOM    179  CG2 ILE A  47      50.090  52.630  17.090  1.0  -4.25      PROT C
ATOM    180  CG1 ILE A  47      52.310  51.620  16.600  1.0  -2.81      PROT C
ATOM    181  CD1 ILE A  47      52.290  50.710  17.870  1.0  -4.27      PROT C
ATOM    182  C   ILE A  47      53.030  54.450  15.310  1.0   5.71      PROT C
ATOM    183  O   ILE A  47      53.860  53.960  14.530  1.0  -6.23      PROT O
ATOM    184  H   ILE A  47      52.211  53.169  13.593  1.0   3.50      PROT H
ATOM    185  HA  ILE A  47      50.895  54.707  15.652  1.0   1.97      PROT H
ATOM    186  HB  ILE A  47      52.000  53.508  17.615  1.0   1.39      PROT H
ATOM    187 HG12 ILE A  47      53.362  51.821  16.325  1.0   1.32      PROT H
ATOM    188 HG13 ILE A  47      51.890  51.043  15.755  1.0   1.39      PROT H
ATOM    189 HG21 ILE A  47      49.957  52.540  18.180  1.0   1.44      PROT H
ATOM    190 HG22 ILE A  47      49.398  53.415  16.761  1.0   1.61      PROT H
ATOM    191 HG23 ILE A  47      49.749  51.676  16.667  1.0   1.69      PROT H
ATOM    192 HD11 ILE A  47      52.762  51.211  18.718  1.0   1.43      PROT H
ATOM    193 HD12 ILE A  47      51.265  50.440  18.144  1.0   1.60      PROT H
ATOM    194 HD13 ILE A  47      52.840  49.785  17.673  1.0   1.42      PROT H
ATOM    195  N   GLY A  48      53.310  55.580  16.050  1.0  -5.38      PROT N
ATOM    196  CA  GLY A  48      54.580  56.250  15.930  1.0  -1.63      PROT C
ATOM    197  C   GLY A  48      55.000  57.160  17.150  1.0   5.89      PROT C
ATOM    198  O   GLY A  48      54.110  57.760  17.790  1.0  -6.27      PROT O
ATOM    199  H   GLY A  48      52.652  55.981  16.716  1.0   3.50      PROT H
ATOM    200  HA2 GLY A  48      54.575  56.923  15.031  1.0   1.78      PROT H
ATOM    201  HA3 GLY A  48      55.388  55.499  15.728  1.0   1.83      PROT H
ATOM    202  N   GLY A  49      56.290  57.130  17.510  1.0  -5.16      PROT N
ATOM    203  CA  GLY A  49      56.840  57.490  18.820  1.0  -1.59      PROT C
ATOM    204  C   GLY A  49      58.100  58.290  18.680  1.0   5.71      PROT C
```

ATOM	205	O	GLY	A	49	58.190	59.150	17.790	1.0	-6.49	PROT	O
ATOM	206	H	GLY	A	49	56.984	56.668	16.916	1.0	3.26	PROT	H
ATOM	207	HA2	GLY	A	49	56.984	56.550	19.418	1.0	1.86	PROT	H
ATOM	208	HA3	GLY	A	49	56.088	58.096	19.402	1.0	2.09	PROT	H
ATOM	209	N	ILE	A	50	59.010	58.140	19.640	1.0	-5.34	PROT	N
ATOM	210	CA	ILE	A	50	60.330	58.840	19.570	1.0	-0.35	PROT	C
ATOM	211	CB	ILE	A	50	60.920	59.270	20.950	1.0	-0.73	PROT	C
ATOM	212	CG2	ILE	A	50	62.220	60.030	20.700	1.0	-4.69	PROT	C
ATOM	213	CG1	ILE	A	50	59.840	60.220	21.600	1.0	-2.52	PROT	C
ATOM	214	CD1	ILE	A	50	60.430	60.890	22.890	1.0	-4.40	PROT	C
ATOM	215	C	ILE	A	50	61.200	58.020	18.590	1.0	5.41	PROT	C
ATOM	216	O	ILE	A	50	61.670	56.950	19.050	1.0	-5.89	PROT	O
ATOM	217	H	ILE	A	50	58.913	57.486	20.431	1.0	3.68	PROT	H
ATOM	218	HA	ILE	A	50	60.112	59.837	19.058	1.0	1.63	PROT	H
ATOM	219	HB	ILE	A	50	61.098	58.391	21.600	1.0	1.60	PROT	H
ATOM	220	HG12	ILE	A	50	58.946	59.633	21.867	1.0	1.51	PROT	H
ATOM	221	HG13	ILE	A	50	59.533	61.003	20.893	1.0	1.36	PROT	H
ATOM	222	HG21	ILE	A	50	62.920	59.441	20.094	1.0	1.48	PROT	H
ATOM	223	HG22	ILE	A	50	62.051	60.984	20.189	1.0	1.48	PROT	H
ATOM	224	HG23	ILE	A	50	62.737	60.249	21.644	1.0	1.62	PROT	H
ATOM	225	HD11	ILE	A	50	59.657	61.484	23.387	1.0	1.51	PROT	H
ATOM	226	HD12	ILE	A	50	60.793	60.130	23.587	1.0	1.48	PROT	H
ATOM	227	HD13	ILE	A	50	61.249	61.565	22.639	1.0	1.36	PROT	H
ATOM	228	N	NME	A	51	61.348	58.535	17.389	1.0	-6.12	PROT	N
ATOM	229	H1	NME	A	51	60.783	59.308	17.062	1.0	3.31	PROT	H
ATOM	230	H2	NME	A	51	61.841	58.036	16.662	1.0	3.16	PROT	H
ATOM	231	C	ACE	A	52	47.553	45.313	19.873	1.0	4.40	PROT	C
ATOM	232	O	ACE	A	52	46.883	46.212	19.323	1.0	-5.99	PROT	O
ATOM	233	HC	ACE	A	52	47.197	44.890	20.840	1.0	1.42	PROT	H
ATOM	234	N	LEU	A	76	48.660	44.850	19.410	1.0	-5.10	PROT	N
ATOM	235	CA	LEU	A	76	49.250	45.350	18.160	1.0	-0.14	PROT	C
ATOM	236	CB	LEU	A	76	49.530	46.900	18.220	1.0	-3.11	PROT	C
ATOM	237	CG	LEU	A	76	48.300	47.810	18.750	1.0	0.13	PROT	C
ATOM	238	CD1	LEU	A	76	48.780	49.270	18.580	1.0	-4.81	PROT	C
ATOM	239	CD2	LEU	A	76	47.010	47.540	17.880	1.0	-3.82	PROT	C
ATOM	240	C	LEU	A	76	50.600	44.610	17.830	1.0	5.62	PROT	C
ATOM	241	O	LEU	A	76	51.650	44.900	18.430	1.0	-6.47	PROT	O
ATOM	242	H	LEU	A	76	49.337	44.410	20.047	1.0	3.31	PROT	H

ATOM	243	HA	LEU	A	76	48.512	45.166	17.335	1.0	1.58	PROT	H
ATOM	244	HB2	LEU	A	76	49.808	47.242	17.216	1.0	1.36	PROT	H
ATOM	245	HB3	LEU	A	76	50.383	47.078	18.895	1.0	1.57	PROT	H
ATOM	246	HG	LEU	A	76	48.158	47.649	19.835	1.0	1.61	PROT	H
ATOM	247	HD11	LEU	A	76	49.037	49.500	17.544	1.0	1.31	PROT	H
ATOM	248	HD12	LEU	A	76	47.989	49.965	18.892	1.0	1.69	PROT	H
ATOM	249	HD13	LEU	A	76	49.655	49.463	19.209	1.0	1.41	PROT	H
ATOM	250	HD21	LEU	A	76	46.992	48.273	17.055	1.0	1.02	PROT	H
ATOM	251	HD22	LEU	A	76	46.996	46.574	17.376	1.0	1.21	PROT	H
ATOM	252	HD23	LEU	A	76	46.084	47.731	18.439	1.0	1.53	PROT	H
ATOM	253	N	NME	A	77	50.532	43.687	16.896	1.0	-6.09	PROT	N
ATOM	254	H1	NME	A	77	49.674	43.401	16.449	1.0	3.26	PROT	H
ATOM	255	H2	NME	A	77	51.344	43.156	16.611	1.0	3.18	PROT	H
ATOM	256	C	ACE	A	78	56.840	46.417	16.065	1.0	4.65	PROT	C
ATOM	257	O	ACE	A	78	55.894	47.147	16.426	1.0	-6.60	PROT	O
ATOM	258	HC	ACE	A	78	56.714	45.818	15.137	1.0	1.45	PROT	H
ATOM	259	N	THR	A	80	57.960	46.320	16.690	1.0	-4.95	PROT	N
ATOM	260	CA	THR	A	80	58.240	47.100	17.950	1.0	-0.71	PROT	C
ATOM	261	CB	THR	A	80	57.220	46.920	19.080	1.0	1.72	PROT	C
ATOM	262	CG2	THR	A	80	56.760	45.490	19.430	1.0	-4.93	PROT	C
ATOM	263	OG1	THR	A	80	57.650	47.630	20.220	1.0	-6.27	PROT	O
ATOM	264	C	THR	A	80	59.620	46.820	18.500	1.0	5.97	PROT	C
ATOM	265	O	THR	A	80	60.150	45.750	18.310	1.0	-6.22	PROT	O
ATOM	266	H	THR	A	80	58.694	45.645	16.461	1.0	3.43	PROT	H
ATOM	267	HA	THR	A	80	58.183	48.189	17.633	1.0	1.87	PROT	H
ATOM	268	HB	THR	A	80	56.298	47.507	18.778	1.0	1.68	PROT	H
ATOM	269	HG21	THR	A	80	57.609	44.810	19.555	1.0	1.57	PROT	H
ATOM	270	HG22	THR	A	80	56.218	45.497	20.389	1.0	1.84	PROT	H
ATOM	271	HG23	THR	A	80	56.102	45.084	18.658	1.0	1.56	PROT	H
ATOM	272	HG1	THR	A	80	57.829	47.034	20.983	1.0	3.79	PROT	H
ATOM	273	N	PRO	A	81	60.340	47.830	19.060	1.0	-4.94	PROT	N
ATOM	274	CD	PRO	A	81	60.060	49.250	19.190	1.0	-0.60	PROT	C
ATOM	275	CG	PRO	A	81	61.060	49.770	20.240	1.0	-2.77	PROT	C
ATOM	276	CB	PRO	A	81	62.280	48.980	19.830	1.0	-2.88	PROT	C
ATOM	277	CA	PRO	A	81	61.750	47.580	19.410	1.0	0.10	PROT	C
ATOM	278	C	PRO	A	81	61.830	46.550	20.600	1.0	5.92	PROT	C
ATOM	279	O	PRO	A	81	62.790	45.790	20.590	1.0	-6.23	PROT	O
ATOM	280	HA	PRO	A	81	62.317	47.138	18.556	1.0	1.81	PROT	H

ATOM	281	HB2	PRO	A	81	63.032	48.895	20.636	1.0	1.62	PROT	H
ATOM	282	HB3	PRO	A	81	62.812	49.451	18.981	1.0	1.66	PROT	H
ATOM	283	HG2	PRO	A	81	60.731	49.552	21.271	1.0	1.53	PROT	H
ATOM	284	HG3	PRO	A	81	61.200	50.862	20.185	1.0	1.62	PROT	H
ATOM	285	HD2	PRO	A	81	59.002	49.407	19.518	1.0	1.74	PROT	H
ATOM	286	HD3	PRO	A	81	60.194	49.768	18.213	1.0	1.44	PROT	H
ATOM	287	N	VAL	A	82	60.750	46.560	21.470	1.0	-5.69	PROT	N
ATOM	288	CA	VAL	A	82	60.510	45.770	22.680	1.0	-0.06	PROT	C
ATOM	289	CB	VAL	A	82	60.930	46.590	23.870	1.0	-0.90	PROT	C
ATOM	290	CG1	VAL	A	82	62.410	46.910	23.830	1.0	-4.47	PROT	C
ATOM	291	CG2	VAL	A	82	60.180	47.910	24.140	1.0	-4.42	PROT	C
ATOM	292	C	VAL	A	82	59.090	45.280	22.790	1.0	5.57	PROT	C
ATOM	293	O	VAL	A	82	58.230	45.970	22.150	1.0	-6.45	PROT	O
ATOM	294	H	VAL	A	82	59.964	47.178	21.238	1.0	3.43	PROT	H
ATOM	295	HA	VAL	A	82	61.198	44.860	22.616	1.0	1.84	PROT	H
ATOM	296	HB	VAL	A	82	60.749	45.946	24.786	1.0	1.49	PROT	H
ATOM	297	HG11	VAL	A	82	63.027	46.016	23.665	1.0	1.48	PROT	H
ATOM	298	HG12	VAL	A	82	62.661	47.611	23.022	1.0	1.52	PROT	H
ATOM	299	HG13	VAL	A	82	62.751	47.364	24.768	1.0	1.47	PROT	H
ATOM	300	HG21	VAL	A	82	60.443	48.684	23.411	1.0	1.48	PROT	H
ATOM	301	HG22	VAL	A	82	59.092	47.766	24.082	1.0	1.59	PROT	H
ATOM	302	HG23	VAL	A	82	60.412	48.290	25.139	1.0	1.48	PROT	H
ATOM	303	N	ASN	A	83	58.770	44.310	23.590	1.0	-4.98	PROT	N
ATOM	304	CA	ASN	A	83	57.340	44.100	24.100	1.0	0.24	PROT	C
ATOM	305	CB	ASN	A	83	57.220	42.720	24.610	1.0	-3.82	PROT	C
ATOM	306	CG	ASN	A	83	57.370	41.680	23.400	1.0	6.01	PROT	C
ATOM	307	OD1	ASN	A	83	56.730	41.710	22.330	1.0	-6.17	PROT	O
ATOM	308	ND2	ASN	A	83	58.280	40.750	23.640	1.0	-6.28	PROT	N
ATOM	309	C	ASN	A	83	56.840	45.060	25.200	1.0	5.46	PROT	C
ATOM	310	O	ASN	A	83	57.530	45.380	26.180	1.0	-6.10	PROT	O
ATOM	311	H	ASN	A	83	59.466	43.834	24.168	1.0	3.32	PROT	H
ATOM	312	HA	ASN	A	83	56.695	44.249	23.164	1.0	2.23	PROT	H
ATOM	313	HB2	ASN	A	83	56.216	42.524	25.052	1.0	1.97	PROT	H
ATOM	314	HB3	ASN	A	83	57.942	42.488	25.416	1.0	1.79	PROT	H
ATOM	315	HD21	ASN	A	83	58.819	40.691	24.489	1.0	3.25	PROT	H
ATOM	316	HD22	ASN	A	83	58.481	40.022	22.968	1.0	3.17	PROT	H
ATOM	317	N	ILE	A	84	55.650	45.570	25.000	1.0	-5.40	PROT	N
ATOM	318	CA	ILE	A	84	55.010	46.740	25.610	1.0	-0.08	PROT	C

ATOM	319	CB	ILE	A	84	54.990	47.920	24.660	1.0	-1.12	PROT	C
ATOM	320	CG2	ILE	A	84	54.260	49.070	25.330	1.0	-4.48	PROT	C
ATOM	321	CG1	ILE	A	84	56.340	48.300	24.150	1.0	-2.84	PROT	C
ATOM	322	CD1	ILE	A	84	56.370	49.500	23.060	1.0	-4.13	PROT	C
ATOM	323	C	ILE	A	84	53.550	46.360	25.990	1.0	5.84	PROT	C
ATOM	324	O	ILE	A	84	52.710	46.010	25.190	1.0	-6.41	PROT	O
ATOM	325	H	ILE	A	84	55.107	45.284	24.150	1.0	3.58	PROT	H
ATOM	326	HA	ILE	A	84	55.591	47.011	26.543	1.0	1.77	PROT	H
ATOM	327	HB	ILE	A	84	54.372	47.611	23.755	1.0	1.68	PROT	H
ATOM	328	HG12	ILE	A	84	56.827	47.437	23.650	1.0	1.51	PROT	H
ATOM	329	HG13	ILE	A	84	57.013	48.599	24.973	1.0	1.36	PROT	H
ATOM	330	HG21	ILE	A	84	53.215	48.827	25.563	1.0	1.56	PROT	H
ATOM	331	HG22	ILE	A	84	54.757	49.386	26.254	1.0	1.38	PROT	H
ATOM	332	HG23	ILE	A	84	54.229	49.951	24.668	1.0	1.72	PROT	H
ATOM	333	HD11	ILE	A	84	57.325	49.477	22.537	1.0	1.37	PROT	H
ATOM	334	HD12	ILE	A	84	55.577	49.349	22.327	1.0	1.51	PROT	H
ATOM	335	HD13	ILE	A	84	56.236	50.459	23.557	1.0	1.44	PROT	H
ATOM	336	N	NME	A	85	53.254	46.438	27.269	1.0	-6.24	PROT	N
ATOM	337	H1	NME	A	85	53.904	46.781	27.963	1.0	3.40	PROT	H
ATOM	338	H2	NME	A	85	52.327	46.222	27.608	1.0	3.21	PROT	H
TER	339		NME	A	85							
ATOM	340	C	ACE	B	7	44.564	58.547	28.862	1.0	4.44	PROT	C
ATOM	341	O	ACE	B	7	45.199	58.047	27.910	1.0	-6.18	PROT	O
ATOM	342	HC	ACE	B	7	43.454	58.479	28.844	1.0	1.47	PROT	H
ATOM	343	N	ARG	B	8	45.110	59.130	29.870	1.0	-4.85	PROT	N
ATOM	344	CA	ARG	B	8	46.520	59.260	29.990	1.0	-0.28	PROT	C
ATOM	345	CB	ARG	B	8	47.110	59.970	28.810	1.0	-2.95	PROT	C
ATOM	346	CG	ARG	B	8	47.310	59.050	27.670	1.0	-3.14	PROT	C
ATOM	347	CD	ARG	B	8	48.040	59.800	26.600	1.0	-1.18	PROT	C
ATOM	348	NE	ARG	B	8	48.350	58.960	25.490	1.0	-4.98	PROT	N
ATOM	349	CZ	ARG	B	8	48.930	59.390	24.420	1.0	6.24	PROT	C
ATOM	350	NH1	ARG	B	8	49.390	60.550	24.230	1.0	-6.08	PROT	N
ATOM	351	NH2	ARG	B	8	48.960	58.540	23.440	1.0	-6.47	PROT	N
ATOM	352	C	ARG	B	8	47.020	59.970	31.230	1.0	5.52	PROT	C
ATOM	353	O	ARG	B	8	46.420	61.030	31.590	1.0	-6.71	PROT	O
ATOM	354	H	ARG	B	8	44.560	59.614	30.591	1.0	3.33	PROT	H
ATOM	355	HA	ARG	B	8	46.937	58.184	30.002	1.0	1.97	PROT	H
ATOM	356	HB2	ARG	B	8	48.078	60.459	29.089	1.0	1.52	PROT	H

ATOM	357	HB3	ARG	B	8	46.465	60.838	28.525	1.0	1.64	PROT	H
ATOM	358	HG2	ARG	B	8	46.388	58.701	27.129	1.0	2.41	PROT	H
ATOM	359	HG3	ARG	B	8	47.829	58.110	27.962	1.0	1.76	PROT	H
ATOM	360	HD2	ARG	B	8	48.996	60.240	27.009	1.0	1.48	PROT	H
ATOM	361	HD3	ARG	B	8	47.424	60.685	26.275	1.0	1.44	PROT	H
ATOM	362	HE	ARG	B	8	47.895	57.990	25.510	1.0	4.08	PROT	H
ATOM	363	HH11	ARG	B	8	49.412	61.279	24.939	1.0	3.32	PROT	H
ATOM	364	HH12	ARG	B	8	49.823	60.845	23.354	1.0	3.45	PROT	H
ATOM	365	HH21	ARG	B	8	48.442	57.600	23.460	1.0	4.09	PROT	H
ATOM	366	HH22	ARG	B	8	49.604	58.604	22.661	1.0	3.32	PROT	H
ATOM	367	N	NME	B	9	48.049	59.413	31.831	1.0	-5.85	PROT	N
ATOM	368	H1	NME	B	9	48.514	58.577	31.493	1.0	3.41	PROT	H
ATOM	369	H2	NME	B	9	48.479	59.827	32.648	1.0	3.26	PROT	H
ATOM	370	C	ACE	B	10	52.652	64.826	29.311	1.0	4.94	PROT	C
ATOM	371	O	ACE	B	10	51.618	64.552	28.666	1.0	-6.92	PROT	O
ATOM	372	HC	ACE	B	10	53.015	65.877	29.290	1.0	1.48	PROT	H
ATOM	373	N	LEU	B	23	53.330	63.970	29.990	1.0	-4.93	PROT	N
ATOM	374	CA	LEU	B	23	52.940	62.560	30.070	1.0	-0.20	PROT	C
ATOM	375	CB	LEU	B	23	52.820	62.020	28.620	1.0	-3.31	PROT	C
ATOM	376	CG	LEU	B	23	52.190	60.620	28.330	1.0	-0.52	PROT	C
ATOM	377	CD1	LEU	B	23	50.830	60.380	28.910	1.0	-4.65	PROT	C
ATOM	378	CD2	LEU	B	23	52.140	60.460	26.860	1.0	-4.57	PROT	C
ATOM	379	C	LEU	B	23	53.940	61.830	30.940	1.0	5.63	PROT	C
ATOM	380	O	LEU	B	23	55.180	61.920	30.700	1.0	-6.53	PROT	O
ATOM	381	H	LEU	B	23	54.295	64.164	30.294	1.0	3.43	PROT	H
ATOM	382	HA	LEU	B	23	51.907	62.511	30.530	1.0	1.73	PROT	H
ATOM	383	HB2	LEU	B	23	53.834	62.041	28.166	1.0	1.63	PROT	H
ATOM	384	HB3	LEU	B	23	52.224	62.752	28.026	1.0	1.98	PROT	H
ATOM	385	HG	LEU	B	23	52.893	59.857	28.773	1.0	1.38	PROT	H
ATOM	386	HD11	LEU	B	23	50.451	59.379	28.640	1.0	1.76	PROT	H
ATOM	387	HD12	LEU	B	23	50.835	60.417	30.006	1.0	1.29	PROT	H
ATOM	388	HD13	LEU	B	23	50.102	61.131	28.576	1.0	1.43	PROT	H
ATOM	389	HD21	LEU	B	23	51.728	59.478	26.563	1.0	1.70	PROT	H
ATOM	390	HD22	LEU	B	23	51.523	61.226	26.371	1.0	1.32	PROT	H
ATOM	391	HD23	LEU	B	23	53.137	60.521	26.392	1.0	1.52	PROT	H
ATOM	392	N	LEU	B	24	53.520	61.150	32.000	1.0	-5.01	PROT	N
ATOM	393	CA	LEU	B	24	54.410	60.160	32.680	1.0	0.00	PROT	C
ATOM	394	CB	LEU	B	24	54.070	60.010	34.220	1.0	-3.09	PROT	C

ATOM	395	CG	LEU	B	24	53.670	61.250	35.040	1.0	-0.49	PROT	C
ATOM	396	CD1	LEU	B	24	53.740	61.090	36.570	1.0	-4.63	PROT	C
ATOM	397	CD2	LEU	B	24	54.570	62.490	34.750	1.0	-4.58	PROT	C
ATOM	398	C	LEU	B	24	54.340	58.770	32.030	1.0	5.67	PROT	C
ATOM	399	O	LEU	B	24	53.260	58.230	31.960	1.0	-6.13	PROT	O
ATOM	400	H	LEU	B	24	52.530	60.984	32.177	1.0	3.25	PROT	H
ATOM	401	HA	LEU	B	24	55.458	60.578	32.615	1.0	1.89	PROT	H
ATOM	402	HB2	LEU	B	24	54.962	59.544	34.689	1.0	1.64	PROT	H
ATOM	403	HB3	LEU	B	24	53.253	59.264	34.319	1.0	1.64	PROT	H
ATOM	404	HG	LEU	B	24	52.621	61.515	34.761	1.0	1.29	PROT	H
ATOM	405	HD11	LEU	B	24	53.145	60.236	36.914	1.0	1.43	PROT	H
ATOM	406	HD12	LEU	B	24	54.769	60.930	36.912	1.0	1.52	PROT	H
ATOM	407	HD13	LEU	B	24	53.354	61.981	37.074	1.0	1.42	PROT	H
ATOM	408	HD21	LEU	B	24	54.518	62.783	33.697	1.0	1.52	PROT	H
ATOM	409	HD22	LEU	B	24	54.258	63.349	35.347	1.0	1.35	PROT	H
ATOM	410	HD23	LEU	B	24	55.617	62.275	34.983	1.0	1.50	PROT	H
ATOM	411	N	ASP	B	25	55.460	58.190	31.580	1.0	-5.47	PROT	N
ATOM	412	CA	ASP	B	25	55.550	56.900	30.890	1.0	0.53	PROT	C
ATOM	413	CB	ASP	B	25	56.000	57.140	29.510	1.0	-4.23	PROT	C
ATOM	414	CG	ASP	B	25	55.990	55.790	28.860	1.0	7.77	PROT	C
ATOM	415	OD1	ASP	B	25	55.470	54.840	29.440	1.0	-8.02	PROT	O
ATOM	416	OD2	ASP	B	25	56.610	55.580	27.770	1.0	-8.30	PROT	O
ATOM	417	C	ASP	B	25	56.580	56.040	31.640	1.0	5.76	PROT	C
ATOM	418	O	ASP	B	25	57.640	56.620	31.960	1.0	-6.59	PROT	O
ATOM	419	H	ASP	B	25	56.376	58.631	31.736	1.0	3.42	PROT	H
ATOM	420	HA	ASP	B	25	54.530	56.394	30.906	1.0	2.12	PROT	H
ATOM	421	HB2	ASP	B	25	57.015	57.598	29.443	1.0	1.95	PROT	H
ATOM	422	HB3	ASP	B	25	55.339	57.845	28.955	1.0	1.94	PROT	H
ATOM	423	N	THR	B	26	56.330	54.790	32.080	1.0	-5.22	PROT	N
ATOM	424	CA	THR	B	26	57.240	53.900	32.870	1.0	-0.38	PROT	C
ATOM	425	CB	THR	B	26	56.350	52.880	33.670	1.0	1.93	PROT	C
ATOM	426	CG2	THR	B	26	55.730	53.620	34.820	1.0	-5.02	PROT	C
ATOM	427	OG1	THR	B	26	55.400	52.380	32.770	1.0	-6.45	PROT	O
ATOM	428	C	THR	B	26	58.280	53.050	32.140	1.0	5.62	PROT	C
ATOM	429	O	THR	B	26	59.270	52.730	32.760	1.0	-6.03	PROT	O
ATOM	430	H	THR	B	26	55.447	54.340	31.826	1.0	3.42	PROT	H
ATOM	431	HA	THR	B	26	57.811	54.561	33.586	1.0	2.00	PROT	H
ATOM	432	HB	THR	B	26	56.985	52.036	34.037	1.0	1.31	PROT	H

ATOM	433	HG21	THR	B	26	55.086	54.446	34.475	1.0	1.79	PROT H
ATOM	434	HG22	THR	B	26	55.082	52.965	35.419	1.0	1.56	PROT H
ATOM	435	HG23	THR	B	26	56.485	54.041	35.496	1.0	1.64	PROT H
ATOM	436	HG1	THR	B	26	55.504	51.419	32.567	1.0	3.77	PROT H
ATOM	437	N	GLY	B	27	58.040	52.620	30.940	1.0	-5.33	PROT N
ATOM	438	CA	GLY	B	27	58.720	51.540	30.190	1.0	-1.36	PROT C
ATOM	439	C	GLY	B	27	60.050	52.020	29.560	1.0	5.86	PROT C
ATOM	440	O	GLY	B	27	60.980	51.300	29.170	1.0	-6.03	PROT O
ATOM	441	H	GLY	B	27	57.203	52.954	30.431	1.0	3.36	PROT H
ATOM	442	HA2	GLY	B	27	58.045	51.172	29.383	1.0	1.85	PROT H
ATOM	443	HA3	GLY	B	27	58.947	50.684	30.865	1.0	1.71	PROT H
ATOM	444	N	ALA	B	28	60.150	53.360	29.380	1.0	-5.25	PROT N
ATOM	445	CA	ALA	B	28	61.360	53.990	28.870	1.0	0.09	PROT C
ATOM	446	CB	ALA	B	28	61.020	55.050	27.840	1.0	-4.81	PROT C
ATOM	447	C	ALA	B	28	62.340	54.420	29.960	1.0	5.53	PROT C
ATOM	448	O	ALA	B	28	62.380	53.790	31.010	1.0	-5.67	PROT O
ATOM	449	H	ALA	B	28	59.469	53.991	29.785	1.0	3.20	PROT H
ATOM	450	HA	ALA	B	28	61.942	53.167	28.317	1.0	1.83	PROT H
ATOM	451	HB1	ALA	B	28	60.634	55.977	28.289	1.0	1.73	PROT H
ATOM	452	HB2	ALA	B	28	61.883	55.323	27.213	1.0	1.83	PROT H
ATOM	453	HB3	ALA	B	28	60.235	54.703	27.151	1.0	1.62	PROT H
ATOM	454	N	ASP	B	29	63.090	55.480	29.750	1.0	-5.63	PROT N
ATOM	455	CA	ASP	B	29	64.040	56.060	30.700	1.0	0.35	PROT C
ATOM	456	CB	ASP	B	29	65.410	55.570	30.260	1.0	-4.30	PROT C
ATOM	457	CG	ASP	B	29	65.440	54.160	29.730	1.0	7.73	PROT C
ATOM	458	OD1	ASP	B	29	65.560	54.020	28.520	1.0	-8.46	PROT O
ATOM	459	OD2	ASP	B	29	65.460	53.110	30.440	1.0	-7.96	PROT O
ATOM	460	C	ASP	B	29	64.080	57.590	30.800	1.0	5.79	PROT C
ATOM	461	O	ASP	B	29	64.350	58.140	31.880	1.0	-6.29	PROT O
ATOM	462	H	ASP	B	29	63.156	55.889	28.804	1.0	3.36	PROT H
ATOM	463	HA	ASP	B	29	63.795	55.660	31.735	1.0	2.08	PROT H
ATOM	464	HB2	ASP	B	29	66.130	55.639	31.108	1.0	1.87	PROT H
ATOM	465	HB3	ASP	B	29	65.839	56.234	29.470	1.0	1.87	PROT H
ATOM	466	N	ASP	B	30	63.810	58.220	29.670	1.0	-5.23	PROT N
ATOM	467	CA	ASP	B	30	64.070	59.670	29.370	1.0	0.01	PROT C
ATOM	468	CB	ASP	B	30	64.730	59.880	27.980	1.0	-4.25	PROT C
ATOM	469	CG	ASP	B	30	65.970	59.100	27.830	1.0	7.59	PROT C
ATOM	470	OD1	ASP	B	30	65.950	58.160	26.970	1.0	-8.47	PROT O

ATOM	471	OD2	ASP	B	30	66.960	59.420	28.520	1.0	-7.57	PROT	O
ATOM	472	C	ASP	B	30	62.980	60.640	29.630	1.0	5.47	PROT	C
ATOM	473	O	ASP	B	30	61.790	60.420	29.360	1.0	-5.70	PROT	O
ATOM	474	H	ASP	B	30	63.568	57.708	28.813	1.0	3.38	PROT	H
ATOM	475	HA	ASP	B	30	64.918	59.924	30.117	1.0	2.01	PROT	H
ATOM	476	HB2	ASP	B	30	64.959	60.964	27.845	1.0	1.87	PROT	H
ATOM	477	HB3	ASP	B	30	63.991	59.642	27.185	1.0	1.65	PROT	H
ATOM	478	N	THR	B	31	63.350	61.870	29.970	1.0	-5.59	PROT	N
ATOM	479	CA	THR	B	31	62.510	63.050	29.780	1.0	-0.67	PROT	C
ATOM	480	CB	THR	B	31	62.860	63.920	30.950	1.0	1.77	PROT	C
ATOM	481	CG2	THR	B	31	62.300	65.350	30.930	1.0	-4.89	PROT	C
ATOM	482	OG1	THR	B	31	62.480	63.320	32.130	1.0	-6.16	PROT	O
ATOM	483	C	THR	B	31	62.880	63.760	28.460	1.0	5.75	PROT	C
ATOM	484	O	THR	B	31	64.070	63.950	28.270	1.0	-5.95	PROT	O
ATOM	485	H	THR	B	31	64.349	62.098	30.101	1.0	3.38	PROT	H
ATOM	486	HA	THR	B	31	61.415	62.756	29.790	1.0	2.17	PROT	H
ATOM	487	HB	THR	B	31	63.991	64.027	31.018	1.0	1.38	PROT	H
ATOM	488	HG21	THR	B	31	62.454	65.838	31.899	1.0	1.58	PROT	H
ATOM	489	HG22	THR	B	31	62.788	65.955	30.159	1.0	1.61	PROT	H
ATOM	490	HG23	THR	B	31	61.220	65.356	30.732	1.0	1.66	PROT	H
ATOM	491	HG1	THR	B	31	62.572	62.340	32.106	1.0	3.62	PROT	H
ATOM	492	N	VAL	B	32	61.870	64.130	27.630	1.0	-5.58	PROT	N
ATOM	493	CA	VAL	B	32	61.970	64.760	26.310	1.0	-0.34	PROT	C
ATOM	494	CB	VAL	B	32	61.960	63.660	25.140	1.0	-0.70	PROT	C
ATOM	495	CG1	VAL	B	32	62.660	64.100	23.870	1.0	-4.67	PROT	C
ATOM	496	CG2	VAL	B	32	62.610	62.350	25.490	1.0	-4.45	PROT	C
ATOM	497	C	VAL	B	32	60.830	65.700	26.070	1.0	6.00	PROT	C
ATOM	498	O	VAL	B	32	59.710	65.290	26.200	1.0	-6.25	PROT	O
ATOM	499	H	VAL	B	32	60.895	63.934	27.913	1.0	3.53	PROT	H
ATOM	500	HA	VAL	B	32	62.972	65.278	26.257	1.0	1.72	PROT	H
ATOM	501	HB	VAL	B	32	60.872	63.479	24.918	1.0	1.56	PROT	H
ATOM	502	HG11	VAL	B	32	62.325	65.088	23.529	1.0	1.37	PROT	H
ATOM	503	HG12	VAL	B	32	63.749	64.157	23.989	1.0	1.46	PROT	H
ATOM	504	HG13	VAL	B	32	62.456	63.409	23.040	1.0	1.59	PROT	H
ATOM	505	HG21	VAL	B	32	62.075	61.827	26.298	1.0	1.56	PROT	H
ATOM	506	HG22	VAL	B	32	62.632	61.662	24.634	1.0	1.52	PROT	H
ATOM	507	HG23	VAL	B	32	63.645	62.486	25.836	1.0	1.55	PROT	H
ATOM	508	N	NME	B	33	61.163	66.926	25.729	1.0	-6.38	PROT	N

ATOM	509	H1	NME	B	33	62.110	67.247	25.592	1.0	3.42	PROT	H
ATOM	510	H2	NME	B	33	60.452	67.622	25.544	1.0	3.15	PROT	H
ATOM	511	C	ACE	B	34	69.514	60.929	20.206	1.0	4.40	PROT	C
ATOM	512	O	ACE	B	34	69.790	60.823	21.419	1.0	-6.35	PROT	O
ATOM	513	HC	ACE	B	34	70.019	61.724	19.615	1.0	1.33	PROT	H
ATOM	514	N	ILE	B	47	68.660	60.190	19.590	1.0	-5.14	PROT	N
ATOM	515	CA	ILE	B	47	67.890	59.090	20.300	1.0	-0.27	PROT	C
ATOM	516	CB	ILE	B	47	67.250	59.710	21.550	1.0	-0.93	PROT	C
ATOM	517	CG2	ILE	B	47	68.280	60.200	22.610	1.0	-3.80	PROT	C
ATOM	518	CG1	ILE	B	47	66.180	60.790	21.200	1.0	-2.71	PROT	C
ATOM	519	CD1	ILE	B	47	65.450	61.380	22.440	1.0	-4.32	PROT	C
ATOM	520	C	ILE	B	47	66.900	58.320	19.330	1.0	5.37	PROT	C
ATOM	521	O	ILE	B	47	66.450	58.900	18.320	1.0	-6.42	PROT	O
ATOM	522	H	ILE	B	47	68.364	60.330	18.625	1.0	3.35	PROT	H
ATOM	523	HA	ILE	B	47	68.666	58.341	20.630	1.0	1.71	PROT	H
ATOM	524	HB	ILE	B	47	66.682	58.873	22.048	1.0	1.39	PROT	H
ATOM	525	HG12	ILE	B	47	65.436	60.350	20.513	1.0	1.38	PROT	H
ATOM	526	HG13	ILE	B	47	66.662	61.612	20.645	1.0	1.29	PROT	H
ATOM	527	HG21	ILE	B	47	67.809	60.054	23.607	1.0	1.26	PROT	H
ATOM	528	HG22	ILE	B	47	69.174	59.569	22.668	1.0	1.58	PROT	H
ATOM	529	HG23	ILE	B	47	68.447	61.283	22.597	1.0	1.57	PROT	H
ATOM	530	HD11	ILE	B	47	64.935	60.594	23.004	1.0	1.59	PROT	H
ATOM	531	HD12	ILE	B	47	66.155	61.877	23.113	1.0	1.50	PROT	H
ATOM	532	HD13	ILE	B	47	64.705	62.116	22.127	1.0	1.35	PROT	H
ATOM	533	N	GLY	B	48	66.490	57.150	19.780	1.0	-4.69	PROT	N
ATOM	534	CA	GLY	B	48	65.550	56.470	18.960	1.0	-2.05	PROT	C
ATOM	535	C	GLY	B	48	64.990	55.200	19.600	1.0	5.81	PROT	C
ATOM	536	O	GLY	B	48	65.280	54.820	20.740	1.0	-6.11	PROT	O
ATOM	537	H	GLY	B	48	66.836	56.674	20.616	1.0	3.62	PROT	H
ATOM	538	HA2	GLY	B	48	65.994	56.227	17.955	1.0	1.86	PROT	H
ATOM	539	HA3	GLY	B	48	64.674	57.149	18.722	1.0	2.05	PROT	H
ATOM	540	N	GLY	B	49	64.290	54.460	18.790	1.0	-5.20	PROT	N
ATOM	541	CA	GLY	B	49	63.200	53.600	19.370	1.0	-1.60	PROT	C
ATOM	542	C	GLY	B	49	62.280	53.220	18.260	1.0	5.80	PROT	C
ATOM	543	O	GLY	B	49	62.660	52.940	17.110	1.0	-6.42	PROT	O
ATOM	544	H	GLY	B	49	64.117	54.701	17.808	1.0	3.37	PROT	H
ATOM	545	HA2	GLY	B	49	62.680	54.157	20.198	1.0	2.08	PROT	H
ATOM	546	HA3	GLY	B	49	63.661	52.697	19.843	1.0	1.93	PROT	H

ATOM	547	N	ILE	B	50	60.940	53.370	18.560	1.0	-5.67	PROT	N
ATOM	548	CA	ILE	B	50	59.860	53.090	17.580	1.0	-0.17	PROT	C
ATOM	549	CB	ILE	B	50	58.470	53.440	18.160	1.0	-0.95	PROT	C
ATOM	550	CG2	ILE	B	50	57.360	53.610	17.110	1.0	-4.56	PROT	C
ATOM	551	CG1	ILE	B	50	58.100	52.410	19.170	1.0	-2.73	PROT	C
ATOM	552	CD1	ILE	B	50	56.850	52.710	20.040	1.0	-4.19	PROT	C
ATOM	553	C	ILE	B	50	60.080	53.790	16.180	1.0	5.82	PROT	C
ATOM	554	O	ILE	B	50	59.830	54.970	16.090	1.0	-6.13	PROT	O
ATOM	555	H	ILE	B	50	60.609	53.780	19.451	1.0	3.80	PROT	H
ATOM	556	HA	ILE	B	50	59.890	51.977	17.389	1.0	1.44	PROT	H
ATOM	557	HB	ILE	B	50	58.577	54.444	18.670	1.0	1.66	PROT	H
ATOM	558	HG12	ILE	B	50	58.944	52.243	19.879	1.0	1.44	PROT	H
ATOM	559	HG13	ILE	B	50	57.934	51.427	18.677	1.0	1.32	PROT	H
ATOM	560	HG21	ILE	B	50	57.568	54.442	16.428	1.0	1.50	PROT	H
ATOM	561	HG22	ILE	B	50	57.239	52.709	16.497	1.0	1.52	PROT	H
ATOM	562	HG23	ILE	B	50	56.397	53.805	17.596	1.0	1.57	PROT	H
ATOM	563	HD11	ILE	B	50	56.958	53.667	20.563	1.0	1.56	PROT	H
ATOM	564	HD12	ILE	B	50	55.940	52.755	19.437	1.0	1.41	PROT	H
ATOM	565	HD13	ILE	B	50	56.717	51.930	20.796	1.0	1.43	PROT	H
ATOM	566	N	NME	B	51	60.524	53.013	15.216	1.0	-6.17	PROT	N
ATOM	567	H1	NME	B	51	60.863	52.076	15.382	1.0	3.30	PROT	H
ATOM	568	H2	NME	B	51	60.740	53.376	14.299	1.0	3.17	PROT	H
ATOM	569	C	ACE	B	52	66.973	66.200	27.932	1.0	4.04	PROT	C
ATOM	570	O	ACE	B	52	67.931	65.551	27.462	1.0	-5.95	PROT	O
ATOM	571	HC	ACE	B	52	66.839	66.228	29.037	1.0	1.44	PROT	H
ATOM	572	N	LEU	B	76	66.100	66.820	27.220	1.0	-4.75	PROT	N
ATOM	573	CA	LEU	B	76	66.150	66.820	25.830	1.0	-0.31	PROT	C
ATOM	574	CB	LEU	B	76	66.160	65.350	25.350	1.0	-3.13	PROT	C
ATOM	575	CG	LEU	B	76	67.180	64.410	26.070	1.0	0.22	PROT	C
ATOM	576	CD1	LEU	B	76	67.090	62.990	25.570	1.0	-4.90	PROT	C
ATOM	577	CD2	LEU	B	76	68.610	64.770	25.690	1.0	-4.05	PROT	C
ATOM	578	C	LEU	B	76	64.970	67.650	25.190	1.0	5.85	PROT	C
ATOM	579	O	LEU	B	76	63.870	67.730	25.710	1.0	-6.65	PROT	O
ATOM	580	H	LEU	B	76	65.237	67.206	27.637	1.0	3.36	PROT	H
ATOM	581	HA	LEU	B	76	67.119	67.300	25.480	1.0	1.67	PROT	H
ATOM	582	HB2	LEU	B	76	66.354	65.317	24.263	1.0	1.39	PROT	H
ATOM	583	HB3	LEU	B	76	65.147	64.919	25.503	1.0	1.58	PROT	H
ATOM	584	HG	LEU	B	76	66.853	64.199	27.129	1.0	1.67	PROT	H

```
ATOM    585 HD11 LEU B  76      67.293  62.886  24.497  1.0   1.34      PROT H
ATOM    586 HD12 LEU B  76      67.807  62.340  26.094  1.0   1.60      PROT H
ATOM    587 HD13 LEU B  76      66.094  62.553  25.739  1.0   1.42      PROT H
ATOM    588 HD21 LEU B  76      69.347  64.338  26.381  1.0   1.42      PROT H
ATOM    589 HD22 LEU B  76      68.865  64.392  24.687  1.0   1.14      PROT H
ATOM    590 HD23 LEU B  76      68.808  65.847  25.625  1.0   1.23      PROT H
ATOM    591  N   NME B  77      65.246  68.254  24.055  1.0  -6.06      PROT N
ATOM    592  H1  NME B  77      66.123  68.165  23.565  1.0   3.28      PROT H
ATOM    593  H2  NME B  77      64.546  68.792  23.558  1.0   3.25      PROT H
ATOM    594  C   ACE B  78      60.396  65.595  19.288  1.0   4.60      PROT C
ATOM    595  O   ACE B  78      60.879  64.565  19.802  1.0  -6.50      PROT O
ATOM    596  HC  ACE B  78      61.091  66.323  18.816  1.0   1.44      PROT H
ATOM    597  N   THR B  80      59.140  65.870  19.260  1.0  -4.92      PROT N
ATOM    598  CA  THR B  80      58.110  64.950  19.860  1.0  -0.69      PROT C
ATOM    599  CB  THR B  80      58.420  64.670  21.340  1.0   1.50      PROT C
ATOM    600  CG2 THR B  80      58.380  65.920  22.290  1.0  -4.94      PROT C
ATOM    601  OG1 THR B  80      57.600  63.640  21.960  1.0  -6.02      PROT O
ATOM    602  C   THR B  80      56.660  65.410  19.750  1.0   5.85      PROT C
ATOM    603  O   THR B  80      56.400  66.580  19.700  1.0  -6.24      PROT O
ATOM    604  H   THR B  80      58.746  66.749  18.919  1.0   3.42      PROT H
ATOM    605  HA  THR B  80      58.231  63.968  19.300  1.0   1.93      PROT H
ATOM    606  HB  THR B  80      59.438  64.194  21.399  1.0   1.80      PROT H
ATOM    607 HG21 THR B  80      59.129  66.658  22.001  1.0   1.52      PROT H
ATOM    608 HG22 THR B  80      57.397  66.400  22.269  1.0   1.62      PROT H
ATOM    609 HG23 THR B  80      58.582  65.611  23.325  1.0   1.81      PROT H
ATOM    610  HG1 THR B  80      57.188  63.986  22.786  1.0   3.60      PROT H
ATOM    611  N   PRO B  81      55.660  64.490  19.730  1.0  -4.82      PROT N
ATOM    612  CD  PRO B  81      55.840  63.030  19.680  1.0  -0.51      PROT C
ATOM    613  CG  PRO B  81      54.600  62.530  19.010  1.0  -2.98      PROT C
ATOM    614  CB  PRO B  81      53.620  63.410  19.600  1.0  -2.72      PROT C
ATOM    615  CA  PRO B  81      54.250  64.850  19.770  1.0  -0.02      PROT C
ATOM    616  C   PRO B  81      53.720  65.590  20.990  1.0   5.76      PROT C
ATOM    617  O   PRO B  81      52.880  66.480  20.930  1.0  -6.38      PROT O
ATOM    618  HA  PRO B  81      54.005  65.496  18.879  1.0   1.83      PROT H
ATOM    619  HB2 PRO B  81      53.264  63.054  20.594  1.0   1.58      PROT H
ATOM    620  HB3 PRO B  81      52.685  63.503  19.004  1.0   1.62      PROT H
ATOM    621  HG2 PRO B  81      54.415  61.447  19.192  1.0   1.76      PROT H
ATOM    622  HG3 PRO B  81      54.663  62.602  17.900  1.0   1.63      PROT H
```

ATOM	623	HD2	PRO	B	81	55.975	62.624	20.716	1.0	1.73	PROT	H
ATOM	624	HD3	PRO	B	81	56.771	62.766	19.127	1.0	1.51	PROT	H
ATOM	625	N	VAL	B	82	54.230	65.260	22.190	1.0	-5.49	PROT	N
ATOM	626	CA	VAL	B	82	53.820	65.720	23.540	1.0	0.08	PROT	C
ATOM	627	CB	VAL	B	82	52.850	64.720	24.130	1.0	-0.95	PROT	C
ATOM	628	CG1	VAL	B	82	51.420	64.860	23.580	1.0	-4.45	PROT	C
ATOM	629	CG2	VAL	B	82	53.230	63.260	23.930	1.0	-4.39	PROT	C
ATOM	630	C	VAL	B	82	55.080	65.810	24.420	1.0	5.44	PROT	C
ATOM	631	O	VAL	B	82	56.020	65.050	24.200	1.0	-6.11	PROT	O
ATOM	632	H	VAL	B	82	54.995	64.574	22.237	1.0	3.33	PROT	H
ATOM	633	HA	VAL	B	82	53.334	66.731	23.437	1.0	1.81	PROT	H
ATOM	634	HB	VAL	B	82	52.802	64.913	25.242	1.0	1.55	PROT	H
ATOM	635	HG11	VAL	B	82	51.006	65.855	23.778	1.0	1.38	PROT	H
ATOM	636	HG12	VAL	B	82	51.393	64.717	22.493	1.0	1.56	PROT	H
ATOM	637	HG13	VAL	B	82	50.746	64.131	24.039	1.0	1.46	PROT	H
ATOM	638	HG21	VAL	B	82	52.627	62.603	24.568	1.0	1.47	PROT	H
ATOM	639	HG22	VAL	B	82	53.100	62.929	22.894	1.0	1.46	PROT	H
ATOM	640	HG23	VAL	B	82	54.282	63.075	24.202	1.0	1.62	PROT	H
ATOM	641	N	ASN	B	83	55.170	66.680	25.390	1.0	-4.97	PROT	N
ATOM	642	CA	ASN	B	83	56.170	66.540	26.410	1.0	0.39	PROT	C
ATOM	643	CB	ASN	B	83	56.000	67.740	27.320	1.0	-3.90	PROT	C
ATOM	644	CG	ASN	B	83	56.150	69.160	26.790	1.0	5.95	PROT	C
ATOM	645	OD1	ASN	B	83	57.210	69.580	26.270	1.0	-6.88	PROT	O
ATOM	646	ND2	ASN	B	83	55.290	70.060	27.070	1.0	-5.83	PROT	N
ATOM	647	C	ASN	B	83	56.060	65.220	27.260	1.0	5.60	PROT	C
ATOM	648	O	ASN	B	83	54.920	64.960	27.650	1.0	-6.22	PROT	O
ATOM	649	H	ASN	B	83	54.398	67.317	25.609	1.0	3.26	PROT	H
ATOM	650	HA	ASN	B	83	57.194	66.555	25.913	1.0	1.97	PROT	H
ATOM	651	HB2	ASN	B	83	56.768	67.666	28.141	1.0	2.04	PROT	H
ATOM	652	HB3	ASN	B	83	55.028	67.639	27.866	1.0	1.86	PROT	H
ATOM	653	HD21	ASN	B	83	54.390	69.901	27.508	1.0	3.20	PROT	H
ATOM	654	HD22	ASN	B	83	55.442	71.034	26.816	1.0	3.24	PROT	H
ATOM	655	N	ILE	B	84	57.160	64.550	27.560	1.0	-5.28	PROT	N
ATOM	656	CA	ILE	B	84	57.260	63.210	28.200	1.0	-0.35	PROT	C
ATOM	657	CB	ILE	B	84	57.650	62.130	27.130	1.0	-0.82	PROT	C
ATOM	658	CG2	ILE	B	84	57.560	60.790	27.850	1.0	-4.72	PROT	C
ATOM	659	CG1	ILE	B	84	56.690	62.140	25.920	1.0	-2.70	PROT	C
ATOM	660	CD1	ILE	B	84	57.450	61.600	24.730	1.0	-4.40	PROT	C

ATOM	661	C	ILE	B	84	58.290	63.170	29.360	1.0	5.90	PROT	C
ATOM	662	O	ILE	B	84	59.360	63.740	29.270	1.0	-6.51	PROT	O
ATOM	663	H	ILE	B	84	58.080	64.815	27.164	1.0	3.58	PROT	H
ATOM	664	HA	ILE	B	84	56.234	62.962	28.603	1.0	1.80	PROT	H
ATOM	665	HB	ILE	B	84	58.691	62.335	26.787	1.0	1.59	PROT	H
ATOM	666	HG12	ILE	B	84	56.322	63.161	25.698	1.0	1.53	PROT	H
ATOM	667	HG13	ILE	B	84	55.784	61.545	26.130	1.0	1.37	PROT	H
ATOM	668	HG21	ILE	B	84	56.609	60.664	28.380	1.0	1.46	PROT	H
ATOM	669	HG22	ILE	B	84	57.636	59.956	27.132	1.0	1.71	PROT	H
ATOM	670	HG23	ILE	B	84	58.378	60.648	28.567	1.0	1.56	PROT	H
ATOM	671	HD11	ILE	B	84	57.886	60.612	24.927	1.0	1.51	PROT	H
ATOM	672	HD12	ILE	B	84	56.797	61.487	23.852	1.0	1.46	PROT	H
ATOM	673	HD13	ILE	B	84	58.258	62.282	24.428	1.0	1.51	PROT	H
ATOM	674	N	NME	B	85	57.926	62.488	30.424	1.0	-6.15	PROT	N
ATOM	675	H1	NME	B	85	57.047	61.996	30.500	1.0	3.44	PROT	H
ATOM	676	H2	NME	B	85	58.567	62.360	31.198	1.0	3.24	PROT	H
HETATM	677	C1	AMP	C	100	50.780	55.200	21.820	1.0	-0.48	PROT	C
HETATM	678	C2	AMP	C	100	51.350	52.860	21.090	1.0	-3.50	PROT	C
HETATM	679	C3	AMP	C	100	54.100	54.870	22.340	1.0	7.65	PROT	C
HETATM	680	C4	AMP	C	100	51.900	54.260	21.310	1.0	1.43	PROT	C
HETATM	681	C5	AMP	C	100	55.990	55.360	23.800	1.0	0.27	PROT	C
HETATM	682	C6	AMP	C	100	56.910	54.380	24.450	1.0	1.44	PROT	C
HETATM	683	C7	AMP	C	100	55.560	56.650	24.660	1.0	-2.99	PROT	C
HETATM	684	C8	AMP	C	100	55.120	57.880	23.740	1.0	0.22	PROT	C
HETATM	685	C9	AMP	C	100	56.050	58.530	22.910	1.0	-1.72	PROT	C
HETATM	686	C10	AMP	C	100	53.750	58.310	23.720	1.0	-1.71	PROT	C
HETATM	687	C11	AMP	C	100	55.750	59.670	22.160	1.0	-1.59	PROT	C
HETATM	688	C12	AMP	C	100	53.460	59.400	22.830	1.0	-1.38	PROT	C
HETATM	689	C13	AMP	C	100	54.450	60.090	22.120	1.0	-1.79	PROT	C
HETATM	690	C14	AMP	C	100	58.380	55.040	24.660	1.0	-1.32	PROT	C
HETATM	691	C15	AMP	C	100	60.410	53.620	24.200	1.0	-0.91	PROT	C
HETATM	692	C16	AMP	C	100	59.800	52.310	24.750	1.0	-0.83	PROT	C
HETATM	693	C17	AMP	C	100	61.330	56.280	23.080	1.0	-5.88	PROT	C
HETATM	694	C18	AMP	C	100	61.040	57.240	24.120	1.0	-0.03	PROT	C
HETATM	695	C19	AMP	C	100	62.120	57.930	24.680	1.0	-2.85	PROT	C
HETATM	696	C20	AMP	C	100	63.400	57.790	24.030	1.0	3.34	PROT	C
HETATM	697	C21	AMP	C	100	63.710	56.980	22.990	1.0	-3.87	PROT	C
HETATM	698	C22	AMP	C	100	62.620	56.170	22.470	1.0	0.83	PROT	C

HETATM	699	C23	AMP	C	100	59.170	51.480	23.560	1.0	-4.48	PROT	C
HETATM	700	C24	AMP	C	100	60.780	51.460	25.640	1.0	-4.60	PROT	C
HETATM	701	C25	AMP	C	100	49.860	52.980	21.520	1.0	0.04	PROT	C
HETATM	702	N1	AMP	C	100	54.790	54.670	23.490	1.0	-5.76	PROT	N
HETATM	703	N2	AMP	C	100	59.330	54.510	23.610	1.0	-7.63	PROT	N
HETATM	704	N3	AMP	C	100	64.390	58.520	24.530	1.0	-5.87	PROT	N
HETATM	705	O1	AMP	C	100	52.990	54.070	22.360	1.0	-5.00	PROT	O
HETATM	706	O2	AMP	C	100	54.390	55.610	21.480	1.0	-6.33	PROT	O
HETATM	707	O3	AMP	C	100	56.420	53.930	25.640	1.0	-6.22	PROT	O
HETATM	708	O4	AMP	C	100	58.830	56.570	22.240	1.0	-9.86	PROT	O
HETATM	709	O5	AMP	C	100	60.280	54.670	21.350	1.0	-9.91	PROT	O
HETATM	710	O6	AMP	C	100	49.910	54.170	22.360	1.0	-4.40	PROT	O
HETATM	711	S1	AMP	C	100	59.890	55.540	22.440	1.0	24.32	PROT	S
HETATM	712	HO3	AMP	C	100	56.430	54.599	26.384	1.0	3.83	PROT	H
HETATM	713	H11A	AMP	C	100	50.274	55.759	21.028	1.0	1.45	PROT	H
HETATM	714	H12A	AMP	C	100	51.042	55.826	22.681	1.0	1.64	PROT	H
HETATM	715	H21A	AMP	C	100	51.885	52.124	21.723	1.0	1.78	PROT	H
HETATM	716	H22A	AMP	C	100	51.465	52.519	20.052	1.0	1.47	PROT	H
HETATM	717	H4	AMP	C	100	52.423	54.674	20.426	1.0	1.28	PROT	H
HETATM	718	H5	AMP	C	100	56.482	55.770	22.860	1.0	1.95	PROT	H
HETATM	719	H6	AMP	C	100	57.028	53.448	23.843	1.0	1.56	PROT	H
HETATM	720	H71	AMP	C	100	56.408	56.978	25.282	1.0	1.69	PROT	H
HETATM	721	H72	AMP	C	100	54.734	56.386	25.330	1.0	1.47	PROT	H
HETATM	722	H9	AMP	C	100	57.076	58.140	22.888	1.0	1.80	PROT	H
HETATM	723	H10	AMP	C	100	52.981	57.825	24.300	1.0	1.76	PROT	H
HETATM	724	H11	AMP	C	100	56.537	60.179	21.608	1.0	1.48	PROT	H
HETATM	725	H12	AMP	C	100	52.425	59.711	22.735	1.0	1.29	PROT	H
HETATM	726	H13	AMP	C	100	54.160	60.950	21.519	1.0	1.36	PROT	H
HETATM	727	H141	AMP	C	100	58.293	56.145	24.622	1.0	1.72	PROT	H
HETATM	728	H142	AMP	C	100	58.719	54.775	25.679	1.0	1.55	PROT	H
HETATM	729	H151	AMP	C	100	61.144	53.358	23.406	1.0	1.52	PROT	H
HETATM	730	H152	AMP	C	100	60.956	54.152	25.009	1.0	1.36	PROT	H
HETATM	731	H16	AMP	C	100	58.940	52.580	25.418	1.0	1.54	PROT	H
HETATM	732	H18	AMP	C	100	60.035	57.385	24.518	1.0	1.76	PROT	H
HETATM	733	H19	AMP	C	100	62.003	58.580	25.535	1.0	1.73	PROT	H
HETATM	734	H21	AMP	C	100	64.685	56.889	22.536	1.0	1.72	PROT	H
HETATM	735	H22	AMP	C	100	62.804	55.494	21.644	1.0	1.48	PROT	H
HETATM	736	H231	AMP	C	100	58.452	52.101	23.015	1.0	1.64	PROT	H

```
HETATM  737 H232 AMP C 100      59.942  51.163  22.859  1.0   1.28      PROT H
HETATM  738 H233 AMP C 100      58.660  50.598  23.948  1.0   1.37      PROT H
HETATM  739 H241 AMP C 100      61.066  52.018  26.538  1.0   1.64      PROT H
HETATM  740 H242 AMP C 100      60.280  50.544  25.964  1.0   1.44      PROT H
HETATM  741 H243 AMP C 100      61.673  51.193  25.077  1.0   1.28      PROT H
HETATM  742 H251 AMP C 100      49.515  52.185  22.191  1.0   1.46      PROT H
HETATM  743 H252 AMP C 100      49.166  53.148  20.689  1.0   1.42      PROT H
HETATM  744  HN1 AMP C 100      54.360  54.149  24.264  1.0   3.47      PROT H
HETATM  745 HN31 AMP C 100      65.328  58.414  24.162  1.0   2.99      PROT H
HETATM  746 HN32 AMP C 100      64.419  58.779  25.511  1.0   3.20      PROT H
HETATM  747  OW  SOL D2989      52.800  57.460  20.120  1.0  -7.59      PROT O
HETATM  748  HW1 SOL D2989      53.284  56.900  20.732  1.0   3.70      PROT H
HETATM  749  HW2 SOL D2989      53.407  57.630  19.403  1.0   3.64      PROT H
HETATM  750  OW  SOL D9011      63.900  56.440  27.060  1.0  -7.79      PROT O
HETATM  751  HW1 SOL D9011      64.617  57.114  26.924  1.0   3.92      PROT H
HETATM  752  HW2 SOL D9011      64.397  55.621  27.198  1.0   3.62      PROT H
HETATM  753  OW  SOL D9015      58.550  57.190  26.780  1.0  -7.47      PROT O
HETATM  754  HW1 SOL D9015      57.864  56.589  27.138  1.0   3.81      PROT H
HETATM  755  HW2 SOL D9015      58.857  57.696  27.522  1.0   3.37      PROT H
END
```

4.2 DAR-HIV^{Pro}

```
HEADER  data-set: HIV_DAR_full_wH2O_OPT
REMARK  MOPAC, Version: 23.1.2
REMARK  99
REMARK  99 MOE v2014.09 (Chemical Computing Group Inc.)
ATOM      1  C   ACE A   7      65.617  50.276  34.888  1.0   4.56      PROT C
ATOM      2  O   ACE A   7      65.798  51.095  33.963  1.0  -6.45      PROT O
ATOM      3  HC  ACE A   7      66.310  50.309  35.757  1.0   1.46      PROT H
ATOM      4  N   ARG A   8      64.677  49.398  34.905  1.0  -5.03      PROT N
ATOM      5  CA  ARG A   8      63.710  49.279  33.813  1.0  -0.17      PROT C
ATOM      6  CB  ARG A   8      64.579  48.996  32.556  1.0  -2.94      PROT C
ATOM      7  CG  ARG A   8      63.753  48.861  31.231  1.0  -3.11      PROT C
ATOM      8  CD  ARG A   8      64.458  48.541  29.923  1.0  -0.51      PROT C
ATOM      9  NE  ARG A   8      65.509  49.520  29.700  1.0  -5.22      PROT N
ATOM     10  CZ  ARG A   8      66.796  49.345  29.559  1.0   6.26      PROT C
ATOM     11  NH1 ARG A   8      67.340  48.173  29.620  1.0  -6.28      PROT N
```

ATOM	12	NH2	ARG	A	8	67.635	50.335	29.404	1.0	-6.34	PROT	N
ATOM	13	C	ARG	A	8	62.736	48.178	34.102	1.0	5.56	PROT	C
ATOM	14	O	ARG	A	8	63.113	47.006	34.328	1.0	-6.60	PROT	O
ATOM	15	H	ARG	A	8	64.603	48.685	35.639	1.0	3.36	PROT	H
ATOM	16	HA	ARG	A	8	63.199	50.282	33.671	1.0	2.03	PROT	H
ATOM	17	HB2	ARG	A	8	65.170	48.078	32.713	1.0	1.45	PROT	H
ATOM	18	HB3	ARG	A	8	65.305	49.826	32.406	1.0	2.00	PROT	H
ATOM	19	HG2	ARG	A	8	63.199	49.825	31.107	1.0	1.83	PROT	H
ATOM	20	HG3	ARG	A	8	62.972	48.086	31.394	1.0	1.57	PROT	H
ATOM	21	HD2	ARG	A	8	63.731	48.607	29.068	1.0	1.79	PROT	H
ATOM	22	HD3	ARG	A	8	64.839	47.496	29.919	1.0	1.44	PROT	H
ATOM	23	HE	ARG	A	8	65.149	50.525	29.678	1.0	3.98	PROT	H
ATOM	24	HH11	ARG	A	8	66.825	47.322	29.825	1.0	3.36	PROT	H
ATOM	25	HH12	ARG	A	8	68.334	48.007	29.494	1.0	3.44	PROT	H
ATOM	26	HH21	ARG	A	8	67.249	51.313	29.254	1.0	3.98	PROT	H
ATOM	27	HH22	ARG	A	8	68.605	50.217	29.145	1.0	3.46	PROT	H
ATOM	28	N	NME	A	9	61.467	48.524	34.102	1.0	-5.87	PROT	N
ATOM	29	H1	NME	A	9	61.131	49.458	33.898	1.0	3.38	PROT	H
ATOM	30	H2	NME	A	9	60.731	47.851	34.271	1.0	3.24	PROT	H
ATOM	31	C	ACE	A	10	58.563	44.669	27.905	1.0	4.91	PROT	C
ATOM	32	O	ACE	A	10	59.770	44.923	28.101	1.0	-6.99	PROT	O
ATOM	33	HC	ACE	A	10	58.311	43.855	27.188	1.0	1.57	PROT	H
ATOM	34	N	LEU	A	23	57.590	45.297	28.465	1.0	-4.83	PROT	N
ATOM	35	CA	LEU	A	23	57.817	46.390	29.405	1.0	-0.47	PROT	C
ATOM	36	CB	LEU	A	23	58.524	47.511	28.523	1.0	-3.11	PROT	C
ATOM	37	CG	LEU	A	23	59.148	48.677	29.162	1.0	-0.74	PROT	C
ATOM	38	CD1	LEU	A	23	60.473	48.325	29.857	1.0	-4.51	PROT	C
ATOM	39	CD2	LEU	A	23	59.430	49.637	28.039	1.0	-4.57	PROT	C
ATOM	40	C	LEU	A	23	56.542	46.992	29.949	1.0	5.85	PROT	C
ATOM	41	O	LEU	A	23	55.631	47.356	29.200	1.0	-6.53	PROT	O
ATOM	42	H	LEU	A	23	56.625	45.212	28.121	1.0	3.39	PROT	H
ATOM	43	HA	LEU	A	23	58.517	46.055	30.213	1.0	1.76	PROT	H
ATOM	44	HB2	LEU	A	23	57.747	47.829	27.785	1.0	1.62	PROT	H
ATOM	45	HB3	LEU	A	23	59.298	46.995	27.900	1.0	1.87	PROT	H
ATOM	46	HG	LEU	A	23	58.467	49.151	29.920	1.0	1.49	PROT	H
ATOM	47	HD11	LEU	A	23	60.934	49.224	30.286	1.0	1.59	PROT	H
ATOM	48	HD12	LEU	A	23	60.320	47.604	30.664	1.0	1.26	PROT	H
ATOM	49	HD13	LEU	A	23	61.185	47.879	29.154	1.0	1.42	PROT	H

ATOM	50	HD21	LEU	A	23	58.510	50.029	27.582	1.0	1.49	PROT	H
ATOM	51	HD22	LEU	A	23	60.007	50.517	28.381	1.0	1.81	PROT	H
ATOM	52	HD23	LEU	A	23	60.021	49.183	27.233	1.0	1.41	PROT	H
ATOM	53	N	LEU	A	24	56.363	47.094	31.269	1.0	-5.17	PROT	N
ATOM	54	CA	LEU	A	24	55.309	47.926	31.848	1.0	0.01	PROT	C
ATOM	55	CB	LEU	A	24	55.333	47.674	33.428	1.0	-3.01	PROT	C
ATOM	56	CG	LEU	A	24	55.218	46.200	33.863	1.0	-0.52	PROT	C
ATOM	57	CD1	LEU	A	24	55.253	46.106	35.400	1.0	-4.64	PROT	C
ATOM	58	CD2	LEU	A	24	53.914	45.600	33.448	1.0	-4.66	PROT	C
ATOM	59	C	LEU	A	24	55.537	49.399	31.534	1.0	5.57	PROT	C
ATOM	60	O	LEU	A	24	56.636	49.887	31.841	1.0	-5.91	PROT	O
ATOM	61	H	LEU	A	24	57.110	46.842	31.920	1.0	3.26	PROT	H
ATOM	62	HA	LEU	A	24	54.317	47.572	31.450	1.0	1.78	PROT	H
ATOM	63	HB2	LEU	A	24	54.503	48.260	33.866	1.0	1.62	PROT	H
ATOM	64	HB3	LEU	A	24	56.265	48.115	33.831	1.0	1.63	PROT	H
ATOM	65	HG	LEU	A	24	56.073	45.618	33.443	1.0	1.34	PROT	H
ATOM	66	HD11	LEU	A	24	56.174	46.534	35.811	1.0	1.45	PROT	H
ATOM	67	HD12	LEU	A	24	54.412	46.640	35.857	1.0	1.50	PROT	H
ATOM	68	HD13	LEU	A	24	55.203	45.065	35.734	1.0	1.43	PROT	H
ATOM	69	HD21	LEU	A	24	53.799	44.567	33.805	1.0	1.44	PROT	H
ATOM	70	HD22	LEU	A	24	53.051	46.163	33.832	1.0	1.54	PROT	H
ATOM	71	HD23	LEU	A	24	53.800	45.557	32.354	1.0	1.51	PROT	H
ATOM	72	N	ASH	A	25	54.560	50.096	30.943	1.0	-5.61	PROT	N
ATOM	73	CA	ASH	A	25	54.656	51.495	30.412	1.0	0.53	PROT	C
ATOM	74	CB	ASH	A	25	54.594	51.265	28.896	1.0	-3.58	PROT	C
ATOM	75	CG	ASH	A	25	54.734	52.495	28.096	1.0	5.87	PROT	C
ATOM	76	OD1	ASH	A	25	53.853	53.315	27.860	1.0	-5.06	PROT	O
ATOM	77	OD2	ASH	A	25	56.005	52.787	27.818	1.0	-5.39	PROT	O
ATOM	78	C	ASH	A	25	53.423	52.223	30.914	1.0	5.88	PROT	C
ATOM	79	O	ASH	A	25	52.400	51.682	31.246	1.0	-5.84	PROT	O
ATOM	80	H	ASH	A	25	53.639	49.672	30.790	1.0	3.42	PROT	H
ATOM	81	HA	ASH	A	25	55.619	51.979	30.730	1.0	2.13	PROT	H
ATOM	82	HB2	ASH	A	25	53.660	50.712	28.630	1.0	1.86	PROT	H
ATOM	83	HB3	ASH	A	25	55.436	50.573	28.612	1.0	2.14	PROT	H
ATOM	84	HD1	ASH	A	25	54.217	54.160	27.385	1.0	4.18	PROT	H
ATOM	85	N	THR	A	26	53.484	53.609	30.895	1.0	-5.80	PROT	N
ATOM	86	CA	THR	A	26	52.234	54.385	31.144	1.0	-0.62	PROT	C
ATOM	87	CB	THR	A	26	52.258	55.130	32.538	1.0	1.78	PROT	C

ATOM	88	CG2	THR	A	26	52.003	54.107	33.628	1.0	-4.87	PROT	C
ATOM	89	OG1	THR	A	26	53.569	55.614	32.868	1.0	-6.21	PROT	O
ATOM	90	C	THR	A	26	51.843	55.448	30.154	1.0	5.65	PROT	C
ATOM	91	O	THR	A	26	50.688	55.846	30.188	1.0	-5.89	PROT	O
ATOM	92	H	THR	A	26	54.355	54.117	30.780	1.0	3.51	PROT	H
ATOM	93	HA	THR	A	26	51.372	53.644	31.177	1.0	2.20	PROT	H
ATOM	94	HB	THR	A	26	51.545	55.979	32.552	1.0	1.40	PROT	H
ATOM	95	HG21	THR	A	26	52.733	53.284	33.576	1.0	1.76	PROT	H
ATOM	96	HG22	THR	A	26	52.109	54.555	34.624	1.0	1.55	PROT	H
ATOM	97	HG23	THR	A	26	51.000	53.670	33.564	1.0	1.61	PROT	H
ATOM	98	HG1	THR	A	26	53.701	56.495	32.443	1.0	3.46	PROT	H
ATOM	99	N	GLY	A	27	52.704	55.873	29.240	1.0	-5.44	PROT	N
ATOM	100	CA	GLY	A	27	52.408	56.852	28.194	1.0	-1.46	PROT	C
ATOM	101	C	GLY	A	27	51.785	56.281	26.927	1.0	5.90	PROT	C
ATOM	102	O	GLY	A	27	51.102	57.047	26.186	1.0	-6.14	PROT	O
ATOM	103	H	GLY	A	27	53.678	55.561	29.258	1.0	3.26	PROT	H
ATOM	104	HA2	GLY	A	27	53.345	57.398	27.911	1.0	1.87	PROT	H
ATOM	105	HA3	GLY	A	27	51.693	57.627	28.590	1.0	2.02	PROT	H
ATOM	106	N	ALA	A	28	51.897	54.969	26.679	1.0	-5.30	PROT	N
ATOM	107	CA	ALA	A	28	51.171	54.255	25.603	1.0	0.17	PROT	C
ATOM	108	CB	ALA	A	28	51.938	52.982	25.199	1.0	-4.72	PROT	C
ATOM	109	C	ALA	A	28	49.664	53.864	25.850	1.0	5.69	PROT	C
ATOM	110	O	ALA	A	28	49.091	54.113	26.891	1.0	-5.39	PROT	O
ATOM	111	H	ALA	A	28	52.171	54.350	27.443	1.0	3.23	PROT	H
ATOM	112	HA	ALA	A	28	51.139	54.971	24.724	1.0	1.80	PROT	H
ATOM	113	HB1	ALA	A	28	51.386	52.357	24.491	1.0	1.67	PROT	H
ATOM	114	HB2	ALA	A	28	52.900	53.233	24.736	1.0	1.60	PROT	H
ATOM	115	HB3	ALA	A	28	52.149	52.348	26.073	1.0	1.70	PROT	H
ATOM	116	N	ASP	A	29	49.021	53.288	24.807	1.0	-6.01	PROT	N
ATOM	117	CA	ASP	A	29	47.541	53.290	24.677	1.0	0.12	PROT	C
ATOM	118	CB	ASP	A	29	47.117	54.168	23.513	1.0	-3.81	PROT	C
ATOM	119	CG	ASP	A	29	47.665	55.677	23.573	1.0	7.50	PROT	C
ATOM	120	OD1	ASP	A	29	47.422	56.381	24.569	1.0	-7.24	PROT	O
ATOM	121	OD2	ASP	A	29	48.258	56.199	22.599	1.0	-8.45	PROT	O
ATOM	122	C	ASP	A	29	46.967	51.851	24.741	1.0	5.75	PROT	C
ATOM	123	O	ASP	A	29	46.054	51.619	25.550	1.0	-6.18	PROT	O
ATOM	124	H	ASP	A	29	49.521	53.056	23.957	1.0	3.06	PROT	H
ATOM	125	HA	ASP	A	29	47.137	53.796	25.625	1.0	2.18	PROT	H

ATOM	126	HB2	ASP	A	29	46.010	54.259	23.487	1.0	2.00	PROT	H
ATOM	127	HB3	ASP	A	29	47.406	53.749	22.534	1.0	1.68	PROT	H
ATOM	128	N	ASP	A	30	47.456	51.044	23.852	1.0	-4.52	PROT	N
ATOM	129	CA	ASP	A	30	47.233	49.638	23.795	1.0	-0.11	PROT	C
ATOM	130	CB	ASP	A	30	46.427	49.220	22.608	1.0	-4.39	PROT	C
ATOM	131	CG	ASP	A	30	45.306	50.226	22.301	1.0	7.63	PROT	C
ATOM	132	OD1	ASP	A	30	44.104	49.816	22.602	1.0	-8.79	PROT	O
ATOM	133	OD2	ASP	A	30	45.481	51.278	21.682	1.0	-7.46	PROT	O
ATOM	134	C	ASP	A	30	48.532	48.832	23.955	1.0	5.75	PROT	C
ATOM	135	O	ASP	A	30	49.625	49.366	23.906	1.0	-6.21	PROT	O
ATOM	136	H	ASP	A	30	48.170	51.365	23.196	1.0	3.10	PROT	H
ATOM	137	HA	ASP	A	30	46.598	49.370	24.724	1.0	2.06	PROT	H
ATOM	138	HB2	ASP	A	30	45.952	48.228	22.754	1.0	1.78	PROT	H
ATOM	139	HB3	ASP	A	30	47.040	49.118	21.687	1.0	1.74	PROT	H
ATOM	140	N	THR	A	31	48.330	47.516	24.099	1.0	-5.41	PROT	N
ATOM	141	CA	THR	A	31	49.396	46.527	24.258	1.0	-0.85	PROT	C
ATOM	142	CB	THR	A	31	48.911	45.359	25.120	1.0	1.89	PROT	C
ATOM	143	CG2	THR	A	31	49.979	44.284	25.264	1.0	-4.99	PROT	C
ATOM	144	OG1	THR	A	31	48.687	45.846	26.405	1.0	-6.07	PROT	O
ATOM	145	C	THR	A	31	50.039	46.173	22.871	1.0	5.86	PROT	C
ATOM	146	O	THR	A	31	49.257	45.983	21.956	1.0	-5.98	PROT	O
ATOM	147	H	THR	A	31	47.409	47.106	23.909	1.0	3.40	PROT	H
ATOM	148	HA	THR	A	31	50.228	47.035	24.859	1.0	1.98	PROT	H
ATOM	149	HB	THR	A	31	47.951	44.929	24.739	1.0	1.37	PROT	H
ATOM	150	HG21	THR	A	31	50.947	44.711	25.574	1.0	1.79	PROT	H
ATOM	151	HG22	THR	A	31	49.699	43.551	26.030	1.0	1.61	PROT	H
ATOM	152	HG23	THR	A	31	50.143	43.743	24.325	1.0	1.63	PROT	H
ATOM	153	HG1	THR	A	31	48.153	46.673	26.397	1.0	3.53	PROT	H
ATOM	154	N	VAL	A	32	51.385	46.065	22.804	1.0	-5.84	PROT	N
ATOM	155	CA	VAL	A	32	52.227	46.076	21.580	1.0	-0.01	PROT	C
ATOM	156	CB	VAL	A	32	53.076	47.307	21.405	1.0	-0.76	PROT	C
ATOM	157	CG1	VAL	A	32	53.480	47.296	19.959	1.0	-4.68	PROT	C
ATOM	158	CG2	VAL	A	32	52.285	48.583	21.661	1.0	-4.53	PROT	C
ATOM	159	C	VAL	A	32	53.066	44.789	21.599	1.0	5.75	PROT	C
ATOM	160	O	VAL	A	32	53.761	44.487	22.584	1.0	-6.53	PROT	O
ATOM	161	H	VAL	A	32	51.949	46.153	23.662	1.0	3.57	PROT	H
ATOM	162	HA	VAL	A	32	51.489	46.020	20.710	1.0	1.88	PROT	H
ATOM	163	HB	VAL	A	32	53.972	47.279	22.074	1.0	1.46	PROT	H

ATOM	164	HG11	VAL	A	32	52.622	47.278	19.269	1.0	1.63	PROT H
ATOM	165	HG12	VAL	A	32	54.075	48.183	19.691	1.0	1.58	PROT H
ATOM	166	HG13	VAL	A	32	54.109	46.433	19.696	1.0	1.49	PROT H
ATOM	167	HG21	VAL	A	32	51.377	48.624	21.043	1.0	1.58	PROT H
ATOM	168	HG22	VAL	A	32	51.960	48.657	22.709	1.0	1.57	PROT H
ATOM	169	HG23	VAL	A	32	52.890	49.468	21.431	1.0	1.43	PROT H
ATOM	170	N	NME	A	33	52.994	44.045	20.517	1.0	-6.17	PROT N
ATOM	171	H1	NME	A	33	53.557	43.209	20.435	1.0	3.17	PROT H
ATOM	172	H2	NME	A	33	52.473	44.286	19.681	1.0	3.44	PROT H
ATOM	173	C	ACE	A	34	49.380	52.134	14.270	1.0	4.40	PROT C
ATOM	174	O	ACE	A	34	48.581	52.031	15.225	1.0	-6.32	PROT O
ATOM	175	HC	ACE	A	34	49.155	51.580	13.332	1.0	1.36	PROT H
ATOM	176	N	ILE	A	47	50.461	52.829	14.302	1.0	-5.08	PROT N
ATOM	177	CA	ILE	A	47	50.868	53.591	15.506	1.0	-0.28	PROT C
ATOM	178	CB	ILE	A	47	50.981	52.678	16.815	1.0	-0.86	PROT C
ATOM	179	CG2	ILE	A	47	49.505	52.254	17.064	1.0	-4.06	PROT C
ATOM	180	CG1	ILE	A	47	51.860	51.418	16.661	1.0	-2.74	PROT C
ATOM	181	CD1	ILE	A	47	52.059	50.658	17.987	1.0	-4.39	PROT C
ATOM	182	C	ILE	A	47	52.190	54.262	15.117	1.0	5.48	PROT C
ATOM	183	O	ILE	A	47	53.032	53.749	14.342	1.0	-6.39	PROT O
ATOM	184	H	ILE	A	47	51.164	52.807	13.554	1.0	3.43	PROT H
ATOM	185	HA	ILE	A	47	50.078	54.364	15.718	1.0	1.83	PROT H
ATOM	186	HB	ILE	A	47	51.362	53.313	17.637	1.0	1.37	PROT H
ATOM	187	HG12	ILE	A	47	52.846	51.706	16.247	1.0	1.38	PROT H
ATOM	188	HG13	ILE	A	47	51.414	50.740	15.909	1.0	1.47	PROT H
ATOM	189	HG21	ILE	A	47	49.369	52.153	18.162	1.0	1.23	PROT H
ATOM	190	HG22	ILE	A	47	48.773	53.028	16.809	1.0	1.67	PROT H
ATOM	191	HG23	ILE	A	47	49.288	51.237	16.712	1.0	1.75	PROT H
ATOM	192	HD11	ILE	A	47	51.102	50.300	18.385	1.0	1.55	PROT H
ATOM	193	HD12	ILE	A	47	52.705	49.785	17.837	1.0	1.47	PROT H
ATOM	194	HD13	ILE	A	47	52.524	51.295	18.745	1.0	1.40	PROT H
ATOM	195	N	GLY	A	48	52.469	55.382	15.781	1.0	-5.26	PROT N
ATOM	196	CA	GLY	A	48	53.823	55.919	16.004	1.0	-1.48	PROT C
ATOM	197	C	GLY	A	48	53.937	57.375	16.624	1.0	6.16	PROT C
ATOM	198	O	GLY	A	48	53.021	57.998	17.130	1.0	-5.89	PROT O
ATOM	199	H	GLY	A	48	51.774	55.835	16.386	1.0	3.35	PROT H
ATOM	200	HA2	GLY	A	48	54.390	55.902	15.042	1.0	1.84	PROT H
ATOM	201	HA3	GLY	A	48	54.381	55.242	16.705	1.0	1.96	PROT H

ATOM	202	N	GLY	A	49	55.186	57.941	16.570	1.0	-5.98	PROT	N
ATOM	203	CA	GLY	A	49	55.396	59.354	17.020	1.0	-1.26	PROT	C
ATOM	204	C	GLY	A	49	56.854	59.749	17.338	1.0	5.76	PROT	C
ATOM	205	O	GLY	A	49	57.060	60.974	17.564	1.0	-6.16	PROT	O
ATOM	206	H	GLY	A	49	55.979	57.457	16.169	1.0	3.38	PROT	H
ATOM	207	HA2	GLY	A	49	54.777	59.532	17.939	1.0	2.04	PROT	H
ATOM	208	HA3	GLY	A	49	55.022	60.049	16.232	1.0	1.78	PROT	H
ATOM	209	N	ILE	A	50	57.835	58.817	17.439	1.0	-5.60	PROT	N
ATOM	210	CA	ILE	A	50	59.330	59.039	17.354	1.0	-0.20	PROT	C
ATOM	211	CB	ILE	A	50	60.160	58.323	18.475	1.0	-0.88	PROT	C
ATOM	212	CG2	ILE	A	50	61.653	58.852	18.445	1.0	-4.53	PROT	C
ATOM	213	CG1	ILE	A	50	59.572	58.558	19.888	1.0	-2.52	PROT	C
ATOM	214	CD1	ILE	A	50	59.245	60.060	20.128	1.0	-4.45	PROT	C
ATOM	215	C	ILE	A	50	59.770	58.779	15.947	1.0	5.63	PROT	C
ATOM	216	O	ILE	A	50	59.816	57.695	15.390	1.0	-6.31	PROT	O
ATOM	217	H	ILE	A	50	57.603	57.823	17.419	1.0	3.28	PROT	H
ATOM	218	HA	ILE	A	50	59.459	60.151	17.585	1.0	1.91	PROT	H
ATOM	219	HB	ILE	A	50	60.176	57.235	18.252	1.0	1.34	PROT	H
ATOM	220	HG12	ILE	A	50	60.289	58.208	20.654	1.0	1.56	PROT	H
ATOM	221	HG13	ILE	A	50	58.654	57.956	20.025	1.0	1.50	PROT	H
ATOM	222	HG21	ILE	A	50	61.700	59.916	18.686	1.0	1.52	PROT	H
ATOM	223	HG22	ILE	A	50	62.252	58.317	19.189	1.0	1.66	PROT	H
ATOM	224	HG23	ILE	A	50	62.109	58.691	17.468	1.0	1.40	PROT	H
ATOM	225	HD11	ILE	A	50	58.392	60.393	19.529	1.0	1.56	PROT	H
ATOM	226	HD12	ILE	A	50	58.985	60.224	21.181	1.0	1.52	PROT	H
ATOM	227	HD13	ILE	A	50	60.100	60.701	19.901	1.0	1.44	PROT	H
ATOM	228	N	NME	A	51	60.140	59.838	15.260	1.0	-6.24	PROT	N
ATOM	229	H1	NME	A	51	60.447	59.763	14.298	1.0	3.22	PROT	H
ATOM	230	H2	NME	A	51	60.106	60.785	15.608	1.0	3.22	PROT	H
ATOM	231	C	ACE	A	52	47.368	45.341	19.989	1.0	4.64	PROT	C
ATOM	232	O	ACE	A	52	46.703	46.308	19.561	1.0	-6.25	PROT	O
ATOM	233	HC	ACE	A	52	47.037	44.850	20.931	1.0	1.48	PROT	H
ATOM	234	N	LEU	A	76	48.432	44.889	19.427	1.0	-5.08	PROT	N
ATOM	235	CA	LEU	A	76	48.972	45.481	18.205	1.0	-0.22	PROT	C
ATOM	236	CB	LEU	A	76	49.258	46.998	18.376	1.0	-2.90	PROT	C
ATOM	237	CG	LEU	A	76	48.009	47.878	18.670	1.0	-0.29	PROT	C
ATOM	238	CD1	LEU	A	76	48.387	49.303	18.838	1.0	-4.80	PROT	C
ATOM	239	CD2	LEU	A	76	47.183	47.815	17.409	1.0	-4.42	PROT	C

ATOM	240	C	LEU	A	76	50.238	44.711	17.800	1.0	5.60	PROT	C
ATOM	241	O	LEU	A	76	51.368	45.011	18.218	1.0	-6.47	PROT	O
ATOM	242	H	LEU	A	76	49.088	44.296	19.949	1.0	3.28	PROT	H
ATOM	243	HA	LEU	A	76	48.190	45.378	17.395	1.0	1.72	PROT	H
ATOM	244	HB2	LEU	A	76	49.769	47.368	17.469	1.0	1.43	PROT	H
ATOM	245	HB3	LEU	A	76	49.987	47.120	19.203	1.0	1.51	PROT	H
ATOM	246	HG	LEU	A	76	47.524	47.674	19.655	1.0	2.03	PROT	H
ATOM	247	HD11	LEU	A	76	49.058	49.461	19.695	1.0	1.28	PROT	H
ATOM	248	HD12	LEU	A	76	48.902	49.722	17.959	1.0	1.42	PROT	H
ATOM	249	HD13	LEU	A	76	47.503	49.940	19.007	1.0	1.66	PROT	H
ATOM	250	HD21	LEU	A	76	46.789	46.809	17.219	1.0	1.31	PROT	H
ATOM	251	HD22	LEU	A	76	46.300	48.468	17.471	1.0	1.48	PROT	H
ATOM	252	HD23	LEU	A	76	47.752	48.123	16.525	1.0	1.26	PROT	H
ATOM	253	N	NME	A	77	50.055	43.703	16.974	1.0	-6.07	PROT	N
ATOM	254	H1	NME	A	77	49.150	43.402	16.644	1.0	3.27	PROT	H
ATOM	255	H2	NME	A	77	50.824	43.131	16.650	1.0	3.20	PROT	H
ATOM	256	C	ACE	A	78	56.186	46.865	15.193	1.0	4.55	PROT	C
ATOM	257	O	ACE	A	78	55.352	47.671	15.657	1.0	-6.48	PROT	O
ATOM	258	HC	ACE	A	78	55.909	46.295	14.279	1.0	1.47	PROT	H
ATOM	259	N	THR	A	80	57.349	46.655	15.700	1.0	-4.93	PROT	N
ATOM	260	CA	THR	A	80	57.807	47.354	16.888	1.0	-0.49	PROT	C
ATOM	261	CB	THR	A	80	56.812	47.147	18.068	1.0	2.08	PROT	C
ATOM	262	CG2	THR	A	80	56.593	45.654	18.426	1.0	-5.08	PROT	C
ATOM	263	OG1	THR	A	80	57.501	47.795	19.155	1.0	-6.61	PROT	O
ATOM	264	C	THR	A	80	59.227	46.897	17.253	1.0	5.82	PROT	C
ATOM	265	O	THR	A	80	59.570	45.721	17.274	1.0	-6.21	PROT	O
ATOM	266	H	THR	A	80	57.976	45.916	15.355	1.0	3.42	PROT	H
ATOM	267	HA	THR	A	80	57.819	48.467	16.670	1.0	1.87	PROT	H
ATOM	268	HB	THR	A	80	55.866	47.703	17.905	1.0	1.69	PROT	H
ATOM	269	HG21	THR	A	80	57.526	45.082	18.370	1.0	1.68	PROT	H
ATOM	270	HG22	THR	A	80	56.215	45.555	19.453	1.0	1.75	PROT	H
ATOM	271	HG23	THR	A	80	55.869	45.188	17.753	1.0	1.60	PROT	H
ATOM	272	HG1	THR	A	80	57.366	47.328	20.013	1.0	3.79	PROT	H
ATOM	273	N	PRO	A	81	60.196	47.786	17.704	1.0	-4.74	PROT	N
ATOM	274	CD	PRO	A	81	60.195	49.216	17.639	1.0	-0.73	PROT	C
ATOM	275	CG	PRO	A	81	61.631	49.602	17.603	1.0	-3.06	PROT	C
ATOM	276	CB	PRO	A	81	62.341	48.581	18.327	1.0	-2.78	PROT	C
ATOM	277	CA	PRO	A	81	61.439	47.333	18.261	1.0	0.10	PROT	C

ATOM	278	C	PRO	A	81	61.332	46.589	19.609	1.0	5.89	PROT	C
ATOM	279	O	PRO	A	81	62.298	46.134	20.142	1.0	-6.54	PROT	O
ATOM	280	HA	PRO	A	81	61.906	46.568	17.561	1.0	1.82	PROT	H
ATOM	281	HB2	PRO	A	81	62.545	48.853	19.390	1.0	1.68	PROT	H
ATOM	282	HB3	PRO	A	81	63.349	48.349	17.921	1.0	1.61	PROT	H
ATOM	283	HG2	PRO	A	81	61.787	50.631	18.013	1.0	1.72	PROT	H
ATOM	284	HG3	PRO	A	81	61.989	49.708	16.548	1.0	1.58	PROT	H
ATOM	285	HD2	PRO	A	81	59.667	49.642	18.537	1.0	1.62	PROT	H
ATOM	286	HD3	PRO	A	81	59.626	49.591	16.753	1.0	1.53	PROT	H
ATOM	287	N	VAL	A	82	60.210	46.639	20.305	1.0	-5.39	PROT	N
ATOM	288	CA	VAL	A	82	60.004	46.255	21.663	1.0	-0.22	PROT	C
ATOM	289	CB	VAL	A	82	60.495	47.414	22.535	1.0	-0.90	PROT	C
ATOM	290	CG1	VAL	A	82	59.607	48.730	22.361	1.0	-4.50	PROT	C
ATOM	291	CG2	VAL	A	82	60.423	47.197	24.072	1.0	-4.60	PROT	C
ATOM	292	C	VAL	A	82	58.551	45.877	21.983	1.0	5.79	PROT	C
ATOM	293	O	VAL	A	82	57.587	46.413	21.407	1.0	-6.07	PROT	O
ATOM	294	H	VAL	A	82	59.363	47.051	19.865	1.0	3.42	PROT	H
ATOM	295	HA	VAL	A	82	60.664	45.353	21.887	1.0	1.84	PROT	H
ATOM	296	HB	VAL	A	82	61.544	47.649	22.247	1.0	1.56	PROT	H
ATOM	297	HG11	VAL	A	82	59.678	49.109	21.340	1.0	1.42	PROT	H
ATOM	298	HG12	VAL	A	82	58.556	48.511	22.567	1.0	1.59	PROT	H
ATOM	299	HG13	VAL	A	82	59.949	49.509	23.044	1.0	1.57	PROT	H
ATOM	300	HG21	VAL	A	82	60.891	48.036	24.600	1.0	1.61	PROT	H
ATOM	301	HG22	VAL	A	82	59.390	47.119	24.431	1.0	1.59	PROT	H
ATOM	302	HG23	VAL	A	82	60.953	46.290	24.380	1.0	1.40	PROT	H
ATOM	303	N	ASN	A	83	58.404	44.950	22.938	1.0	-5.32	PROT	N
ATOM	304	CA	ASN	A	83	57.058	44.578	23.384	1.0	0.16	PROT	C
ATOM	305	CB	ASN	A	83	57.030	43.056	23.715	1.0	-3.78	PROT	C
ATOM	306	CG	ASN	A	83	57.532	42.217	22.543	1.0	6.02	PROT	C
ATOM	307	OD1	ASN	A	83	56.691	41.733	21.723	1.0	-6.64	PROT	O
ATOM	308	ND2	ASN	A	83	58.829	42.096	22.436	1.0	-5.90	PROT	N
ATOM	309	C	ASN	A	83	56.603	45.490	24.600	1.0	5.77	PROT	C
ATOM	310	O	ASN	A	83	57.305	45.488	25.569	1.0	-6.15	PROT	O
ATOM	311	H	ASN	A	83	59.162	44.711	23.577	1.0	3.36	PROT	H
ATOM	312	HA	ASN	A	83	56.333	44.745	22.526	1.0	2.04	PROT	H
ATOM	313	HB2	ASN	A	83	55.976	42.765	23.934	1.0	2.01	PROT	H
ATOM	314	HB3	ASN	A	83	57.593	42.846	24.644	1.0	1.77	PROT	H
ATOM	315	HD21	ASN	A	83	59.485	42.479	23.099	1.0	3.28	PROT	H

```
ATOM    316 HD22 ASN A  83      59.257  41.581  21.677  1.0   3.20      PROT H
ATOM    317  N   ILE A  84      55.493  46.201  24.461  1.0  -5.51      PROT N
ATOM    318  CA  ILE A  84      54.985  47.220  25.447  1.0  -0.22      PROT C
ATOM    319  CB  ILE A  84      54.873  48.578  24.696  1.0  -0.95      PROT C
ATOM    320  CG2 ILE A  84      54.262  49.707  25.538  1.0  -4.49      PROT C
ATOM    321  CG1 ILE A  84      56.258  48.959  24.102  1.0  -2.64      PROT C
ATOM    322  CD1 ILE A  84      56.253  50.278  23.369  1.0  -4.40      PROT C
ATOM    323  C   ILE A  84      53.655  46.804  26.014  1.0   5.69      PROT C
ATOM    324  O   ILE A  84      52.719  46.378  25.292  1.0  -6.57      PROT O
ATOM    325  H   ILE A  84      54.820  45.986  23.709  1.0   3.55      PROT H
ATOM    326  HA  ILE A  84      55.765  47.314  26.258  1.0   1.82      PROT H
ATOM    327  HB  ILE A  84      54.177  48.411  23.828  1.0   1.52      PROT H
ATOM    328 HG12 ILE A  84      56.587  48.154  23.411  1.0   1.46      PROT H
ATOM    329 HG13 ILE A  84      57.012  48.982  24.914  1.0   1.41      PROT H
ATOM    330 HG21 ILE A  84      54.934  50.007  26.350  1.0   1.51      PROT H
ATOM    331 HG22 ILE A  84      54.080  50.593  24.918  1.0   1.55      PROT H
ATOM    332 HG23 ILE A  84      53.299  49.414  25.971  1.0   1.54      PROT H
ATOM    333 HD11 ILE A  84      55.456  50.331  22.619  1.0   1.38      PROT H
ATOM    334 HD12 ILE A  84      56.140  51.131  24.053  1.0   1.56      PROT H
ATOM    335 HD13 ILE A  84      57.203  50.440  22.839  1.0   1.46      PROT H
ATOM    336  N   NME A  85      53.531  46.914  27.318  1.0  -6.00      PROT N
ATOM    337  H1  NME A  85      52.660  46.686  27.784  1.0   3.27      PROT H
ATOM    338  H2  NME A  85      54.267  47.277  27.913  1.0   3.45      PROT H
TER     339      NME A  85
ATOM    340  C   ACE B   7      44.937  57.999  28.510  1.0   4.35      PROT C
ATOM    341  O   ACE B   7      45.611  57.625  27.528  1.0  -6.01      PROT O
ATOM    342  HC  ACE B   7      43.872  57.682  28.573  1.0   1.46      PROT H
ATOM    343  N   ARG B   8      45.392  58.731  29.465  1.0  -5.08      PROT N
ATOM    344  CA  ARG B   8      46.795  59.209  29.486  1.0  -0.12      PROT C
ATOM    345  CB  ARG B   8      47.149  60.064  28.193  1.0  -2.80      PROT C
ATOM    346  CG  ARG B   8      46.901  59.250  26.891  1.0  -2.65      PROT C
ATOM    347  CD  ARG B   8      47.570  59.871  25.723  1.0  -0.84      PROT C
ATOM    348  NE  ARG B   8      47.332  59.116  24.500  1.0  -5.77      PROT N
ATOM    349  CZ  ARG B   8      47.342  59.603  23.248  1.0   6.23      PROT C
ATOM    350  NH1 ARG B   8      47.335  60.846  22.902  1.0  -6.23      PROT N
ATOM    351  NH2 ARG B   8      47.467  58.770  22.319  1.0  -6.26      PROT N
ATOM    352  C   ARG B   8      47.144  60.079  30.726  1.0   5.51      PROT C
ATOM    353  O   ARG B   8      46.423  61.073  30.955  1.0  -6.50      PROT O
```

ATOM	354	H	ARG	B	8	44.803	59.118	30.205	1.0	3.31	PROT	H
ATOM	355	HA	ARG	B	8	47.446	58.287	29.454	1.0	1.83	PROT	H
ATOM	356	HB2	ARG	B	8	48.205	60.373	28.260	1.0	1.45	PROT	H
ATOM	357	HB3	ARG	B	8	46.540	60.983	28.194	1.0	1.49	PROT	H
ATOM	358	HG2	ARG	B	8	45.812	59.254	26.650	1.0	1.72	PROT	H
ATOM	359	HG3	ARG	B	8	47.303	58.208	27.012	1.0	2.15	PROT	H
ATOM	360	HD2	ARG	B	8	48.684	59.933	25.885	1.0	1.50	PROT	H
ATOM	361	HD3	ARG	B	8	47.239	60.933	25.596	1.0	1.31	PROT	H
ATOM	362	HE	ARG	B	8	47.439	58.045	24.626	1.0	4.14	PROT	H
ATOM	363	HH11	ARG	B	8	47.145	61.598	23.560	1.0	3.29	PROT	H
ATOM	364	HH12	ARG	B	8	47.225	61.150	21.936	1.0	3.42	PROT	H
ATOM	365	HH21	ARG	B	8	47.767	57.749	22.482	1.0	4.03	PROT	H
ATOM	366	HH22	ARG	B	8	47.357	58.974	21.327	1.0	3.41	PROT	H
ATOM	367	N	NME	B	9	48.181	59.681	31.429	1.0	-5.94	PROT	N
ATOM	368	H1	NME	B	9	48.498	60.182	32.248	1.0	3.25	PROT	H
ATOM	369	H2	NME	B	9	48.733	58.864	31.198	1.0	3.36	PROT	H
ATOM	370	C	ACE	B	10	52.537	64.917	28.964	1.0	4.94	PROT	C
ATOM	371	O	ACE	B	10	51.538	64.568	28.302	1.0	-6.94	PROT	O
ATOM	372	HC	ACE	B	10	52.860	65.980	28.905	1.0	1.53	PROT	H
ATOM	373	N	LEU	B	23	53.227	64.124	29.706	1.0	-4.89	PROT	N
ATOM	374	CA	LEU	B	23	52.889	62.688	29.847	1.0	-0.13	PROT	C
ATOM	375	CB	LEU	B	23	52.838	62.041	28.457	1.0	-3.48	PROT	C
ATOM	376	CG	LEU	B	23	52.336	60.627	28.230	1.0	-0.52	PROT	C
ATOM	377	CD1	LEU	B	23	50.916	60.386	28.625	1.0	-4.59	PROT	C
ATOM	378	CD2	LEU	B	23	52.471	60.396	26.729	1.0	-4.58	PROT	C
ATOM	379	C	LEU	B	23	53.839	61.939	30.775	1.0	5.93	PROT	C
ATOM	380	O	LEU	B	23	55.027	62.275	30.788	1.0	-6.60	PROT	O
ATOM	381	H	LEU	B	23	54.178	64.362	30.020	1.0	3.46	PROT	H
ATOM	382	HA	LEU	B	23	51.845	62.650	30.288	1.0	1.77	PROT	H
ATOM	383	HB2	LEU	B	23	53.863	62.129	28.020	1.0	1.64	PROT	H
ATOM	384	HB3	LEU	B	23	52.215	62.710	27.803	1.0	2.04	PROT	H
ATOM	385	HG	LEU	B	23	53.003	59.908	28.776	1.0	1.28	PROT	H
ATOM	386	HD11	LEU	B	23	50.243	61.149	28.205	1.0	1.54	PROT	H
ATOM	387	HD12	LEU	B	23	50.551	59.409	28.265	1.0	1.69	PROT	H
ATOM	388	HD13	LEU	B	23	50.770	60.407	29.711	1.0	1.21	PROT	H
ATOM	389	HD21	LEU	B	23	53.506	60.500	26.383	1.0	1.42	PROT	H
ATOM	390	HD22	LEU	B	23	52.141	59.382	26.446	1.0	1.76	PROT	H
ATOM	391	HD23	LEU	B	23	51.858	61.095	26.148	1.0	1.47	PROT	H

ATOM	392	N	LEU	B	24	53.401	60.903	31.541	1.0	-5.35	PROT	N
ATOM	393	CA	LEU	B	24	54.335	60.196	32.425	1.0	-0.18	PROT	C
ATOM	394	CB	LEU	B	24	53.861	60.301	33.959	1.0	-2.96	PROT	C
ATOM	395	CG	LEU	B	24	53.365	61.697	34.518	1.0	-0.46	PROT	C
ATOM	396	CD1	LEU	B	24	52.742	61.559	35.887	1.0	-4.66	PROT	C
ATOM	397	CD2	LEU	B	24	54.487	62.684	34.383	1.0	-4.69	PROT	C
ATOM	398	C	LEU	B	24	54.575	58.786	31.921	1.0	5.71	PROT	C
ATOM	399	O	LEU	B	24	53.609	58.072	31.634	1.0	-6.06	PROT	O
ATOM	400	H	LEU	B	24	52.480	60.495	31.446	1.0	3.31	PROT	H
ATOM	401	HA	LEU	B	24	55.320	60.764	32.408	1.0	1.92	PROT	H
ATOM	402	HB2	LEU	B	24	54.708	59.959	34.577	1.0	1.57	PROT	H
ATOM	403	HB3	LEU	B	24	53.045	59.571	34.101	1.0	1.55	PROT	H
ATOM	404	HG	LEU	B	24	52.540	62.026	33.821	1.0	1.35	PROT	H
ATOM	405	HD11	LEU	B	24	51.901	60.855	35.883	1.0	1.52	PROT	H
ATOM	406	HD12	LEU	B	24	53.461	61.201	36.635	1.0	1.50	PROT	H
ATOM	407	HD13	LEU	B	24	52.360	62.521	36.249	1.0	1.47	PROT	H
ATOM	408	HD21	LEU	B	24	55.381	62.379	34.944	1.0	1.51	PROT	H
ATOM	409	HD22	LEU	B	24	54.793	62.819	33.336	1.0	1.58	PROT	H
ATOM	410	HD23	LEU	B	24	54.207	63.677	34.759	1.0	1.42	PROT	H
ATOM	411	N	ASP	B	25	55.873	58.294	31.852	1.0	-5.70	PROT	N
ATOM	412	CA	ASP	B	25	56.166	56.954	31.257	1.0	0.11	PROT	C
ATOM	413	CB	ASP	B	25	56.401	57.082	29.760	1.0	-4.32	PROT	C
ATOM	414	CG	ASP	B	25	56.427	55.706	29.058	1.0	7.66	PROT	C
ATOM	415	OD1	ASP	B	25	56.106	54.652	29.704	1.0	-8.20	PROT	O
ATOM	416	OD2	ASP	B	25	56.730	55.688	27.827	1.0	-7.68	PROT	O
ATOM	417	C	ASP	B	25	57.183	56.031	32.086	1.0	5.79	PROT	C
ATOM	418	O	ASP	B	25	58.349	56.278	32.020	1.0	-5.55	PROT	O
ATOM	419	H	ASP	B	25	56.671	58.886	32.038	1.0	3.36	PROT	H
ATOM	420	HA	ASP	B	25	55.175	56.386	31.331	1.0	1.62	PROT	H
ATOM	421	HB2	ASP	B	25	57.360	57.599	29.543	1.0	1.92	PROT	H
ATOM	422	HB3	ASP	B	25	55.613	57.701	29.291	1.0	1.70	PROT	H
ATOM	423	N	THR	B	26	56.624	54.948	32.589	1.0	-5.52	PROT	N
ATOM	424	CA	THR	B	26	57.460	53.896	33.276	1.0	-0.84	PROT	C
ATOM	425	CB	THR	B	26	56.672	52.983	34.230	1.0	1.87	PROT	C
ATOM	426	CG2	THR	B	26	55.769	53.769	35.213	1.0	-4.80	PROT	C
ATOM	427	OG1	THR	B	26	55.752	52.246	33.481	1.0	-6.12	PROT	O
ATOM	428	C	THR	B	26	58.357	53.142	32.307	1.0	5.90	PROT	C
ATOM	429	O	THR	B	26	59.320	52.587	32.786	1.0	-6.39	PROT	O

ATOM	430	H	THR	B	26	55.618	54.780	32.610	1.0	3.49	PROT	H
ATOM	431	HA	THR	B	26	58.190	54.479	33.936	1.0	1.99	PROT	H
ATOM	432	HB	THR	B	26	57.371	52.300	34.764	1.0	1.38	PROT	H
ATOM	433	HG21	THR	B	26	56.323	54.558	35.728	1.0	1.57	PROT	H
ATOM	434	HG22	THR	B	26	54.922	54.229	34.681	1.0	1.77	PROT	H
ATOM	435	HG23	THR	B	26	55.345	53.095	35.963	1.0	1.51	PROT	H
ATOM	436	HG1	THR	B	26	56.189	51.567	32.913	1.0	3.53	PROT	H
ATOM	437	N	GLY	B	27	58.023	53.114	31.043	1.0	-5.41	PROT	N
ATOM	438	CA	GLY	B	27	58.762	52.501	29.912	1.0	-1.56	PROT	C
ATOM	439	C	GLY	B	27	59.839	53.381	29.385	1.0	5.79	PROT	C
ATOM	440	O	GLY	B	27	60.861	52.888	28.915	1.0	-5.82	PROT	O
ATOM	441	H	GLY	B	27	57.191	53.662	30.718	1.0	3.67	PROT	H
ATOM	442	HA2	GLY	B	27	58.011	52.273	29.103	1.0	2.05	PROT	H
ATOM	443	HA3	GLY	B	27	59.196	51.523	30.242	1.0	1.83	PROT	H
ATOM	444	N	ALA	B	28	59.589	54.705	29.499	1.0	-5.38	PROT	N
ATOM	445	CA	ALA	B	28	60.564	55.734	29.146	1.0	0.45	PROT	C
ATOM	446	CB	ALA	B	28	59.970	57.128	29.214	1.0	-4.70	PROT	C
ATOM	447	C	ALA	B	28	61.810	55.622	30.016	1.0	5.94	PROT	C
ATOM	448	O	ALA	B	28	61.739	55.846	31.212	1.0	-6.44	PROT	O
ATOM	449	H	ALA	B	28	58.788	55.045	30.027	1.0	3.34	PROT	H
ATOM	450	HA	ALA	B	28	60.870	55.541	28.060	1.0	1.90	PROT	H
ATOM	451	HB1	ALA	B	28	59.576	57.353	30.218	1.0	1.71	PROT	H
ATOM	452	HB2	ALA	B	28	60.732	57.893	28.992	1.0	1.76	PROT	H
ATOM	453	HB3	ALA	B	28	59.161	57.247	28.481	1.0	1.59	PROT	H
ATOM	454	N	ASP	B	29	63.006	55.423	29.452	1.0	-5.65	PROT	N
ATOM	455	CA	ASP	B	29	64.286	55.560	30.279	1.0	0.95	PROT	C
ATOM	456	CB	ASP	B	29	65.519	54.807	29.766	1.0	-4.35	PROT	C
ATOM	457	CG	ASP	B	29	65.361	53.340	29.437	1.0	7.65	PROT	C
ATOM	458	OD1	ASP	B	29	64.350	52.788	29.883	1.0	-7.36	PROT	O
ATOM	459	OD2	ASP	B	29	66.183	52.632	28.847	1.0	-9.05	PROT	O
ATOM	460	C	ASP	B	29	64.792	57.041	30.336	1.0	5.72	PROT	C
ATOM	461	O	ASP	B	29	65.388	57.420	31.369	1.0	-6.47	PROT	O
ATOM	462	H	ASP	B	29	63.120	55.067	28.509	1.0	3.49	PROT	H
ATOM	463	HA	ASP	B	29	64.030	55.240	31.327	1.0	2.04	PROT	H
ATOM	464	HB2	ASP	B	29	66.320	54.883	30.539	1.0	1.89	PROT	H
ATOM	465	HB3	ASP	B	29	65.931	55.310	28.864	1.0	1.67	PROT	H
ATOM	466	N	ASH	B	30	64.432	57.903	29.329	1.0	-5.62	PROT	N
ATOM	467	CA	ASH	B	30	64.727	59.371	29.366	1.0	0.33	PROT	C

ATOM	468	CB	ASH	B	30	65.628	59.817	28.187	1.0	-3.70	PROT	C
ATOM	469	CG	ASH	B	30	66.790	58.885	27.876	1.0	6.84	PROT	C
ATOM	470	OD1	ASH	B	30	67.844	58.843	28.482	1.0	-5.79	PROT	O
ATOM	471	OD2	ASH	B	30	66.534	57.959	26.904	1.0	-5.36	PROT	O
ATOM	472	C	ASH	B	30	63.442	60.277	29.457	1.0	5.45	PROT	C
ATOM	473	O	ASH	B	30	62.418	59.914	28.913	1.0	-5.45	PROT	O
ATOM	474	H	ASH	B	30	63.793	57.610	28.586	1.0	3.57	PROT	H
ATOM	475	HA	ASH	B	30	65.317	59.538	30.322	1.0	1.87	PROT	H
ATOM	476	HB2	ASH	B	30	66.041	60.828	28.402	1.0	1.89	PROT	H
ATOM	477	HB3	ASH	B	30	65.002	59.930	27.270	1.0	1.92	PROT	H
ATOM	478	HD2	ASH	B	30	67.301	57.341	26.746	1.0	3.79	PROT	H
ATOM	479	N	THR	B	31	63.585	61.470	30.025	1.0	-5.50	PROT	N
ATOM	480	CA	THR	B	31	62.608	62.546	29.786	1.0	-0.98	PROT	C
ATOM	481	CB	THR	B	31	62.542	63.473	30.977	1.0	1.91	PROT	C
ATOM	482	CG2	THR	B	31	61.657	64.691	30.782	1.0	-5.03	PROT	C
ATOM	483	OG1	THR	B	31	62.147	62.884	32.117	1.0	-6.13	PROT	O
ATOM	484	C	THR	B	31	62.920	63.230	28.482	1.0	5.66	PROT	C
ATOM	485	O	THR	B	31	64.043	63.670	28.276	1.0	-6.26	PROT	O
ATOM	486	H	THR	B	31	64.477	61.788	30.409	1.0	3.34	PROT	H
ATOM	487	HA	THR	B	31	61.578	62.036	29.704	1.0	2.24	PROT	H
ATOM	488	HB	THR	B	31	63.596	63.844	31.200	1.0	1.33	PROT	H
ATOM	489	HG21	THR	B	31	62.096	65.413	30.085	1.0	1.58	PROT	H
ATOM	490	HG22	THR	B	31	60.664	64.419	30.388	1.0	1.80	PROT	H
ATOM	491	HG23	THR	B	31	61.489	65.206	31.736	1.0	1.60	PROT	H
ATOM	492	HG1	THR	B	31	62.074	61.904	32.078	1.0	3.66	PROT	H
ATOM	493	N	VAL	B	32	61.884	63.361	27.668	1.0	-5.19	PROT	N
ATOM	494	CA	VAL	B	32	62.014	64.206	26.458	1.0	-0.55	PROT	C
ATOM	495	CB	VAL	B	32	61.824	63.285	25.243	1.0	-0.76	PROT	C
ATOM	496	CG1	VAL	B	32	62.586	63.768	24.018	1.0	-4.67	PROT	C
ATOM	497	CG2	VAL	B	32	62.077	61.766	25.453	1.0	-4.23	PROT	C
ATOM	498	C	VAL	B	32	61.038	65.348	26.547	1.0	5.83	PROT	C
ATOM	499	O	VAL	B	32	59.956	65.193	27.146	1.0	-6.65	PROT	O
ATOM	500	H	VAL	B	32	60.916	63.124	27.935	1.0	3.74	PROT	H
ATOM	501	HA	VAL	B	32	63.076	64.615	26.426	1.0	1.99	PROT	H
ATOM	502	HB	VAL	B	32	60.722	63.351	24.983	1.0	1.50	PROT	H
ATOM	503	HG11	VAL	B	32	63.667	63.615	24.121	1.0	1.47	PROT	H
ATOM	504	HG12	VAL	B	32	62.266	63.235	23.112	1.0	1.60	PROT	H
ATOM	505	HG13	VAL	B	32	62.432	64.836	23.827	1.0	1.42	PROT	H

ATOM	506	HG21	VAL	B	32	63.093	61.586	25.817	1.0	1.45	PROT	H
ATOM	507	HG22	VAL	B	32	61.380	61.346	26.188	1.0	1.57	PROT	H
ATOM	508	HG23	VAL	B	32	61.944	61.232	24.507	1.0	1.49	PROT	H
ATOM	509	N	NME	B	33	61.413	66.467	25.965	1.0	-6.07	PROT	N
ATOM	510	H1	NME	B	33	60.797	67.271	25.944	1.0	3.21	PROT	H
ATOM	511	H2	NME	B	33	62.269	66.567	25.447	1.0	3.46	PROT	H
ATOM	512	C	ACE	B	34	68.969	60.011	20.535	1.0	4.57	PROT	C
ATOM	513	O	ACE	B	34	69.293	59.782	21.718	1.0	-6.52	PROT	O
ATOM	514	HC	ACE	B	34	69.552	60.769	19.966	1.0	1.42	PROT	H
ATOM	515	N	ILE	B	47	67.994	59.434	19.926	1.0	-5.04	PROT	N
ATOM	516	CA	ILE	B	47	67.126	58.393	20.608	1.0	-0.43	PROT	C
ATOM	517	CB	ILE	B	47	66.431	58.989	21.866	1.0	-1.05	PROT	C
ATOM	518	CG2	ILE	B	47	67.491	59.396	22.932	1.0	-4.11	PROT	C
ATOM	519	CG1	ILE	B	47	65.449	60.148	21.543	1.0	-2.79	PROT	C
ATOM	520	CD1	ILE	B	47	64.707	60.775	22.747	1.0	-4.25	PROT	C
ATOM	521	C	ILE	B	47	66.040	57.842	19.717	1.0	5.52	PROT	C
ATOM	522	O	ILE	B	47	65.371	58.498	18.892	1.0	-6.12	PROT	O
ATOM	523	H	ILE	B	47	67.676	59.686	18.990	1.0	3.37	PROT	H
ATOM	524	HA	ILE	B	47	67.834	57.583	20.945	1.0	1.84	PROT	H
ATOM	525	HB	ILE	B	47	65.825	58.158	22.322	1.0	1.62	PROT	H
ATOM	526	HG12	ILE	B	47	64.692	59.779	20.821	1.0	1.51	PROT	H
ATOM	527	HG13	ILE	B	47	66.002	60.945	21.013	1.0	1.32	PROT	H
ATOM	528	HG21	ILE	B	47	67.007	59.361	23.925	1.0	1.36	PROT	H
ATOM	529	HG22	ILE	B	47	68.312	58.678	22.995	1.0	1.59	PROT	H
ATOM	530	HG23	ILE	B	47	67.823	60.431	22.822	1.0	1.57	PROT	H
ATOM	531	HD11	ILE	B	47	64.183	60.005	23.324	1.0	1.54	PROT	H
ATOM	532	HD12	ILE	B	47	65.404	61.287	23.417	1.0	1.43	PROT	H
ATOM	533	HD13	ILE	B	47	63.970	61.505	22.399	1.0	1.43	PROT	H
ATOM	534	N	GLY	B	48	65.864	56.528	19.802	1.0	-5.10	PROT	N
ATOM	535	CA	GLY	B	48	64.794	55.844	19.039	1.0	-1.65	PROT	C
ATOM	536	C	GLY	B	48	63.438	55.767	19.747	1.0	5.81	PROT	C
ATOM	537	O	GLY	B	48	63.317	56.086	20.939	1.0	-6.09	PROT	O
ATOM	538	H	GLY	B	48	66.237	55.978	20.581	1.0	3.37	PROT	H
ATOM	539	HA2	GLY	B	48	65.146	54.808	18.811	1.0	1.84	PROT	H
ATOM	540	HA3	GLY	B	48	64.670	56.364	18.052	1.0	1.99	PROT	H
ATOM	541	N	GLY	B	49	62.378	55.268	19.074	1.0	-5.46	PROT	N
ATOM	542	CA	GLY	B	49	61.032	54.966	19.538	1.0	-1.23	PROT	C
ATOM	543	C	GLY	B	49	60.189	54.243	18.448	1.0	5.55	PROT	C

ATOM	544	O	GLY	B	49	60.660	53.522	17.522	1.0	-6.66	PROT	O
ATOM	545	H	GLY	B	49	62.510	54.982	18.095	1.0	3.24	PROT	H
ATOM	546	HA2	GLY	B	49	60.516	55.901	19.885	1.0	2.00	PROT	H
ATOM	547	HA3	GLY	B	49	61.063	54.304	20.445	1.0	1.96	PROT	H
ATOM	548	N	ILE	B	50	58.859	54.267	18.620	1.0	-5.24	PROT	N
ATOM	549	CA	ILE	B	50	57.893	53.755	17.601	1.0	-0.50	PROT	C
ATOM	550	CB	ILE	B	50	56.516	53.244	18.280	1.0	-0.54	PROT	C
ATOM	551	CG2	ILE	B	50	55.779	52.443	17.191	1.0	-4.77	PROT	C
ATOM	552	CG1	ILE	B	50	56.704	52.333	19.565	1.0	-2.43	PROT	C
ATOM	553	CD1	ILE	B	50	57.881	51.353	19.451	1.0	-4.44	PROT	C
ATOM	554	C	ILE	B	50	57.756	54.652	16.400	1.0	5.82	PROT	C
ATOM	555	O	ILE	B	50	56.897	55.516	16.312	1.0	-6.53	PROT	O
ATOM	556	H	ILE	B	50	58.423	54.791	19.393	1.0	3.65	PROT	H
ATOM	557	HA	ILE	B	50	58.355	52.777	17.233	1.0	1.77	PROT	H
ATOM	558	HB	ILE	B	50	55.938	54.145	18.568	1.0	1.56	PROT	H
ATOM	559	HG12	ILE	B	50	55.764	51.784	19.734	1.0	1.36	PROT	H
ATOM	560	HG13	ILE	B	50	56.857	53.002	20.435	1.0	1.57	PROT	H
ATOM	561	HG21	ILE	B	50	54.779	52.149	17.533	1.0	1.57	PROT	H
ATOM	562	HG22	ILE	B	50	55.643	53.028	16.274	1.0	1.54	PROT	H
ATOM	563	HG23	ILE	B	50	56.311	51.523	16.928	1.0	1.52	PROT	H
ATOM	564	HD11	ILE	B	50	58.845	51.869	19.438	1.0	1.43	PROT	H
ATOM	565	HD12	ILE	B	50	57.890	50.660	20.302	1.0	1.48	PROT	H
ATOM	566	HD13	ILE	B	50	57.798	50.741	18.547	1.0	1.42	PROT	H
ATOM	567	N	NME	B	51	58.627	54.453	15.435	1.0	-6.08	PROT	N
ATOM	568	H1	NME	B	51	58.689	55.095	14.653	1.0	3.24	PROT	H
ATOM	569	H2	NME	B	51	59.456	53.882	15.572	1.0	3.32	PROT	H
ATOM	570	C	ACE	B	52	66.526	65.069	28.356	1.0	4.57	PROT	C
ATOM	571	O	ACE	B	52	67.505	64.355	28.053	1.0	-6.39	PROT	O
ATOM	572	HC	ACE	B	52	66.200	65.095	29.420	1.0	1.48	PROT	H
ATOM	573	N	LEU	B	76	65.845	65.761	27.512	1.0	-4.88	PROT	N
ATOM	574	CA	LEU	B	76	66.160	65.773	26.105	1.0	-0.32	PROT	C
ATOM	575	CB	LEU	B	76	66.172	64.334	25.600	1.0	-3.13	PROT	C
ATOM	576	CG	LEU	B	76	67.211	63.304	26.178	1.0	-0.18	PROT	C
ATOM	577	CD1	LEU	B	76	67.113	61.870	25.575	1.0	-4.82	PROT	C
ATOM	578	CD2	LEU	B	76	68.670	63.683	26.068	1.0	-4.30	PROT	C
ATOM	579	C	LEU	B	76	65.194	66.679	25.314	1.0	6.04	PROT	C
ATOM	580	O	LEU	B	76	64.008	66.563	25.396	1.0	-6.53	PROT	O
ATOM	581	H	LEU	B	76	64.902	66.102	27.747	1.0	3.34	PROT	H

ATOM	582	HA	LEU	B	76	67.216	66.180	25.997	1.0	1.69	PROT	H
ATOM	583	HB2	LEU	B	76	66.300	64.340	24.498	1.0	1.40	PROT	H
ATOM	584	HB3	LEU	B	76	65.166	63.890	25.786	1.0	1.68	PROT	H
ATOM	585	HG	LEU	B	76	66.873	63.034	27.221	1.0	1.80	PROT	H
ATOM	586	HD11	LEU	B	76	67.794	61.197	26.102	1.0	1.50	PROT	H
ATOM	587	HD12	LEU	B	76	66.096	61.477	25.656	1.0	1.37	PROT	H
ATOM	588	HD13	LEU	B	76	67.390	61.858	24.516	1.0	1.40	PROT	H
ATOM	589	HD21	LEU	B	76	69.063	63.512	25.059	1.0	1.24	PROT	H
ATOM	590	HD22	LEU	B	76	68.847	64.740	26.301	1.0	1.36	PROT	H
ATOM	591	HD23	LEU	B	76	69.282	63.108	26.774	1.0	1.44	PROT	H
ATOM	592	N	NME	B	77	65.760	67.584	24.546	1.0	-6.18	PROT	N
ATOM	593	H1	NME	B	77	65.209	68.225	23.986	1.0	3.25	PROT	H
ATOM	594	H2	NME	B	77	66.753	67.757	24.508	1.0	3.28	PROT	H
ATOM	595	C	ACE	B	78	61.424	64.875	19.253	1.0	4.49	PROT	C
ATOM	596	O	ACE	B	78	61.652	63.744	19.731	1.0	-6.36	PROT	O
ATOM	597	HC	ACE	B	78	62.258	65.408	18.748	1.0	1.42	PROT	H
ATOM	598	N	THR	B	80	60.283	65.466	19.303	1.0	-4.82	PROT	N
ATOM	599	CA	THR	B	80	59.140	64.861	19.929	1.0	-0.55	PROT	C
ATOM	600	CB	THR	B	80	59.268	64.567	21.427	1.0	1.65	PROT	C
ATOM	601	CG2	THR	B	80	59.712	65.829	22.186	1.0	-5.12	PROT	C
ATOM	602	OG1	THR	B	80	58.100	63.981	21.955	1.0	-6.13	PROT	O
ATOM	603	C	THR	B	80	57.859	65.772	19.866	1.0	5.81	PROT	C
ATOM	604	O	THR	B	80	57.967	67.019	19.866	1.0	-6.55	PROT	O
ATOM	605	H	THR	B	80	60.139	66.440	19.001	1.0	3.44	PROT	H
ATOM	606	HA	THR	B	80	58.922	63.882	19.400	1.0	1.91	PROT	H
ATOM	607	HB	THR	B	80	60.044	63.757	21.558	1.0	1.66	PROT	H
ATOM	608	HG21	THR	B	80	59.657	65.671	23.274	1.0	1.76	PROT	H
ATOM	609	HG22	THR	B	80	60.743	66.100	21.941	1.0	1.56	PROT	H
ATOM	610	HG23	THR	B	80	59.076	66.696	21.967	1.0	1.70	PROT	H
ATOM	611	HG1	THR	B	80	57.925	64.332	22.870	1.0	3.72	PROT	H
ATOM	612	N	PRO	B	81	56.625	65.225	19.716	1.0	-4.89	PROT	N
ATOM	613	CD	PRO	B	81	56.355	63.920	19.124	1.0	-0.72	PROT	C
ATOM	614	CG	PRO	B	81	54.857	63.895	18.754	1.0	-2.67	PROT	C
ATOM	615	CB	PRO	B	81	54.458	65.393	18.773	1.0	-2.74	PROT	C
ATOM	616	CA	PRO	B	81	55.326	66.010	19.890	1.0	-0.09	PROT	C
ATOM	617	C	PRO	B	81	54.744	66.054	21.230	1.0	5.80	PROT	C
ATOM	618	O	PRO	B	81	53.718	66.722	21.400	1.0	-6.41	PROT	O
ATOM	619	HA	PRO	B	81	55.570	67.089	19.613	1.0	2.01	PROT	H

ATOM	620	HB2	PRO	B	81	53.381	65.530	18.968	1.0	1.66	PROT	H
ATOM	621	HB3	PRO	B	81	54.668	65.874	17.804	1.0	1.57	PROT	H
ATOM	622	HG2	PRO	B	81	54.268	63.318	19.485	1.0	1.55	PROT	H
ATOM	623	HG3	PRO	B	81	54.686	63.436	17.769	1.0	1.60	PROT	H
ATOM	624	HD2	PRO	B	81	56.607	63.106	19.847	1.0	1.69	PROT	H
ATOM	625	HD3	PRO	B	81	56.986	63.754	18.221	1.0	1.54	PROT	H
ATOM	626	N	VAL	B	82	55.290	65.279	22.172	1.0	-5.21	PROT	N
ATOM	627	CA	VAL	B	82	54.923	65.500	23.613	1.0	-0.14	PROT	C
ATOM	628	CB	VAL	B	82	54.249	64.302	24.244	1.0	-0.90	PROT	C
ATOM	629	CG1	VAL	B	82	52.840	64.207	23.604	1.0	-4.47	PROT	C
ATOM	630	CG2	VAL	B	82	54.929	62.960	24.071	1.0	-4.53	PROT	C
ATOM	631	C	VAL	B	82	56.146	65.859	24.482	1.0	5.62	PROT	C
ATOM	632	O	VAL	B	82	57.283	65.425	24.248	1.0	-5.82	PROT	O
ATOM	633	H	VAL	B	82	56.147	64.744	22.013	1.0	3.53	PROT	H
ATOM	634	HA	VAL	B	82	54.203	66.378	23.633	1.0	1.94	PROT	H
ATOM	635	HB	VAL	B	82	54.124	64.498	25.345	1.0	1.57	PROT	H
ATOM	636	HG11	VAL	B	82	52.285	63.351	24.002	1.0	1.52	PROT	H
ATOM	637	HG12	VAL	B	82	52.243	65.103	23.803	1.0	1.43	PROT	H
ATOM	638	HG13	VAL	B	82	52.906	64.090	22.517	1.0	1.56	PROT	H
ATOM	639	HG21	VAL	B	82	55.971	62.981	24.424	1.0	1.59	PROT	H
ATOM	640	HG22	VAL	B	82	54.411	62.177	24.638	1.0	1.53	PROT	H
ATOM	641	HG23	VAL	B	82	54.954	62.641	23.019	1.0	1.62	PROT	H
ATOM	642	N	ASN	B	83	55.925	66.607	25.625	1.0	-5.60	PROT	N
ATOM	643	CA	ASN	B	83	56.720	66.586	26.834	1.0	0.52	PROT	C
ATOM	644	CB	ASN	B	83	56.443	67.768	27.722	1.0	-4.01	PROT	C
ATOM	645	CG	ASN	B	83	56.730	69.096	27.102	1.0	6.20	PROT	C
ATOM	646	OD1	ASN	B	83	57.750	69.244	26.434	1.0	-6.39	PROT	O
ATOM	647	ND2	ASN	B	83	55.793	70.037	27.140	1.0	-6.37	PROT	N
ATOM	648	C	ASN	B	83	56.395	65.313	27.630	1.0	5.61	PROT	C
ATOM	649	O	ASN	B	83	55.232	65.075	27.887	1.0	-6.32	PROT	O
ATOM	650	H	ASN	B	83	54.960	66.914	25.795	1.0	3.17	PROT	H
ATOM	651	HA	ASN	B	83	57.820	66.587	26.525	1.0	2.11	PROT	H
ATOM	652	HB2	ASN	B	83	57.072	67.681	28.649	1.0	2.02	PROT	H
ATOM	653	HB3	ASN	B	83	55.396	67.713	28.115	1.0	1.89	PROT	H
ATOM	654	HD21	ASN	B	83	54.959	69.972	27.700	1.0	3.23	PROT	H
ATOM	655	HD22	ASN	B	83	55.938	70.935	26.701	1.0	3.20	PROT	H
ATOM	656	N	ILE	B	84	57.387	64.413	27.853	1.0	-5.61	PROT	N
ATOM	657	CA	ILE	B	84	57.206	63.039	28.400	1.0	-0.35	PROT	C

ATOM	658	CB	ILE	B	84	56.998	61.933	27.307	1.0	-0.90	PROT	C
ATOM	659	CG2	ILE	B	84	57.988	62.155	26.146	1.0	-4.49	PROT	C
ATOM	660	CG1	ILE	B	84	57.017	60.508	27.826	1.0	-2.72	PROT	C
ATOM	661	CD1	ILE	B	84	56.473	59.392	26.872	1.0	-4.25	PROT	C
ATOM	662	C	ILE	B	84	58.275	62.743	29.469	1.0	5.88	PROT	C
ATOM	663	O	ILE	B	84	59.467	62.767	29.135	1.0	-6.40	PROT	O
ATOM	664	H	ILE	B	84	58.364	64.592	27.591	1.0	3.68	PROT	H
ATOM	665	HA	ILE	B	84	56.197	63.067	28.937	1.0	1.72	PROT	H
ATOM	666	HB	ILE	B	84	55.970	62.131	26.892	1.0	1.37	PROT	H
ATOM	667	HG12	ILE	B	84	56.438	60.453	28.769	1.0	1.27	PROT	H
ATOM	668	HG13	ILE	B	84	58.061	60.236	28.098	1.0	1.57	PROT	H
ATOM	669	HG21	ILE	B	84	59.022	62.158	26.515	1.0	1.59	PROT	H
ATOM	670	HG22	ILE	B	84	57.901	61.367	25.391	1.0	1.54	PROT	H
ATOM	671	HG23	ILE	B	84	57.821	63.117	25.648	1.0	1.48	PROT	H
ATOM	672	HD11	ILE	B	84	55.395	59.482	26.727	1.0	1.39	PROT	H
ATOM	673	HD12	ILE	B	84	56.953	59.449	25.892	1.0	1.46	PROT	H
ATOM	674	HD13	ILE	B	84	56.679	58.399	27.287	1.0	1.61	PROT	H
ATOM	675	N	NME	B	85	57.819	62.483	30.675	1.0	-6.16	PROT	N
ATOM	676	H1	NME	B	85	56.840	62.505	30.911	1.0	3.42	PROT	H
ATOM	677	H2	NME	B	85	58.454	62.328	31.447	1.0	3.22	PROT	H
HETATM	678	N1	DAR	C	100	50.449	51.567	22.108	1.0	-6.11	PROT	N
HETATM	679	C2	DAR	C	100	51.312	52.567	21.930	1.0	3.22	PROT	C
HETATM	680	C3	DAR	C	100	52.661	52.264	22.066	1.0	-3.66	PROT	C
HETATM	681	C4	DAR	C	100	53.638	53.237	21.876	1.0	0.24	PROT	C
HETATM	682	C5	DAR	C	100	53.211	54.578	21.456	1.0	-5.24	PROT	C
HETATM	683	C6	DAR	C	100	51.802	54.925	21.329	1.0	0.62	PROT	C
HETATM	684	C7	DAR	C	100	50.854	53.891	21.586	1.0	-3.51	PROT	C
HETATM	685	S8	DAR	C	100	54.472	55.797	21.085	1.0	23.78	PROT	S
HETATM	686	O9	DAR	C	100	55.791	55.219	21.107	1.0	-9.72	PROT	O
HETATM	687	O10	DAR	C	100	53.974	56.528	19.938	1.0	-9.14	PROT	O
HETATM	688	N11	DAR	C	100	54.489	56.899	22.402	1.0	-7.66	PROT	N
HETATM	689	C12	DAR	C	100	53.848	58.217	22.202	1.0	-0.77	PROT	C
HETATM	690	C13	DAR	C	100	54.722	59.317	21.489	1.0	-0.71	PROT	C
HETATM	691	C14	DAR	C	100	53.928	60.588	21.045	1.0	-4.62	PROT	C
HETATM	692	C15	DAR	C	100	55.950	59.779	22.335	1.0	-4.56	PROT	C
HETATM	693	C16	DAR	C	100	54.305	56.336	23.744	1.0	-1.09	PROT	C
HETATM	694	C17	DAR	C	100	55.491	56.711	24.649	1.0	1.51	PROT	C
HETATM	695	O18	DAR	C	100	55.002	56.571	26.002	1.0	-6.74	PROT	O

HETATM	696	C19	DAR	C	100	56.771	55.810	24.494	1.0	0.39	PROT	C
HETATM	697	N20	DAR	C	100	57.853	56.340	25.310	1.0	-5.70	PROT	N
HETATM	698	C21	DAR	C	100	58.894	56.938	24.723	1.0	7.41	PROT	C
HETATM	699	O22	DAR	C	100	59.000	57.224	23.542	1.0	-6.50	PROT	O
HETATM	700	O23	DAR	C	100	59.965	57.020	25.524	1.0	-4.37	PROT	O
HETATM	701	C24	DAR	C	100	61.188	57.503	25.073	1.0	1.26	PROT	C
HETATM	702	C25	DAR	C	100	61.821	58.284	26.263	1.0	-0.29	PROT	C
HETATM	703	O26	DAR	C	100	62.503	57.193	26.918	1.0	-4.66	PROT	O
HETATM	704	C27	DAR	C	100	63.140	56.320	25.982	1.0	3.32	PROT	C
HETATM	705	O28	DAR	C	100	63.088	54.965	26.445	1.0	-4.71	PROT	O
HETATM	706	C29	DAR	C	100	62.785	54.050	25.377	1.0	0.06	PROT	C
HETATM	707	C30	DAR	C	100	61.768	54.880	24.640	1.0	-3.21	PROT	C
HETATM	708	C31	DAR	C	100	62.215	56.332	24.762	1.0	-2.26	PROT	C
HETATM	709	C32	DAR	C	100	56.468	54.357	24.798	1.0	-3.24	PROT	C
HETATM	710	C33	DAR	C	100	58.489	53.228	25.795	1.0	-1.59	PROT	C
HETATM	711	C34	DAR	C	100	59.633	52.427	25.678	1.0	-1.42	PROT	C
HETATM	712	C35	DAR	C	100	59.988	51.787	24.477	1.0	-1.79	PROT	C
HETATM	713	C36	DAR	C	100	59.226	52.141	23.373	1.0	-1.72	PROT	C
HETATM	714	C37	DAR	C	100	58.134	52.988	23.434	1.0	-1.77	PROT	C
HETATM	715	C38	DAR	C	100	57.702	53.468	24.669	1.0	0.01	PROT	C
HETATM	716	H11	DAR	C	100	49.517	51.617	21.730	1.0	3.06	PROT	H
HETATM	717	H12	DAR	C	100	50.709	50.653	22.450	1.0	3.34	PROT	H
HETATM	718	H3	DAR	C	100	52.974	51.257	22.339	1.0	1.63	PROT	H
HETATM	719	H4	DAR	C	100	54.694	53.014	21.989	1.0	1.53	PROT	H
HETATM	720	H6	DAR	C	100	51.485	55.923	21.055	1.0	1.65	PROT	H
HETATM	721	H7	DAR	C	100	49.796	54.106	21.507	1.0	1.81	PROT	H
HETATM	722	H121	DAR	C	100	53.547	58.629	23.194	1.0	1.48	PROT	H
HETATM	723	H122	DAR	C	100	52.900	58.120	21.622	1.0	1.42	PROT	H
HETATM	724	H13	DAR	C	100	55.121	58.846	20.557	1.0	1.42	PROT	H
HETATM	725	H141	DAR	C	100	53.118	60.319	20.362	1.0	1.43	PROT	H
HETATM	726	H142	DAR	C	100	54.594	61.282	20.527	1.0	1.41	PROT	H
HETATM	727	H143	DAR	C	100	53.493	61.103	21.905	1.0	1.42	PROT	H
HETATM	728	H151	DAR	C	100	56.567	58.914	22.608	1.0	1.60	PROT	H
HETATM	729	H152	DAR	C	100	55.636	60.278	23.253	1.0	1.36	PROT	H
HETATM	730	H153	DAR	C	100	56.578	60.467	21.763	1.0	1.43	PROT	H
HETATM	731	H161	DAR	C	100	54.170	55.232	23.731	1.0	1.41	PROT	H
HETATM	732	H162	DAR	C	100	53.371	56.725	24.234	1.0	1.73	PROT	H
HETATM	733	H17	DAR	C	100	55.764	57.782	24.530	1.0	1.39	PROT	H

```
HETATM  734  H18 DAR C 100      55.721  56.322  26.642  1.0   3.83      PROT H
HETATM  735  H19 DAR C 100      57.065  55.885  23.397  1.0   1.80      PROT H
HETATM  736  H20 DAR C 100      57.862  56.050  26.300  1.0   3.76      PROT H
HETATM  737  H24 DAR C 100      61.046  58.130  24.167  1.0   1.44      PROT H
HETATM  738 H251 DAR C 100      61.110  58.652  27.017  1.0   1.78      PROT H
HETATM  739 H252 DAR C 100      62.538  59.051  25.959  1.0   1.26      PROT H
HETATM  740  H27 DAR C 100      64.197  56.611  25.905  1.0   1.35      PROT H
HETATM  741 H291 DAR C 100      62.395  53.157  25.894  1.0   1.62      PROT H
HETATM  742 H292 DAR C 100      63.704  53.813  24.827  1.0   1.07      PROT H
HETATM  743 H301 DAR C 100      60.756  54.750  25.095  1.0   1.89      PROT H
HETATM  744 H302 DAR C 100      61.636  54.580  23.585  1.0   1.46      PROT H
HETATM  745  H31 DAR C 100      62.748  56.630  23.830  1.0   1.52      PROT H
HETATM  746 H321 DAR C 100      55.669  53.980  24.134  1.0   1.45      PROT H
HETATM  747 H322 DAR C 100      56.076  54.255  25.845  1.0   1.96      PROT H
HETATM  748  H33 DAR C 100      58.201  53.643  26.768  1.0   1.97      PROT H
HETATM  749  H34 DAR C 100      60.245  52.266  26.572  1.0   1.74      PROT H
HETATM  750  H35 DAR C 100      60.827  51.110  24.419  1.0   1.46      PROT H
HETATM  751  H36 DAR C 100      59.507  51.741  22.393  1.0   1.37      PROT H
HETATM  752  H37 DAR C 100      57.610  53.276  22.521  1.0   1.64      PROT H
HETATM  753  OW  SOL D5793      65.197  55.861  23.054  1.0  -7.32      PROT O
HETATM  754  HW1 SOL D5793      64.775  55.198  23.594  1.0   3.48      PROT H
HETATM  755  HW2 SOL D5793      64.574  56.033  22.333  1.0   3.69      PROT H
HETATM  756  OW  SOL D8297      47.967  55.770  20.033  1.0  -7.66      PROT O
HETATM  757  HW1 SOL D8297      48.852  55.861  19.702  1.0   3.41      PROT H
HETATM  758  HW2 SOL D8297      48.047  55.895  21.004  1.0   3.89      PROT H
HETATM  759  OW  SOL D8556      50.661  57.637  23.494  1.0  -7.59      PROT O
HETATM  760  HW1 SOL D8556      50.782  57.412  24.424  1.0   3.73      PROT H
HETATM  761  HW2 SOL D8556      49.819  57.232  23.256  1.0   3.64      PROT H
HETATM  762  OW  SOL D 401      58.398  55.471  21.193  1.0  -7.94      PROT O
HETATM  763  HW1 SOL D 401      57.431  55.439  21.203  1.0   3.94      PROT H
HETATM  764  HW2 SOL D 401      58.644  56.069  21.905  1.0   3.80      PROT H
END
```

4.3 IND-HIV^{Pro}

```
HEADER  data-set: HIV_IND_full_wH2O_OPT
REMARK  MOPAC, Version: 23.1.2
REMARK  99
```

```
REMARK  99 MOE v2014.09 (Chemical Computing Group Inc.)
ATOM      1  C   ACE A   7      41.663  57.891  50.840  1.0   4.60      PROT C
ATOM      2  O   ACE A   7      42.151  58.571  49.914  1.0  -6.33      PROT O
ATOM      3  HC  ACE A   7      41.565  58.367  51.841  1.0   1.54      PROT H
ATOM      4  N   ARG A   8      41.250  56.680  50.710  1.0  -5.10      PROT N
ATOM      5  CA  ARG A   8      41.320  55.950  49.390  1.0  -0.16      PROT C
ATOM      6  CB  ARG A   8      42.710  55.890  48.780  1.0  -2.80      PROT C
ATOM      7  CG  ARG A   8      43.020  57.190  47.990  1.0  -3.29      PROT C
ATOM      8  CD  ARG A   8      44.420  57.120  47.530  1.0  -0.59      PROT C
ATOM      9  NE  ARG A   8      44.730  58.240  46.630  1.0  -5.62      PROT N
ATOM     10  CZ  ARG A   8      45.880  58.380  45.950  1.0   6.23      PROT C
ATOM     11  NH1 ARG A   8      46.770  57.480  45.960  1.0  -6.05      PROT N
ATOM     12  NH2 ARG A   8      46.080  59.420  45.200  1.0  -6.20      PROT N
ATOM     13  C   ARG A   8      40.770  54.520  49.480  1.0   5.63      PROT C
ATOM     14  O   ARG A   8      41.400  53.830  50.260  1.0  -6.33      PROT O
ATOM     15  H   ARG A   8      40.953  56.096  51.492  1.0   3.34      PROT H
ATOM     16  HA  ARG A   8      40.665  56.575  48.700  1.0   1.82      PROT H
ATOM     17  HB2 ARG A   8      42.806  55.018  48.104  1.0   1.49      PROT H
ATOM     18  HB3 ARG A   8      43.468  55.731  49.573  1.0   1.61      PROT H
ATOM     19  HG2 ARG A   8      42.852  58.148  48.529  1.0   2.47      PROT H
ATOM     20  HG3 ARG A   8      42.304  57.272  47.140  1.0   1.61      PROT H
ATOM     21  HD2 ARG A   8      44.619  56.158  46.986  1.0   1.39      PROT H
ATOM     22  HD3 ARG A   8      45.127  57.135  48.402  1.0   1.42      PROT H
ATOM     23  HE  ARG A   8      44.067  59.076  46.696  1.0   4.08      PROT H
ATOM     24 HH11 ARG A   8      46.739  56.648  46.548  1.0   3.41      PROT H
ATOM     25 HH12 ARG A   8      47.642  57.534  45.435  1.0   3.39      PROT H
ATOM     26 HH21 ARG A   8      45.324  60.158  45.112  1.0   3.99      PROT H
ATOM     27 HH22 ARG A   8      46.858  59.529  44.566  1.0   3.43      PROT H
ATOM     28  N   NME A   9      39.728  54.260  48.721  1.0  -6.06      PROT N
ATOM     29  H1  NME A   9      39.305  54.940  48.102  1.0   3.40      PROT H
ATOM     30  H2  NME A   9      39.305  53.342  48.677  1.0   3.23      PROT H
ATOM     31  C   ACE A  10      44.369  50.005  44.450  1.0   4.90      PROT C
ATOM     32  O   ACE A  10      44.589  51.005  45.165  1.0  -6.90      PROT O
ATOM     33  HC  ACE A  10      45.213  49.306  44.255  1.0   1.54      PROT H
ATOM     34  N   LEU A  23      43.230  49.730  43.920  1.0  -4.75      PROT N
ATOM     35  CA  LEU A  23      42.070  50.580  44.110  1.0  -0.40      PROT C
ATOM     36  CB  LEU A  23      42.500  52.010  43.730  1.0  -3.32      PROT C
ATOM     37  CG  LEU A  23      41.580  53.120  44.240  1.0  -0.69      PROT C
```

ATOM	38	CD1	LEU	A	23	42.260	54.470	44.040	1.0	-4.47	PROT	C
ATOM	39	CD2	LEU	A	23	40.250	53.240	43.470	1.0	-4.72	PROT	C
ATOM	40	C	LEU	A	23	40.790	50.110	43.410	1.0	6.01	PROT	C
ATOM	41	O	LEU	A	23	40.830	49.630	42.300	1.0	-6.41	PROT	O
ATOM	42	H	LEU	A	23	43.147	49.057	43.145	1.0	3.38	PROT	H
ATOM	43	HA	LEU	A	23	41.863	50.590	45.233	1.0	1.81	PROT	H
ATOM	44	HB2	LEU	A	23	42.612	52.073	42.628	1.0	1.59	PROT	H
ATOM	45	HB3	LEU	A	23	43.522	52.237	44.117	1.0	2.08	PROT	H
ATOM	46	HG	LEU	A	23	41.369	52.956	45.322	1.0	1.35	PROT	H
ATOM	47	HD11	LEU	A	23	42.464	54.672	42.982	1.0	1.43	PROT	H
ATOM	48	HD12	LEU	A	23	41.650	55.294	44.436	1.0	1.64	PROT	H
ATOM	49	HD13	LEU	A	23	43.224	54.519	44.565	1.0	1.48	PROT	H
ATOM	50	HD21	LEU	A	23	39.734	54.178	43.731	1.0	1.69	PROT	H
ATOM	51	HD22	LEU	A	23	40.411	53.224	42.390	1.0	1.38	PROT	H
ATOM	52	HD23	LEU	A	23	39.540	52.453	43.719	1.0	1.34	PROT	H
ATOM	53	N	LEU	A	24	39.650	50.130	44.140	1.0	-5.53	PROT	N
ATOM	54	CA	LEU	A	24	38.370	49.720	43.570	1.0	0.19	PROT	C
ATOM	55	CB	LEU	A	24	37.480	49.270	44.760	1.0	-3.19	PROT	C
ATOM	56	CG	LEU	A	24	38.030	48.140	45.690	1.0	-0.44	PROT	C
ATOM	57	CD1	LEU	A	24	37.340	48.210	47.050	1.0	-4.61	PROT	C
ATOM	58	CD2	LEU	A	24	37.680	46.910	44.970	1.0	-4.72	PROT	C
ATOM	59	C	LEU	A	24	37.660	50.870	42.750	1.0	5.64	PROT	C
ATOM	60	O	LEU	A	24	36.890	51.690	43.260	1.0	-6.40	PROT	O
ATOM	61	H	LEU	A	24	39.614	50.538	45.075	1.0	3.43	PROT	H
ATOM	62	HA	LEU	A	24	38.535	48.849	42.874	1.0	1.83	PROT	H
ATOM	63	HB2	LEU	A	24	36.489	48.974	44.361	1.0	1.68	PROT	H
ATOM	64	HB3	LEU	A	24	37.257	50.168	45.378	1.0	1.64	PROT	H
ATOM	65	HG	LEU	A	24	39.131	48.253	45.826	1.0	1.40	PROT	H
ATOM	66	HD11	LEU	A	24	37.586	49.136	47.583	1.0	1.49	PROT	H
ATOM	67	HD12	LEU	A	24	36.248	48.165	46.958	1.0	1.50	PROT	H
ATOM	68	HD13	LEU	A	24	37.645	47.374	47.690	1.0	1.49	PROT	H
ATOM	69	HD21	LEU	A	24	38.052	46.001	45.474	1.0	1.47	PROT	H
ATOM	70	HD22	LEU	A	24	36.593	46.760	44.866	1.0	1.55	PROT	H
ATOM	71	HD23	LEU	A	24	38.099	46.869	43.952	1.0	1.50	PROT	H
ATOM	72	N	ASH	A	25	38.090	51.060	41.520	1.0	-5.31	PROT	N
ATOM	73	CA	ASH	A	25	37.880	52.260	40.670	1.0	0.21	PROT	C
ATOM	74	CB	ASH	A	25	39.090	52.350	39.720	1.0	-3.33	PROT	C
ATOM	75	CG	ASH	A	25	39.130	53.830	39.170	1.0	6.31	PROT	C

ATOM	76	OD1	ASH	A	25	38.270	54.720	39.410	1.0	-5.63	PROT	O
ATOM	77	OD2	ASH	A	25	40.240	54.280	38.490	1.0	-5.34	PROT	O
ATOM	78	C	ASH	A	25	36.630	52.200	39.730	1.0	5.72	PROT	C
ATOM	79	O	ASH	A	25	36.580	51.610	38.700	1.0	-5.47	PROT	O
ATOM	80	H	ASH	A	25	38.755	50.385	41.113	1.0	3.38	PROT	H
ATOM	81	HA	ASH	A	25	37.804	53.161	41.344	1.0	1.96	PROT	H
ATOM	82	HB2	ASH	A	25	39.011	51.643	38.865	1.0	2.17	PROT	H
ATOM	83	HB3	ASH	A	25	40.053	52.142	40.224	1.0	1.90	PROT	H
ATOM	84	HD2	ASH	A	25	40.179	55.283	38.370	1.0	3.84	PROT	H
ATOM	85	N	THR	A	26	35.620	53.030	40.090	1.0	-5.90	PROT	N
ATOM	86	CA	THR	A	26	34.340	53.110	39.330	1.0	-0.07	PROT	C
ATOM	87	CB	THR	A	26	33.220	53.720	40.180	1.0	1.67	PROT	C
ATOM	88	CG2	THR	A	26	32.790	52.850	41.310	1.0	-4.92	PROT	C
ATOM	89	OG1	THR	A	26	33.450	54.990	40.730	1.0	-6.22	PROT	O
ATOM	90	C	THR	A	26	34.520	54.060	38.040	1.0	5.36	PROT	C
ATOM	91	O	THR	A	26	33.810	53.840	37.050	1.0	-6.13	PROT	O
ATOM	92	H	THR	A	26	35.602	53.526	40.985	1.0	3.52	PROT	H
ATOM	93	HA	THR	A	26	34.053	52.100	38.940	1.0	2.02	PROT	H
ATOM	94	HB	THR	A	26	32.347	53.839	39.462	1.0	1.39	PROT	H
ATOM	95	HG21	THR	A	26	31.904	53.263	41.817	1.0	1.66	PROT	H
ATOM	96	HG22	THR	A	26	32.543	51.828	40.995	1.0	1.64	PROT	H
ATOM	97	HG23	THR	A	26	33.566	52.776	42.091	1.0	1.72	PROT	H
ATOM	98	HG1	THR	A	26	32.927	55.717	40.349	1.0	3.68	PROT	H
ATOM	99	N	GLY	A	27	35.510	54.920	38.050	1.0	-5.15	PROT	N
ATOM	100	CA	GLY	A	27	35.850	55.830	36.990	1.0	-1.49	PROT	C
ATOM	101	C	GLY	A	27	36.650	55.160	35.870	1.0	5.79	PROT	C
ATOM	102	O	GLY	A	27	37.400	55.920	35.210	1.0	-6.62	PROT	O
ATOM	103	H	GLY	A	27	36.148	54.969	38.863	1.0	3.53	PROT	H
ATOM	104	HA2	GLY	A	27	36.462	56.677	37.395	1.0	1.87	PROT	H
ATOM	105	HA3	GLY	A	27	34.931	56.285	36.531	1.0	1.88	PROT	H
ATOM	106	N	ALA	A	28	36.660	53.820	35.770	1.0	-5.25	PROT	N
ATOM	107	CA	ALA	A	28	37.310	52.990	34.720	1.0	0.26	PROT	C
ATOM	108	CB	ALA	A	28	38.670	52.430	35.070	1.0	-4.67	PROT	C
ATOM	109	C	ALA	A	28	36.400	51.840	34.230	1.0	5.73	PROT	C
ATOM	110	O	ALA	A	28	35.780	51.170	34.990	1.0	-5.50	PROT	O
ATOM	111	H	ALA	A	28	36.098	53.250	36.411	1.0	3.41	PROT	H
ATOM	112	HA	ALA	A	28	37.466	53.707	33.845	1.0	1.77	PROT	H
ATOM	113	HB1	ALA	A	28	39.380	53.227	35.329	1.0	1.63	PROT	H

ATOM	114	HB2	ALA	A	28	38.628	51.748	35.935	1.0	1.76	PROT	H
ATOM	115	HB3	ALA	A	28	39.106	51.855	34.240	1.0	1.72	PROT	H
ATOM	116	N	ASP	A	29	36.370	51.590	32.900	1.0	-6.04	PROT	N
ATOM	117	CA	ASP	A	29	35.570	50.580	32.230	1.0	0.42	PROT	C
ATOM	118	CB	ASP	A	29	35.430	50.770	30.730	1.0	-4.17	PROT	C
ATOM	119	CG	ASP	A	29	34.770	52.080	30.280	1.0	7.59	PROT	C
ATOM	120	OD1	ASP	A	29	33.950	52.730	30.980	1.0	-7.71	PROT	O
ATOM	121	OD2	ASP	A	29	35.060	52.530	29.170	1.0	-8.38	PROT	O
ATOM	122	C	ASP	A	29	36.160	49.210	32.570	1.0	5.78	PROT	C
ATOM	123	O	ASP	A	29	35.350	48.300	32.850	1.0	-6.13	PROT	O
ATOM	124	H	ASP	A	29	36.811	52.259	32.258	1.0	3.30	PROT	H
ATOM	125	HA	ASP	A	29	34.518	50.602	32.682	1.0	2.12	PROT	H
ATOM	126	HB2	ASP	A	29	34.812	49.942	30.308	1.0	2.02	PROT	H
ATOM	127	HB3	ASP	A	29	36.412	50.694	30.215	1.0	1.82	PROT	H
ATOM	128	N	ASH	A	30	37.490	49.080	32.610	1.0	-5.45	PROT	N
ATOM	129	CA	ASH	A	30	38.360	47.890	32.470	1.0	0.20	PROT	C
ATOM	130	CB	ASH	A	30	38.620	47.590	30.980	1.0	-3.63	PROT	C
ATOM	131	CG	ASH	A	30	38.970	46.110	30.700	1.0	6.10	PROT	C
ATOM	132	OD1	ASH	A	30	40.120	45.660	30.500	1.0	-5.12	PROT	O
ATOM	133	OD2	ASH	A	30	37.920	45.340	30.580	1.0	-5.50	PROT	O
ATOM	134	C	ASH	A	30	39.640	47.900	33.380	1.0	5.16	PROT	C
ATOM	135	O	ASH	A	30	40.240	48.980	33.750	1.0	-5.61	PROT	O
ATOM	136	H	ASH	A	30	38.053	49.939	32.527	1.0	3.24	PROT	H
ATOM	137	HA	ASH	A	30	37.717	47.027	32.861	1.0	1.90	PROT	H
ATOM	138	HB2	ASH	A	30	39.457	48.212	30.590	1.0	1.97	PROT	H
ATOM	139	HB3	ASH	A	30	37.727	47.859	30.373	1.0	2.03	PROT	H
ATOM	140	HD2	ASH	A	30	38.116	44.386	30.338	1.0	3.99	PROT	H
ATOM	141	N	THR	A	31	39.920	46.710	33.850	1.0	-4.97	PROT	N
ATOM	142	CA	THR	A	31	40.950	46.630	34.900	1.0	-0.94	PROT	C
ATOM	143	CB	THR	A	31	40.710	45.390	35.730	1.0	1.78	PROT	C
ATOM	144	CG2	THR	A	31	41.890	44.910	36.600	1.0	-4.81	PROT	C
ATOM	145	OG1	THR	A	31	39.570	45.550	36.510	1.0	-6.07	PROT	O
ATOM	146	C	THR	A	31	42.380	46.770	34.360	1.0	5.67	PROT	C
ATOM	147	O	THR	A	31	42.710	46.090	33.330	1.0	-6.36	PROT	O
ATOM	148	H	THR	A	31	39.656	45.834	33.400	1.0	3.44	PROT	H
ATOM	149	HA	THR	A	31	40.771	47.532	35.592	1.0	2.10	PROT	H
ATOM	150	HB	THR	A	31	40.458	44.525	35.040	1.0	1.35	PROT	H
ATOM	151	HG21	THR	A	31	41.576	44.084	37.247	1.0	1.62	PROT	H

```
ATOM    152 HG22 THR A  31      42.721  44.568  35.974  1.0   1.66      PROT H
ATOM    153 HG23 THR A  31      42.258  45.713  37.255  1.0   1.75      PROT H
ATOM    154  HG1 THR A  31      38.914  46.149  36.093  1.0   3.53      PROT H
ATOM    155  N   VAL A  32      43.190  47.540  35.050  1.0  -5.17      PROT N
ATOM    156  CA  VAL A  32      44.570  47.920  34.750  1.0  -0.34      PROT C
ATOM    157  CB  VAL A  32      44.570  49.360  34.290  1.0  -0.71      PROT C
ATOM    158  CG1 VAL A  32      45.920  50.080  34.660  1.0  -4.57      PROT C
ATOM    159  CG2 VAL A  32      44.280  49.580  32.810  1.0  -4.53      PROT C
ATOM    160  C   VAL A  32      45.530  47.670  35.930  1.0   5.89      PROT C
ATOM    161  O   VAL A  32      45.230  47.930  37.110  1.0  -6.57      PROT O
ATOM    162  H   VAL A  32      42.878  47.946  35.957  1.0   3.58      PROT H
ATOM    163  HA  VAL A  32      44.910  47.268  33.873  1.0   2.12      PROT H
ATOM    164  HB  VAL A  32      43.771  49.908  34.869  1.0   1.46      PROT H
ATOM    165 HG11 VAL A  32      46.074  50.106  35.743  1.0   1.52      PROT H
ATOM    166 HG12 VAL A  32      46.771  49.581  34.189  1.0   1.54      PROT H
ATOM    167 HG13 VAL A  32      45.909  51.113  34.304  1.0   1.44      PROT H
ATOM    168 HG21 VAL A  32      43.345  49.084  32.512  1.0   1.59      PROT H
ATOM    169 HG22 VAL A  32      44.176  50.644  32.578  1.0   1.39      PROT H
ATOM    170 HG23 VAL A  32      45.082  49.169  32.181  1.0   1.60      PROT H
ATOM    171  N   NME A  33      46.701  47.159  35.616  1.0  -6.14      PROT N
ATOM    172  H1  NME A  33      46.997  47.009  34.656  1.0   3.40      PROT H
ATOM    173  H2  NME A  33      47.407  47.009  36.325  1.0   3.23      PROT H
ATOM    174  C   ACE A  34      45.955  52.329  25.621  1.0   4.28      PROT C
ATOM    175  O   ACE A  34      44.846  51.824  25.896  1.0  -6.24      PROT O
ATOM    176  HC  ACE A  34      46.587  51.845  24.843  1.0   1.44      PROT H
ATOM    177  N   ILE A  47      46.440  53.370  26.200  1.0  -4.98      PROT N
ATOM    178  CA  ILE A  47      45.690  54.100  27.300  1.0  -0.66      PROT C
ATOM    179  CB  ILE A  47      45.140  53.100  28.310  1.0  -0.91      PROT C
ATOM    180  CG2 ILE A  47      44.360  51.950  27.730  1.0  -3.72      PROT C
ATOM    181  CG1 ILE A  47      46.280  52.590  29.160  1.0  -2.74      PROT C
ATOM    182  CD1 ILE A  47      45.890  51.490  30.160  1.0  -4.36      PROT C
ATOM    183  C   ILE A  47      46.340  55.300  27.910  1.0   5.71      PROT C
ATOM    184  O   ILE A  47      47.530  55.470  27.920  1.0  -6.36      PROT O
ATOM    185  H   ILE A  47      47.380  53.734  26.045  1.0   3.37      PROT H
ATOM    186  HA  ILE A  47      44.769  54.493  26.733  1.0   1.81      PROT H
ATOM    187  HB  ILE A  47      44.430  53.669  28.981  1.0   1.32      PROT H
ATOM    188 HG12 ILE A  47      46.748  53.428  29.719  1.0   1.35      PROT H
ATOM    189 HG13 ILE A  47      47.092  52.198  28.510  1.0   1.45      PROT H
```

ATOM	190	HG21	ILE	A	47	43.548	52.260	27.047	1.0	1.59	PROT	H
ATOM	191	HG22	ILE	A	47	44.941	51.037	27.510	1.0	1.67	PROT	H
ATOM	192	HG23	ILE	A	47	43.727	51.538	28.564	1.0	1.07	PROT	H
ATOM	193	HD11	ILE	A	47	46.717	51.278	30.848	1.0	1.46	PROT	H
ATOM	194	HD12	ILE	A	47	45.024	51.785	30.762	1.0	1.40	PROT	H
ATOM	195	HD13	ILE	A	47	45.642	50.550	29.655	1.0	1.51	PROT	H
ATOM	196	N	GLY	A	48	45.570	56.030	28.630	1.0	-4.93	PROT	N
ATOM	197	CA	GLY	A	48	46.070	57.110	29.440	1.0	-1.88	PROT	C
ATOM	198	C	GLY	A	48	45.050	57.640	30.420	1.0	5.56	PROT	C
ATOM	199	O	GLY	A	48	43.800	57.520	30.210	1.0	-5.85	PROT	O
ATOM	200	H	GLY	A	48	44.538	55.965	28.637	1.0	3.44	PROT	H
ATOM	201	HA2	GLY	A	48	46.403	57.971	28.790	1.0	2.00	PROT	H
ATOM	202	HA3	GLY	A	48	47.019	56.796	29.963	1.0	2.01	PROT	H
ATOM	203	N	GLY	A	49	45.560	58.320	31.500	1.0	-5.58	PROT	N
ATOM	204	CA	GLY	A	49	44.680	59.070	32.330	1.0	-1.78	PROT	C
ATOM	205	C	GLY	A	49	44.100	60.230	31.640	1.0	5.93	PROT	C
ATOM	206	O	GLY	A	49	42.880	60.300	31.510	1.0	-6.43	PROT	O
ATOM	207	H	GLY	A	49	46.577	58.426	31.627	1.0	3.62	PROT	H
ATOM	208	HA2	GLY	A	49	45.188	59.359	33.293	1.0	1.90	PROT	H
ATOM	209	HA3	GLY	A	49	43.831	58.405	32.689	1.0	2.04	PROT	H
ATOM	210	N	ILE	A	50	44.940	61.150	31.210	1.0	-5.26	PROT	N
ATOM	211	CA	ILE	A	50	44.590	62.300	30.360	1.0	-0.13	PROT	C
ATOM	212	CB	ILE	A	50	44.020	63.400	31.260	1.0	-1.15	PROT	C
ATOM	213	CG2	ILE	A	50	42.510	63.470	31.280	1.0	-4.56	PROT	C
ATOM	214	CG1	ILE	A	50	44.540	63.490	32.700	1.0	-2.51	PROT	C
ATOM	215	CD1	ILE	A	50	46.000	63.840	32.870	1.0	-4.57	PROT	C
ATOM	216	C	ILE	A	50	45.850	62.810	29.590	1.0	5.53	PROT	C
ATOM	217	O	ILE	A	50	46.950	62.820	30.140	1.0	-6.08	PROT	O
ATOM	218	H	ILE	A	50	45.941	61.098	31.422	1.0	3.31	PROT	H
ATOM	219	HA	ILE	A	50	43.806	61.964	29.625	1.0	1.72	PROT	H
ATOM	220	HB	ILE	A	50	44.358	64.376	30.778	1.0	1.46	PROT	H
ATOM	221	HG12	ILE	A	50	44.320	62.542	33.236	1.0	1.45	PROT	H
ATOM	222	HG13	ILE	A	50	43.934	64.255	33.239	1.0	1.47	PROT	H
ATOM	223	HG21	ILE	A	50	42.094	63.574	30.272	1.0	1.28	PROT	H
ATOM	224	HG22	ILE	A	50	42.071	62.557	31.717	1.0	1.69	PROT	H
ATOM	225	HG23	ILE	A	50	42.152	64.325	31.870	1.0	1.56	PROT	H
ATOM	226	HD11	ILE	A	50	46.666	63.107	32.399	1.0	1.52	PROT	H
ATOM	227	HD12	ILE	A	50	46.246	64.814	32.431	1.0	1.43	PROT	H

ATOM	228	HD13	ILE	A	50	46.271	63.888	33.938	1.0	1.71	PROT	H
ATOM	229	N	NME	A	51	45.641	63.211	28.355	1.0	-6.11	PROT	N
ATOM	230	H1	NME	A	51	44.738	63.214	27.907	1.0	3.27	PROT	H
ATOM	231	H2	NME	A	51	46.393	63.581	27.784	1.0	3.31	PROT	H
ATOM	232	C	ACE	A	52	43.367	44.449	31.142	1.0	4.81	PROT	C
ATOM	233	O	ACE	A	52	42.987	44.726	29.986	1.0	-6.48	PROT	O
ATOM	234	HC	ACE	A	52	42.697	43.831	31.779	1.0	1.49	PROT	H
ATOM	235	N	LEU	A	76	44.470	44.860	31.660	1.0	-4.96	PROT	N
ATOM	236	CA	LEU	A	76	45.390	45.700	30.930	1.0	-0.41	PROT	C
ATOM	237	CB	LEU	A	76	44.480	46.920	30.510	1.0	-2.71	PROT	C
ATOM	238	CG	LEU	A	76	43.640	46.810	29.230	1.0	-1.09	PROT	C
ATOM	239	CD1	LEU	A	76	42.430	47.730	29.120	1.0	-4.45	PROT	C
ATOM	240	CD2	LEU	A	76	44.440	46.930	27.950	1.0	-4.62	PROT	C
ATOM	241	C	LEU	A	76	46.650	46.080	31.730	1.0	5.71	PROT	C
ATOM	242	O	LEU	A	76	46.580	46.920	32.650	1.0	-6.44	PROT	O
ATOM	243	H	LEU	A	76	44.563	44.906	32.687	1.0	3.37	PROT	H
ATOM	244	HA	LEU	A	76	45.694	45.172	29.980	1.0	1.68	PROT	H
ATOM	245	HB2	LEU	A	76	45.126	47.817	30.427	1.0	1.35	PROT	H
ATOM	246	HB3	LEU	A	76	43.789	47.137	31.358	1.0	1.78	PROT	H
ATOM	247	HG	LEU	A	76	43.172	45.807	29.029	1.0	2.22	PROT	H
ATOM	248	HD11	LEU	A	76	41.905	47.591	28.171	1.0	1.36	PROT	H
ATOM	249	HD12	LEU	A	76	41.712	47.503	29.923	1.0	1.54	PROT	H
ATOM	250	HD13	LEU	A	76	42.704	48.786	29.206	1.0	1.30	PROT	H
ATOM	251	HD21	LEU	A	76	45.257	46.199	27.915	1.0	1.30	PROT	H
ATOM	252	HD22	LEU	A	76	43.817	46.760	27.063	1.0	1.39	PROT	H
ATOM	253	HD23	LEU	A	76	44.893	47.924	27.835	1.0	1.36	PROT	H
ATOM	254	N	NME	A	77	47.759	45.468	31.374	1.0	-5.99	PROT	N
ATOM	255	H1	NME	A	77	47.802	44.744	30.671	1.0	3.30	PROT	H
ATOM	256	H2	NME	A	77	48.639	45.653	31.838	1.0	3.23	PROT	H
ATOM	257	C	ACE	A	78	51.228	51.078	33.391	1.0	4.47	PROT	C
ATOM	258	O	ACE	A	78	50.065	51.381	33.053	1.0	-6.52	PROT	O
ATOM	259	HC	ACE	A	78	51.857	50.525	32.659	1.0	1.49	PROT	H
ATOM	260	N	THR	A	80	51.750	51.370	34.530	1.0	-4.85	PROT	N
ATOM	261	CA	THR	A	80	51.020	52.090	35.540	1.0	-0.64	PROT	C
ATOM	262	CB	THR	A	80	49.660	51.390	35.890	1.0	2.02	PROT	C
ATOM	263	CG2	THR	A	80	49.840	50.080	36.450	1.0	-5.29	PROT	C
ATOM	264	OG1	THR	A	80	49.040	52.280	36.800	1.0	-6.48	PROT	O
ATOM	265	C	THR	A	80	51.920	52.280	36.780	1.0	5.78	PROT	C

ATOM	266	O	THR	A	80	52.550	51.290	37.140	1.0	-6.18	PROT	O
ATOM	267	H	THR	A	80	52.665	51.000	34.831	1.0	3.47	PROT	H
ATOM	268	HA	THR	A	80	50.737	53.102	35.116	1.0	1.71	PROT	H
ATOM	269	HB	THR	A	80	49.003	51.422	34.975	1.0	1.72	PROT	H
ATOM	270	HG21	THR	A	80	50.474	50.070	37.360	1.0	1.84	PROT	H
ATOM	271	HG22	THR	A	80	48.886	49.611	36.764	1.0	1.77	PROT	H
ATOM	272	HG23	THR	A	80	50.313	49.364	35.756	1.0	1.72	PROT	H
ATOM	273	HG1	THR	A	80	48.529	51.790	37.490	1.0	3.72	PROT	H
ATOM	274	N	PRO	A	81	51.850	53.400	37.530	1.0	-4.75	PROT	N
ATOM	275	CD	PRO	A	81	51.370	54.680	37.060	1.0	-0.85	PROT	C
ATOM	276	CG	PRO	A	81	51.240	55.570	38.250	1.0	-2.84	PROT	C
ATOM	277	CB	PRO	A	81	52.260	55.040	39.270	1.0	-2.82	PROT	C
ATOM	278	CA	PRO	A	81	52.440	53.530	38.890	1.0	0.24	PROT	C
ATOM	279	C	PRO	A	81	51.660	52.620	39.890	1.0	5.72	PROT	C
ATOM	280	O	PRO	A	81	52.320	52.010	40.730	1.0	-6.17	PROT	O
ATOM	281	HA	PRO	A	81	53.510	53.210	38.894	1.0	1.88	PROT	H
ATOM	282	HB2	PRO	A	81	51.926	55.164	40.311	1.0	1.60	PROT	H
ATOM	283	HB3	PRO	A	81	53.227	55.567	39.190	1.0	1.65	PROT	H
ATOM	284	HG2	PRO	A	81	50.217	55.550	38.677	1.0	1.53	PROT	H
ATOM	285	HG3	PRO	A	81	51.430	56.635	38.008	1.0	1.70	PROT	H
ATOM	286	HD2	PRO	A	81	50.385	54.556	36.536	1.0	1.60	PROT	H
ATOM	287	HD3	PRO	A	81	52.078	55.103	36.301	1.0	1.53	PROT	H
ATOM	288	N	VAL	A	82	50.350	52.350	39.650	1.0	-5.40	PROT	N
ATOM	289	CA	VAL	A	82	49.460	51.790	40.680	1.0	-0.09	PROT	C
ATOM	290	CB	VAL	A	82	48.690	52.900	41.420	1.0	-0.98	PROT	C
ATOM	291	CG1	VAL	A	82	49.510	54.000	42.120	1.0	-4.43	PROT	C
ATOM	292	CG2	VAL	A	82	47.830	53.720	40.430	1.0	-4.63	PROT	C
ATOM	293	C	VAL	A	82	48.430	50.740	40.130	1.0	5.94	PROT	C
ATOM	294	O	VAL	A	82	48.020	50.820	39.010	1.0	-5.88	PROT	O
ATOM	295	H	VAL	A	82	49.882	52.735	38.827	1.0	3.43	PROT	H
ATOM	296	HA	VAL	A	82	50.121	51.252	41.428	1.0	1.89	PROT	H
ATOM	297	HB	VAL	A	82	48.028	52.413	42.177	1.0	1.53	PROT	H
ATOM	298	HG11	VAL	A	82	50.199	53.581	42.863	1.0	1.50	PROT	H
ATOM	299	HG12	VAL	A	82	50.112	54.576	41.410	1.0	1.44	PROT	H
ATOM	300	HG13	VAL	A	82	48.849	54.701	42.643	1.0	1.55	PROT	H
ATOM	301	HG21	VAL	A	82	47.188	54.435	40.962	1.0	1.61	PROT	H
ATOM	302	HG22	VAL	A	82	48.441	54.294	39.729	1.0	1.44	PROT	H
ATOM	303	HG23	VAL	A	82	47.166	53.077	39.838	1.0	1.57	PROT	H

ATOM	304	N	ASN	A	83	47.920	49.820	40.970	1.0	-5.53	PROT	N
ATOM	305	CA	ASN	A	83	46.840	48.890	40.580	1.0	0.57	PROT	C
ATOM	306	CB	ASN	A	83	46.960	47.480	41.220	1.0	-3.94	PROT	C
ATOM	307	CG	ASN	A	83	48.300	46.840	41.040	1.0	6.26	PROT	C
ATOM	308	OD1	ASN	A	83	48.640	46.480	39.940	1.0	-6.37	PROT	O
ATOM	309	ND2	ASN	A	83	49.160	46.700	42.040	1.0	-6.35	PROT	N
ATOM	310	C	ASN	A	83	45.300	49.390	40.720	1.0	5.65	PROT	C
ATOM	311	O	ASN	A	83	44.860	50.070	41.640	1.0	-5.89	PROT	O
ATOM	312	H	ASN	A	83	48.148	49.817	41.961	1.0	3.29	PROT	H
ATOM	313	HA	ASN	A	83	46.963	48.747	39.453	1.0	2.01	PROT	H
ATOM	314	HB2	ASN	A	83	46.193	46.820	40.744	1.0	2.00	PROT	H
ATOM	315	HB3	ASN	A	83	46.664	47.530	42.290	1.0	1.80	PROT	H
ATOM	316	HD21	ASN	A	83	48.971	46.967	42.990	1.0	3.20	PROT	H
ATOM	317	HD22	ASN	A	83	50.065	46.276	41.888	1.0	3.28	PROT	H
ATOM	318	N	ILE	A	84	44.400	49.120	39.700	1.0	-6.22	PROT	N
ATOM	319	CA	ILE	A	84	43.140	49.810	39.290	1.0	0.21	PROT	C
ATOM	320	CB	ILE	A	84	43.470	50.840	38.190	1.0	-1.21	PROT	C
ATOM	321	CG2	ILE	A	84	42.250	51.270	37.320	1.0	-4.42	PROT	C
ATOM	322	CG1	ILE	A	84	44.230	52.090	38.700	1.0	-2.61	PROT	C
ATOM	323	CD1	ILE	A	84	45.180	52.650	37.660	1.0	-4.40	PROT	C
ATOM	324	C	ILE	A	84	42.180	48.670	38.870	1.0	5.75	PROT	C
ATOM	325	O	ILE	A	84	42.210	48.200	37.720	1.0	-6.31	PROT	O
ATOM	326	H	ILE	A	84	44.706	48.523	38.913	1.0	3.40	PROT	H
ATOM	327	HA	ILE	A	84	42.725	50.353	40.184	1.0	1.64	PROT	H
ATOM	328	HB	ILE	A	84	44.164	50.301	37.476	1.0	1.70	PROT	H
ATOM	329	HG12	ILE	A	84	44.796	51.828	39.618	1.0	1.46	PROT	H
ATOM	330	HG13	ILE	A	84	43.502	52.861	39.016	1.0	1.36	PROT	H
ATOM	331	HG21	ILE	A	84	42.577	51.948	36.526	1.0	1.48	PROT	H
ATOM	332	HG22	ILE	A	84	41.788	50.399	36.841	1.0	1.61	PROT	H
ATOM	333	HG23	ILE	A	84	41.493	51.784	37.916	1.0	1.39	PROT	H
ATOM	334	HD11	ILE	A	84	45.645	53.583	38.001	1.0	1.40	PROT	H
ATOM	335	HD12	ILE	A	84	46.000	51.946	37.449	1.0	1.57	PROT	H
ATOM	336	HD13	ILE	A	84	44.673	52.861	36.711	1.0	1.42	PROT	H
ATOM	337	N	NME	A	85	41.352	48.249	39.801	1.0	-6.08	PROT	N
ATOM	338	H1	NME	A	85	41.372	48.609	40.745	1.0	3.37	PROT	H
ATOM	339	H2	NME	A	85	40.728	47.471	39.636	1.0	3.25	PROT	H
TER	340		NME	A	85							
ATOM	341	C	ACE	B	7	29.168	52.319	32.078	1.0	4.32	PROT	C

ATOM	342	O	ACE	B	7	30.375	52.415	31.774	1.0	-6.22	PROT	O
ATOM	343	HC	ACE	B	7	28.553	51.548	31.561	1.0	1.50	PROT	H
ATOM	344	N	ARG	B	8	28.580	53.060	32.950	1.0	-4.99	PROT	N
ATOM	345	CA	ARG	B	8	29.300	54.120	33.690	1.0	-0.23	PROT	C
ATOM	346	CB	ARG	B	8	29.990	55.170	32.680	1.0	-2.65	PROT	C
ATOM	347	CG	ARG	B	8	30.950	54.460	31.710	1.0	-2.80	PROT	C
ATOM	348	CD	ARG	B	8	31.550	55.410	30.730	1.0	-0.84	PROT	C
ATOM	349	NE	ARG	B	8	32.540	54.780	29.820	1.0	-5.63	PROT	N
ATOM	350	CZ	ARG	B	8	32.660	55.070	28.530	1.0	6.14	PROT	C
ATOM	351	NH1	ARG	B	8	31.950	56.060	27.970	1.0	-6.41	PROT	N
ATOM	352	NH2	ARG	B	8	33.430	54.330	27.770	1.0	-6.26	PROT	N
ATOM	353	C	ARG	B	8	28.350	54.840	34.660	1.0	5.52	PROT	C
ATOM	354	O	ARG	B	8	27.240	55.240	34.230	1.0	-6.45	PROT	O
ATOM	355	H	ARG	B	8	27.567	53.050	33.105	1.0	3.40	PROT	H
ATOM	356	HA	ARG	B	8	30.148	53.623	34.242	1.0	1.79	PROT	H
ATOM	357	HB2	ARG	B	8	30.523	55.921	33.284	1.0	1.44	PROT	H
ATOM	358	HB3	ARG	B	8	29.190	55.697	32.133	1.0	1.54	PROT	H
ATOM	359	HG2	ARG	B	8	30.347	53.860	30.966	1.0	1.98	PROT	H
ATOM	360	HG3	ARG	B	8	31.715	53.858	32.240	1.0	1.94	PROT	H
ATOM	361	HD2	ARG	B	8	32.100	56.241	31.253	1.0	1.51	PROT	H
ATOM	362	HD3	ARG	B	8	30.738	55.912	30.149	1.0	1.40	PROT	H
ATOM	363	HE	ARG	B	8	33.091	53.945	30.258	1.0	4.20	PROT	H
ATOM	364	HH11	ARG	B	8	31.388	56.697	28.517	1.0	3.42	PROT	H
ATOM	365	HH12	ARG	B	8	32.103	56.347	27.013	1.0	3.39	PROT	H
ATOM	366	HH21	ARG	B	8	34.042	53.589	28.216	1.0	3.91	PROT	H
ATOM	367	HH22	ARG	B	8	33.711	54.601	26.836	1.0	3.41	PROT	H
ATOM	368	N	NME	B	9	28.790	54.981	35.891	1.0	-5.88	PROT	N
ATOM	369	H1	NME	B	9	29.679	54.625	36.217	1.0	3.35	PROT	H
ATOM	370	H2	NME	B	9	28.244	55.451	36.603	1.0	3.30	PROT	H
ATOM	371	C	ACE	B	10	30.360	61.685	34.772	1.0	4.81	PROT	C
ATOM	372	O	ACE	B	10	30.357	60.764	33.928	1.0	-6.85	PROT	O
ATOM	373	HC	ACE	B	10	30.002	62.688	34.449	1.0	1.52	PROT	H
ATOM	374	N	LEU	B	23	30.750	61.550	35.990	1.0	-4.94	PROT	N
ATOM	375	CA	LEU	B	23	31.240	60.270	36.510	1.0	-0.03	PROT	C
ATOM	376	CB	LEU	B	23	32.430	59.830	35.620	1.0	-3.33	PROT	C
ATOM	377	CG	LEU	B	23	32.800	58.360	35.630	1.0	-0.63	PROT	C
ATOM	378	CD1	LEU	B	23	31.910	57.400	34.950	1.0	-4.53	PROT	C
ATOM	379	CD2	LEU	B	23	34.090	58.300	34.790	1.0	-4.58	PROT	C

ATOM	380	C	LEU	B	23	31.630	60.480	38.040	1.0	5.63	PROT	C
ATOM	381	O	LEU	B	23	32.490	61.300	38.420	1.0	-6.10	PROT	O
ATOM	382	H	LEU	B	23	31.010	62.373	36.555	1.0	3.45	PROT	H
ATOM	383	HA	LEU	B	23	30.422	59.507	36.423	1.0	1.70	PROT	H
ATOM	384	HB2	LEU	B	23	33.306	60.457	35.901	1.0	1.64	PROT	H
ATOM	385	HB3	LEU	B	23	32.221	60.121	34.562	1.0	1.98	PROT	H
ATOM	386	HG	LEU	B	23	33.003	58.035	36.681	1.0	1.27	PROT	H
ATOM	387	HD11	LEU	B	23	31.712	57.683	33.903	1.0	1.54	PROT	H
ATOM	388	HD12	LEU	B	23	32.326	56.379	34.941	1.0	1.60	PROT	H
ATOM	389	HD13	LEU	B	23	30.920	57.331	35.427	1.0	1.38	PROT	H
ATOM	390	HD21	LEU	B	23	34.460	57.273	34.674	1.0	1.54	PROT	H
ATOM	391	HD22	LEU	B	23	33.927	58.687	33.777	1.0	1.48	PROT	H
ATOM	392	HD23	LEU	B	23	34.901	58.886	35.235	1.0	1.35	PROT	H
ATOM	393	N	LEU	B	24	30.950	59.630	38.860	1.0	-5.55	PROT	N
ATOM	394	CA	LEU	B	24	31.230	59.470	40.290	1.0	0.12	PROT	C
ATOM	395	CB	LEU	B	24	29.960	59.030	40.950	1.0	-3.33	PROT	C
ATOM	396	CG	LEU	B	24	28.730	59.970	40.960	1.0	-0.46	PROT	C
ATOM	397	CD1	LEU	B	24	27.640	59.320	41.870	1.0	-4.61	PROT	C
ATOM	398	CD2	LEU	B	24	29.160	61.370	41.420	1.0	-4.62	PROT	C
ATOM	399	C	LEU	B	24	32.470	58.590	40.530	1.0	5.87	PROT	C
ATOM	400	O	LEU	B	24	32.280	57.400	40.590	1.0	-6.06	PROT	O
ATOM	401	H	LEU	B	24	30.264	58.977	38.479	1.0	3.40	PROT	H
ATOM	402	HA	LEU	B	24	31.491	60.510	40.694	1.0	1.88	PROT	H
ATOM	403	HB2	LEU	B	24	30.194	58.777	42.016	1.0	1.77	PROT	H
ATOM	404	HB3	LEU	B	24	29.639	58.047	40.529	1.0	1.64	PROT	H
ATOM	405	HG	LEU	B	24	28.315	60.046	39.928	1.0	1.30	PROT	H
ATOM	406	HD11	LEU	B	24	26.722	59.913	41.849	1.0	1.48	PROT	H
ATOM	407	HD12	LEU	B	24	27.398	58.310	41.529	1.0	1.46	PROT	H
ATOM	408	HD13	LEU	B	24	27.976	59.261	42.909	1.0	1.54	PROT	H
ATOM	409	HD21	LEU	B	24	28.288	62.011	41.595	1.0	1.48	PROT	H
ATOM	410	HD22	LEU	B	24	29.725	61.332	42.358	1.0	1.57	PROT	H
ATOM	411	HD23	LEU	B	24	29.778	61.867	40.665	1.0	1.47	PROT	H
ATOM	412	N	ASH	B	25	33.720	59.180	40.580	1.0	-5.75	PROT	N
ATOM	413	CA	ASH	B	25	35.010	58.500	40.590	1.0	0.50	PROT	C
ATOM	414	CB	ASH	B	25	36.060	59.410	39.890	1.0	-3.73	PROT	C
ATOM	415	CG	ASH	B	25	37.250	58.620	39.480	1.0	6.81	PROT	C
ATOM	416	OD1	ASH	B	25	37.450	57.520	39.900	1.0	-5.83	PROT	O
ATOM	417	OD2	ASH	B	25	38.050	59.140	38.570	1.0	-5.64	PROT	O

ATOM	418	C	ASH	B	25	35.420	58.150	42.000	1.0	5.46	PROT	C
ATOM	419	O	ASH	B	25	35.910	59.060	42.700	1.0	-5.83	PROT	O
ATOM	420	H	ASH	B	25	33.761	60.198	40.467	1.0	3.41	PROT	H
ATOM	421	HA	ASH	B	25	34.909	57.543	39.986	1.0	1.70	PROT	H
ATOM	422	HB2	ASH	B	25	36.372	60.234	40.589	1.0	2.34	PROT	H
ATOM	423	HB3	ASH	B	25	35.607	59.927	39.014	1.0	1.95	PROT	H
ATOM	424	HD2	ASH	B	25	38.892	58.580	38.416	1.0	4.06	PROT	H
ATOM	425	N	THR	B	26	35.490	56.860	42.420	1.0	-5.52	PROT	N
ATOM	426	CA	THR	B	26	36.200	56.490	43.660	1.0	-0.18	PROT	C
ATOM	427	CB	THR	B	26	35.720	55.100	44.120	1.0	1.85	PROT	C
ATOM	428	CG2	THR	B	26	34.290	55.110	44.630	1.0	-5.03	PROT	C
ATOM	429	OG1	THR	B	26	35.880	54.240	43.040	1.0	-6.47	PROT	O
ATOM	430	C	THR	B	26	37.740	56.480	43.510	1.0	5.77	PROT	C
ATOM	431	O	THR	B	26	38.390	56.560	44.540	1.0	-5.96	PROT	O
ATOM	432	H	THR	B	26	35.047	56.094	41.911	1.0	3.51	PROT	H
ATOM	433	HA	THR	B	26	35.970	57.260	44.460	1.0	2.12	PROT	H
ATOM	434	HB	THR	B	26	36.404	54.756	44.947	1.0	1.38	PROT	H
ATOM	435	HG21	THR	B	26	34.149	55.828	45.446	1.0	1.69	PROT	H
ATOM	436	HG22	THR	B	26	33.579	55.362	43.830	1.0	1.70	PROT	H
ATOM	437	HG23	THR	B	26	33.998	54.119	44.998	1.0	1.58	PROT	H
ATOM	438	HG1	THR	B	26	36.174	53.338	43.284	1.0	3.76	PROT	H
ATOM	439	N	GLY	B	27	38.260	56.450	42.270	1.0	-5.75	PROT	N
ATOM	440	CA	GLY	B	27	39.740	56.480	41.890	1.0	-1.23	PROT	C
ATOM	441	C	GLY	B	27	40.330	57.910	41.900	1.0	5.78	PROT	C
ATOM	442	O	GLY	B	27	41.420	58.110	41.360	1.0	-6.56	PROT	O
ATOM	443	H	GLY	B	27	37.655	56.369	41.454	1.0	3.52	PROT	H
ATOM	444	HA2	GLY	B	27	39.825	56.063	40.864	1.0	1.77	PROT	H
ATOM	445	HA3	GLY	B	27	40.318	55.852	42.601	1.0	1.79	PROT	H
ATOM	446	N	ALA	B	28	39.670	58.940	42.440	1.0	-4.97	PROT	N
ATOM	447	CA	ALA	B	28	40.120	60.290	42.500	1.0	0.03	PROT	C
ATOM	448	CB	ALA	B	28	39.590	61.140	41.340	1.0	-4.57	PROT	C
ATOM	449	C	ALA	B	28	39.940	60.960	43.800	1.0	5.55	PROT	C
ATOM	450	O	ALA	B	28	38.830	60.930	44.390	1.0	-6.07	PROT	O
ATOM	451	H	ALA	B	28	38.733	58.783	42.839	1.0	3.32	PROT	H
ATOM	452	HA	ALA	B	28	41.264	60.238	42.296	1.0	1.96	PROT	H
ATOM	453	HB1	ALA	B	28	38.495	61.228	41.366	1.0	1.61	PROT	H
ATOM	454	HB2	ALA	B	28	39.979	62.170	41.385	1.0	1.87	PROT	H
ATOM	455	HB3	ALA	B	28	39.869	60.713	40.372	1.0	1.50	PROT	H

ATOM	456	N	ASP	B	29	40.930	61.740	44.280	1.0	-5.42	PROT	N
ATOM	457	CA	ASP	B	29	40.810	62.450	45.520	1.0	0.33	PROT	C
ATOM	458	CB	ASP	B	29	42.190	62.700	46.150	1.0	-4.17	PROT	C
ATOM	459	CG	ASP	B	29	43.020	61.420	46.160	1.0	7.62	PROT	C
ATOM	460	OD1	ASP	B	29	42.940	60.630	47.140	1.0	-8.21	PROT	O
ATOM	461	OD2	ASP	B	29	43.780	61.200	45.220	1.0	-8.31	PROT	O
ATOM	462	C	ASP	B	29	40.070	63.760	45.300	1.0	5.93	PROT	C
ATOM	463	O	ASP	B	29	39.540	64.280	46.280	1.0	-6.59	PROT	O
ATOM	464	H	ASP	B	29	41.883	61.701	43.888	1.0	3.47	PROT	H
ATOM	465	HA	ASP	B	29	40.186	61.841	46.254	1.0	2.00	PROT	H
ATOM	466	HB2	ASP	B	29	42.073	63.081	47.184	1.0	1.90	PROT	H
ATOM	467	HB3	ASP	B	29	42.742	63.493	45.590	1.0	2.05	PROT	H
ATOM	468	N	ASP	B	30	40.150	64.370	44.110	1.0	-5.16	PROT	N
ATOM	469	CA	ASP	B	30	39.760	65.780	43.850	1.0	0.41	PROT	C
ATOM	470	CB	ASP	B	30	40.970	66.710	43.520	1.0	-4.33	PROT	C
ATOM	471	CG	ASP	B	30	42.290	66.120	44.080	1.0	7.46	PROT	C
ATOM	472	OD1	ASP	B	30	42.810	65.160	43.420	1.0	-7.93	PROT	O
ATOM	473	OD2	ASP	B	30	42.760	66.500	45.180	1.0	-8.09	PROT	O
ATOM	474	C	ASP	B	30	38.820	65.780	42.650	1.0	5.50	PROT	C
ATOM	475	O	ASP	B	30	38.680	64.850	41.920	1.0	-5.99	PROT	O
ATOM	476	H	ASP	B	30	40.724	63.978	43.355	1.0	3.46	PROT	H
ATOM	477	HA	ASP	B	30	39.238	66.168	44.770	1.0	1.86	PROT	H
ATOM	478	HB2	ASP	B	30	40.804	67.710	43.948	1.0	1.63	PROT	H
ATOM	479	HB3	ASP	B	30	41.101	66.826	42.431	1.0	1.63	PROT	H
ATOM	480	N	THR	B	31	38.190	66.850	42.410	1.0	-5.17	PROT	N
ATOM	481	CA	THR	B	31	37.210	66.970	41.300	1.0	-1.18	PROT	C
ATOM	482	CB	THR	B	31	35.820	67.560	41.760	1.0	2.00	PROT	C
ATOM	483	CG2	THR	B	31	34.900	67.870	40.570	1.0	-4.98	PROT	C
ATOM	484	OG1	THR	B	31	35.290	66.540	42.580	1.0	-6.19	PROT	O
ATOM	485	C	THR	B	31	37.710	67.720	40.140	1.0	5.91	PROT	C
ATOM	486	O	THR	B	31	38.020	68.890	40.270	1.0	-6.36	PROT	O
ATOM	487	H	THR	B	31	38.323	67.738	42.910	1.0	3.37	PROT	H
ATOM	488	HA	THR	B	31	36.955	65.889	40.986	1.0	2.27	PROT	H
ATOM	489	HB	THR	B	31	35.956	68.464	42.396	1.0	1.36	PROT	H
ATOM	490	HG21	THR	B	31	33.881	68.075	40.915	1.0	1.59	PROT	H
ATOM	491	HG22	THR	B	31	35.257	68.745	40.016	1.0	1.67	PROT	H
ATOM	492	HG23	THR	B	31	34.836	67.021	39.874	1.0	1.74	PROT	H
ATOM	493	HG1	THR	B	31	35.970	66.201	43.203	1.0	3.59	PROT	H

ATOM	494	N	VAL	B	32	37.630	67.160	38.960	1.0	-5.35	PROT	N
ATOM	495	CA	VAL	B	32	38.110	67.650	37.650	1.0	-0.19	PROT	C
ATOM	496	CB	VAL	B	32	39.200	66.720	37.000	1.0	-0.86	PROT	C
ATOM	497	CG1	VAL	B	32	40.140	67.340	36.020	1.0	-4.59	PROT	C
ATOM	498	CG2	VAL	B	32	40.090	66.090	38.150	1.0	-4.35	PROT	C
ATOM	499	C	VAL	B	32	36.950	67.820	36.650	1.0	5.77	PROT	C
ATOM	500	O	VAL	B	32	36.150	66.880	36.430	1.0	-6.75	PROT	O
ATOM	501	H	VAL	B	32	37.207	66.216	38.868	1.0	3.40	PROT	H
ATOM	502	HA	VAL	B	32	38.603	68.656	37.835	1.0	1.96	PROT	H
ATOM	503	HB	VAL	B	32	38.650	65.878	36.507	1.0	1.42	PROT	H
ATOM	504	HG11	VAL	B	32	40.758	68.132	36.473	1.0	1.62	PROT	H
ATOM	505	HG12	VAL	B	32	40.825	66.598	35.585	1.0	1.56	PROT	H
ATOM	506	HG13	VAL	B	32	39.620	67.815	35.176	1.0	1.42	PROT	H
ATOM	507	HG21	VAL	B	32	40.922	65.527	37.719	1.0	1.48	PROT	H
ATOM	508	HG22	VAL	B	32	40.493	66.878	38.791	1.0	1.55	PROT	H
ATOM	509	HG23	VAL	B	32	39.508	65.404	38.771	1.0	1.52	PROT	H
ATOM	510	N	NME	B	33	36.869	68.994	36.064	1.0	-6.08	PROT	N
ATOM	511	H1	NME	B	33	37.562	69.730	36.160	1.0	3.46	PROT	H
ATOM	512	H2	NME	B	33	36.161	69.166	35.359	1.0	3.19	PROT	H
ATOM	513	C	ACE	B	34	47.875	69.464	37.458	1.0	4.51	PROT	C
ATOM	514	O	ACE	B	34	47.356	69.458	38.594	1.0	-6.41	PROT	O
ATOM	515	HC	ACE	B	34	48.171	70.442	37.018	1.0	1.43	PROT	H
ATOM	516	N	ILE	B	47	48.080	68.410	36.750	1.0	-5.01	PROT	N
ATOM	517	CA	ILE	B	47	47.710	67.060	37.230	1.0	-0.34	PROT	C
ATOM	518	CB	ILE	B	47	46.200	67.000	37.540	1.0	-1.10	PROT	C
ATOM	519	CG2	ILE	B	47	45.980	67.830	38.860	1.0	-3.97	PROT	C
ATOM	520	CG1	ILE	B	47	45.250	67.340	36.360	1.0	-2.65	PROT	C
ATOM	521	CD1	ILE	B	47	43.820	67.110	36.770	1.0	-4.38	PROT	C
ATOM	522	C	ILE	B	47	48.040	65.890	36.220	1.0	5.80	PROT	C
ATOM	523	O	ILE	B	47	48.170	66.020	35.040	1.0	-5.75	PROT	O
ATOM	524	H	ILE	B	47	48.442	68.431	35.793	1.0	3.36	PROT	H
ATOM	525	HA	ILE	B	47	48.299	66.867	38.172	1.0	1.84	PROT	H
ATOM	526	HB	ILE	B	47	45.971	65.932	37.811	1.0	1.40	PROT	H
ATOM	527	HG12	ILE	B	47	45.520	66.716	35.486	1.0	1.38	PROT	H
ATOM	528	HG13	ILE	B	47	45.406	68.387	36.040	1.0	1.38	PROT	H
ATOM	529	HG21	ILE	B	47	45.190	67.325	39.446	1.0	1.29	PROT	H
ATOM	530	HG22	ILE	B	47	46.852	67.829	39.517	1.0	1.61	PROT	H
ATOM	531	HG23	ILE	B	47	45.563	68.822	38.663	1.0	1.63	PROT	H

ATOM	532	HD11	ILE	B	47	43.648	66.075	37.100	1.0	1.52	PROT H
ATOM	533	HD12	ILE	B	47	43.517	67.771	37.594	1.0	1.56	PROT H
ATOM	534	HD13	ILE	B	47	43.129	67.296	35.937	1.0	1.41	PROT H
ATOM	535	N	GLY	B	48	48.400	64.690	36.840	1.0	-5.84	PROT N
ATOM	536	CA	GLY	B	48	48.940	63.600	36.020	1.0	-1.41	PROT C
ATOM	537	C	GLY	B	48	47.900	62.460	36.030	1.0	5.64	PROT C
ATOM	538	O	GLY	B	48	46.700	62.640	36.070	1.0	-5.46	PROT O
ATOM	539	H	GLY	B	48	48.191	64.484	37.804	1.0	3.34	PROT H
ATOM	540	HA2	GLY	B	48	49.927	63.285	36.423	1.0	1.69	PROT H
ATOM	541	HA3	GLY	B	48	49.100	63.922	34.961	1.0	1.99	PROT H
ATOM	542	N	GLY	B	49	48.410	61.220	35.880	1.0	-5.94	PROT N
ATOM	543	CA	GLY	B	49	47.570	60.060	35.350	1.0	-1.23	PROT C
ATOM	544	C	GLY	B	49	48.510	58.940	34.820	1.0	5.72	PROT C
ATOM	545	O	GLY	B	49	49.710	59.130	35.050	1.0	-6.29	PROT O
ATOM	546	H	GLY	B	49	49.408	61.028	35.830	1.0	3.38	PROT H
ATOM	547	HA2	GLY	B	49	46.902	60.438	34.550	1.0	1.68	PROT H
ATOM	548	HA3	GLY	B	49	46.931	59.677	36.176	1.0	1.85	PROT H
ATOM	549	N	ILE	B	50	47.940	57.900	34.170	1.0	-5.60	PROT N
ATOM	550	CA	ILE	B	50	48.710	57.000	33.300	1.0	-0.31	PROT C
ATOM	551	CB	ILE	B	50	47.830	55.700	33.210	1.0	-0.70	PROT C
ATOM	552	CG2	ILE	B	50	48.400	54.870	32.110	1.0	-4.84	PROT C
ATOM	553	CG1	ILE	B	50	47.970	54.930	34.560	1.0	-2.45	PROT C
ATOM	554	CD1	ILE	B	50	46.790	54.010	34.710	1.0	-4.41	PROT C
ATOM	555	C	ILE	B	50	49.200	57.730	32.030	1.0	5.68	PROT C
ATOM	556	O	ILE	B	50	48.410	58.430	31.350	1.0	-6.70	PROT O
ATOM	557	H	ILE	B	50	46.916	57.805	34.145	1.0	3.63	PROT H
ATOM	558	HA	ILE	B	50	49.644	56.706	33.875	1.0	1.65	PROT H
ATOM	559	HB	ILE	B	50	46.767	55.966	33.020	1.0	1.49	PROT H
ATOM	560	HG12	ILE	B	50	48.014	55.637	35.411	1.0	1.47	PROT H
ATOM	561	HG13	ILE	B	50	48.918	54.361	34.582	1.0	1.39	PROT H
ATOM	562	HG21	ILE	B	50	49.465	54.635	32.252	1.0	1.61	PROT H
ATOM	563	HG22	ILE	B	50	47.901	53.888	32.034	1.0	1.71	PROT H
ATOM	564	HG23	ILE	B	50	48.302	55.336	31.119	1.0	1.50	PROT H
ATOM	565	HD11	ILE	B	50	46.653	53.362	33.835	1.0	1.42	PROT H
ATOM	566	HD12	ILE	B	50	46.928	53.336	35.574	1.0	1.64	PROT H
ATOM	567	HD13	ILE	B	50	45.854	54.558	34.872	1.0	1.41	PROT H
ATOM	568	N	NME	B	51	50.470	57.564	31.730	1.0	-5.76	PROT N
ATOM	569	H1	NME	B	51	51.102	56.980	32.256	1.0	3.32	PROT H

ATOM	570	H2	NME	B	51	50.883	58.003	30.913	1.0	3.33	PROT H
ATOM	571	C	ACE	B	52	38.991	71.486	40.857	1.0	4.39	PROT C
ATOM	572	O	ACE	B	52	40.090	71.587	41.439	1.0	-6.13	PROT O
ATOM	573	HC	ACE	B	52	38.078	71.302	41.468	1.0	1.48	PROT H
ATOM	574	N	LEU	B	76	38.840	71.530	39.580	1.0	-5.04	PROT N
ATOM	575	CA	LEU	B	76	39.990	71.710	38.660	1.0	-0.25	PROT C
ATOM	576	CB	LEU	B	76	41.090	70.730	38.920	1.0	-3.07	PROT C
ATOM	577	CG	LEU	B	76	41.650	70.790	40.370	1.0	-0.79	PROT C
ATOM	578	CD1	LEU	B	76	42.420	69.530	40.700	1.0	-4.68	PROT C
ATOM	579	CD2	LEU	B	76	42.460	71.980	40.700	1.0	-4.27	PROT C
ATOM	580	C	LEU	B	76	39.490	71.720	37.240	1.0	5.75	PROT C
ATOM	581	O	LEU	B	76	39.190	70.680	36.650	1.0	-6.55	PROT O
ATOM	582	H	LEU	B	76	38.014	71.102	39.145	1.0	3.32	PROT H
ATOM	583	HA	LEU	B	76	40.412	72.742	38.918	1.0	1.72	PROT H
ATOM	584	HB2	LEU	B	76	41.926	70.886	38.208	1.0	1.45	PROT H
ATOM	585	HB3	LEU	B	76	40.729	69.695	38.722	1.0	1.62	PROT H
ATOM	586	HG	LEU	B	76	40.854	70.408	41.110	1.0	2.33	PROT H
ATOM	587	HD11	LEU	B	76	41.827	68.620	40.531	1.0	1.39	PROT H
ATOM	588	HD12	LEU	B	76	43.333	69.418	40.098	1.0	1.35	PROT H
ATOM	589	HD13	LEU	B	76	42.742	69.520	41.752	1.0	1.57	PROT H
ATOM	590	HD21	LEU	B	76	41.924	72.919	40.503	1.0	1.29	PROT H
ATOM	591	HD22	LEU	B	76	42.727	72.011	41.767	1.0	1.48	PROT H
ATOM	592	HD23	LEU	B	76	43.396	72.018	40.124	1.0	1.27	PROT H
ATOM	593	N	NME	B	77	39.396	72.904	36.676	1.0	-6.05	PROT N
ATOM	594	H1	NME	B	77	39.585	73.775	37.151	1.0	3.30	PROT H
ATOM	595	H2	NME	B	77	39.064	73.016	35.727	1.0	3.20	PROT H
ATOM	596	C	ACE	B	78	41.236	68.291	31.186	1.0	4.56	PROT C
ATOM	597	O	ACE	B	78	41.774	67.761	32.180	1.0	-6.46	PROT O
ATOM	598	HC	ACE	B	78	41.610	69.286	30.856	1.0	1.55	PROT H
ATOM	599	N	THR	B	80	40.280	67.760	30.510	1.0	-4.85	PROT N
ATOM	600	CA	THR	B	80	39.730	66.470	30.850	1.0	-0.65	PROT C
ATOM	601	CB	THR	B	80	39.250	66.400	32.290	1.0	2.09	PROT C
ATOM	602	CG2	THR	B	80	38.060	67.280	32.680	1.0	-5.04	PROT C
ATOM	603	OG1	THR	B	80	38.840	65.080	32.450	1.0	-6.91	PROT O
ATOM	604	C	THR	B	80	38.600	66.060	29.890	1.0	5.72	PROT C
ATOM	605	O	THR	B	80	37.730	66.920	29.620	1.0	-6.05	PROT O
ATOM	606	H	THR	B	80	39.766	68.268	29.775	1.0	3.47	PROT H
ATOM	607	HA	THR	B	80	40.567	65.701	30.754	1.0	1.79	PROT H

ATOM	608	HB	THR	B	80	40.114	66.538	32.998	1.0	1.78	PROT H
ATOM	609	HG21	THR	B	80	38.289	68.347	32.583	1.0	1.67	PROT H
ATOM	610	HG22	THR	B	80	37.181	67.081	32.048	1.0	1.81	PROT H
ATOM	611	HG23	THR	B	80	37.757	67.093	33.720	1.0	1.74	PROT H
ATOM	612	HG1	THR	B	80	37.916	64.997	32.798	1.0	3.96	PROT H
ATOM	613	N	PRO	B	81	38.500	64.820	29.350	1.0	-4.76	PROT N
ATOM	614	CD	PRO	B	81	39.530	63.830	29.010	1.0	-0.68	PROT C
ATOM	615	CG	PRO	B	81	38.900	62.720	28.180	1.0	-2.93	PROT C
ATOM	616	CB	PRO	B	81	37.920	63.450	27.370	1.0	-2.84	PROT C
ATOM	617	CA	PRO	B	81	37.430	64.540	28.350	1.0	-0.28	PROT C
ATOM	618	C	PRO	B	81	36.070	64.160	28.970	1.0	6.06	PROT C
ATOM	619	O	PRO	B	81	35.090	64.120	28.300	1.0	-6.15	PROT O
ATOM	620	HA	PRO	B	81	37.220	65.494	27.771	1.0	1.94	PROT H
ATOM	621	HB2	PRO	B	81	37.077	62.833	26.998	1.0	1.73	PROT H
ATOM	622	HB3	PRO	B	81	38.355	63.910	26.458	1.0	1.67	PROT H
ATOM	623	HG2	PRO	B	81	38.436	61.943	28.823	1.0	1.48	PROT H
ATOM	624	HG3	PRO	B	81	39.663	62.175	27.587	1.0	1.66	PROT H
ATOM	625	HD2	PRO	B	81	39.970	63.426	29.955	1.0	1.54	PROT H
ATOM	626	HD3	PRO	B	81	40.363	64.317	28.447	1.0	1.53	PROT H
ATOM	627	N	VAL	B	82	36.100	63.980	30.300	1.0	-5.74	PROT N
ATOM	628	CA	VAL	B	82	34.950	63.630	31.190	1.0	-0.34	PROT C
ATOM	629	CB	VAL	B	82	35.130	62.130	31.540	1.0	-0.67	PROT C
ATOM	630	CG1	VAL	B	82	36.420	61.820	32.270	1.0	-4.53	PROT C
ATOM	631	CG2	VAL	B	82	34.030	61.570	32.430	1.0	-4.80	PROT C
ATOM	632	C	VAL	B	82	34.960	64.560	32.360	1.0	6.04	PROT C
ATOM	633	O	VAL	B	82	36.040	64.890	32.800	1.0	-6.05	PROT O
ATOM	634	H	VAL	B	82	36.969	64.103	30.827	1.0	3.43	PROT H
ATOM	635	HA	VAL	B	82	34.002	63.736	30.581	1.0	1.93	PROT H
ATOM	636	HB	VAL	B	82	35.116	61.582	30.560	1.0	1.52	PROT H
ATOM	637	HG11	VAL	B	82	36.467	60.769	32.575	1.0	1.33	PROT H
ATOM	638	HG12	VAL	B	82	37.307	62.023	31.654	1.0	1.45	PROT H
ATOM	639	HG13	VAL	B	82	36.536	62.434	33.178	1.0	1.69	PROT H
ATOM	640	HG21	VAL	B	82	33.028	61.729	32.010	1.0	1.53	PROT H
ATOM	641	HG22	VAL	B	82	34.134	60.487	32.567	1.0	1.51	PROT H
ATOM	642	HG23	VAL	B	82	34.022	62.021	33.434	1.0	1.71	PROT H
ATOM	643	N	ASN	B	83	33.750	64.810	32.890	1.0	-5.47	PROT N
ATOM	644	CA	ASN	B	83	33.610	65.270	34.320	1.0	0.02	PROT C
ATOM	645	CB	ASN	B	83	32.340	66.090	34.630	1.0	-3.85	PROT C

ATOM	646	CG	ASN	B	83	32.200	67.210	33.660	1.0	6.36	PROT	C
ATOM	647	OD1	ASN	B	83	33.030	68.050	33.510	1.0	-6.18	PROT	O
ATOM	648	ND2	ASN	B	83	31.060	67.270	33.000	1.0	-6.49	PROT	N
ATOM	649	C	ASN	B	83	33.780	64.150	35.290	1.0	5.48	PROT	C
ATOM	650	O	ASN	B	83	32.950	63.230	35.220	1.0	-6.40	PROT	O
ATOM	651	H	ASN	B	83	32.913	64.360	32.523	1.0	3.34	PROT	H
ATOM	652	HA	ASN	B	83	34.478	66.022	34.437	1.0	2.14	PROT	H
ATOM	653	HB2	ASN	B	83	32.426	66.505	35.664	1.0	2.03	PROT	H
ATOM	654	HB3	ASN	B	83	31.454	65.420	34.669	1.0	1.82	PROT	H
ATOM	655	HD21	ASN	B	83	30.324	66.587	33.079	1.0	3.21	PROT	H
ATOM	656	HD22	ASN	B	83	30.871	68.010	32.337	1.0	3.20	PROT	H
ATOM	657	N	ILE	B	84	34.670	64.330	36.240	1.0	-5.11	PROT	N
ATOM	658	CA	ILE	B	84	34.870	63.420	37.400	1.0	-0.60	PROT	C
ATOM	659	CB	ILE	B	84	36.140	62.550	37.250	1.0	-0.86	PROT	C
ATOM	660	CG2	ILE	B	84	36.020	61.400	36.310	1.0	-4.63	PROT	C
ATOM	661	CG1	ILE	B	84	37.370	63.430	36.930	1.0	-2.61	PROT	C
ATOM	662	CD1	ILE	B	84	38.670	62.660	36.600	1.0	-4.27	PROT	C
ATOM	663	C	ILE	B	84	34.800	64.060	38.810	1.0	5.79	PROT	C
ATOM	664	O	ILE	B	84	35.710	64.720	39.300	1.0	-6.05	PROT	O
ATOM	665	H	ILE	B	84	35.289	65.159	36.272	1.0	3.68	PROT	H
ATOM	666	HA	ILE	B	84	33.997	62.680	37.343	1.0	1.90	PROT	H
ATOM	667	HB	ILE	B	84	36.330	62.119	38.280	1.0	1.51	PROT	H
ATOM	668	HG12	ILE	B	84	37.551	64.101	37.797	1.0	1.63	PROT	H
ATOM	669	HG13	ILE	B	84	37.147	64.094	36.072	1.0	1.37	PROT	H
ATOM	670	HG21	ILE	B	84	36.880	60.720	36.384	1.0	1.54	PROT	H
ATOM	671	HG22	ILE	B	84	35.118	60.798	36.498	1.0	1.40	PROT	H
ATOM	672	HG23	ILE	B	84	35.952	61.729	35.262	1.0	1.47	PROT	H
ATOM	673	HD11	ILE	B	84	39.527	63.345	36.613	1.0	1.49	PROT	H
ATOM	674	HD12	ILE	B	84	38.858	61.871	37.335	1.0	1.47	PROT	H
ATOM	675	HD13	ILE	B	84	38.622	62.212	35.605	1.0	1.25	PROT	H
ATOM	676	N	NME	B	85	33.684	63.855	39.476	1.0	-6.13	PROT	N
ATOM	677	H1	NME	B	85	32.925	63.273	39.155	1.0	3.28	PROT	H
ATOM	678	H2	NME	B	85	33.569	64.243	40.408	1.0	3.31	PROT	H
HETATM	679	N1	MK1	C	100	39.400	58.990	32.520	1.0	-1.17	PROT	N
HETATM	680	C1	MK1	C	100	40.460	57.890	32.570	1.0	-1.95	PROT	C
HETATM	681	C2	MK1	C	100	40.400	57.010	33.790	1.0	-0.79	PROT	C
HETATM	682	C3	MK1	C	100	41.740	56.100	33.690	1.0	5.91	PROT	C
HETATM	683	O1	MK1	C	100	42.680	56.290	34.380	1.0	-5.54	PROT	O

HETATM	684	N2	MK1	C	100	41.610	55.040	32.870	1.0	-5.94	PROT	N
HETATM	685	C4	MK1	C	100	42.630	54.020	32.690	1.0	3.21	PROT	C
HETATM	686	C5	MK1	C	100	42.140	53.040	31.630	1.0	-4.93	PROT	C
HETATM	687	C6	MK1	C	100	42.850	53.290	34.040	1.0	-4.89	PROT	C
HETATM	688	C7	MK1	C	100	44.050	54.530	32.290	1.0	-4.91	PROT	C
HETATM	689	N3	MK1	C	100	40.480	57.860	35.040	1.0	-3.90	PROT	N
HETATM	690	C8	MK1	C	100	39.430	58.950	35.080	1.0	-1.49	PROT	C
HETATM	691	C9	MK1	C	100	39.460	59.830	33.850	1.0	-1.50	PROT	C
HETATM	692	C10	MK1	C	100	40.310	57.060	36.290	1.0	-1.75	PROT	C
HETATM	693	C11	MK1	C	100	41.240	57.570	37.400	1.0	1.61	PROT	C
HETATM	694	O2	MK1	C	100	40.440	57.590	38.620	1.0	-6.65	PROT	O
HETATM	695	C12	MK1	C	100	42.510	56.790	37.500	1.0	-3.37	PROT	C
HETATM	696	C13	MK1	C	100	43.510	57.480	38.470	1.0	-1.94	PROT	C
HETATM	697	C14	MK1	C	100	44.800	56.550	38.730	1.0	-2.68	PROT	C
HETATM	698	C15	MK1	C	100	45.550	56.960	40.050	1.0	-0.24	PROT	C
HETATM	699	C16	MK1	C	100	45.050	56.510	41.300	1.0	-1.61	PROT	C
HETATM	700	C17	MK1	C	100	45.910	56.500	42.440	1.0	-1.33	PROT	C
HETATM	701	C18	MK1	C	100	47.220	57.030	42.320	1.0	-1.81	PROT	C
HETATM	702	C19	MK1	C	100	47.660	57.560	41.080	1.0	-1.31	PROT	C
HETATM	703	C20	MK1	C	100	46.800	57.600	39.950	1.0	-1.49	PROT	C
HETATM	704	C21	MK1	C	100	43.950	58.850	37.970	1.0	6.14	PROT	C
HETATM	705	O3	MK1	C	100	43.910	59.170	36.830	1.0	-6.19	PROT	O
HETATM	706	N4	MK1	C	100	44.380	59.750	38.910	1.0	-5.70	PROT	N
HETATM	707	C22	MK1	C	100	44.820	61.110	38.560	1.0	0.06	PROT	C
HETATM	708	C23	MK1	C	100	46.170	61.520	39.140	1.0	1.34	PROT	C
HETATM	709	O4	MK1	C	100	46.500	60.740	40.340	1.0	-5.92	PROT	O
HETATM	710	C24	MK1	C	100	46.050	62.990	39.470	1.0	-3.44	PROT	C
HETATM	711	C25	MK1	C	100	44.580	63.260	39.570	1.0	-0.06	PROT	C
HETATM	712	C26	MK1	C	100	43.970	64.460	39.920	1.0	-1.44	PROT	C
HETATM	713	C27	MK1	C	100	42.520	64.480	39.920	1.0	-0.92	PROT	C
HETATM	714	C28	MK1	C	100	41.790	63.320	39.580	1.0	-1.67	PROT	C
HETATM	715	C29	MK1	C	100	42.440	62.240	39.000	1.0	-1.41	PROT	C
HETATM	716	C30	MK1	C	100	43.860	62.200	39.040	1.0	-0.77	PROT	C
HETATM	717	C31	MK1	C	100	39.680	59.970	31.380	1.0	-1.54	PROT	C
HETATM	718	C32	MK1	C	100	39.340	59.220	30.060	1.0	-1.77	PROT	C
HETATM	719	C33	MK1	C	100	40.400	58.920	29.180	1.0	0.78	PROT	C
HETATM	720	N5	MK1	C	100	40.230	58.180	28.060	1.0	-4.16	PROT	N
HETATM	721	C34	MK1	C	100	39.050	57.720	27.730	1.0	0.79	PROT	C

```
HETATM  722  C35 MK1 C 100      37.940  58.110  28.500  1.0  -2.32      PROT C
HETATM  723  C36 MK1 C 100      38.100  58.840  29.690  1.0  -0.90      PROT C
HETATM  724  H1  MK1 C 100      38.457  58.563  32.424  1.0   3.06      PROT H
HETATM  725  H11 MK1 C 100      41.467  58.402  32.513  1.0   2.17      PROT H
HETATM  726  H12 MK1 C 100      40.371  57.291  31.632  1.0   1.80      PROT H
HETATM  727  H2  MK1 C 100      39.505  56.340  33.810  1.0   1.78      PROT H
HETATM  728  H21 MK1 C 100      40.807  54.974  32.244  1.0   3.27      PROT H
HETATM  729  H51 MK1 C 100      42.864  52.230  31.470  1.0   1.66      PROT H
HETATM  730  H52 MK1 C 100      41.986  53.525  30.657  1.0   1.60      PROT H
HETATM  731  H53 MK1 C 100      41.195  52.557  31.915  1.0   1.64      PROT H
HETATM  732  H61 MK1 C 100      43.533  52.442  33.925  1.0   1.57      PROT H
HETATM  733  H62 MK1 C 100      41.908  52.895  34.437  1.0   1.54      PROT H
HETATM  734  H63 MK1 C 100      43.270  53.966  34.794  1.0   1.64      PROT H
HETATM  735  H71 MK1 C 100      44.015  55.100  31.355  1.0   1.57      PROT H
HETATM  736  H72 MK1 C 100      44.739  53.693  32.156  1.0   1.56      PROT H
HETATM  737  H73 MK1 C 100      44.458  55.196  33.059  1.0   1.62      PROT H
HETATM  738  H81 MK1 C 100      39.661  59.573  35.980  1.0   1.79      PROT H
HETATM  739  H82 MK1 C 100      38.410  58.520  35.231  1.0   1.48      PROT H
HETATM  740  H91 MK1 C 100      38.615  60.554  33.829  1.0   1.82      PROT H
HETATM  741  H92 MK1 C 100      40.413  60.417  33.813  1.0   2.03      PROT H
HETATM  742 H101 MK1 C 100      40.534  55.989  36.075  1.0   1.52      PROT H
HETATM  743 H102 MK1 C 100      39.252  57.044  36.649  1.0   1.59      PROT H
HETATM  744  H15 MK1 C 100      41.466  58.659  37.214  1.0   1.57      PROT H
HETATM  745  H25 MK1 C 100      40.996  57.817  39.405  1.0   3.76      PROT H
HETATM  746 H121 MK1 C 100      42.977  56.715  36.488  1.0   2.02      PROT H
HETATM  747 H122 MK1 C 100      42.340  55.746  37.828  1.0   1.58      PROT H
HETATM  748  H13 MK1 C 100      43.010  57.591  39.469  1.0   1.83      PROT H
HETATM  749 H141 MK1 C 100      45.476  56.618  37.867  1.0   1.60      PROT H
HETATM  750 H142 MK1 C 100      44.471  55.506  38.822  1.0   1.55      PROT H
HETATM  751  H16 MK1 C 100      44.045  56.113  41.376  1.0   1.61      PROT H
HETATM  752  H17 MK1 C 100      45.554  56.106  43.382  1.0   1.54      PROT H
HETATM  753  H18 MK1 C 100      47.898  57.008  43.162  1.0   1.41      PROT H
HETATM  754  H19 MK1 C 100      48.659  57.977  41.001  1.0   1.51      PROT H
HETATM  755  H20 MK1 C 100      47.136  58.044  39.022  1.0   1.60      PROT H
HETATM  756  H4  MK1 C 100      44.523  59.480  39.879  1.0   3.35      PROT H
HETATM  757  H22 MK1 C 100      44.880  61.187  37.428  1.0   1.91      PROT H
HETATM  758  H23 MK1 C 100      47.026  61.256  38.486  1.0   1.45      PROT H
HETATM  759  H30 MK1 C 100      45.888  60.972  41.069  1.0   3.35      PROT H
```

```
HETATM  760 H241 MK1 C 100      46.501  63.615  38.672  1.0   1.62      PROT H
HETATM  761 H242 MK1 C 100      46.595  63.251  40.402  1.0   1.91      PROT H
HETATM  762  H26 MK1 C 100      44.525  65.332  40.234  1.0   1.68      PROT H
HETATM  763  H27 MK1 C 100      42.010  65.372  40.267  1.0   1.63      PROT H
HETATM  764  H28 MK1 C 100      40.706  63.329  39.677  1.0   1.60      PROT H
HETATM  765  H29 MK1 C 100      41.895  61.398  38.592  1.0   1.41      PROT H
HETATM  766 H311 MK1 C 100      40.745  60.304  31.403  1.0   2.24      PROT H
HETATM  767 H312 MK1 C 100      39.045  60.879  31.498  1.0   2.08      PROT H
HETATM  768  H33 MK1 C 100      41.434  59.248  29.381  1.0   2.01      PROT H
HETATM  769  H34 MK1 C 100      38.961  57.081  26.841  1.0   1.93      PROT H
HETATM  770  H35 MK1 C 100      36.938  57.809  28.191  1.0   1.79      PROT H
HETATM  771  H36 MK1 C 100      37.207  59.086  30.276  1.0   1.74      PROT H
HETATM  772  OW  SOL D8286      39.430  62.560  32.840  1.0  -7.53      PROT O
HETATM  773  HW1 SOL D8286      39.173  63.453  32.591  1.0   3.79      PROT H
HETATM  774  HW2 SOL D8286      40.213  62.697  33.378  1.0   3.54      PROT H
HETATM  775  OW  SOL D 774      45.210  57.710  35.100  1.0  -7.86      PROT O
HETATM  776  HW1 SOL D 774      44.585  57.090  34.719  1.0   3.66      PROT H
HETATM  777  HW2 SOL D 774      44.683  58.264  35.695  1.0   3.93      PROT H
END
```

4.4 NEL-HIV^{Pro}

```
HEADER  data-set: HIV_NEL_full_wH2O_OPT
REMARK  MOPAC, Version: 23.1.2
REMARK  99
REMARK  99 MOE v2014.09 (Chemical Computing Group Inc.)
ATOM      1  C   ACE A   7      20.375  16.843  14.387  1.0   4.46      PROT C
ATOM      2  O   ACE A   7      20.713  17.850  13.731  1.0  -6.23      PROT O
ATOM      3  HC  ACE A   7      20.896  15.882  14.179  1.0   1.49      PROT H
ATOM      4  N   ARG A   8      19.465  16.843  15.296  1.0  -5.04      PROT N
ATOM      5  CA  ARG A   8      18.728  18.052  15.651  1.0  -0.09      PROT C
ATOM      6  C   ARG A   8      17.742  17.688  16.751  1.0   5.60      PROT C
ATOM      7  O   ARG A   8      17.003  16.710  16.628  1.0  -6.48      PROT O
ATOM      8  CB  ARG A   8      17.967  18.611  14.449  1.0  -2.92      PROT C
ATOM      9  CG  ARG A   8      18.862  19.247  13.386  1.0  -2.85      PROT C
ATOM     10  CD  ARG A   8      18.057  19.913  12.267  1.0  -0.90      PROT C
ATOM     11  NE  ARG A   8      18.948  20.656  11.375  1.0  -5.24      PROT N
ATOM     12  CZ  ARG A   8      18.569  21.504  10.419  1.0   6.10      PROT C
```

ATOM	13	NH1	ARG	A	8	17.286	21.750	10.175	1.0	-6.18	PROT	N
ATOM	14	NH2	ARG	A	8	19.491	22.116	9.681	1.0	-6.25	PROT	N
ATOM	15	H	ARG	A	8	19.131	15.981	15.742	1.0	3.37	PROT	H
ATOM	16	HA	ARG	A	8	19.480	18.828	15.997	1.0	1.93	PROT	H
ATOM	17	HB2	ARG	A	8	17.353	17.805	13.991	1.0	1.60	PROT	H
ATOM	18	HB3	ARG	A	8	17.226	19.362	14.803	1.0	1.52	PROT	H
ATOM	19	HG2	ARG	A	8	19.430	18.466	12.818	1.0	2.08	PROT	H
ATOM	20	HG3	ARG	A	8	19.608	19.935	13.833	1.0	1.90	PROT	H
ATOM	21	HD2	ARG	A	8	17.284	20.593	12.704	1.0	1.41	PROT	H
ATOM	22	HD3	ARG	A	8	17.507	19.140	11.677	1.0	1.52	PROT	H
ATOM	23	HE	ARG	A	8	19.967	20.706	11.706	1.0	4.04	PROT	H
ATOM	24	HH11	ARG	A	8	16.984	22.211	9.324	1.0	3.39	PROT	H
ATOM	25	HH12	ARG	A	8	16.546	21.240	10.641	1.0	3.36	PROT	H
ATOM	26	HH21	ARG	A	8	20.515	21.908	9.855	1.0	3.97	PROT	H
ATOM	27	HH22	ARG	A	8	19.275	22.631	8.842	1.0	3.46	PROT	H
ATOM	28	N	NME	A	9	17.745	18.476	17.804	1.0	-6.01	PROT	N
ATOM	29	H1	NME	A	9	17.126	18.316	18.590	1.0	3.25	PROT	H
ATOM	30	H2	NME	A	9	18.378	19.255	17.933	1.0	3.34	PROT	H
ATOM	31	C	ACE	A	10	11.867	22.121	15.817	1.0	4.99	PROT	C
ATOM	32	O	ACE	A	10	12.565	21.475	15.007	1.0	-6.93	PROT	O
ATOM	33	HC	ACE	A	10	10.792	22.272	15.574	1.0	1.52	PROT	H
ATOM	34	N	LEU	A	23	12.312	22.630	16.911	1.0	-4.88	PROT	N
ATOM	35	CA	LEU	A	23	13.708	22.494	17.314	1.0	-0.18	PROT	C
ATOM	36	C	LEU	A	23	13.962	23.188	18.644	1.0	5.81	PROT	C
ATOM	37	O	LEU	A	23	13.700	24.385	18.769	1.0	-6.54	PROT	O
ATOM	38	CB	LEU	A	23	14.579	23.167	16.249	1.0	-3.47	PROT	C
ATOM	39	CG	LEU	A	23	16.101	23.087	16.316	1.0	-0.56	PROT	C
ATOM	40	CD1	LEU	A	23	16.568	21.668	16.184	1.0	-4.57	PROT	C
ATOM	41	CD2	LEU	A	23	16.702	23.945	15.199	1.0	-4.54	PROT	C
ATOM	42	H	LEU	A	23	11.788	23.365	17.407	1.0	3.41	PROT	H
ATOM	43	HA	LEU	A	23	13.956	21.394	17.362	1.0	1.76	PROT	H
ATOM	44	HB2	LEU	A	23	14.272	22.760	15.250	1.0	1.93	PROT	H
ATOM	45	HB3	LEU	A	23	14.277	24.241	16.198	1.0	1.66	PROT	H
ATOM	46	HG	LEU	A	23	16.444	23.499	17.303	1.0	1.33	PROT	H
ATOM	47	HD11	LEU	A	23	16.208	21.205	15.254	1.0	1.50	PROT	H
ATOM	48	HD12	LEU	A	23	16.214	21.037	17.008	1.0	1.31	PROT	H
ATOM	49	HD13	LEU	A	23	17.667	21.602	16.179	1.0	1.61	PROT	H
ATOM	50	HD21	LEU	A	23	16.347	23.626	14.213	1.0	1.42	PROT	H

ATOM	51	HD22	LEU	A	23	17.800	23.872	15.181	1.0	1.68	PROT	H
ATOM	52	HD23	LEU	A	23	16.439	25.001	15.319	1.0	1.41	PROT	H
ATOM	53	N	LEU	A	24	14.500	22.465	19.622	1.0	-5.19	PROT	N
ATOM	54	CA	LEU	A	24	14.879	23.074	20.891	1.0	-0.12	PROT	C
ATOM	55	C	LEU	A	24	16.199	23.798	20.676	1.0	5.25	PROT	C
ATOM	56	O	LEU	A	24	17.228	23.151	20.463	1.0	-5.24	PROT	O
ATOM	57	CB	LEU	A	24	15.030	22.044	22.011	1.0	-3.22	PROT	C
ATOM	58	CG	LEU	A	24	13.807	21.157	22.246	1.0	-0.54	PROT	C
ATOM	59	CD1	LEU	A	24	14.155	20.109	23.278	1.0	-4.68	PROT	C
ATOM	60	CD2	LEU	A	24	12.578	21.966	22.654	1.0	-4.61	PROT	C
ATOM	61	H	LEU	A	24	14.762	21.488	19.503	1.0	3.34	PROT	H
ATOM	62	HA	LEU	A	24	14.057	23.799	21.187	1.0	1.83	PROT	H
ATOM	63	HB2	LEU	A	24	15.919	21.403	21.806	1.0	1.71	PROT	H
ATOM	64	HB3	LEU	A	24	15.288	22.580	22.951	1.0	1.68	PROT	H
ATOM	65	HG	LEU	A	24	13.558	20.629	21.285	1.0	1.34	PROT	H
ATOM	66	HD11	LEU	A	24	14.422	20.555	24.245	1.0	1.51	PROT	H
ATOM	67	HD12	LEU	A	24	15.003	19.487	22.965	1.0	1.49	PROT	H
ATOM	68	HD13	LEU	A	24	13.314	19.429	23.466	1.0	1.47	PROT	H
ATOM	69	HD21	LEU	A	24	12.764	22.562	23.556	1.0	1.52	PROT	H
ATOM	70	HD22	LEU	A	24	11.725	21.312	22.870	1.0	1.44	PROT	H
ATOM	71	HD23	LEU	A	24	12.262	22.654	21.861	1.0	1.49	PROT	H
ATOM	72	N	ASH	A	25	16.147	25.125	20.767	1.0	-5.43	PROT	N
ATOM	73	CA	ASH	A	25	17.211	25.997	20.286	1.0	-0.07	PROT	C
ATOM	74	C	ASH	A	25	17.712	26.980	21.350	1.0	5.56	PROT	C
ATOM	75	O	ASH	A	25	17.171	28.072	21.533	1.0	-5.50	PROT	O
ATOM	76	CB	ASH	A	25	16.707	26.754	19.056	1.0	-3.71	PROT	C
ATOM	77	CG	ASH	A	25	17.818	27.463	18.304	1.0	6.87	PROT	C
ATOM	78	OD1	ASH	A	25	17.533	27.943	17.189	1.0	-6.49	PROT	O
ATOM	79	OD2	ASH	A	25	18.960	27.541	18.809	1.0	-5.49	PROT	O
ATOM	80	H	ASH	A	25	15.249	25.601	20.913	1.0	3.40	PROT	H
ATOM	81	HA	ASH	A	25	18.084	25.334	19.962	1.0	2.01	PROT	H
ATOM	82	HB2	ASH	A	25	15.946	27.522	19.347	1.0	2.14	PROT	H
ATOM	83	HB3	ASH	A	25	16.182	26.067	18.355	1.0	1.95	PROT	H
ATOM	84	HD2	ASH	A	25	19.665	28.063	18.230	1.0	4.51	PROT	H
ATOM	85	N	THR	A	26	18.774	26.584	22.044	1.0	-5.59	PROT	N
ATOM	86	CA	THR	A	26	19.395	27.443	23.055	1.0	-0.73	PROT	C
ATOM	87	C	THR	A	26	20.037	28.705	22.476	1.0	5.82	PROT	C
ATOM	88	O	THR	A	26	20.295	29.669	23.199	1.0	-5.85	PROT	O

ATOM	89	CB	THR	A	26	20.445	26.654	23.853	1.0	1.41	PROT	C
ATOM	90	OG1	THR	A	26	21.457	26.146	22.972	1.0	-5.05	PROT	O
ATOM	91	CG2	THR	A	26	19.780	25.496	24.586	1.0	-5.28	PROT	C
ATOM	92	H	THR	A	26	19.078	25.623	21.989	1.0	3.12	PROT	H
ATOM	93	HA	THR	A	26	18.588	27.807	23.771	1.0	2.13	PROT	H
ATOM	94	HB	THR	A	26	21.037	27.322	24.526	1.0	1.88	PROT	H
ATOM	95	HG21	THR	A	26	19.261	24.785	23.935	1.0	1.60	PROT	H
ATOM	96	HG22	THR	A	26	19.043	25.855	25.319	1.0	1.79	PROT	H
ATOM	97	HG23	THR	A	26	20.524	24.916	25.151	1.0	1.70	PROT	H
ATOM	98	HG1	THR	A	26	21.013	25.547	22.334	1.0	2.62	PROT	H
ATOM	99	N	GLY	A	27	20.309	28.712	21.177	1.0	-5.20	PROT	N
ATOM	100	CA	GLY	A	27	20.862	29.896	20.521	1.0	-1.70	PROT	C
ATOM	101	C	GLY	A	27	19.858	30.915	20.001	1.0	5.86	PROT	C
ATOM	102	O	GLY	A	27	20.259	31.925	19.412	1.0	-6.53	PROT	O
ATOM	103	H	GLY	A	27	20.190	27.913	20.559	1.0	3.44	PROT	H
ATOM	104	HA2	GLY	A	27	21.551	30.435	21.232	1.0	2.01	PROT	H
ATOM	105	HA3	GLY	A	27	21.503	29.571	19.659	1.0	1.97	PROT	H
ATOM	106	N	ALA	A	28	18.569	30.655	20.194	1.0	-5.21	PROT	N
ATOM	107	CA	ALA	A	28	17.510	31.592	19.821	1.0	0.22	PROT	C
ATOM	108	C	ALA	A	28	16.936	32.267	21.063	1.0	5.62	PROT	C
ATOM	109	O	ALA	A	28	16.565	31.583	22.014	1.0	-5.86	PROT	O
ATOM	110	CB	ALA	A	28	16.401	30.861	19.093	1.0	-4.79	PROT	C
ATOM	111	H	ALA	A	28	18.258	29.844	20.739	1.0	3.47	PROT	H
ATOM	112	HA	ALA	A	28	17.960	32.364	19.119	1.0	1.82	PROT	H
ATOM	113	HB1	ALA	A	28	15.977	30.050	19.708	1.0	1.71	PROT	H
ATOM	114	HB2	ALA	A	28	16.755	30.406	18.159	1.0	1.60	PROT	H
ATOM	115	HB3	ALA	A	28	15.552	31.523	18.857	1.0	1.85	PROT	H
ATOM	116	N	ASP	A	29	16.846	33.595	21.048	1.0	-5.61	PROT	N
ATOM	117	CA	ASP	A	29	16.264	34.350	22.162	1.0	0.40	PROT	C
ATOM	118	C	ASP	A	29	14.745	34.183	22.155	1.0	5.84	PROT	C
ATOM	119	O	ASP	A	29	14.092	34.211	23.206	1.0	-6.46	PROT	O
ATOM	120	CB	ASP	A	29	16.566	35.848	22.055	1.0	-4.16	PROT	C
ATOM	121	CG	ASP	A	29	18.046	36.170	22.027	1.0	7.65	PROT	C
ATOM	122	OD1	ASP	A	29	18.883	35.363	22.478	1.0	-7.76	PROT	O
ATOM	123	OD2	ASP	A	29	18.371	37.284	21.571	1.0	-8.20	PROT	O
ATOM	124	H	ASP	A	29	17.331	34.157	20.346	1.0	3.30	PROT	H
ATOM	125	HA	ASP	A	29	16.671	33.945	23.137	1.0	1.99	PROT	H
ATOM	126	HB2	ASP	A	29	16.112	36.382	22.923	1.0	2.05	PROT	H

ATOM	127	HB3	ASP	A	29	16.066	36.305	21.168	1.0	2.04	PROT	H
ATOM	128	N	ASP	A	30	14.204	34.025	20.949	1.0	-4.99	PROT	N
ATOM	129	CA	ASP	A	30	12.765	34.010	20.696	1.0	0.24	PROT	C
ATOM	130	C	ASP	A	30	12.328	32.681	20.116	1.0	5.51	PROT	C
ATOM	131	O	ASP	A	30	13.151	31.907	19.612	1.0	-6.09	PROT	O
ATOM	132	CB	ASP	A	30	12.376	35.097	19.690	1.0	-4.43	PROT	C
ATOM	133	CG	ASP	A	30	13.036	36.425	19.993	1.0	7.60	PROT	C
ATOM	134	OD1	ASP	A	30	12.692	37.022	21.027	1.0	-8.39	PROT	O
ATOM	135	OD2	ASP	A	30	13.911	36.859	19.216	1.0	-7.73	PROT	O
ATOM	136	H	ASP	A	30	14.758	34.030	20.091	1.0	3.37	PROT	H
ATOM	137	HA	ASP	A	30	12.236	34.208	21.681	1.0	1.97	PROT	H
ATOM	138	HB2	ASP	A	30	11.278	35.244	19.681	1.0	1.67	PROT	H
ATOM	139	HB3	ASP	A	30	12.658	34.800	18.657	1.0	1.73	PROT	H
ATOM	140	N	THR	A	31	11.020	32.466	20.164	1.0	-5.24	PROT	N
ATOM	141	CA	THR	A	31	10.382	31.303	19.563	1.0	-0.67	PROT	C
ATOM	142	C	THR	A	31	9.828	31.740	18.211	1.0	5.61	PROT	C
ATOM	143	O	THR	A	31	9.107	32.732	18.134	1.0	-6.01	PROT	O
ATOM	144	CB	THR	A	31	9.296	30.761	20.516	1.0	1.79	PROT	C
ATOM	145	OG1	THR	A	31	9.942	30.180	21.655	1.0	-5.94	PROT	O
ATOM	146	CG2	THR	A	31	8.399	29.723	19.853	1.0	-5.29	PROT	C
ATOM	147	H	THR	A	31	10.364	33.165	20.524	1.0	3.41	PROT	H
ATOM	148	HA	THR	A	31	11.162	30.488	19.420	1.0	2.04	PROT	H
ATOM	149	HB	THR	A	31	8.701	31.582	20.983	1.0	1.61	PROT	H
ATOM	150	HG21	THR	A	31	8.980	28.944	19.337	1.0	1.74	PROT	H
ATOM	151	HG22	THR	A	31	7.732	30.175	19.109	1.0	1.67	PROT	H
ATOM	152	HG23	THR	A	31	7.770	29.218	20.594	1.0	1.60	PROT	H
ATOM	153	HG1	THR	A	31	10.493	29.419	21.377	1.0	3.36	PROT	H
ATOM	154	N	VAL	A	32	10.197	31.025	17.150	1.0	-5.43	PROT	N
ATOM	155	CA	VAL	A	32	9.818	31.397	15.792	1.0	-0.39	PROT	C
ATOM	156	C	VAL	A	32	9.182	30.215	15.070	1.0	5.88	PROT	C
ATOM	157	O	VAL	A	32	9.836	29.207	14.805	1.0	-6.74	PROT	O
ATOM	158	CB	VAL	A	32	11.027	31.883	14.960	1.0	-0.71	PROT	C
ATOM	159	CG1	VAL	A	32	10.555	32.566	13.683	1.0	-4.64	PROT	C
ATOM	160	CG2	VAL	A	32	11.899	32.829	15.777	1.0	-4.48	PROT	C
ATOM	161	H	VAL	A	32	10.686	30.125	17.247	1.0	3.53	PROT	H
ATOM	162	HA	VAL	A	32	9.078	32.261	15.862	1.0	2.04	PROT	H
ATOM	163	HB	VAL	A	32	11.639	30.983	14.679	1.0	1.51	PROT	H
ATOM	164	HG11	VAL	A	32	9.940	33.450	13.890	1.0	1.50	PROT	H

ATOM	165	HG12	VAL	A	32	9.956	31.894	13.057	1.0	1.43	PROT	H
ATOM	166	HG13	VAL	A	32	11.401	32.895	13.065	1.0	1.56	PROT	H
ATOM	167	HG21	VAL	A	32	11.311	33.665	16.183	1.0	1.61	PROT	H
ATOM	168	HG22	VAL	A	32	12.701	33.252	15.162	1.0	1.44	PROT	H
ATOM	169	HG23	VAL	A	32	12.358	32.315	16.633	1.0	1.54	PROT	H
ATOM	170	N	NME	A	33	7.911	30.352	14.759	1.0	-6.12	PROT	N
ATOM	171	H1	NME	A	33	7.411	29.619	14.273	1.0	3.16	PROT	H
ATOM	172	H2	NME	A	33	7.394	31.199	14.922	1.0	3.46	PROT	H
ATOM	173	C	ACE	A	34	12.591	40.515	13.614	1.0	4.43	PROT	C
ATOM	174	O	ACE	A	34	12.270	40.388	14.814	1.0	-6.38	PROT	O
ATOM	175	HC	ACE	A	34	11.911	41.086	12.946	1.0	1.33	PROT	H
ATOM	176	N	ILE	A	47	13.656	40.016	13.093	1.0	-4.93	PROT	N
ATOM	177	CA	ILE	A	47	14.602	39.242	13.874	1.0	-0.50	PROT	C
ATOM	178	C	ILE	A	47	15.746	38.797	12.972	1.0	5.56	PROT	C
ATOM	179	O	ILE	A	47	15.539	38.467	11.799	1.0	-6.32	PROT	O
ATOM	180	CB	ILE	A	47	13.934	38.024	14.551	1.0	-0.82	PROT	C
ATOM	181	CG1	ILE	A	47	13.282	37.109	13.513	1.0	-2.80	PROT	C
ATOM	182	CG2	ILE	A	47	12.940	38.499	15.605	1.0	-4.22	PROT	C
ATOM	183	CD1	ILE	A	47	12.756	35.809	14.098	1.0	-4.32	PROT	C
ATOM	184	H	ILE	A	47	13.841	40.038	12.083	1.0	3.37	PROT	H
ATOM	185	HA	ILE	A	47	14.997	39.914	14.700	1.0	1.83	PROT	H
ATOM	186	HB	ILE	A	47	14.745	37.444	15.071	1.0	1.53	PROT	H
ATOM	187	HG12	ILE	A	47	12.456	37.647	13.006	1.0	1.34	PROT	H
ATOM	188	HG13	ILE	A	47	14.010	36.879	12.706	1.0	1.45	PROT	H
ATOM	189	HG21	ILE	A	47	11.908	38.558	15.245	1.0	1.56	PROT	H
ATOM	190	HG22	ILE	A	47	13.267	39.396	16.143	1.0	1.62	PROT	H
ATOM	191	HG23	ILE	A	47	12.887	37.731	16.409	1.0	1.55	PROT	H
ATOM	192	HD11	ILE	A	47	11.971	35.993	14.844	1.0	1.55	PROT	H
ATOM	193	HD12	ILE	A	47	13.556	35.251	14.601	1.0	1.46	PROT	H
ATOM	194	HD13	ILE	A	47	12.329	35.167	13.320	1.0	1.34	PROT	H
ATOM	195	N	GLY	A	48	16.947	38.811	13.537	1.0	-5.02	PROT	N
ATOM	196	CA	GLY	A	48	18.156	38.537	12.786	1.0	-1.82	PROT	C
ATOM	197	C	GLY	A	48	18.730	37.188	13.156	1.0	5.63	PROT	C
ATOM	198	O	GLY	A	48	18.823	36.841	14.331	1.0	-5.65	PROT	O
ATOM	199	H	GLY	A	48	17.082	38.930	14.545	1.0	3.49	PROT	H
ATOM	200	HA2	GLY	A	48	17.956	38.630	11.686	1.0	1.89	PROT	H
ATOM	201	HA3	GLY	A	48	18.925	39.322	13.012	1.0	1.81	PROT	H
ATOM	202	N	GLY	A	49	19.121	36.435	12.135	1.0	-5.19	PROT	N

ATOM	203	CA	GLY	A	49	19.897	35.219	12.323	1.0	-1.79	PROT	C
ATOM	204	C	GLY	A	49	21.107	35.296	11.416	1.0	5.66	PROT	C
ATOM	205	O	GLY	A	49	21.453	36.366	10.913	1.0	-6.30	PROT	O
ATOM	206	H	GLY	A	49	19.057	36.746	11.165	1.0	3.35	PROT	H
ATOM	207	HA2	GLY	A	49	20.198	35.102	13.400	1.0	2.06	PROT	H
ATOM	208	HA3	GLY	A	49	19.260	34.321	12.104	1.0	1.92	PROT	H
ATOM	209	N	ILE	A	50	21.774	34.160	11.257	1.0	-5.26	PROT	N
ATOM	210	CA	ILE	A	50	22.770	34.021	10.207	1.0	-0.23	PROT	C
ATOM	211	C	ILE	A	50	22.054	34.331	8.903	1.0	5.77	PROT	C
ATOM	212	O	ILE	A	50	20.905	33.941	8.696	1.0	-6.55	PROT	O
ATOM	213	CB	ILE	A	50	23.393	32.608	10.190	1.0	-0.93	PROT	C
ATOM	214	CG1	ILE	A	50	24.425	32.483	11.314	1.0	-2.56	PROT	C
ATOM	215	CG2	ILE	A	50	24.047	32.286	8.845	1.0	-4.66	PROT	C
ATOM	216	CD1	ILE	A	50	25.613	33.420	11.208	1.0	-4.45	PROT	C
ATOM	217	H	ILE	A	50	21.369	33.277	11.605	1.0	3.43	PROT	H
ATOM	218	HA	ILE	A	50	23.585	34.783	10.396	1.0	1.80	PROT	H
ATOM	219	HB	ILE	A	50	22.579	31.856	10.369	1.0	1.46	PROT	H
ATOM	220	HG12	ILE	A	50	23.920	32.650	12.289	1.0	1.55	PROT	H
ATOM	221	HG13	ILE	A	50	24.786	31.433	11.356	1.0	1.44	PROT	H
ATOM	222	HG21	ILE	A	50	24.801	33.028	8.560	1.0	1.55	PROT	H
ATOM	223	HG22	ILE	A	50	23.315	32.223	8.031	1.0	1.50	PROT	H
ATOM	224	HG23	ILE	A	50	24.557	31.314	8.881	1.0	1.60	PROT	H
ATOM	225	HD11	ILE	A	50	25.322	34.474	11.175	1.0	1.28	PROT	H
ATOM	226	HD12	ILE	A	50	26.232	33.219	10.327	1.0	1.44	PROT	H
ATOM	227	HD13	ILE	A	50	26.266	33.303	12.091	1.0	1.77	PROT	H
ATOM	228	N	NME	A	51	22.740	35.034	8.028	1.0	-6.13	PROT	N
ATOM	229	H1	NME	A	51	22.343	35.314	7.140	1.0	3.23	PROT	H
ATOM	230	H2	NME	A	51	23.664	35.398	8.198	1.0	3.25	PROT	H
ATOM	231	C	ACE	A	52	6.784	34.725	18.740	1.0	4.25	PROT	C
ATOM	232	O	ACE	A	52	7.143	35.909	18.912	1.0	-6.06	PROT	O
ATOM	233	HC	ACE	A	52	6.611	34.080	19.631	1.0	1.37	PROT	H
ATOM	234	N	LEU	A	76	6.616	34.180	17.587	1.0	-4.92	PROT	N
ATOM	235	CA	LEU	A	76	6.834	34.915	16.348	1.0	-0.25	PROT	C
ATOM	236	C	LEU	A	76	6.554	34.001	15.158	1.0	5.88	PROT	C
ATOM	237	O	LEU	A	76	6.994	32.849	15.137	1.0	-6.79	PROT	O
ATOM	238	CB	LEU	A	76	8.278	35.417	16.277	1.0	-3.13	PROT	C
ATOM	239	CG	LEU	A	76	8.813	36.124	17.526	1.0	-0.12	PROT	C
ATOM	240	CD1	LEU	A	76	10.263	36.554	17.309	1.0	-4.75	PROT	C

ATOM	241	CD2	LEU	A	76	7.944	37.326	17.899	1.0	-3.76	PROT	C
ATOM	242	H	LEU	A	76	6.636	33.157	17.472	1.0	3.37	PROT	H
ATOM	243	HA	LEU	A	76	6.133	35.802	16.348	1.0	1.69	PROT	H
ATOM	244	HB2	LEU	A	76	8.944	34.545	16.071	1.0	1.63	PROT	H
ATOM	245	HB3	LEU	A	76	8.383	36.091	15.402	1.0	1.41	PROT	H
ATOM	246	HG	LEU	A	76	8.927	35.367	18.352	1.0	1.60	PROT	H
ATOM	247	HD11	LEU	A	76	10.372	37.249	16.469	1.0	1.38	PROT	H
ATOM	248	HD12	LEU	A	76	10.914	35.695	17.110	1.0	1.41	PROT	H
ATOM	249	HD13	LEU	A	76	10.657	37.064	18.199	1.0	1.65	PROT	H
ATOM	250	HD21	LEU	A	76	8.338	38.224	17.377	1.0	1.03	PROT	H
ATOM	251	HD22	LEU	A	76	8.011	37.611	18.962	1.0	1.49	PROT	H
ATOM	252	HD23	LEU	A	76	6.904	37.297	17.556	1.0	1.22	PROT	H
ATOM	253	N	NME	A	77	5.831	34.525	14.193	1.0	-6.01	PROT	N
ATOM	254	H1	NME	A	77	5.606	34.007	13.353	1.0	3.22	PROT	H
ATOM	255	H2	NME	A	77	5.528	35.488	14.176	1.0	3.31	PROT	H
ATOM	256	C	ACE	A	78	10.122	32.122	8.629	1.0	4.57	PROT	C
ATOM	257	O	ACE	A	78	10.943	32.731	9.347	1.0	-6.55	PROT	O
ATOM	258	HC	ACE	A	78	9.248	32.688	8.240	1.0	1.47	PROT	H
ATOM	259	N	THR	A	80	10.227	30.883	8.301	1.0	-4.89	PROT	N
ATOM	260	CA	THR	A	80	11.342	30.058	8.750	1.0	-0.67	PROT	C
ATOM	261	C	THR	A	80	11.152	28.652	8.181	1.0	5.72	PROT	C
ATOM	262	O	THR	A	80	10.022	28.179	8.088	1.0	-6.30	PROT	O
ATOM	263	CB	THR	A	80	11.435	30.009	10.291	1.0	1.93	PROT	C
ATOM	264	OG1	THR	A	80	12.447	29.075	10.692	1.0	-6.57	PROT	O
ATOM	265	CG2	THR	A	80	10.101	29.615	10.941	1.0	-5.14	PROT	C
ATOM	266	H	THR	A	80	9.483	30.369	7.808	1.0	3.47	PROT	H
ATOM	267	HA	THR	A	80	12.298	30.524	8.362	1.0	1.89	PROT	H
ATOM	268	HB	THR	A	80	11.812	30.991	10.673	1.0	1.71	PROT	H
ATOM	269	HG21	THR	A	80	9.670	28.716	10.483	1.0	1.67	PROT	H
ATOM	270	HG22	THR	A	80	9.360	30.416	10.870	1.0	1.58	PROT	H
ATOM	271	HG23	THR	A	80	10.240	29.390	12.013	1.0	1.87	PROT	H
ATOM	272	HG1	THR	A	80	12.102	28.421	11.343	1.0	3.80	PROT	H
ATOM	273	N	PRO	A	81	12.247	27.968	7.813	1.0	-4.75	PROT	N
ATOM	274	CA	PRO	A	81	12.128	26.586	7.347	1.0	0.01	PROT	C
ATOM	275	C	PRO	A	81	11.878	25.572	8.463	1.0	5.71	PROT	C
ATOM	276	O	PRO	A	81	11.557	24.415	8.175	1.0	-6.43	PROT	O
ATOM	277	CB	PRO	A	81	13.495	26.314	6.715	1.0	-2.91	PROT	C
ATOM	278	CG	PRO	A	81	14.428	27.180	7.464	1.0	-2.99	PROT	C

ATOM	279	CD	PRO	A	81	13.649	28.425	7.810	1.0	-0.54	PROT C
ATOM	280	HA	PRO	A	81	11.282	26.493	6.610	1.0	1.93	PROT H
ATOM	281	HB2	PRO	A	81	13.761	25.237	6.749	1.0	1.66	PROT H
ATOM	282	HB3	PRO	A	81	13.471	26.552	5.628	1.0	1.69	PROT H
ATOM	283	HG2	PRO	A	81	15.345	27.420	6.881	1.0	1.71	PROT H
ATOM	284	HG3	PRO	A	81	14.810	26.674	8.380	1.0	1.51	PROT H
ATOM	285	HD2	PRO	A	81	13.782	29.234	7.053	1.0	1.64	PROT H
ATOM	286	HD3	PRO	A	81	13.919	28.844	8.810	1.0	1.69	PROT H
ATOM	287	N	VAL	A	82	12.068	25.997	9.712	1.0	-5.33	PROT N
ATOM	288	CA	VAL	A	82	11.973	25.116	10.865	1.0	-0.34	PROT C
ATOM	289	C	VAL	A	82	11.287	25.883	11.995	1.0	5.74	PROT C
ATOM	290	O	VAL	A	82	11.539	27.074	12.181	1.0	-6.11	PROT O
ATOM	291	CB	VAL	A	82	13.378	24.664	11.329	1.0	-0.69	PROT C
ATOM	292	CG1	VAL	A	82	13.289	23.720	12.514	1.0	-4.79	PROT C
ATOM	293	CG2	VAL	A	82	14.141	24.005	10.185	1.0	-4.50	PROT C
ATOM	294	H	VAL	A	82	12.313	26.972	9.927	1.0	3.53	PROT H
ATOM	295	HA	VAL	A	82	11.377	24.199	10.577	1.0	1.93	PROT H
ATOM	296	HB	VAL	A	82	13.944	25.582	11.647	1.0	1.45	PROT H
ATOM	297	HG11	VAL	A	82	12.691	22.826	12.300	1.0	1.55	PROT H
ATOM	298	HG12	VAL	A	82	12.847	24.190	13.407	1.0	1.76	PROT H
ATOM	299	HG13	VAL	A	82	14.279	23.362	12.822	1.0	1.52	PROT H
ATOM	300	HG21	VAL	A	82	13.580	23.162	9.759	1.0	1.59	PROT H
ATOM	301	HG22	VAL	A	82	15.109	23.621	10.523	1.0	1.40	PROT H
ATOM	302	HG23	VAL	A	82	14.333	24.702	9.361	1.0	1.44	PROT H
ATOM	303	N	ASN	A	83	10.414	25.206	12.735	1.0	-5.18	PROT N
ATOM	304	CA	ASN	A	83	9.838	25.777	13.950	1.0	0.28	PROT C
ATOM	305	C	ASN	A	83	10.923	25.760	15.033	1.0	5.59	PROT C
ATOM	306	O	ASN	A	83	11.534	24.717	15.273	1.0	-6.43	PROT O
ATOM	307	CB	ASN	A	83	8.631	24.968	14.432	1.0	-3.93	PROT C
ATOM	308	CG	ASN	A	83	7.459	24.993	13.468	1.0	6.17	PROT C
ATOM	309	OD1	ASN	A	83	6.918	26.051	13.154	1.0	-6.29	PROT O
ATOM	310	ND2	ASN	A	83	7.032	23.815	13.026	1.0	-6.35	PROT N
ATOM	311	H	ASN	A	83	10.250	24.210	12.597	1.0	3.33	PROT H
ATOM	312	HA	ASN	A	83	9.511	26.845	13.722	1.0	2.17	PROT H
ATOM	313	HB2	ASN	A	83	8.272	25.400	15.400	1.0	2.04	PROT H
ATOM	314	HB3	ASN	A	83	8.942	23.933	14.692	1.0	1.78	PROT H
ATOM	315	HD21	ASN	A	83	6.237	23.743	12.408	1.0	3.20	PROT H
ATOM	316	HD22	ASN	A	83	7.443	22.935	13.289	1.0	3.26	PROT H

ATOM	317	N	ILE	A	84	11.125	26.903	15.685	1.0	-5.26	PROT	N
ATOM	318	CA	ILE	A	84	12.217	27.137	16.621	1.0	-0.40	PROT	C
ATOM	319	C	ILE	A	84	11.674	27.451	18.010	1.0	5.86	PROT	C
ATOM	320	O	ILE	A	84	10.974	28.449	18.183	1.0	-6.58	PROT	O
ATOM	321	CB	ILE	A	84	13.054	28.364	16.210	1.0	-0.95	PROT	C
ATOM	322	CG1	ILE	A	84	13.662	28.193	14.815	1.0	-2.65	PROT	C
ATOM	323	CG2	ILE	A	84	14.154	28.630	17.217	1.0	-4.56	PROT	C
ATOM	324	CD1	ILE	A	84	14.701	27.107	14.703	1.0	-4.46	PROT	C
ATOM	325	H	ILE	A	84	10.624	27.779	15.441	1.0	3.74	PROT	H
ATOM	326	HA	ILE	A	84	12.880	26.222	16.642	1.0	1.77	PROT	H
ATOM	327	HB	ILE	A	84	12.365	29.254	16.172	1.0	1.59	PROT	H
ATOM	328	HG12	ILE	A	84	12.845	28.004	14.078	1.0	1.60	PROT	H
ATOM	329	HG13	ILE	A	84	14.097	29.164	14.494	1.0	1.43	PROT	H
ATOM	330	HG21	ILE	A	84	14.792	27.753	17.377	1.0	1.47	PROT	H
ATOM	331	HG22	ILE	A	84	13.759	28.946	18.193	1.0	1.60	PROT	H
ATOM	332	HG23	ILE	A	84	14.820	29.437	16.877	1.0	1.64	PROT	H
ATOM	333	HD11	ILE	A	84	14.309	26.135	15.035	1.0	1.41	PROT	H
ATOM	334	HD12	ILE	A	84	15.592	27.325	15.309	1.0	1.56	PROT	H
ATOM	335	HD13	ILE	A	84	15.014	26.968	13.661	1.0	1.28	PROT	H
ATOM	336	N	NME	A	85	12.005	26.602	18.958	1.0	-6.15	PROT	N
ATOM	337	H1	NME	A	85	11.726	26.738	19.919	1.0	3.23	PROT	H
ATOM	338	H2	NME	A	85	12.633	25.828	18.789	1.0	3.43	PROT	H
TER	339		NME	A	85							
ATOM	340	C	ACE	B	7	20.590	35.113	27.352	1.0	4.46	PROT	C
ATOM	341	O	ACE	B	7	20.157	35.335	26.202	1.0	-6.27	PROT	O
ATOM	342	HC	ACE	B	7	20.111	35.642	28.205	1.0	1.46	PROT	H
ATOM	343	N	ARG	B	8	21.559	34.310	27.619	1.0	-5.02	PROT	N
ATOM	344	CA	ARG	B	8	22.258	33.567	26.572	1.0	-0.12	PROT	C
ATOM	345	C	ARG	B	8	23.365	32.689	27.172	1.0	5.57	PROT	C
ATOM	346	O	ARG	B	8	24.168	33.175	27.978	1.0	-6.49	PROT	O
ATOM	347	CB	ARG	B	8	22.868	34.530	25.555	1.0	-2.92	PROT	C
ATOM	348	CG	ARG	B	8	21.853	35.272	24.723	1.0	-2.74	PROT	C
ATOM	349	CD	ARG	B	8	22.524	35.953	23.541	1.0	-1.02	PROT	C
ATOM	350	NE	ARG	B	8	21.651	36.779	22.783	1.0	-4.66	PROT	N
ATOM	351	CZ	ARG	B	8	21.590	36.982	21.467	1.0	6.39	PROT	C
ATOM	352	NH1	ARG	B	8	22.354	36.287	20.626	1.0	-6.42	PROT	N
ATOM	353	NH2	ARG	B	8	20.762	37.898	20.988	1.0	-7.12	PROT	N
ATOM	354	H	ARG	B	8	21.956	34.216	28.560	1.0	3.35	PROT	H

ATOM	355	HA	ARG	B	8	21.486	32.925	26.041	1.0	1.89	PROT	H
ATOM	356	HB2	ARG	B	8	23.535	35.248	26.084	1.0	1.59	PROT	H
ATOM	357	HB3	ARG	B	8	23.555	33.955	24.890	1.0	1.55	PROT	H
ATOM	358	HG2	ARG	B	8	21.427	36.139	25.294	1.0	1.87	PROT	H
ATOM	359	HG3	ARG	B	8	21.017	34.624	24.381	1.0	1.95	PROT	H
ATOM	360	HD2	ARG	B	8	23.012	35.171	22.896	1.0	1.55	PROT	H
ATOM	361	HD3	ARG	B	8	23.378	36.592	23.907	1.0	1.54	PROT	H
ATOM	362	HE	ARG	B	8	20.809	37.091	23.318	1.0	3.55	PROT	H
ATOM	363	HH11	ARG	B	8	22.358	36.512	19.639	1.0	3.21	PROT	H
ATOM	364	HH12	ARG	B	8	22.547	35.299	20.832	1.0	3.58	PROT	H
ATOM	365	HH21	ARG	B	8	19.702	37.827	21.367	1.0	4.23	PROT	H
ATOM	366	HH22	ARG	B	8	20.780	38.134	20.000	1.0	3.25	PROT	H
ATOM	367	N	NME	B	9	23.381	31.436	26.771	1.0	-5.99	PROT	N
ATOM	368	H1	NME	B	9	24.073	30.778	27.106	1.0	3.25	PROT	H
ATOM	369	H2	NME	B	9	22.708	31.037	26.130	1.0	3.34	PROT	H
ATOM	370	C	ACE	B	10	28.980	32.348	22.064	1.0	5.03	PROT	C
ATOM	371	O	ACE	B	10	28.294	33.301	22.488	1.0	-6.96	PROT	O
ATOM	372	HC	ACE	B	10	30.020	32.562	21.734	1.0	1.45	PROT	H
ATOM	373	N	LEU	B	23	28.561	31.135	21.981	1.0	-4.87	PROT	N
ATOM	374	CA	LEU	B	23	27.214	30.756	22.385	1.0	-0.26	PROT	C
ATOM	375	C	LEU	B	23	27.009	29.263	22.173	1.0	5.83	PROT	C
ATOM	376	O	LEU	B	23	27.198	28.764	21.062	1.0	-6.41	PROT	O
ATOM	377	CB	LEU	B	23	26.196	31.536	21.556	1.0	-3.43	PROT	C
ATOM	378	CG	LEU	B	23	24.701	31.262	21.707	1.0	-0.58	PROT	C
ATOM	379	CD1	LEU	B	23	24.163	31.881	22.992	1.0	-4.55	PROT	C
ATOM	380	CD2	LEU	B	23	23.986	31.817	20.487	1.0	-4.46	PROT	C
ATOM	381	H	LEU	B	23	29.040	30.448	21.381	1.0	3.39	PROT	H
ATOM	382	HA	LEU	B	23	27.086	31.037	23.473	1.0	1.70	PROT	H
ATOM	383	HB2	LEU	B	23	26.367	32.627	21.738	1.0	1.90	PROT	H
ATOM	384	HB3	LEU	B	23	26.478	31.405	20.480	1.0	1.78	PROT	H
ATOM	385	HG	LEU	B	23	24.530	30.155	21.751	1.0	1.32	PROT	H
ATOM	386	HD11	LEU	B	23	24.272	32.972	22.987	1.0	1.47	PROT	H
ATOM	387	HD12	LEU	B	23	24.689	31.502	23.872	1.0	1.24	PROT	H
ATOM	388	HD13	LEU	B	23	23.095	31.654	23.120	1.0	1.62	PROT	H
ATOM	389	HD21	LEU	B	23	24.288	32.849	20.272	1.0	1.44	PROT	H
ATOM	390	HD22	LEU	B	23	22.894	31.835	20.621	1.0	1.66	PROT	H
ATOM	391	HD23	LEU	B	23	24.197	31.222	19.589	1.0	1.47	PROT	H
ATOM	392	N	LEU	B	24	26.618	28.576	23.244	1.0	-5.26	PROT	N

ATOM	393	CA	LEU	B	24	26.237	27.168	23.178	1.0	-0.07	PROT	C
ATOM	394	C	LEU	B	24	24.862	27.027	22.523	1.0	5.24	PROT	C
ATOM	395	O	LEU	B	24	23.822	27.373	23.091	1.0	-5.42	PROT	O
ATOM	396	CB	LEU	B	24	26.267	26.535	24.570	1.0	-3.24	PROT	C
ATOM	397	CG	LEU	B	24	27.581	26.629	25.350	1.0	-0.52	PROT	C
ATOM	398	CD1	LEU	B	24	27.434	25.939	26.692	1.0	-4.68	PROT	C
ATOM	399	CD2	LEU	B	24	28.767	26.051	24.599	1.0	-4.62	PROT	C
ATOM	400	H	LEU	B	24	26.425	29.033	24.135	1.0	3.31	PROT	H
ATOM	401	HA	LEU	B	24	27.017	26.638	22.543	1.0	1.81	PROT	H
ATOM	402	HB2	LEU	B	24	25.447	26.969	25.186	1.0	1.65	PROT	H
ATOM	403	HB3	LEU	B	24	25.986	25.462	24.464	1.0	1.66	PROT	H
ATOM	404	HG	LEU	B	24	27.794	27.717	25.539	1.0	1.34	PROT	H
ATOM	405	HD11	LEU	B	24	27.244	24.864	26.582	1.0	1.50	PROT	H
ATOM	406	HD12	LEU	B	24	26.606	26.354	27.281	1.0	1.47	PROT	H
ATOM	407	HD13	LEU	B	24	28.341	26.041	27.300	1.0	1.46	PROT	H
ATOM	408	HD21	LEU	B	24	28.610	24.998	24.334	1.0	1.51	PROT	H
ATOM	409	HD22	LEU	B	24	29.684	26.098	25.199	1.0	1.43	PROT	H
ATOM	410	HD23	LEU	B	24	28.969	26.596	23.668	1.0	1.51	PROT	H
ATOM	411	N	ASP	B	25	24.868	26.533	21.291	1.0	-5.34	PROT	N
ATOM	412	CA	ASP	B	25	23.695	26.617	20.433	1.0	-0.04	PROT	C
ATOM	413	C	ASP	B	25	23.242	25.273	19.892	1.0	5.72	PROT	C
ATOM	414	O	ASP	B	25	23.772	24.764	18.897	1.0	-5.71	PROT	O
ATOM	415	CB	ASP	B	25	24.010	27.552	19.270	1.0	-4.15	PROT	C
ATOM	416	CG	ASP	B	25	22.799	27.869	18.431	1.0	7.66	PROT	C
ATOM	417	OD1	ASP	B	25	21.685	27.379	18.717	1.0	-8.48	PROT	O
ATOM	418	OD2	ASP	B	25	22.995	28.648	17.484	1.0	-7.78	PROT	O
ATOM	419	H	ASP	B	25	25.744	26.330	20.800	1.0	3.38	PROT	H
ATOM	420	HA	ASP	B	25	22.840	27.086	21.033	1.0	2.08	PROT	H
ATOM	421	HB2	ASP	B	25	24.787	27.106	18.605	1.0	1.95	PROT	H
ATOM	422	HB3	ASP	B	25	24.447	28.506	19.641	1.0	1.85	PROT	H
ATOM	423	N	THR	B	26	22.233	24.709	20.543	1.0	-5.66	PROT	N
ATOM	424	CA	THR	B	26	21.718	23.402	20.156	1.0	-0.75	PROT	C
ATOM	425	C	THR	B	26	20.953	23.439	18.831	1.0	5.90	PROT	C
ATOM	426	O	THR	B	26	20.741	22.399	18.209	1.0	-5.92	PROT	O
ATOM	427	CB	THR	B	26	20.811	22.839	21.247	1.0	1.36	PROT	C
ATOM	428	OG1	THR	B	26	19.692	23.712	21.437	1.0	-5.04	PROT	O
ATOM	429	CG2	THR	B	26	21.600	22.669	22.564	1.0	-5.20	PROT	C
ATOM	430	H	THR	B	26	22.007	25.037	21.473	1.0	3.17	PROT	H

ATOM	431	HA	THR	B	26	22.590	22.687	19.989	1.0	2.15	PROT	H
ATOM	432	HB	THR	B	26	20.314	21.891	20.929	1.0	1.83	PROT	H
ATOM	433	HG21	THR	B	26	22.026	23.597	22.956	1.0	1.61	PROT	H
ATOM	434	HG22	THR	B	26	22.426	21.957	22.436	1.0	1.79	PROT	H
ATOM	435	HG23	THR	B	26	20.947	22.263	23.348	1.0	1.66	PROT	H
ATOM	436	HG1	THR	B	26	20.032	24.580	21.736	1.0	2.61	PROT	H
ATOM	437	N	GLY	B	27	20.539	24.631	18.403	1.0	-5.42	PROT	N
ATOM	438	CA	GLY	B	27	19.851	24.798	17.128	1.0	-1.62	PROT	C
ATOM	439	C	GLY	B	27	20.772	24.903	15.927	1.0	5.74	PROT	C
ATOM	440	O	GLY	B	27	20.295	24.957	14.794	1.0	-6.72	PROT	O
ATOM	441	H	GLY	B	27	20.799	25.522	18.853	1.0	3.64	PROT	H
ATOM	442	HA2	GLY	B	27	19.163	23.923	16.953	1.0	1.96	PROT	H
ATOM	443	HA3	GLY	B	27	19.198	25.701	17.172	1.0	1.71	PROT	H
ATOM	444	N	ALA	B	28	22.080	24.964	16.165	1.0	-5.07	PROT	N
ATOM	445	CA	ALA	B	28	23.071	25.051	15.094	1.0	0.24	PROT	C
ATOM	446	C	ALA	B	28	23.745	23.693	14.870	1.0	5.61	PROT	C
ATOM	447	O	ALA	B	28	24.235	23.082	15.818	1.0	-5.82	PROT	O
ATOM	448	CB	ALA	B	28	24.111	26.102	15.447	1.0	-4.81	PROT	C
ATOM	449	H	ALA	B	28	22.475	24.854	17.108	1.0	3.57	PROT	H
ATOM	450	HA	ALA	B	28	22.535	25.366	14.148	1.0	1.59	PROT	H
ATOM	451	HB1	ALA	B	28	24.632	25.849	16.388	1.0	1.83	PROT	H
ATOM	452	HB2	ALA	B	28	23.665	27.093	15.608	1.0	1.68	PROT	H
ATOM	453	HB3	ALA	B	28	24.901	26.180	14.685	1.0	1.83	PROT	H
ATOM	454	N	ASP	B	29	23.757	23.221	13.627	1.0	-5.61	PROT	N
ATOM	455	CA	ASP	B	29	24.399	21.959	13.264	1.0	0.35	PROT	C
ATOM	456	C	ASP	B	29	25.914	22.120	13.323	1.0	5.85	PROT	C
ATOM	457	O	ASP	B	29	26.638	21.208	13.735	1.0	-6.29	PROT	O
ATOM	458	CB	ASP	B	29	24.052	21.545	11.831	1.0	-4.25	PROT	C
ATOM	459	CG	ASP	B	29	22.569	21.352	11.590	1.0	7.50	PROT	C
ATOM	460	OD1	ASP	B	29	21.772	21.179	12.537	1.0	-7.73	PROT	O
ATOM	461	OD2	ASP	B	29	22.201	21.348	10.399	1.0	-8.74	PROT	O
ATOM	462	H	ASP	B	29	23.137	23.614	12.912	1.0	3.35	PROT	H
ATOM	463	HA	ASP	B	29	24.081	21.150	13.988	1.0	1.98	PROT	H
ATOM	464	HB2	ASP	B	29	24.561	20.583	11.586	1.0	2.03	PROT	H
ATOM	465	HB3	ASP	B	29	24.455	22.263	11.082	1.0	1.92	PROT	H
ATOM	466	N	ASP	B	30	26.361	23.302	12.902	1.0	-5.07	PROT	N
ATOM	467	CA	ASP	B	30	27.763	23.632	12.716	1.0	0.30	PROT	C
ATOM	468	C	ASP	B	30	28.237	24.678	13.716	1.0	5.49	PROT	C

ATOM	469	O	ASP	B	30	27.433	25.400	14.301	1.0	-5.98	PROT	O
ATOM	470	CB	ASP	B	30	27.965	24.207	11.317	1.0	-4.46	PROT	C
ATOM	471	CG	ASP	B	30	27.441	23.292	10.239	1.0	7.64	PROT	C
ATOM	472	OD1	ASP	B	30	27.835	22.113	10.232	1.0	-8.20	PROT	O
ATOM	473	OD2	ASP	B	30	26.647	23.760	9.400	1.0	-8.03	PROT	O
ATOM	474	H	ASP	B	30	25.714	24.042	12.636	1.0	3.23	PROT	H
ATOM	475	HA	ASP	B	30	28.368	22.676	12.823	1.0	2.03	PROT	H
ATOM	476	HB2	ASP	B	30	29.044	24.382	11.118	1.0	1.73	PROT	H
ATOM	477	HB3	ASP	B	30	27.481	25.201	11.220	1.0	1.65	PROT	H
ATOM	478	N	THR	B	31	29.555	24.758	13.864	1.0	-5.31	PROT	N
ATOM	479	CA	THR	B	31	30.211	25.760	14.692	1.0	-0.60	PROT	C
ATOM	480	C	THR	B	31	30.687	26.891	13.773	1.0	5.59	PROT	C
ATOM	481	O	THR	B	31	31.395	26.646	12.790	1.0	-6.10	PROT	O
ATOM	482	CB	THR	B	31	31.352	25.095	15.488	1.0	1.81	PROT	C
ATOM	483	OG1	THR	B	31	30.790	24.223	16.475	1.0	-5.93	PROT	O
ATOM	484	CG2	THR	B	31	32.236	26.111	16.175	1.0	-5.34	PROT	C
ATOM	485	H	THR	B	31	30.199	24.185	13.312	1.0	3.39	PROT	H
ATOM	486	HA	THR	B	31	29.457	26.169	15.439	1.0	2.02	PROT	H
ATOM	487	HB	THR	B	31	31.939	24.382	14.860	1.0	1.60	PROT	H
ATOM	488	HG21	THR	B	31	31.657	26.839	16.765	1.0	1.81	PROT	H
ATOM	489	HG22	THR	B	31	32.837	26.688	15.460	1.0	1.66	PROT	H
ATOM	490	HG23	THR	B	31	32.935	25.626	16.868	1.0	1.60	PROT	H
ATOM	491	HG1	THR	B	31	30.255	24.739	17.114	1.0	3.36	PROT	H
ATOM	492	N	VAL	B	32	30.264	28.117	14.070	1.0	-5.32	PROT	N
ATOM	493	CA	VAL	B	32	30.572	29.280	13.240	1.0	-0.41	PROT	C
ATOM	494	C	VAL	B	32	31.167	30.419	14.064	1.0	5.86	PROT	C
ATOM	495	O	VAL	B	32	30.527	30.948	14.974	1.0	-6.61	PROT	O
ATOM	496	CB	VAL	B	32	29.332	29.835	12.505	1.0	-0.69	PROT	C
ATOM	497	CG1	VAL	B	32	29.759	30.849	11.441	1.0	-4.65	PROT	C
ATOM	498	CG2	VAL	B	32	28.566	28.718	11.830	1.0	-4.50	PROT	C
ATOM	499	H	VAL	B	32	29.807	28.329	14.969	1.0	3.53	PROT	H
ATOM	500	HA	VAL	B	32	31.318	28.942	12.447	1.0	2.02	PROT	H
ATOM	501	HB	VAL	B	32	28.673	30.346	13.256	1.0	1.58	PROT	H
ATOM	502	HG11	VAL	B	32	30.413	30.403	10.685	1.0	1.45	PROT	H
ATOM	503	HG12	VAL	B	32	30.295	31.701	11.877	1.0	1.45	PROT	H
ATOM	504	HG13	VAL	B	32	28.889	31.267	10.920	1.0	1.55	PROT	H
ATOM	505	HG21	VAL	B	32	29.200	28.148	11.137	1.0	1.53	PROT	H
ATOM	506	HG22	VAL	B	32	27.713	29.107	11.262	1.0	1.43	PROT	H

ATOM	507	HG23	VAL	B	32	28.168	27.997	12.561	1.0	1.63	PROT	H
ATOM	508	N	NME	B	33	32.387	30.784	13.736	1.0	-6.19	PROT	N
ATOM	509	H1	NME	B	33	32.864	31.514	14.249	1.0	3.15	PROT	H
ATOM	510	H2	NME	B	33	32.932	30.330	13.025	1.0	3.45	PROT	H
ATOM	511	C	ACE	B	34	27.714	27.558	3.963	1.0	4.51	PROT	C
ATOM	512	O	ACE	B	34	28.073	26.485	4.490	1.0	-6.42	PROT	O
ATOM	513	HC	ACE	B	34	28.377	28.014	3.196	1.0	1.37	PROT	H
ATOM	514	N	ILE	B	47	26.625	28.179	4.253	1.0	-4.95	PROT	N
ATOM	515	CA	ILE	B	47	25.685	27.668	5.253	1.0	-0.49	PROT	C
ATOM	516	C	ILE	B	47	24.491	28.603	5.389	1.0	5.60	PROT	C
ATOM	517	O	ILE	B	47	24.590	29.804	5.147	1.0	-6.39	PROT	O
ATOM	518	CB	ILE	B	47	26.315	27.491	6.658	1.0	-0.85	PROT	C
ATOM	519	CG1	ILE	B	47	26.825	28.829	7.207	1.0	-2.84	PROT	C
ATOM	520	CG2	ILE	B	47	27.407	26.439	6.622	1.0	-4.29	PROT	C
ATOM	521	CD1	ILE	B	47	27.293	28.761	8.646	1.0	-4.34	PROT	C
ATOM	522	H	ILE	B	47	26.390	29.107	3.884	1.0	3.38	PROT	H
ATOM	523	HA	ILE	B	47	25.343	26.647	4.897	1.0	1.87	PROT	H
ATOM	524	HB	ILE	B	47	25.501	27.122	7.340	1.0	1.46	PROT	H
ATOM	525	HG12	ILE	B	47	27.649	29.201	6.563	1.0	1.40	PROT	H
ATOM	526	HG13	ILE	B	47	26.031	29.599	7.117	1.0	1.38	PROT	H
ATOM	527	HG21	ILE	B	47	28.400	26.843	6.399	1.0	1.59	PROT	H
ATOM	528	HG22	ILE	B	47	27.161	25.582	5.983	1.0	1.67	PROT	H
ATOM	529	HG23	ILE	B	47	27.513	25.988	7.632	1.0	1.59	PROT	H
ATOM	530	HD11	ILE	B	47	28.194	28.137	8.744	1.0	1.60	PROT	H
ATOM	531	HD12	ILE	B	47	26.527	28.322	9.300	1.0	1.47	PROT	H
ATOM	532	HD13	ILE	B	47	27.541	29.758	9.030	1.0	1.38	PROT	H
ATOM	533	N	GLY	B	48	23.370	28.035	5.812	1.0	-5.06	PROT	N
ATOM	534	CA	GLY	B	48	22.108	28.749	5.826	1.0	-1.79	PROT	C
ATOM	535	C	GLY	B	48	21.533	28.854	7.220	1.0	5.79	PROT	C
ATOM	536	O	GLY	B	48	21.619	27.922	8.019	1.0	-6.16	PROT	O
ATOM	537	H	GLY	B	48	23.322	27.063	6.131	1.0	3.50	PROT	H
ATOM	538	HA2	GLY	B	48	22.230	29.756	5.343	1.0	1.96	PROT	H
ATOM	539	HA3	GLY	B	48	21.363	28.207	5.182	1.0	1.85	PROT	H
ATOM	540	N	GLY	B	49	20.947	30.011	7.499	1.0	-5.07	PROT	N
ATOM	541	CA	GLY	B	49	20.199	30.213	8.726	1.0	-1.81	PROT	C
ATOM	542	C	GLY	B	49	18.881	30.902	8.456	1.0	5.62	PROT	C
ATOM	543	O	GLY	B	49	18.371	30.878	7.332	1.0	-6.07	PROT	O
ATOM	544	H	GLY	B	49	20.863	30.774	6.823	1.0	3.44	PROT	H

ATOM	545	HA2	GLY	B	49	20.014	29.229	9.234	1.0	1.84	PROT	H
ATOM	546	HA3	GLY	B	49	20.829	30.808	9.444	1.0	1.88	PROT	H
ATOM	547	N	ILE	B	50	18.350	31.581	9.468	1.0	-5.64	PROT	N
ATOM	548	CA	ILE	B	50	17.011	32.150	9.363	1.0	-0.36	PROT	C
ATOM	549	C	ILE	B	50	16.950	33.266	8.325	1.0	6.01	PROT	C
ATOM	550	O	ILE	B	50	15.949	33.402	7.621	1.0	-6.58	PROT	O
ATOM	551	CB	ILE	B	50	16.471	32.654	10.722	1.0	-0.89	PROT	C
ATOM	552	CG1	ILE	B	50	15.036	32.159	10.917	1.0	-2.71	PROT	C
ATOM	553	CG2	ILE	B	50	16.522	34.177	10.817	1.0	-4.59	PROT	C
ATOM	554	CD1	ILE	B	50	14.367	32.632	12.193	1.0	-4.33	PROT	C
ATOM	555	H	ILE	B	50	18.811	31.662	10.386	1.0	3.57	PROT	H
ATOM	556	HA	ILE	B	50	16.326	31.333	8.971	1.0	1.79	PROT	H
ATOM	557	HB	ILE	B	50	17.107	32.234	11.548	1.0	1.53	PROT	H
ATOM	558	HG12	ILE	B	50	14.418	32.478	10.047	1.0	1.55	PROT	H
ATOM	559	HG13	ILE	B	50	15.016	31.046	10.889	1.0	1.34	PROT	H
ATOM	560	HG21	ILE	B	50	15.843	34.665	10.106	1.0	1.63	PROT	H
ATOM	561	HG22	ILE	B	50	17.528	34.577	10.662	1.0	1.42	PROT	H
ATOM	562	HG23	ILE	B	50	16.218	34.518	11.819	1.0	1.68	PROT	H
ATOM	563	HD11	ILE	B	50	14.177	33.713	12.180	1.0	1.52	PROT	H
ATOM	564	HD12	ILE	B	50	14.981	32.410	13.073	1.0	1.44	PROT	H
ATOM	565	HD13	ILE	B	50	13.396	32.136	12.329	1.0	1.47	PROT	H
ATOM	566	N	NME	B	51	18.013	34.036	8.251	1.0	-6.12	PROT	N
ATOM	567	H1	NME	B	51	18.102	34.766	7.559	1.0	3.21	PROT	H
ATOM	568	H2	NME	B	51	18.848	33.877	8.806	1.0	3.41	PROT	H
ATOM	569	C	ACE	B	52	33.667	25.469	11.155	1.0	4.39	PROT	C
ATOM	570	O	ACE	B	52	33.281	24.851	10.140	1.0	-6.22	PROT	O
ATOM	571	HC	ACE	B	52	33.918	24.887	12.070	1.0	1.40	PROT	H
ATOM	572	N	LEU	B	76	33.782	26.748	11.224	1.0	-4.91	PROT	N
ATOM	573	CA	LEU	B	76	33.465	27.611	10.097	1.0	-0.24	PROT	C
ATOM	574	C	LEU	B	76	33.715	29.047	10.534	1.0	5.87	PROT	C
ATOM	575	O	LEU	B	76	33.365	29.435	11.651	1.0	-6.76	PROT	O
ATOM	576	CB	LEU	B	76	32.012	27.421	9.660	1.0	-3.13	PROT	C
ATOM	577	CG	LEU	B	76	31.597	25.971	9.401	1.0	-0.09	PROT	C
ATOM	578	CD1	LEU	B	76	30.132	25.919	9.006	1.0	-4.82	PROT	C
ATOM	579	CD2	LEU	B	76	32.471	25.328	8.319	1.0	-3.95	PROT	C
ATOM	580	H	LEU	B	76	33.777	27.233	12.136	1.0	3.41	PROT	H
ATOM	581	HA	LEU	B	76	34.146	27.327	9.240	1.0	1.71	PROT	H
ATOM	582	HB2	LEU	B	76	31.346	27.834	10.456	1.0	1.67	PROT	H

ATOM	583	HB3	LEU	B	76	31.820	28.030	8.754	1.0	1.38	PROT	H
ATOM	584	HG	LEU	B	76	31.553	25.416	10.381	1.0	1.70	PROT	H
ATOM	585	HD11	LEU	B	76	29.923	26.480	8.087	1.0	1.37	PROT	H
ATOM	586	HD12	LEU	B	76	29.483	26.327	9.791	1.0	1.42	PROT	H
ATOM	587	HD13	LEU	B	76	29.810	24.884	8.820	1.0	1.65	PROT	H
ATOM	588	HD21	LEU	B	76	32.070	25.578	7.321	1.0	1.08	PROT	H
ATOM	589	HD22	LEU	B	76	32.462	24.231	8.370	1.0	1.47	PROT	H
ATOM	590	HD23	LEU	B	76	33.507	25.677	8.291	1.0	1.24	PROT	H
ATOM	591	N	NME	B	77	34.315	29.816	9.652	1.0	-6.03	PROT	N
ATOM	592	H1	NME	B	77	34.542	29.525	8.712	1.0	3.31	PROT	H
ATOM	593	H2	NME	B	77	34.506	30.793	9.838	1.0	3.22	PROT	H
ATOM	594	C	ACE	B	78	29.511	35.626	10.186	1.0	4.57	PROT	C
ATOM	595	O	ACE	B	78	28.898	34.568	9.933	1.0	-6.69	PROT	O
ATOM	596	HC	ACE	B	78	30.301	35.952	9.475	1.0	1.46	PROT	H
ATOM	597	N	THR	B	80	29.287	36.364	11.216	1.0	-4.86	PROT	N
ATOM	598	CA	THR	B	80	28.270	36.021	12.205	1.0	-0.76	PROT	C
ATOM	599	C	THR	B	80	28.271	37.103	13.284	1.0	5.67	PROT	C
ATOM	600	O	THR	B	80	29.318	37.669	13.602	1.0	-6.29	PROT	O
ATOM	601	CB	THR	B	80	28.507	34.615	12.796	1.0	1.82	PROT	C
ATOM	602	OG1	THR	B	80	27.492	34.308	13.764	1.0	-6.34	PROT	O
ATOM	603	CG2	THR	B	80	29.885	34.501	13.440	1.0	-5.11	PROT	C
ATOM	604	H	THR	B	80	29.877	37.171	11.468	1.0	3.50	PROT	H
ATOM	605	HA	THR	B	80	27.271	35.989	11.671	1.0	1.82	PROT	H
ATOM	606	HB	THR	B	80	28.353	33.842	12.000	1.0	1.70	PROT	H
ATOM	607	HG21	THR	B	80	30.080	35.312	14.153	1.0	1.68	PROT	H
ATOM	608	HG22	THR	B	80	30.688	34.510	12.697	1.0	1.56	PROT	H
ATOM	609	HG23	THR	B	80	29.975	33.556	14.005	1.0	1.89	PROT	H
ATOM	610	HG1	THR	B	80	27.883	34.057	14.633	1.0	3.78	PROT	H
ATOM	611	N	PRO	B	81	27.092	37.441	13.821	1.0	-4.64	PROT	N
ATOM	612	CA	PRO	B	81	27.018	38.385	14.933	1.0	-0.02	PROT	C
ATOM	613	C	PRO	B	81	27.639	37.878	16.221	1.0	5.72	PROT	C
ATOM	614	O	PRO	B	81	28.036	38.671	17.079	1.0	-6.35	PROT	O
ATOM	615	CB	PRO	B	81	25.512	38.583	15.151	1.0	-2.80	PROT	C
ATOM	616	CG	PRO	B	81	24.823	37.904	14.032	1.0	-3.06	PROT	C
ATOM	617	CD	PRO	B	81	25.769	36.964	13.383	1.0	-0.58	PROT	C
ATOM	618	HA	PRO	B	81	27.536	39.345	14.649	1.0	1.88	PROT	H
ATOM	619	HB2	PRO	B	81	25.191	38.184	16.139	1.0	1.62	PROT	H
ATOM	620	HB3	PRO	B	81	25.266	39.665	15.210	1.0	1.66	PROT	H

ATOM	621	HG2	PRO	B	81	24.435	38.649	13.293	1.0	1.63	PROT	H
ATOM	622	HG3	PRO	B	81	23.899	37.383	14.366	1.0	1.70	PROT	H
ATOM	623	HD2	PRO	B	81	25.695	36.974	12.270	1.0	1.50	PROT	H
ATOM	624	HD3	PRO	B	81	25.632	35.897	13.704	1.0	1.75	PROT	H
ATOM	625	N	VAL	B	82	27.675	36.557	16.358	1.0	-5.26	PROT	N
ATOM	626	CA	VAL	B	82	28.090	35.909	17.588	1.0	-0.36	PROT	C
ATOM	627	C	VAL	B	82	28.859	34.646	17.221	1.0	5.72	PROT	C
ATOM	628	O	VAL	B	82	28.540	33.974	16.239	1.0	-6.05	PROT	O
ATOM	629	CB	VAL	B	82	26.860	35.522	18.438	1.0	-0.69	PROT	C
ATOM	630	CG1	VAL	B	82	27.280	34.753	19.674	1.0	-4.85	PROT	C
ATOM	631	CG2	VAL	B	82	26.073	36.745	18.847	1.0	-4.47	PROT	C
ATOM	632	H	VAL	B	82	27.371	35.919	15.612	1.0	3.49	PROT	H
ATOM	633	HA	VAL	B	82	28.737	36.623	18.181	1.0	1.92	PROT	H
ATOM	634	HB	VAL	B	82	26.202	34.860	17.810	1.0	1.53	PROT	H
ATOM	635	HG11	VAL	B	82	28.054	35.263	20.257	1.0	1.47	PROT	H
ATOM	636	HG12	VAL	B	82	27.654	33.744	19.445	1.0	1.84	PROT	H
ATOM	637	HG13	VAL	B	82	26.438	34.597	20.362	1.0	1.58	PROT	H
ATOM	638	HG21	VAL	B	82	26.694	37.471	19.390	1.0	1.54	PROT	H
ATOM	639	HG22	VAL	B	82	25.235	36.485	19.506	1.0	1.50	PROT	H
ATOM	640	HG23	VAL	B	82	25.651	37.278	17.986	1.0	1.41	PROT	H
ATOM	641	N	ASN	B	83	29.867	34.326	18.023	1.0	-5.20	PROT	N
ATOM	642	CA	ASN	B	83	30.531	33.029	17.946	1.0	0.27	PROT	C
ATOM	643	C	ASN	B	83	29.546	31.944	18.386	1.0	5.52	PROT	C
ATOM	644	O	ASN	B	83	28.909	32.042	19.441	1.0	-6.40	PROT	O
ATOM	645	CB	ASN	B	83	31.794	33.019	18.814	1.0	-3.89	PROT	C
ATOM	646	CG	ASN	B	83	32.842	34.024	18.357	1.0	6.14	PROT	C
ATOM	647	OD1	ASN	B	83	33.139	34.132	17.169	1.0	-6.27	PROT	O
ATOM	648	ND2	ASN	B	83	33.417	34.756	19.304	1.0	-6.37	PROT	N
ATOM	649	H	ASN	B	83	30.092	34.882	18.848	1.0	3.32	PROT	H
ATOM	650	HA	ASN	B	83	30.834	32.861	16.858	1.0	2.13	PROT	H
ATOM	651	HB2	ASN	B	83	32.260	32.005	18.765	1.0	1.98	PROT	H
ATOM	652	HB3	ASN	B	83	31.524	33.153	19.883	1.0	1.78	PROT	H
ATOM	653	HD21	ASN	B	83	34.142	35.423	19.085	1.0	3.19	PROT	H
ATOM	654	HD22	ASN	B	83	33.211	34.666	20.285	1.0	3.26	PROT	H
ATOM	655	N	ILE	B	84	29.398	30.913	17.561	1.0	-5.14	PROT	N
ATOM	656	CA	ILE	B	84	28.416	29.865	17.806	1.0	-0.52	PROT	C
ATOM	657	C	ILE	B	84	29.096	28.504	17.897	1.0	5.88	PROT	C
ATOM	658	O	ILE	B	84	29.813	28.089	16.984	1.0	-6.67	PROT	O

ATOM	659	CB	ILE	B	84	27.355	29.846	16.689	1.0	-0.90	PROT	C
ATOM	660	CG1	ILE	B	84	26.542	31.145	16.707	1.0	-2.66	PROT	C
ATOM	661	CG2	ILE	B	84	26.423	28.673	16.848	1.0	-4.61	PROT	C
ATOM	662	CD1	ILE	B	84	26.063	31.546	15.344	1.0	-4.31	PROT	C
ATOM	663	H	ILE	B	84	29.836	30.850	16.628	1.0	3.72	PROT	H
ATOM	664	HA	ILE	B	84	27.882	30.092	18.785	1.0	1.88	PROT	H
ATOM	665	HB	ILE	B	84	27.893	29.767	15.705	1.0	1.56	PROT	H
ATOM	666	HG12	ILE	B	84	25.687	31.048	17.409	1.0	1.42	PROT	H
ATOM	667	HG13	ILE	B	84	27.151	31.977	17.128	1.0	1.45	PROT	H
ATOM	668	HG21	ILE	B	84	25.989	28.624	17.855	1.0	1.49	PROT	H
ATOM	669	HG22	ILE	B	84	26.912	27.712	16.641	1.0	1.54	PROT	H
ATOM	670	HG23	ILE	B	84	25.566	28.744	16.159	1.0	1.70	PROT	H
ATOM	671	HD11	ILE	B	84	25.389	30.803	14.903	1.0	1.40	PROT	H
ATOM	672	HD12	ILE	B	84	26.900	31.687	14.641	1.0	1.50	PROT	H
ATOM	673	HD13	ILE	B	84	25.547	32.514	15.369	1.0	1.48	PROT	H
ATOM	674	N	NME	B	85	28.865	27.822	18.998	1.0	-6.18	PROT	N
ATOM	675	H1	NME	B	85	29.235	26.894	19.141	1.0	3.20	PROT	H
ATOM	676	H2	NME	B	85	28.223	28.158	19.704	1.0	3.43	PROT	H
HETATM	677	C1	1UN	B	201	17.652	27.449	12.799	1.0	-1.40	PROT	C
HETATM	678	C2	1UN	B	201	18.374	26.811	11.621	1.0	-1.02	PROT	C
HETATM	679	C3	1UN	B	201	17.542	26.750	10.345	1.0	-2.90	PROT	C
HETATM	680	C4	1UN	B	201	16.888	28.099	10.083	1.0	-2.68	PROT	C
HETATM	681	C5	1UN	B	201	16.166	28.586	11.331	1.0	-2.59	PROT	C
HETATM	682	C6	1UN	B	201	17.124	28.836	12.494	1.0	-2.93	PROT	C
HETATM	683	N7	1UN	B	201	19.700	28.322	13.727	1.0	-0.03	PROT	N
HETATM	684	C8	1UN	B	201	20.465	27.644	12.689	1.0	-1.81	PROT	C
HETATM	685	C9	1UN	B	201	19.663	27.580	11.387	1.0	-3.16	PROT	C
HETATM	686	C10	1UN	B	201	18.519	27.519	14.044	1.0	-2.63	PROT	C
HETATM	687	C11	1UN	B	201	21.710	28.377	12.321	1.0	5.68	PROT	C
HETATM	688	N12	1UN	B	201	22.742	27.555	12.137	1.0	-5.29	PROT	N
HETATM	689	C13	1UN	B	201	24.063	27.918	11.675	1.0	3.17	PROT	C
HETATM	690	C14	1UN	B	201	24.692	28.754	12.789	1.0	-4.90	PROT	C
HETATM	691	C15	1UN	B	201	23.961	28.678	10.350	1.0	-5.21	PROT	C
HETATM	692	C16	1UN	B	201	24.863	26.630	11.448	1.0	-4.80	PROT	C
HETATM	693	O17	1UN	B	201	21.681	29.593	12.185	1.0	-6.29	PROT	O
HETATM	694	C18	1UN	B	201	20.427	28.349	14.983	1.0	-3.10	PROT	C
HETATM	695	C19	1UN	B	201	19.936	29.535	15.793	1.0	1.12	PROT	C
HETATM	696	C20	1UN	B	201	20.599	30.846	15.391	1.0	0.24	PROT	C

HETATM	697	O21	1UN	B	201	20.218	29.192	17.133	1.0	-6.14	PROT	O
HETATM	698	N22	1UN	B	201	19.883	31.916	16.047	1.0	-5.52	PROT	N
HETATM	699	C23	1UN	B	201	22.074	31.006	15.749	1.0	-3.81	PROT	C
HETATM	700	C24	1UN	B	201	18.896	32.595	15.470	1.0	6.15	PROT	C
HETATM	701	O25	1UN	B	201	18.562	32.485	14.305	1.0	-6.56	PROT	O
HETATM	702	C29	1UN	B	201	18.170	33.550	16.360	1.0	-1.04	PROT	C
HETATM	703	C30	1UN	B	201	18.900	34.427	17.164	1.0	-1.83	PROT	C
HETATM	704	C31	1UN	B	201	18.281	35.330	18.026	1.0	-1.04	PROT	C
HETATM	705	C32	1UN	B	201	16.896	35.393	18.100	1.0	-2.27	PROT	C
HETATM	706	C33	1UN	B	201	16.101	34.547	17.331	1.0	2.70	PROT	C
HETATM	707	C34	1UN	B	201	16.696	33.560	16.406	1.0	-0.52	PROT	C
HETATM	708	O38	1UN	B	201	14.786	34.583	17.403	1.0	-4.40	PROT	O
HETATM	709	C39	1UN	B	201	15.793	32.667	15.591	1.0	-4.52	PROT	C
HETATM	710	S74	1UN	B	201	22.767	32.328	14.801	1.0	0.45	PROT	S
HETATM	711	C77	1UN	B	201	22.652	33.787	15.649	1.0	-0.89	PROT	C
HETATM	712	C78	1UN	B	201	22.472	34.943	14.901	1.0	-1.53	PROT	C
HETATM	713	C79	1UN	B	201	22.360	36.167	15.541	1.0	-1.37	PROT	C
HETATM	714	C80	1UN	B	201	22.449	36.238	16.925	1.0	-1.56	PROT	C
HETATM	715	C81	1UN	B	201	22.634	35.080	17.670	1.0	-1.47	PROT	C
HETATM	716	C82	1UN	B	201	22.735	33.847	17.037	1.0	-1.40	PROT	C
HETATM	717	H1	1UN	B	201	16.772	26.782	13.047	1.0	1.66	PROT	H
HETATM	718	H2	1UN	B	201	18.636	25.752	11.903	1.0	1.49	PROT	H
HETATM	719	H31	1UN	B	201	18.167	26.439	9.485	1.0	1.51	PROT	H
HETATM	720	H32	1UN	B	201	16.766	25.963	10.436	1.0	1.42	PROT	H
HETATM	721	H41	1UN	B	201	17.641	28.842	9.755	1.0	1.36	PROT	H
HETATM	722	H42	1UN	B	201	16.181	28.024	9.234	1.0	1.37	PROT	H
HETATM	723	H51	1UN	B	201	15.371	27.870	11.622	1.0	1.44	PROT	H
HETATM	724	H52	1UN	B	201	15.604	29.522	11.097	1.0	1.55	PROT	H
HETATM	725	H61	1UN	B	201	16.582	29.272	13.356	1.0	1.51	PROT	H
HETATM	726	H62	1UN	B	201	17.895	29.572	12.208	1.0	1.39	PROT	H
HETATM	727	H7	1UN	B	201	19.436	29.300	13.394	1.0	3.16	PROT	H
HETATM	728	H8	1UN	B	201	20.673	26.567	13.034	1.0	2.40	PROT	H
HETATM	729	H91	1UN	B	201	19.456	28.597	10.994	1.0	1.63	PROT	H
HETATM	730	H92	1UN	B	201	20.269	27.089	10.588	1.0	1.84	PROT	H
HETATM	731	H101	1UN	B	201	18.801	26.477	14.377	1.0	2.21	PROT	H
HETATM	732	H102	1UN	B	201	17.938	27.943	14.910	1.0	2.13	PROT	H
HETATM	733	H12	1UN	B	201	22.558	26.535	12.124	1.0	3.46	PROT	H
HETATM	734	H141	1UN	B	201	25.723	29.041	12.544	1.0	1.61	PROT	H

```
HETATM  735 H142 1UN B 201      24.732  28.212  13.743  1.0   1.67      PROT H
HETATM  736 H143 1UN B 201      24.135  29.686  12.958  1.0   1.62      PROT H
HETATM  737 H151 1UN B 201      24.947  28.952   9.960  1.0   1.58      PROT H
HETATM  738 H152 1UN B 201      23.389  29.609  10.459  1.0   1.66      PROT H
HETATM  739 H153 1UN B 201      23.452  28.087   9.575  1.0   1.75      PROT H
HETATM  740 H161 1UN B 201      25.859  26.861  11.043  1.0   1.73      PROT H
HETATM  741 H162 1UN B 201      24.391  25.961  10.717  1.0   1.68      PROT H
HETATM  742 H163 1UN B 201      25.024  26.076  12.382  1.0   1.64      PROT H
HETATM  743 H181 1UN B 201      20.326  27.398  15.581  1.0   2.26      PROT H
HETATM  744 H182 1UN B 201      21.543  28.413  14.845  1.0   2.07      PROT H
HETATM  745  H19 1UN B 201      18.815  29.627  15.817  1.0   1.81      PROT H
HETATM  746  H20 1UN B 201      20.483  30.949  14.266  1.0   1.60      PROT H
HETATM  747  H21 1UN B 201      21.213  28.994  17.274  1.0   4.03      PROT H
HETATM  748  H22 1UN B 201      20.034  31.975  17.074  1.0   3.64      PROT H
HETATM  749 H231 1UN B 201      22.663  30.079  15.533  1.0   2.08      PROT H
HETATM  750 H232 1UN B 201      22.220  31.099  16.853  1.0   2.07      PROT H
HETATM  751  H30 1UN B 201      19.991  34.419  17.112  1.0   1.57      PROT H
HETATM  752  H31 1UN B 201      18.890  35.998  18.636  1.0   1.59      PROT H
HETATM  753  H32 1UN B 201      16.391  36.130  18.743  1.0   2.10      PROT H
HETATM  754  H38 1UN B 201      14.331  33.762  17.085  1.0   3.35      PROT H
HETATM  755 H391 1UN B 201      16.366  32.074  14.863  1.0   1.71      PROT H
HETATM  756 H392 1UN B 201      15.078  33.267  15.008  1.0   1.62      PROT H
HETATM  757 H393 1UN B 201      15.243  31.958  16.226  1.0   1.57      PROT H
HETATM  758  H78 1UN B 201      22.417  34.908  13.810  1.0   1.60      PROT H
HETATM  759  H79 1UN B 201      22.156  37.071  14.963  1.0   1.58      PROT H
HETATM  760  H80 1UN B 201      22.361  37.206  17.418  1.0   1.54      PROT H
HETATM  761  H81 1UN B 201      22.725  35.086  18.748  1.0   1.48      PROT H
HETATM  762  H82 1UN B 201      22.878  32.951  17.646  1.0   1.70      PROT H
HETATM  763  O   HOH B 147      20.834  34.206  21.043  1.0  -7.94      PROT O
HETATM  764  H1  HOH B 147      20.410  33.424  20.693  1.0   3.71      PROT H
HETATM  765  H2  HOH B 147      20.160  34.657  21.587  1.0   3.90      PROT H
HETATM  766  O   HOH B 156      19.836  31.776  12.137  1.0  -8.05      PROT O
HETATM  767  H1  HOH B 156      20.557  31.182  12.326  1.0   3.70      PROT H
HETATM  768  H2  HOH B 156      19.423  32.077  12.969  1.0   4.00      PROT H
HETATM  769  O   HOH B 539      22.036  24.828  11.379  1.0  -7.38      PROT O
HETATM  770  H1  HOH B 539      22.411  24.833  10.488  1.0   3.78      PROT H
HETATM  771  H2  HOH B 539      21.355  24.149  11.357  1.0   3.68      PROT H
END
```

4.5 RIT-HIV^{Pro}

```
HEADER  data-set: HIV_RIT_full_wH2O_OPT
REMARK  MOPAC, Version: 23.1.2
REMARK  99
REMARK  99 MOE v2014.09 (Chemical Computing Group Inc.)
ATOM      1  C   ACE A   7      66.720  70.151  43.687  1.0   4.47      PROT C
ATOM      2  O   ACE A   7      67.242  70.410  42.582  1.0  -6.21      PROT O
ATOM      3  HC  ACE A   7      67.183  70.587  44.600  1.0   1.48      PROT H
ATOM      4  N   ARG A   8      65.680  69.410  43.840  1.0  -5.17      PROT N
ATOM      5  CA  ARG A   8      64.990  68.780  42.690  1.0   0.14      PROT C
ATOM      6  CB  ARG A   8      66.070  67.800  42.130  1.0  -2.81      PROT C
ATOM      7  CG  ARG A   8      67.100  68.400  41.190  1.0  -3.01      PROT C
ATOM      8  CD  ARG A   8      67.990  67.330  40.540  1.0  -0.55      PROT C
ATOM      9  NE  ARG A   8      68.630  67.920  39.310  1.0  -5.68      PROT N
ATOM     10  CZ  ARG A   8      69.350  67.300  38.420  1.0   6.36      PROT C
ATOM     11  NH1 ARG A   8      69.480  66.000  38.430  1.0  -6.50      PROT N
ATOM     12  NH2 ARG A   8      70.040  67.920  37.570  1.0  -6.08      PROT N
ATOM     13  C   ARG A   8      63.800  67.980  43.300  1.0   5.34      PROT C
ATOM     14  O   ARG A   8      64.090  67.100  44.180  1.0  -6.57      PROT O
ATOM     15  H   ARG A   8      65.325  69.141  44.763  1.0   3.36      PROT H
ATOM     16  HA  ARG A   8      64.687  69.544  41.937  1.0   1.89      PROT H
ATOM     17  HB2 ARG A   8      65.538  66.973  41.606  1.0   1.46      PROT H
ATOM     18  HB3 ARG A   8      66.572  67.298  42.984  1.0   1.59      PROT H
ATOM     19  HG2 ARG A   8      67.828  69.075  41.700  1.0   2.17      PROT H
ATOM     20  HG3 ARG A   8      66.611  69.046  40.429  1.0   1.86      PROT H
ATOM     21  HD2 ARG A   8      67.395  66.437  40.248  1.0   1.39      PROT H
ATOM     22  HD3 ARG A   8      68.788  66.997  41.237  1.0   1.45      PROT H
ATOM     23  HE  ARG A   8      68.474  68.982  39.230  1.0   4.11      PROT H
ATOM     24 HH11 ARG A   8      69.042  65.402  39.120  1.0   3.34      PROT H
ATOM     25 HH12 ARG A   8      70.067  65.492  37.778  1.0   3.40      PROT H
ATOM     26 HH21 ARG A   8      69.915  68.983  37.437  1.0   3.99      PROT H
ATOM     27 HH22 ARG A   8      70.539  67.495  36.791  1.0   3.48      PROT H
ATOM     28  N   NME A   9      62.607  68.294  42.844  1.0  -5.80      PROT N
ATOM     29  H1  NME A   9      62.452  68.991  42.125  1.0   3.38      PROT H
ATOM     30  H2  NME A   9      61.767  67.837  43.173  1.0   3.24      PROT H
ATOM     31  C   ACE A  10      63.098  61.939  38.334  1.0   5.07      PROT C
ATOM     32  O   ACE A  10      64.110  62.365  38.929  1.0  -6.94      PROT O
```

ATOM	33	HC	ACE	A	10	63.097	60.876	38.007	1.0	1.49	PROT	H
ATOM	34	N	LEU	A	23	62.060	62.650	38.070	1.0	-4.91	PROT	N
ATOM	35	CA	LEU	A	23	61.970	64.050	38.450	1.0	-0.15	PROT	C
ATOM	36	CB	LEU	A	23	63.110	64.760	37.780	1.0	-3.58	PROT	C
ATOM	37	CG	LEU	A	23	63.300	66.300	37.920	1.0	-0.61	PROT	C
ATOM	38	CD1	LEU	A	23	63.850	66.820	39.230	1.0	-4.41	PROT	C
ATOM	39	CD2	LEU	A	23	64.290	66.600	36.850	1.0	-4.63	PROT	C
ATOM	40	C	LEU	A	23	60.630	64.640	38.010	1.0	6.02	PROT	C
ATOM	41	O	LEU	A	23	60.140	64.470	36.920	1.0	-6.68	PROT	O
ATOM	42	H	LEU	A	23	61.359	62.338	37.386	1.0	3.34	PROT	H
ATOM	43	HA	LEU	A	23	62.079	64.119	39.576	1.0	1.79	PROT	H
ATOM	44	HB2	LEU	A	23	63.074	64.513	36.690	1.0	1.72	PROT	H
ATOM	45	HB3	LEU	A	23	64.074	64.298	38.122	1.0	2.02	PROT	H
ATOM	46	HG	LEU	A	23	62.317	66.797	37.718	1.0	1.31	PROT	H
ATOM	47	HD11	LEU	A	23	64.834	66.390	39.458	1.0	1.51	PROT	H
ATOM	48	HD12	LEU	A	23	63.962	67.913	39.202	1.0	1.60	PROT	H
ATOM	49	HD13	LEU	A	23	63.192	66.578	40.072	1.0	1.27	PROT	H
ATOM	50	HD21	LEU	A	23	65.222	66.026	36.958	1.0	1.47	PROT	H
ATOM	51	HD22	LEU	A	23	63.908	66.396	35.839	1.0	1.44	PROT	H
ATOM	52	HD23	LEU	A	23	64.590	67.664	36.838	1.0	1.71	PROT	H
ATOM	53	N	LEU	A	24	60.020	65.400	38.900	1.0	-5.31	PROT	N
ATOM	54	CA	LEU	A	24	58.720	66.070	38.650	1.0	0.12	PROT	C
ATOM	55	CB	LEU	A	24	57.880	66.380	39.960	1.0	-3.02	PROT	C
ATOM	56	CG	LEU	A	24	57.450	65.160	40.830	1.0	-0.49	PROT	C
ATOM	57	CD1	LEU	A	24	56.640	65.660	42.010	1.0	-4.66	PROT	C
ATOM	58	CD2	LEU	A	24	56.560	64.150	40.050	1.0	-4.61	PROT	C
ATOM	59	C	LEU	A	24	59.040	67.350	37.770	1.0	5.32	PROT	C
ATOM	60	O	LEU	A	24	59.710	68.300	38.290	1.0	-5.95	PROT	O
ATOM	61	H	LEU	A	24	60.424	65.618	39.811	1.0	3.33	PROT	H
ATOM	62	HA	LEU	A	24	58.088	65.362	38.040	1.0	1.71	PROT	H
ATOM	63	HB2	LEU	A	24	56.977	66.929	39.633	1.0	1.62	PROT	H
ATOM	64	HB3	LEU	A	24	58.460	67.083	40.587	1.0	1.62	PROT	H
ATOM	65	HG	LEU	A	24	58.366	64.631	41.179	1.0	1.37	PROT	H
ATOM	66	HD11	LEU	A	24	57.206	66.368	42.629	1.0	1.50	PROT	H
ATOM	67	HD12	LEU	A	24	55.722	66.171	41.692	1.0	1.51	PROT	H
ATOM	68	HD13	LEU	A	24	56.335	64.836	42.667	1.0	1.50	PROT	H
ATOM	69	HD21	LEU	A	24	55.669	64.638	39.646	1.0	1.50	PROT	H
ATOM	70	HD22	LEU	A	24	57.106	63.700	39.216	1.0	1.45	PROT	H

ATOM	71	HD23	LEU	A	24	56.231	63.338	40.705	1.0	1.46	PROT	H
ATOM	72	N	ASH	A	25	58.640	67.280	36.520	1.0	-5.08	PROT	N
ATOM	73	CA	ASH	A	25	58.960	68.300	35.500	1.0	0.02	PROT	C
ATOM	74	CB	ASH	A	25	59.810	67.580	34.400	1.0	-3.56	PROT	C
ATOM	75	CG	ASH	A	25	60.640	68.490	33.540	1.0	6.37	PROT	C
ATOM	76	OD1	ASH	A	25	60.210	69.120	32.540	1.0	-5.17	PROT	O
ATOM	77	OD2	ASH	A	25	61.930	68.620	33.880	1.0	-6.18	PROT	O
ATOM	78	C	ASH	A	25	57.730	68.960	34.790	1.0	5.39	PROT	C
ATOM	79	O	ASH	A	25	57.110	68.270	34.040	1.0	-5.34	PROT	O
ATOM	80	H	ASH	A	25	58.112	66.477	36.160	1.0	3.54	PROT	H
ATOM	81	HA	ASH	A	25	59.592	69.094	35.995	1.0	1.85	PROT	H
ATOM	82	HB2	ASH	A	25	59.129	66.955	33.770	1.0	2.10	PROT	H
ATOM	83	HB3	ASH	A	25	60.516	66.867	34.903	1.0	2.19	PROT	H
ATOM	84	HD1	ASH	A	25	60.915	69.545	31.937	1.0	4.13	PROT	H
ATOM	85	N	THR	A	26	57.540	70.240	34.990	1.0	-5.36	PROT	N
ATOM	86	CA	THR	A	26	56.400	70.870	34.300	1.0	-0.94	PROT	C
ATOM	87	CB	THR	A	26	55.790	72.040	35.040	1.0	1.77	PROT	C
ATOM	88	CG2	THR	A	26	55.100	71.650	36.360	1.0	-4.82	PROT	C
ATOM	89	OG1	THR	A	26	56.800	72.960	35.360	1.0	-6.45	PROT	O
ATOM	90	C	THR	A	26	56.630	71.220	32.780	1.0	5.96	PROT	C
ATOM	91	O	THR	A	26	55.790	71.500	31.990	1.0	-5.47	PROT	O
ATOM	92	H	THR	A	26	57.923	70.755	35.786	1.0	3.51	PROT	H
ATOM	93	HA	THR	A	26	55.572	70.075	34.222	1.0	2.17	PROT	H
ATOM	94	HB	THR	A	26	55.042	72.551	34.373	1.0	1.52	PROT	H
ATOM	95	HG21	THR	A	26	55.810	71.203	37.070	1.0	1.71	PROT	H
ATOM	96	HG22	THR	A	26	54.681	72.539	36.847	1.0	1.64	PROT	H
ATOM	97	HG23	THR	A	26	54.286	70.937	36.192	1.0	1.66	PROT	H
ATOM	98	HG1	THR	A	26	56.942	73.606	34.630	1.0	3.68	PROT	H
ATOM	99	N	GLY	A	27	57.880	71.110	32.350	1.0	-5.68	PROT	N
ATOM	100	CA	GLY	A	27	58.400	71.500	31.020	1.0	-1.49	PROT	C
ATOM	101	C	GLY	A	27	58.140	70.430	29.910	1.0	5.50	PROT	C
ATOM	102	O	GLY	A	27	57.700	70.740	28.790	1.0	-5.60	PROT	O
ATOM	103	H	GLY	A	27	58.619	70.821	32.982	1.0	3.03	PROT	H
ATOM	104	HA2	GLY	A	27	59.484	71.721	31.086	1.0	1.61	PROT	H
ATOM	105	HA3	GLY	A	27	57.886	72.437	30.683	1.0	1.92	PROT	H
ATOM	106	N	ALA	A	28	58.470	69.200	30.260	1.0	-5.27	PROT	N
ATOM	107	CA	ALA	A	28	58.090	68.010	29.500	1.0	0.24	PROT	C
ATOM	108	CB	ALA	A	28	58.590	66.800	30.350	1.0	-4.66	PROT	C

ATOM	109	C	ALA	A	28	56.620	67.920	29.300	1.0	5.47	PROT	C
ATOM	110	O	ALA	A	28	55.840	67.930	30.250	1.0	-5.85	PROT	O
ATOM	111	H	ALA	A	28	58.657	69.011	31.249	1.0	3.24	PROT	H
ATOM	112	HA	ALA	A	28	58.658	68.033	28.522	1.0	1.77	PROT	H
ATOM	113	HB1	ALA	A	28	58.396	65.848	29.830	1.0	1.87	PROT	H
ATOM	114	HB2	ALA	A	28	59.668	66.860	30.523	1.0	1.59	PROT	H
ATOM	115	HB3	ALA	A	28	58.061	66.742	31.310	1.0	1.71	PROT	H
ATOM	116	N	ASP	A	29	56.180	67.720	28.080	1.0	-5.45	PROT	N
ATOM	117	CA	ASP	A	29	54.890	67.320	27.690	1.0	0.50	PROT	C
ATOM	118	CB	ASP	A	29	54.710	67.500	26.190	1.0	-4.35	PROT	C
ATOM	119	CG	ASP	A	29	54.880	68.980	25.610	1.0	7.50	PROT	C
ATOM	120	OD1	ASP	A	29	54.080	69.810	26.010	1.0	-7.77	PROT	O
ATOM	121	OD2	ASP	A	29	55.820	69.320	24.840	1.0	-7.90	PROT	O
ATOM	122	C	ASP	A	29	54.740	65.850	28.070	1.0	5.81	PROT	C
ATOM	123	O	ASP	A	29	53.790	65.510	28.770	1.0	-6.22	PROT	O
ATOM	124	H	ASP	A	29	56.814	67.927	27.280	1.0	3.38	PROT	H
ATOM	125	HA	ASP	A	29	54.083	67.898	28.250	1.0	2.14	PROT	H
ATOM	126	HB2	ASP	A	29	53.687	67.194	25.896	1.0	1.80	PROT	H
ATOM	127	HB3	ASP	A	29	55.406	66.860	25.616	1.0	1.69	PROT	H
ATOM	128	N	ASH	A	30	55.710	64.920	27.830	1.0	-5.48	PROT	N
ATOM	129	CA	ASH	A	30	55.650	63.510	28.120	1.0	-0.12	PROT	C
ATOM	130	CB	ASH	A	30	55.470	62.680	26.810	1.0	-3.37	PROT	C
ATOM	131	CG	ASH	A	30	54.120	62.920	26.130	1.0	6.64	PROT	C
ATOM	132	OD1	ASH	A	30	53.700	64.050	25.850	1.0	-4.66	PROT	O
ATOM	133	OD2	ASH	A	30	53.350	61.860	25.780	1.0	-7.19	PROT	O
ATOM	134	C	ASH	A	30	56.700	63.010	29.050	1.0	5.83	PROT	C
ATOM	135	O	ASH	A	30	57.780	63.510	29.040	1.0	-5.61	PROT	O
ATOM	136	H	ASH	A	30	56.567	65.207	27.351	1.0	3.24	PROT	H
ATOM	137	HA	ASH	A	30	54.643	63.366	28.679	1.0	2.06	PROT	H
ATOM	138	HB2	ASH	A	30	55.558	61.590	27.028	1.0	2.08	PROT	H
ATOM	139	HB3	ASH	A	30	56.284	62.906	26.087	1.0	1.99	PROT	H
ATOM	140	HD1	ASH	A	30	52.786	64.096	25.398	1.0	4.25	PROT	H
ATOM	141	N	THR	A	31	56.390	61.890	29.740	1.0	-5.54	PROT	N
ATOM	142	CA	THR	A	31	57.340	61.130	30.550	1.0	-0.58	PROT	C
ATOM	143	CB	THR	A	31	56.570	60.120	31.470	1.0	1.82	PROT	C
ATOM	144	CG2	THR	A	31	57.350	59.340	32.470	1.0	-5.25	PROT	C
ATOM	145	OG1	THR	A	31	55.600	60.910	32.190	1.0	-6.11	PROT	O
ATOM	146	C	THR	A	31	58.360	60.430	29.650	1.0	5.68	PROT	C

ATOM	147	O	THR	A	31	58.040	59.750	28.690	1.0	-6.07	PROT	O
ATOM	148	H	THR	A	31	55.446	61.500	29.719	1.0	3.38	PROT	H
ATOM	149	HA	THR	A	31	57.874	61.865	31.233	1.0	1.92	PROT	H
ATOM	150	HB	THR	A	31	55.912	59.466	30.843	1.0	1.59	PROT	H
ATOM	151	HG21	THR	A	31	58.112	59.950	32.987	1.0	1.80	PROT	H
ATOM	152	HG22	THR	A	31	56.694	58.920	33.247	1.0	1.72	PROT	H
ATOM	153	HG23	THR	A	31	57.892	58.496	32.019	1.0	1.72	PROT	H
ATOM	154	HG1	THR	A	31	56.055	61.523	32.803	1.0	3.40	PROT	H
ATOM	155	N	VAL	A	32	59.600	60.620	30.000	1.0	-5.23	PROT	N
ATOM	156	CA	VAL	A	32	60.790	60.000	29.450	1.0	-0.35	PROT	C
ATOM	157	CB	VAL	A	32	61.680	61.050	28.760	1.0	-0.73	PROT	C
ATOM	158	CG1	VAL	A	32	62.530	60.400	27.770	1.0	-4.72	PROT	C
ATOM	159	CG2	VAL	A	32	60.950	62.220	28.200	1.0	-4.53	PROT	C
ATOM	160	C	VAL	A	32	61.570	59.250	30.560	1.0	5.89	PROT	C
ATOM	161	O	VAL	A	32	61.700	59.700	31.690	1.0	-6.62	PROT	O
ATOM	162	H	VAL	A	32	59.823	61.171	30.858	1.0	3.49	PROT	H
ATOM	163	HA	VAL	A	32	60.453	59.242	28.671	1.0	1.91	PROT	H
ATOM	164	HB	VAL	A	32	62.349	61.468	29.587	1.0	1.52	PROT	H
ATOM	165	HG11	VAL	A	32	63.258	61.093	27.313	1.0	1.57	PROT	H
ATOM	166	HG12	VAL	A	32	63.135	59.574	28.180	1.0	1.52	PROT	H
ATOM	167	HG13	VAL	A	32	61.966	59.971	26.924	1.0	1.49	PROT	H
ATOM	168	HG21	VAL	A	32	60.175	61.922	27.476	1.0	1.54	PROT	H
ATOM	169	HG22	VAL	A	32	60.417	62.805	28.968	1.0	1.64	PROT	H
ATOM	170	HG23	VAL	A	32	61.622	62.917	27.682	1.0	1.47	PROT	H
ATOM	171	N	NME	A	33	62.088	58.092	30.212	1.0	-6.18	PROT	N
ATOM	172	H1	NME	A	33	61.992	57.662	29.305	1.0	3.44	PROT	H
ATOM	173	H2	NME	A	33	62.614	57.546	30.885	1.0	3.19	PROT	H
ATOM	174	C	ACE	A	34	62.607	61.524	18.509	1.0	4.51	PROT	C
ATOM	175	O	ACE	A	34	61.436	61.529	18.942	1.0	-6.62	PROT	O
ATOM	176	HC	ACE	A	34	62.885	60.758	17.751	1.0	1.47	PROT	H
ATOM	177	N	ILE	A	47	63.520	62.350	18.880	1.0	-4.84	PROT	N
ATOM	178	CA	ILE	A	47	63.260	63.400	19.870	1.0	-0.58	PROT	C
ATOM	179	CB	ILE	A	47	62.570	62.970	21.240	1.0	-0.88	PROT	C
ATOM	180	CG2	ILE	A	47	61.180	62.390	20.910	1.0	-4.16	PROT	C
ATOM	181	CG1	ILE	A	47	63.480	62.080	22.130	1.0	-2.72	PROT	C
ATOM	182	CD1	ILE	A	47	62.920	61.770	23.530	1.0	-4.35	PROT	C
ATOM	183	C	ILE	A	47	64.520	64.280	20.160	1.0	5.60	PROT	C
ATOM	184	O	ILE	A	47	65.590	63.900	19.730	1.0	-6.47	PROT	O

ATOM	185	H	ILE	A	47	64.503	62.293	18.582	1.0	3.48	PROT	H
ATOM	186	HA	ILE	A	47	62.505	64.095	19.381	1.0	1.72	PROT	H
ATOM	187	HB	ILE	A	47	62.393	63.917	21.811	1.0	1.31	PROT	H
ATOM	188	HG12	ILE	A	47	64.465	62.574	22.234	1.0	1.40	PROT	H
ATOM	189	HG13	ILE	A	47	63.675	61.129	21.598	1.0	1.43	PROT	H
ATOM	190	HG21	ILE	A	47	61.192	61.299	20.809	1.0	1.67	PROT	H
ATOM	191	HG22	ILE	A	47	60.513	62.558	21.776	1.0	1.24	PROT	H
ATOM	192	HG23	ILE	A	47	60.686	62.905	20.080	1.0	1.64	PROT	H
ATOM	193	HD11	ILE	A	47	63.646	61.200	24.120	1.0	1.47	PROT	H
ATOM	194	HD12	ILE	A	47	62.682	62.689	24.078	1.0	1.48	PROT	H
ATOM	195	HD13	ILE	A	47	62.000	61.178	23.464	1.0	1.48	PROT	H
ATOM	196	N	GLY	A	48	64.340	65.330	20.880	1.0	-4.96	PROT	N
ATOM	197	CA	GLY	A	48	65.270	66.420	21.120	1.0	-1.76	PROT	C
ATOM	198	C	GLY	A	48	64.640	67.450	22.040	1.0	5.76	PROT	C
ATOM	199	O	GLY	A	48	63.760	67.090	22.810	1.0	-5.93	PROT	O
ATOM	200	H	GLY	A	48	63.426	65.505	21.343	1.0	3.35	PROT	H
ATOM	201	HA2	GLY	A	48	65.609	66.857	20.146	1.0	1.86	PROT	H
ATOM	202	HA3	GLY	A	48	66.209	66.037	21.611	1.0	1.99	PROT	H
ATOM	203	N	GLY	A	49	65.200	68.640	22.050	1.0	-5.26	PROT	N
ATOM	204	CA	GLY	A	49	64.870	69.690	23.050	1.0	-1.54	PROT	C
ATOM	205	C	GLY	A	49	65.970	70.710	23.190	1.0	5.77	PROT	C
ATOM	206	O	GLY	A	49	65.690	71.860	22.880	1.0	-5.98	PROT	O
ATOM	207	H	GLY	A	49	65.962	68.889	21.418	1.0	3.36	PROT	H
ATOM	208	HA2	GLY	A	49	64.632	69.205	24.035	1.0	1.99	PROT	H
ATOM	209	HA3	GLY	A	49	63.930	70.210	22.731	1.0	1.78	PROT	H
ATOM	210	N	ILE	A	50	67.230	70.260	23.380	1.0	-5.48	PROT	N
ATOM	211	CA	ILE	A	50	68.410	71.080	23.490	1.0	-0.37	PROT	C
ATOM	212	CB	ILE	A	50	68.770	71.250	25.030	1.0	-0.84	PROT	C
ATOM	213	CG2	ILE	A	50	70.050	72.120	25.160	1.0	-4.60	PROT	C
ATOM	214	CG1	ILE	A	50	67.620	71.790	25.830	1.0	-2.59	PROT	C
ATOM	215	CD1	ILE	A	50	67.120	73.200	25.520	1.0	-4.39	PROT	C
ATOM	216	C	ILE	A	50	69.570	70.460	22.730	1.0	5.52	PROT	C
ATOM	217	O	ILE	A	50	69.790	69.240	22.800	1.0	-6.07	PROT	O
ATOM	218	H	ILE	A	50	67.390	69.266	23.629	1.0	3.57	PROT	H
ATOM	219	HA	ILE	A	50	68.173	72.111	23.091	1.0	1.81	PROT	H
ATOM	220	HB	ILE	A	50	69.020	70.223	25.403	1.0	1.41	PROT	H
ATOM	221	HG12	ILE	A	50	67.908	71.768	26.908	1.0	1.37	PROT	H
ATOM	222	HG13	ILE	A	50	66.753	71.095	25.759	1.0	1.38	PROT	H

ATOM	223	HG21	ILE	A	50	69.931	73.084	24.655	1.0	1.55	PROT	H
ATOM	224	HG22	ILE	A	50	70.261	72.340	26.213	1.0	1.60	PROT	H
ATOM	225	HG23	ILE	A	50	70.925	71.617	24.740	1.0	1.46	PROT	H
ATOM	226	HD11	ILE	A	50	66.719	73.268	24.502	1.0	1.49	PROT	H
ATOM	227	HD12	ILE	A	50	66.311	73.488	26.206	1.0	1.59	PROT	H
ATOM	228	HD13	ILE	A	50	67.915	73.947	25.627	1.0	1.49	PROT	H
ATOM	229	N	NME	A	51	70.296	71.293	22.017	1.0	-6.16	PROT	N
ATOM	230	H1	NME	A	51	70.127	72.287	21.969	1.0	3.28	PROT	H
ATOM	231	H2	NME	A	51	71.096	70.978	21.483	1.0	3.24	PROT	H
ATOM	232	C	ACE	A	52	57.248	57.838	26.807	1.0	4.19	PROT	C
ATOM	233	O	ACE	A	52	57.000	58.346	25.693	1.0	-6.03	PROT	O
ATOM	234	HC	ACE	A	52	56.466	57.887	27.598	1.0	1.49	PROT	H
ATOM	235	N	LEU	A	76	58.380	57.320	27.130	1.0	-4.91	PROT	N
ATOM	236	CA	LEU	A	76	59.480	57.280	26.220	1.0	-0.38	PROT	C
ATOM	237	CB	LEU	A	76	59.710	58.640	25.620	1.0	-3.16	PROT	C
ATOM	238	CG	LEU	A	76	58.490	59.310	24.940	1.0	0.15	PROT	C
ATOM	239	CD1	LEU	A	76	58.870	60.800	24.630	1.0	-5.13	PROT	C
ATOM	240	CD2	LEU	A	76	58.240	58.690	23.570	1.0	-4.35	PROT	C
ATOM	241	C	LEU	A	76	60.660	56.610	26.900	1.0	5.91	PROT	C
ATOM	242	O	LEU	A	76	60.950	56.950	28.050	1.0	-6.74	PROT	O
ATOM	243	H	LEU	A	76	58.636	57.210	28.125	1.0	3.37	PROT	H
ATOM	244	HA	LEU	A	76	59.150	56.610	25.346	1.0	1.72	PROT	H
ATOM	245	HB2	LEU	A	76	60.541	58.601	24.886	1.0	1.44	PROT	H
ATOM	246	HB3	LEU	A	76	60.059	59.332	26.425	1.0	1.60	PROT	H
ATOM	247	HG	LEU	A	76	57.651	59.699	25.612	1.0	2.12	PROT	H
ATOM	248	HD11	LEU	A	76	59.084	61.350	25.551	1.0	1.37	PROT	H
ATOM	249	HD12	LEU	A	76	59.751	60.881	23.990	1.0	1.33	PROT	H
ATOM	250	HD13	LEU	A	76	58.045	61.302	24.119	1.0	1.44	PROT	H
ATOM	251	HD21	LEU	A	76	57.357	59.131	23.093	1.0	1.40	PROT	H
ATOM	252	HD22	LEU	A	76	59.096	58.829	22.902	1.0	1.29	PROT	H
ATOM	253	HD23	LEU	A	76	58.045	57.614	23.646	1.0	1.35	PROT	H
ATOM	254	N	NME	A	77	61.289	55.701	26.188	1.0	-5.94	PROT	N
ATOM	255	H1	NME	A	77	61.069	55.490	25.225	1.0	3.34	PROT	H
ATOM	256	H2	NME	A	77	62.100	55.209	26.545	1.0	3.25	PROT	H
ATOM	257	C	ACE	A	78	68.500	59.565	26.873	1.0	4.64	PROT	C
ATOM	258	O	ACE	A	78	67.614	60.191	26.255	1.0	-6.72	PROT	O
ATOM	259	HC	ACE	A	78	68.799	58.564	26.491	1.0	1.50	PROT	H
ATOM	260	N	THR	A	80	69.090	60.000	27.930	1.0	-4.88	PROT	N

ATOM	261	CA	THR	A	80	68.760	61.330	28.530	1.0	-0.53	PROT	C
ATOM	262	CB	THR	A	80	67.310	61.330	28.920	1.0	1.89	PROT	C
ATOM	263	CG2	THR	A	80	66.830	60.200	29.840	1.0	-4.99	PROT	C
ATOM	264	OG1	THR	A	80	67.010	62.380	29.780	1.0	-6.77	PROT	O
ATOM	265	C	THR	A	80	69.590	61.590	29.750	1.0	5.85	PROT	C
ATOM	266	O	THR	A	80	69.980	60.620	30.380	1.0	-6.33	PROT	O
ATOM	267	H	THR	A	80	69.754	59.451	28.490	1.0	3.53	PROT	H
ATOM	268	HA	THR	A	80	68.946	62.112	27.733	1.0	1.87	PROT	H
ATOM	269	HB	THR	A	80	66.662	61.444	28.011	1.0	1.60	PROT	H
ATOM	270	HG21	THR	A	80	65.775	60.334	30.124	1.0	1.71	PROT	H
ATOM	271	HG22	THR	A	80	66.900	59.209	29.377	1.0	1.55	PROT	H
ATOM	272	HG23	THR	A	80	67.425	60.158	30.765	1.0	1.74	PROT	H
ATOM	273	HG1	THR	A	80	66.349	62.184	30.487	1.0	3.95	PROT	H
ATOM	274	N	PRO	A	81	69.900	62.830	30.210	1.0	-4.62	PROT	N
ATOM	275	CD	PRO	A	81	69.620	64.110	29.460	1.0	-0.63	PROT	C
ATOM	276	CG	PRO	A	81	69.960	65.290	30.410	1.0	-2.69	PROT	C
ATOM	277	CB	PRO	A	81	70.710	64.560	31.510	1.0	-2.96	PROT	C
ATOM	278	CA	PRO	A	81	70.510	63.070	31.460	1.0	0.06	PROT	C
ATOM	279	C	PRO	A	81	69.810	62.570	32.720	1.0	5.76	PROT	C
ATOM	280	O	PRO	A	81	70.430	62.140	33.730	1.0	-6.34	PROT	O
ATOM	281	HA	PRO	A	81	71.523	62.533	31.484	1.0	1.93	PROT	H
ATOM	282	HB2	PRO	A	81	70.427	64.976	32.505	1.0	1.69	PROT	H
ATOM	283	HB3	PRO	A	81	71.798	64.796	31.437	1.0	1.69	PROT	H
ATOM	284	HG2	PRO	A	81	69.048	65.791	30.777	1.0	1.62	PROT	H
ATOM	285	HG3	PRO	A	81	70.570	66.059	29.912	1.0	1.59	PROT	H
ATOM	286	HD2	PRO	A	81	68.549	64.125	29.162	1.0	1.61	PROT	H
ATOM	287	HD3	PRO	A	81	70.250	64.128	28.549	1.0	1.50	PROT	H
ATOM	288	N	VAL	A	82	68.450	62.680	32.710	1.0	-5.53	PROT	N
ATOM	289	CA	VAL	A	82	67.610	62.220	33.910	1.0	0.05	PROT	C
ATOM	290	CB	VAL	A	82	67.110	63.400	34.770	1.0	-0.84	PROT	C
ATOM	291	CG1	VAL	A	82	66.980	63.100	36.250	1.0	-4.65	PROT	C
ATOM	292	CG2	VAL	A	82	68.070	64.590	34.710	1.0	-4.37	PROT	C
ATOM	293	C	VAL	A	82	66.320	61.480	33.480	1.0	5.70	PROT	C
ATOM	294	O	VAL	A	82	65.830	61.710	32.350	1.0	-6.22	PROT	O
ATOM	295	H	VAL	A	82	67.923	62.860	31.861	1.0	3.47	PROT	H
ATOM	296	HA	VAL	A	82	68.273	61.544	34.515	1.0	1.91	PROT	H
ATOM	297	HB	VAL	A	82	66.114	63.736	34.372	1.0	1.51	PROT	H
ATOM	298	HG11	VAL	A	82	66.153	62.409	36.472	1.0	1.55	PROT	H

ATOM	299	HG12	VAL	A	82	67.895	62.671	36.674	1.0	1.54	PROT	H
ATOM	300	HG13	VAL	A	82	66.744	64.005	36.826	1.0	1.56	PROT	H
ATOM	301	HG21	VAL	A	82	68.151	64.999	33.694	1.0	1.50	PROT	H
ATOM	302	HG22	VAL	A	82	67.730	65.413	35.351	1.0	1.53	PROT	H
ATOM	303	HG23	VAL	A	82	69.081	64.304	35.026	1.0	1.51	PROT	H
ATOM	304	N	ASN	A	83	65.610	60.760	34.420	1.0	-5.67	PROT	N
ATOM	305	CA	ASN	A	83	64.210	60.270	34.260	1.0	0.79	PROT	C
ATOM	306	CB	ASN	A	83	63.940	59.080	35.190	1.0	-3.88	PROT	C
ATOM	307	CG	ASN	A	83	64.630	57.700	35.050	1.0	6.20	PROT	C
ATOM	308	OD1	ASN	A	83	64.800	57.220	33.950	1.0	-6.45	PROT	O
ATOM	309	ND2	ASN	A	83	65.020	57.010	36.100	1.0	-6.28	PROT	N
ATOM	310	C	ASN	A	83	63.280	61.440	34.670	1.0	5.28	PROT	C
ATOM	311	O	ASN	A	83	63.440	62.040	35.760	1.0	-6.40	PROT	O
ATOM	312	H	ASN	A	83	65.993	60.630	35.351	1.0	3.30	PROT	H
ATOM	313	HA	ASN	A	83	64.063	59.976	33.181	1.0	1.99	PROT	H
ATOM	314	HB2	ASN	A	83	62.846	58.845	35.104	1.0	1.94	PROT	H
ATOM	315	HB3	ASN	A	83	64.070	59.433	36.242	1.0	1.80	PROT	H
ATOM	316	HD21	ASN	A	83	64.911	57.312	37.055	1.0	3.22	PROT	H
ATOM	317	HD22	ASN	A	83	65.434	56.092	35.992	1.0	3.27	PROT	H
ATOM	318	N	ILE	A	84	62.270	61.660	33.920	1.0	-4.90	PROT	N
ATOM	319	CA	ILE	A	84	61.440	62.850	33.940	1.0	-0.70	PROT	C
ATOM	320	CB	ILE	A	84	62.030	63.910	32.960	1.0	-0.87	PROT	C
ATOM	321	CG2	ILE	A	84	60.950	64.820	32.210	1.0	-4.42	PROT	C
ATOM	322	CG1	ILE	A	84	63.030	64.820	33.730	1.0	-2.81	PROT	C
ATOM	323	CD1	ILE	A	84	63.880	65.810	32.860	1.0	-4.18	PROT	C
ATOM	324	C	ILE	A	84	59.990	62.540	33.780	1.0	5.79	PROT	C
ATOM	325	O	ILE	A	84	59.540	61.890	32.800	1.0	-6.47	PROT	O
ATOM	326	H	ILE	A	84	62.060	61.070	33.078	1.0	3.63	PROT	H
ATOM	327	HA	ILE	A	84	61.573	63.312	34.988	1.0	1.92	PROT	H
ATOM	328	HB	ILE	A	84	62.584	63.374	32.153	1.0	1.58	PROT	H
ATOM	329	HG12	ILE	A	84	63.731	64.174	34.294	1.0	1.48	PROT	H
ATOM	330	HG13	ILE	A	84	62.478	65.412	34.481	1.0	1.33	PROT	H
ATOM	331	HG21	ILE	A	84	61.467	65.517	31.548	1.0	1.52	PROT	H
ATOM	332	HG22	ILE	A	84	60.291	64.188	31.605	1.0	1.63	PROT	H
ATOM	333	HG23	ILE	A	84	60.347	65.370	32.927	1.0	1.29	PROT	H
ATOM	334	HD11	ILE	A	84	63.261	66.627	32.483	1.0	1.42	PROT	H
ATOM	335	HD12	ILE	A	84	64.677	66.248	33.469	1.0	1.52	PROT	H
ATOM	336	HD13	ILE	A	84	64.341	65.288	32.019	1.0	1.44	PROT	H

ATOM	337	N	NME	A	85	59.201	62.990	34.732	1.0	-6.11	PROT	N
ATOM	338	H1	NME	A	85	59.525	63.596	35.474	1.0	3.44	PROT	H
ATOM	339	H2	NME	A	85	58.201	62.844	34.693	1.0	3.22	PROT	H
TER	340		NME	A	85							
ATOM	341	C	ACE	B	7	50.136	72.506	26.996	1.0	4.39	PROT	C
ATOM	342	O	ACE	B	7	51.095	71.887	26.489	1.0	-6.35	PROT	O
ATOM	343	HC	ACE	B	7	49.105	72.224	26.687	1.0	1.49	PROT	H
ATOM	344	N	ARG	B	8	50.260	73.460	27.850	1.0	-4.95	PROT	N
ATOM	345	CA	ARG	B	8	51.580	73.920	28.320	1.0	-0.31	PROT	C
ATOM	346	CB	ARG	B	8	52.440	74.410	27.100	1.0	-2.81	PROT	C
ATOM	347	CG	ARG	B	8	52.840	73.130	26.320	1.0	-2.59	PROT	C
ATOM	348	CD	ARG	B	8	53.880	73.530	25.260	1.0	-1.03	PROT	C
ATOM	349	NE	ARG	B	8	54.230	72.290	24.560	1.0	-5.38	PROT	N
ATOM	350	CZ	ARG	B	8	54.640	72.200	23.340	1.0	6.08	PROT	C
ATOM	351	NH1	ARG	B	8	54.860	73.190	22.580	1.0	-6.11	PROT	N
ATOM	352	NH2	ARG	B	8	54.700	71.020	22.840	1.0	-6.23	PROT	N
ATOM	353	C	ARG	B	8	51.440	75.040	29.310	1.0	5.65	PROT	C
ATOM	354	O	ARG	B	8	50.520	75.860	29.180	1.0	-6.53	PROT	O
ATOM	355	H	ARG	B	8	49.471	74.026	28.184	1.0	3.43	PROT	H
ATOM	356	HA	ARG	B	8	52.103	73.025	28.780	1.0	1.98	PROT	H
ATOM	357	HB2	ARG	B	8	53.331	74.954	27.454	1.0	1.54	PROT	H
ATOM	358	HB3	ARG	B	8	51.863	75.106	26.473	1.0	1.55	PROT	H
ATOM	359	HG2	ARG	B	8	52.020	72.847	25.602	1.0	2.04	PROT	H
ATOM	360	HG3	ARG	B	8	53.168	72.299	26.961	1.0	1.85	PROT	H
ATOM	361	HD2	ARG	B	8	54.800	73.966	25.720	1.0	1.55	PROT	H
ATOM	362	HD3	ARG	B	8	53.463	74.298	24.574	1.0	1.44	PROT	H
ATOM	363	HE	ARG	B	8	54.166	71.398	25.183	1.0	4.22	PROT	H
ATOM	364	HH11	ARG	B	8	54.575	74.150	22.777	1.0	3.48	PROT	H
ATOM	365	HH12	ARG	B	8	55.298	73.103	21.661	1.0	3.40	PROT	H
ATOM	366	HH21	ARG	B	8	54.900	70.197	23.486	1.0	3.82	PROT	H
ATOM	367	HH22	ARG	B	8	55.072	70.844	21.908	1.0	3.19	PROT	H
ATOM	368	N	NME	B	9	52.335	75.066	30.273	1.0	-5.96	PROT	N
ATOM	369	H1	NME	B	9	53.048	74.362	30.407	1.0	3.32	PROT	H
ATOM	370	H2	NME	B	9	52.323	75.782	30.989	1.0	3.25	PROT	H
ATOM	371	C	ACE	B	10	58.075	80.056	28.485	1.0	4.88	PROT	C
ATOM	372	O	ACE	B	10	57.365	79.537	27.598	1.0	-6.93	PROT	O
ATOM	373	HC	ACE	B	10	58.542	81.043	28.268	1.0	1.52	PROT	H
ATOM	374	N	LEU	B	23	58.310	79.520	29.630	1.0	-4.80	PROT	N

ATOM	375	CA	LEU	B	23	57.760	78.240	30.000	1.0	-0.19	PROT	C
ATOM	376	CB	LEU	B	23	58.190	77.210	28.930	1.0	-3.31	PROT	C
ATOM	377	CG	LEU	B	23	57.880	75.700	29.230	1.0	-0.58	PROT	C
ATOM	378	CD1	LEU	B	23	56.340	75.540	29.300	1.0	-4.56	PROT	C
ATOM	379	CD2	LEU	B	23	58.430	74.760	28.200	1.0	-4.56	PROT	C
ATOM	380	C	LEU	B	23	58.210	77.840	31.440	1.0	5.87	PROT	C
ATOM	381	O	LEU	B	23	59.330	78.060	31.810	1.0	-6.04	PROT	O
ATOM	382	H	LEU	B	23	59.081	79.856	30.227	1.0	3.42	PROT	H
ATOM	383	HA	LEU	B	23	56.632	78.315	29.982	1.0	1.73	PROT	H
ATOM	384	HB2	LEU	B	23	59.280	77.312	28.750	1.0	1.67	PROT	H
ATOM	385	HB3	LEU	B	23	57.708	77.470	27.963	1.0	1.90	PROT	H
ATOM	386	HG	LEU	B	23	58.312	75.456	30.230	1.0	1.22	PROT	H
ATOM	387	HD11	LEU	B	23	55.876	75.805	28.344	1.0	1.52	PROT	H
ATOM	388	HD12	LEU	B	23	56.070	74.501	29.528	1.0	1.57	PROT	H
ATOM	389	HD13	LEU	B	23	55.899	76.171	30.072	1.0	1.20	PROT	H
ATOM	390	HD21	LEU	B	23	58.067	74.994	27.189	1.0	1.52	PROT	H
ATOM	391	HD22	LEU	B	23	59.528	74.787	28.156	1.0	1.52	PROT	H
ATOM	392	HD23	LEU	B	23	58.144	73.716	28.404	1.0	1.66	PROT	H
ATOM	393	N	LEU	B	24	57.230	77.480	32.320	1.0	-5.69	PROT	N
ATOM	394	CA	LEU	B	24	57.520	77.180	33.720	1.0	0.42	PROT	C
ATOM	395	CB	LEU	B	24	56.240	77.390	34.500	1.0	-3.39	PROT	C
ATOM	396	CG	LEU	B	24	55.700	78.890	34.520	1.0	-0.29	PROT	C
ATOM	397	CD1	LEU	B	24	54.320	78.820	35.260	1.0	-4.66	PROT	C
ATOM	398	CD2	LEU	B	24	56.640	79.680	35.390	1.0	-4.69	PROT	C
ATOM	399	C	LEU	B	24	57.930	75.670	33.770	1.0	5.77	PROT	C
ATOM	400	O	LEU	B	24	57.280	74.810	33.330	1.0	-5.97	PROT	O
ATOM	401	H	LEU	B	24	56.296	77.233	32.000	1.0	3.32	PROT	H
ATOM	402	HA	LEU	B	24	58.356	77.835	34.088	1.0	1.88	PROT	H
ATOM	403	HB2	LEU	B	24	56.378	77.066	35.552	1.0	1.68	PROT	H
ATOM	404	HB3	LEU	B	24	55.432	76.739	34.106	1.0	1.62	PROT	H
ATOM	405	HG	LEU	B	24	55.595	79.290	33.500	1.0	1.39	PROT	H
ATOM	406	HD11	LEU	B	24	54.426	78.382	36.255	1.0	1.50	PROT	H
ATOM	407	HD12	LEU	B	24	53.898	79.822	35.378	1.0	1.46	PROT	H
ATOM	408	HD13	LEU	B	24	53.600	78.220	34.697	1.0	1.45	PROT	H
ATOM	409	HD21	LEU	B	24	57.644	79.763	34.951	1.0	1.51	PROT	H
ATOM	410	HD22	LEU	B	24	56.285	80.709	35.543	1.0	1.48	PROT	H
ATOM	411	HD23	LEU	B	24	56.759	79.239	36.389	1.0	1.55	PROT	H
ATOM	412	N	ASH	B	25	59.020	75.290	34.440	1.0	-5.66	PROT	N

ATOM	413	CA	ASH	B	25	59.630	73.980	34.490	1.0	-0.07	PROT	C
ATOM	414	CB	ASH	B	25	60.740	73.820	33.380	1.0	-3.36	PROT	C
ATOM	415	CG	ASH	B	25	61.330	72.390	33.260	1.0	6.16	PROT	C
ATOM	416	OD1	ASH	B	25	61.210	71.520	34.110	1.0	-5.46	PROT	O
ATOM	417	OD2	ASH	B	25	62.140	72.320	32.210	1.0	-5.88	PROT	O
ATOM	418	C	ASH	B	25	60.320	73.640	35.860	1.0	5.36	PROT	C
ATOM	419	O	ASH	B	25	61.350	74.180	36.220	1.0	-5.45	PROT	O
ATOM	420	H	ASH	B	25	59.631	76.017	34.849	1.0	3.33	PROT	H
ATOM	421	HA	ASH	B	25	58.841	73.202	34.270	1.0	1.75	PROT	H
ATOM	422	HB2	ASH	B	25	61.597	74.500	33.597	1.0	2.14	PROT	H
ATOM	423	HB3	ASH	B	25	60.341	74.119	32.392	1.0	1.95	PROT	H
ATOM	424	HD1	ASH	B	25	61.791	70.661	34.000	1.0	4.36	PROT	H
ATOM	425	N	THR	B	26	59.550	72.870	36.640	1.0	-5.45	PROT	N
ATOM	426	CA	THR	B	26	60.010	72.590	37.970	1.0	-0.68	PROT	C
ATOM	427	CB	THR	B	26	58.830	71.910	38.730	1.0	1.93	PROT	C
ATOM	428	CG2	THR	B	26	57.710	72.860	38.860	1.0	-5.10	PROT	C
ATOM	429	OG1	THR	B	26	58.330	70.830	37.890	1.0	-6.53	PROT	O
ATOM	430	C	THR	B	26	61.190	71.610	37.990	1.0	5.59	PROT	C
ATOM	431	O	THR	B	26	61.570	71.190	39.100	1.0	-5.91	PROT	O
ATOM	432	H	THR	B	26	58.608	72.572	36.370	1.0	3.58	PROT	H
ATOM	433	HA	THR	B	26	60.338	73.528	38.510	1.0	2.15	PROT	H
ATOM	434	HB	THR	B	26	59.177	71.468	39.692	1.0	1.60	PROT	H
ATOM	435	HG21	THR	B	26	57.212	73.078	37.897	1.0	1.79	PROT	H
ATOM	436	HG22	THR	B	26	56.909	72.466	39.509	1.0	1.68	PROT	H
ATOM	437	HG23	THR	B	26	58.013	73.828	39.290	1.0	1.77	PROT	H
ATOM	438	HG1	THR	B	26	58.859	70.009	38.046	1.0	3.62	PROT	H
ATOM	439	N	GLY	B	27	61.710	71.140	36.860	1.0	-5.24	PROT	N
ATOM	440	CA	GLY	B	27	62.940	70.370	36.840	1.0	-1.84	PROT	C
ATOM	441	C	GLY	B	27	64.160	71.070	36.240	1.0	5.57	PROT	C
ATOM	442	O	GLY	B	27	65.190	70.430	36.050	1.0	-5.54	PROT	O
ATOM	443	H	GLY	B	27	61.355	71.424	35.963	1.0	3.12	PROT	H
ATOM	444	HA2	GLY	B	27	62.774	69.411	36.278	1.0	1.87	PROT	H
ATOM	445	HA3	GLY	B	27	63.215	70.043	37.884	1.0	2.18	PROT	H
ATOM	446	N	ALA	B	28	64.060	72.380	35.840	1.0	-5.72	PROT	N
ATOM	447	CA	ALA	B	28	65.280	73.140	35.500	1.0	0.38	PROT	C
ATOM	448	CB	ALA	B	28	64.850	74.210	34.460	1.0	-4.48	PROT	C
ATOM	449	C	ALA	B	28	65.920	73.620	36.800	1.0	5.67	PROT	C
ATOM	450	O	ALA	B	28	65.240	74.020	37.760	1.0	-6.19	PROT	O

ATOM	451	H	ALA	B	28	63.276	72.947	36.162	1.0	3.29	PROT H
ATOM	452	HA	ALA	B	28	65.997	72.431	34.983	1.0	1.86	PROT H
ATOM	453	HB1	ALA	B	28	64.416	73.733	33.579	1.0	1.54	PROT H
ATOM	454	HB2	ALA	B	28	64.100	74.895	34.877	1.0	1.61	PROT H
ATOM	455	HB3	ALA	B	28	65.709	74.833	34.170	1.0	1.84	PROT H
ATOM	456	N	ASP	B	29	67.270	73.510	37.000	1.0	-5.68	PROT N
ATOM	457	CA	ASP	B	29	67.950	73.720	38.320	1.0	0.50	PROT C
ATOM	458	CB	ASP	B	29	69.240	72.970	38.400	1.0	-4.25	PROT C
ATOM	459	CG	ASP	B	29	69.050	71.430	38.440	1.0	7.55	PROT C
ATOM	460	OD1	ASP	B	29	68.330	70.890	39.250	1.0	-7.37	PROT O
ATOM	461	OD2	ASP	B	29	69.430	70.790	37.380	1.0	-8.99	PROT O
ATOM	462	C	ASP	B	29	68.210	75.190	38.500	1.0	5.84	PROT C
ATOM	463	O	ASP	B	29	67.680	75.800	39.460	1.0	-6.34	PROT O
ATOM	464	H	ASP	B	29	67.860	73.086	36.287	1.0	3.31	PROT H
ATOM	465	HA	ASP	B	29	67.233	73.376	39.131	1.0	2.04	PROT H
ATOM	466	HB2	ASP	B	29	69.798	73.260	39.320	1.0	1.95	PROT H
ATOM	467	HB3	ASP	B	29	69.923	73.197	37.553	1.0	1.83	PROT H
ATOM	468	N	ASP	B	30	68.760	75.780	37.450	1.0	-5.10	PROT N
ATOM	469	CA	ASP	B	30	68.730	77.230	37.110	1.0	0.75	PROT C
ATOM	470	CB	ASP	B	30	70.110	77.760	36.850	1.0	-4.49	PROT C
ATOM	471	CG	ASP	B	30	71.220	77.590	37.860	1.0	7.58	PROT C
ATOM	472	OD1	ASP	B	30	72.410	77.720	37.450	1.0	-8.22	PROT O
ATOM	473	OD2	ASP	B	30	70.940	77.500	39.090	1.0	-8.09	PROT O
ATOM	474	C	ASP	B	30	67.820	77.430	35.830	1.0	5.56	PROT C
ATOM	475	O	ASP	B	30	67.270	76.580	35.160	1.0	-5.78	PROT O
ATOM	476	H	ASP	B	30	69.179	75.221	36.702	1.0	3.29	PROT H
ATOM	477	HA	ASP	B	30	68.241	77.773	37.970	1.0	1.91	PROT H
ATOM	478	HB2	ASP	B	30	70.069	78.865	36.675	1.0	1.79	PROT H
ATOM	479	HB3	ASP	B	30	70.500	77.358	35.881	1.0	1.81	PROT H
ATOM	480	N	THR	B	31	67.630	78.720	35.460	1.0	-6.01	PROT N
ATOM	481	CA	THR	B	31	66.660	79.260	34.440	1.0	-0.42	PROT C
ATOM	482	CB	THR	B	31	66.010	80.580	35.110	1.0	1.80	PROT C
ATOM	483	CG2	THR	B	31	65.370	81.530	34.010	1.0	-4.92	PROT C
ATOM	484	OG1	THR	B	31	65.010	80.310	36.080	1.0	-5.97	PROT O
ATOM	485	C	THR	B	31	67.480	79.590	33.210	1.0	5.74	PROT C
ATOM	486	O	THR	B	31	68.510	80.230	33.370	1.0	-6.19	PROT O
ATOM	487	H	THR	B	31	68.230	79.461	35.828	1.0	3.27	PROT H
ATOM	488	HA	THR	B	31	65.852	78.511	34.254	1.0	1.90	PROT H

ATOM	489	HB	THR	B	31	66.808	81.140	35.648	1.0	1.40	PROT	H
ATOM	490	HG21	THR	B	31	64.693	80.961	33.365	1.0	1.71	PROT	H
ATOM	491	HG22	THR	B	31	64.795	82.318	34.496	1.0	1.51	PROT	H
ATOM	492	HG23	THR	B	31	66.154	81.978	33.398	1.0	1.58	PROT	H
ATOM	493	HG1	THR	B	31	65.210	79.462	36.538	1.0	3.49	PROT	H
ATOM	494	N	VAL	B	32	66.940	79.290	32.060	1.0	-5.25	PROT	N
ATOM	495	CA	VAL	B	32	67.550	79.490	30.720	1.0	-0.44	PROT	C
ATOM	496	CB	VAL	B	32	67.770	78.170	29.890	1.0	-0.56	PROT	C
ATOM	497	CG1	VAL	B	32	68.890	77.300	30.380	1.0	-4.61	PROT	C
ATOM	498	CG2	VAL	B	32	66.550	77.260	29.800	1.0	-4.54	PROT	C
ATOM	499	C	VAL	B	32	66.860	80.550	29.860	1.0	6.00	PROT	C
ATOM	500	O	VAL	B	32	65.680	80.370	29.690	1.0	-6.58	PROT	O
ATOM	501	H	VAL	B	32	65.985	78.904	31.997	1.0	3.49	PROT	H
ATOM	502	HA	VAL	B	32	68.613	79.853	30.939	1.0	1.85	PROT	H
ATOM	503	HB	VAL	B	32	68.016	78.526	28.851	1.0	1.40	PROT	H
ATOM	504	HG11	VAL	B	32	68.971	76.372	29.794	1.0	1.60	PROT	H
ATOM	505	HG12	VAL	B	32	69.870	77.793	30.308	1.0	1.43	PROT	H
ATOM	506	HG13	VAL	B	32	68.757	77.007	31.432	1.0	1.62	PROT	H
ATOM	507	HG21	VAL	B	32	65.666	77.821	29.460	1.0	1.56	PROT	H
ATOM	508	HG22	VAL	B	32	66.708	76.454	29.074	1.0	1.49	PROT	H
ATOM	509	HG23	VAL	B	32	66.308	76.805	30.766	1.0	1.46	PROT	H
ATOM	510	N	NME	B	33	67.633	81.518	29.417	1.0	-6.28	PROT	N
ATOM	511	H1	NME	B	33	68.566	81.660	29.779	1.0	3.50	PROT	H
ATOM	512	H2	NME	B	33	67.274	82.257	28.830	1.0	3.16	PROT	H
ATOM	513	C	ACE	B	34	76.461	74.323	29.667	1.0	4.51	PROT	C
ATOM	514	O	ACE	B	34	76.235	74.602	30.863	1.0	-6.48	PROT	O
ATOM	515	HC	ACE	B	34	77.315	74.825	29.162	1.0	1.40	PROT	H
ATOM	516	N	ILE	B	47	75.760	73.500	28.970	1.0	-4.94	PROT	N
ATOM	517	CA	ILE	B	47	74.610	72.800	29.530	1.0	-0.45	PROT	C
ATOM	518	CB	ILE	B	47	73.540	73.810	30.110	1.0	-0.84	PROT	C
ATOM	519	CG2	ILE	B	47	74.100	74.820	31.090	1.0	-4.18	PROT	C
ATOM	520	CG1	ILE	B	47	72.850	74.600	28.950	1.0	-2.75	PROT	C
ATOM	521	CD1	ILE	B	47	71.850	75.660	29.420	1.0	-4.34	PROT	C
ATOM	522	C	ILE	B	47	73.960	71.870	28.450	1.0	5.58	PROT	C
ATOM	523	O	ILE	B	47	74.470	71.880	27.320	1.0	-6.40	PROT	O
ATOM	524	H	ILE	B	47	75.930	73.308	27.974	1.0	3.43	PROT	H
ATOM	525	HA	ILE	B	47	74.962	72.152	30.382	1.0	1.82	PROT	H
ATOM	526	HB	ILE	B	47	72.766	73.193	30.628	1.0	1.42	PROT	H

ATOM	527	HG12	ILE	B	47	72.333	73.887	28.281	1.0	1.26	PROT	H
ATOM	528	HG13	ILE	B	47	73.630	75.079	28.331	1.0	1.34	PROT	H
ATOM	529	HG21	ILE	B	47	73.253	75.289	31.637	1.0	1.41	PROT	H
ATOM	530	HG22	ILE	B	47	74.683	74.355	31.898	1.0	1.71	PROT	H
ATOM	531	HG23	ILE	B	47	74.601	75.674	30.624	1.0	1.57	PROT	H
ATOM	532	HD11	ILE	B	47	71.276	76.053	28.571	1.0	1.46	PROT	H
ATOM	533	HD12	ILE	B	47	71.130	75.240	30.135	1.0	1.59	PROT	H
ATOM	534	HD13	ILE	B	47	72.348	76.510	29.900	1.0	1.47	PROT	H
ATOM	535	N	GLY	B	48	72.960	71.070	28.880	1.0	-5.14	PROT	N
ATOM	536	CA	GLY	B	48	72.340	70.120	28.000	1.0	-1.88	PROT	C
ATOM	537	C	GLY	B	48	71.040	69.530	28.540	1.0	5.94	PROT	C
ATOM	538	O	GLY	B	48	70.580	69.830	29.640	1.0	-6.31	PROT	O
ATOM	539	H	GLY	B	48	72.533	71.169	29.806	1.0	3.57	PROT	H
ATOM	540	HA2	GLY	B	48	73.063	69.285	27.778	1.0	1.87	PROT	H
ATOM	541	HA3	GLY	B	48	72.148	70.595	26.996	1.0	1.89	PROT	H
ATOM	542	N	GLY	B	49	70.400	68.850	27.620	1.0	-5.23	PROT	N
ATOM	543	CA	GLY	B	49	69.010	68.420	27.770	1.0	-1.55	PROT	C
ATOM	544	C	GLY	B	49	68.810	67.200	26.840	1.0	5.63	PROT	C
ATOM	545	O	GLY	B	49	69.800	66.680	26.310	1.0	-6.43	PROT	O
ATOM	546	H	GLY	B	49	70.848	68.488	26.765	1.0	3.54	PROT	H
ATOM	547	HA2	GLY	B	49	68.318	69.248	27.486	1.0	1.76	PROT	H
ATOM	548	HA3	GLY	B	49	68.759	68.155	28.828	1.0	2.01	PROT	H
ATOM	549	N	ILE	B	50	67.560	66.840	26.610	1.0	-5.17	PROT	N
ATOM	550	CA	ILE	B	50	67.350	65.690	25.670	1.0	-0.31	PROT	C
ATOM	551	CB	ILE	B	50	65.850	65.210	25.760	1.0	-0.99	PROT	C
ATOM	552	CG2	ILE	B	50	65.550	64.120	24.730	1.0	-4.61	PROT	C
ATOM	553	CG1	ILE	B	50	65.560	64.620	27.230	1.0	-2.51	PROT	C
ATOM	554	CD1	ILE	B	50	64.390	63.690	27.410	1.0	-4.35	PROT	C
ATOM	555	C	ILE	B	50	67.810	65.990	24.250	1.0	5.89	PROT	C
ATOM	556	O	ILE	B	50	67.590	67.090	23.800	1.0	-6.04	PROT	O
ATOM	557	H	ILE	B	50	66.743	67.269	27.047	1.0	3.66	PROT	H
ATOM	558	HA	ILE	B	50	67.985	64.845	26.079	1.0	1.62	PROT	H
ATOM	559	HB	ILE	B	50	65.181	66.079	25.589	1.0	1.53	PROT	H
ATOM	560	HG12	ILE	B	50	65.448	65.496	27.901	1.0	1.40	PROT	H
ATOM	561	HG13	ILE	B	50	66.481	64.100	27.565	1.0	1.44	PROT	H
ATOM	562	HG21	ILE	B	50	65.731	64.463	23.704	1.0	1.47	PROT	H
ATOM	563	HG22	ILE	B	50	66.149	63.218	24.899	1.0	1.54	PROT	H
ATOM	564	HG23	ILE	B	50	64.493	63.825	24.771	1.0	1.60	PROT	H

```
ATOM    565 HD11 ILE B  50      64.244  63.448  28.473  1.0   1.52      PROT H
ATOM    566 HD12 ILE B  50      63.455  64.123  27.033  1.0   1.41      PROT H
ATOM    567 HD13 ILE B  50      64.542  62.737  26.886  1.0   1.43      PROT H
ATOM    568  N   NME B  51      68.419  65.005  23.626  1.0  -6.28      PROT N
ATOM    569  H1  NME B  51      68.652  64.127  24.063  1.0   3.28      PROT H
ATOM    570  H2  NME B  51      68.797  65.124  22.696  1.0   3.23      PROT H
ATOM    571  C   ACE B  52      71.193  81.497  33.664  1.0   4.43      PROT C
ATOM    572  O   ACE B  52      72.102  80.771  34.118  1.0  -6.05      PROT O
ATOM    573  HC  ACE B  52      70.497  81.986  34.382  1.0   1.49      PROT H
ATOM    574  N   LEU B  76      70.980  81.690  32.410  1.0  -4.89      PROT N
ATOM    575  CA  LEU B  76      71.780  81.080  31.410  1.0  -0.56      PROT C
ATOM    576  CB  LEU B  76      71.670  79.520  31.560  1.0  -2.78      PROT C
ATOM    577  CG  LEU B  76      72.270  78.980  32.840  1.0  -0.89      PROT C
ATOM    578  CD1 LEU B  76      72.050  77.560  33.120  1.0  -4.68      PROT C
ATOM    579  CD2 LEU B  76      73.840  79.190  33.020  1.0  -4.22      PROT C
ATOM    580  C   LEU B  76      71.320  81.470  30.040  1.0   6.02      PROT C
ATOM    581  O   LEU B  76      70.200  81.210  29.710  1.0  -6.57      PROT O
ATOM    582  H   LEU B  76      70.078  82.071  32.091  1.0   3.28      PROT H
ATOM    583  HA  LEU B  76      72.867  81.350  31.589  1.0   1.77      PROT H
ATOM    584  HB2 LEU B  76      72.147  79.043  30.682  1.0   1.34      PROT H
ATOM    585  HB3 LEU B  76      70.592  79.249  31.516  1.0   1.61      PROT H
ATOM    586  HG  LEU B  76      71.673  79.340  33.739  1.0   2.20      PROT H
ATOM    587 HD11 LEU B  76      70.983  77.278  33.096  1.0   1.41      PROT H
ATOM    588 HD12 LEU B  76      72.550  76.883  32.408  1.0   1.32      PROT H
ATOM    589 HD13 LEU B  76      72.420  77.274  34.122  1.0   1.68      PROT H
ATOM    590 HD21 LEU B  76      74.120  78.936  34.044  1.0   1.52      PROT H
ATOM    591 HD22 LEU B  76      74.387  78.562  32.319  1.0   1.19      PROT H
ATOM    592 HD23 LEU B  76      74.109  80.232  32.852  1.0   1.22      PROT H
ATOM    593  N   NME B  77      72.211  82.084  29.292  1.0  -6.18      PROT N
ATOM    594  H1  NME B  77      73.138  82.331  29.608  1.0   3.32      PROT H
ATOM    595  H2  NME B  77      72.006  82.384  28.348  1.0   3.18      PROT H
ATOM    596  C   ACE B  78      69.801  77.487  24.713  1.0   4.51      PROT C
ATOM    597  O   ACE B  78      69.792  76.753  25.723  1.0  -6.38      PROT O
ATOM    598  HC  ACE B  78      70.735  78.043  24.477  1.0   1.44      PROT H
ATOM    599  N   THR B  80      68.800  77.640  23.920  1.0  -5.06      PROT N
ATOM    600  CA  THR B  80      67.510  76.940  24.140  1.0  -0.32      PROT C
ATOM    601  CB  THR B  80      66.880  77.270  25.500  1.0   1.54      PROT C
ATOM    602  CG2 THR B  80      66.910  78.740  25.820  1.0  -5.01      PROT C
```

ATOM	603	OG1	THR	B	80	65.620	76.580	25.660	1.0	-6.09	PROT	O
ATOM	604	C	THR	B	80	66.550	77.390	22.960	1.0	5.61	PROT	C
ATOM	605	O	THR	B	80	66.750	78.490	22.440	1.0	-6.47	PROT	O
ATOM	606	H	THR	B	80	68.774	78.330	23.159	1.0	3.48	PROT	H
ATOM	607	HA	THR	B	80	67.687	75.834	24.073	1.0	1.73	PROT	H
ATOM	608	HB	THR	B	80	67.505	76.722	26.278	1.0	1.75	PROT	H
ATOM	609	HG21	THR	B	80	66.269	78.976	26.689	1.0	1.86	PROT	H
ATOM	610	HG22	THR	B	80	67.923	79.079	26.070	1.0	1.65	PROT	H
ATOM	611	HG23	THR	B	80	66.542	79.365	24.995	1.0	1.62	PROT	H
ATOM	612	HG1	THR	B	80	64.974	77.189	26.092	1.0	3.61	PROT	H
ATOM	613	N	PRO	B	81	65.580	76.580	22.550	1.0	-4.67	PROT	N
ATOM	614	CD	PRO	B	81	65.390	75.170	22.810	1.0	-0.80	PROT	C
ATOM	615	CG	PRO	B	81	64.100	74.770	22.030	1.0	-2.65	PROT	C
ATOM	616	CB	PRO	B	81	64.030	75.810	20.900	1.0	-2.79	PROT	C
ATOM	617	CA	PRO	B	81	64.490	77.080	21.650	1.0	0.12	PROT	C
ATOM	618	C	PRO	B	81	63.300	77.800	22.370	1.0	5.51	PROT	C
ATOM	619	O	PRO	B	81	62.410	78.350	21.650	1.0	-6.45	PROT	O
ATOM	620	HA	PRO	B	81	64.914	77.848	20.948	1.0	1.88	PROT	H
ATOM	621	HB2	PRO	B	81	63.026	75.938	20.483	1.0	1.66	PROT	H
ATOM	622	HB3	PRO	B	81	64.705	75.565	20.065	1.0	1.60	PROT	H
ATOM	623	HG2	PRO	B	81	63.213	74.811	22.681	1.0	1.49	PROT	H
ATOM	624	HG3	PRO	B	81	64.180	73.735	21.656	1.0	1.65	PROT	H
ATOM	625	HD2	PRO	B	81	65.276	74.970	23.904	1.0	1.77	PROT	H
ATOM	626	HD3	PRO	B	81	66.261	74.575	22.454	1.0	1.53	PROT	H
ATOM	627	N	VAL	B	82	63.350	77.950	23.680	1.0	-5.10	PROT	N
ATOM	628	CA	VAL	B	82	62.350	78.710	24.520	1.0	-0.27	PROT	C
ATOM	629	CB	VAL	B	82	61.040	78.040	24.870	1.0	-0.84	PROT	C
ATOM	630	CG1	VAL	B	82	60.250	77.680	23.650	1.0	-4.53	PROT	C
ATOM	631	CG2	VAL	B	82	61.210	76.730	25.650	1.0	-4.55	PROT	C
ATOM	632	C	VAL	B	82	62.940	79.300	25.750	1.0	5.67	PROT	C
ATOM	633	O	VAL	B	82	63.940	78.710	26.220	1.0	-6.12	PROT	O
ATOM	634	H	VAL	B	82	64.066	77.480	24.249	1.0	3.44	PROT	H
ATOM	635	HA	VAL	B	82	62.064	79.585	23.825	1.0	1.98	PROT	H
ATOM	636	HB	VAL	B	82	60.441	78.751	25.505	1.0	1.52	PROT	H
ATOM	637	HG11	VAL	B	82	60.761	76.936	23.021	1.0	1.60	PROT	H
ATOM	638	HG12	VAL	B	82	59.265	77.264	23.903	1.0	1.53	PROT	H
ATOM	639	HG13	VAL	B	82	60.069	78.541	22.990	1.0	1.51	PROT	H
ATOM	640	HG21	VAL	B	82	61.824	76.874	26.548	1.0	1.57	PROT	H

ATOM	641	HG22	VAL	B	82	60.241	76.338	25.981	1.0	1.51	PROT	H
ATOM	642	HG23	VAL	B	82	61.692	75.952	25.047	1.0	1.55	PROT	H
ATOM	643	N	ASN	B	83	62.430	80.420	26.360	1.0	-5.31	PROT	N
ATOM	644	CA	ASN	B	83	62.890	80.800	27.670	1.0	0.54	PROT	C
ATOM	645	CB	ASN	B	83	62.430	82.290	27.890	1.0	-3.79	PROT	C
ATOM	646	CG	ASN	B	83	62.880	83.390	26.870	1.0	6.30	PROT	C
ATOM	647	OD1	ASN	B	83	64.010	83.640	26.600	1.0	-6.23	PROT	O
ATOM	648	ND2	ASN	B	83	61.960	84.040	26.150	1.0	-6.60	PROT	N
ATOM	649	C	ASN	B	83	62.130	79.930	28.760	1.0	5.56	PROT	C
ATOM	650	O	ASN	B	83	60.970	79.680	28.580	1.0	-6.07	PROT	O
ATOM	651	H	ASN	B	83	61.530	80.795	26.069	1.0	3.31	PROT	H
ATOM	652	HA	ASN	B	83	64.004	80.691	27.763	1.0	2.10	PROT	H
ATOM	653	HB2	ASN	B	83	62.827	82.618	28.877	1.0	1.91	PROT	H
ATOM	654	HB3	ASN	B	83	61.327	82.310	27.986	1.0	1.72	PROT	H
ATOM	655	HD21	ASN	B	83	60.969	83.947	26.283	1.0	3.16	PROT	H
ATOM	656	HD22	ASN	B	83	62.236	84.749	25.486	1.0	3.24	PROT	H
ATOM	657	N	ILE	B	84	62.840	79.550	29.830	1.0	-5.46	PROT	N
ATOM	658	CA	ILE	B	84	62.380	78.530	30.730	1.0	-0.41	PROT	C
ATOM	659	CB	ILE	B	84	63.000	77.160	30.490	1.0	-1.02	PROT	C
ATOM	660	CG2	ILE	B	84	62.440	76.070	31.510	1.0	-4.46	PROT	C
ATOM	661	CG1	ILE	B	84	62.770	76.740	29.000	1.0	-2.78	PROT	C
ATOM	662	CD1	ILE	B	84	63.350	75.410	28.450	1.0	-4.26	PROT	C
ATOM	663	C	ILE	B	84	62.770	78.910	32.220	1.0	5.63	PROT	C
ATOM	664	O	ILE	B	84	64.000	78.860	32.550	1.0	-6.36	PROT	O
ATOM	665	H	ILE	B	84	63.848	79.779	29.911	1.0	3.67	PROT	H
ATOM	666	HA	ILE	B	84	61.255	78.441	30.651	1.0	1.84	PROT	H
ATOM	667	HB	ILE	B	84	64.103	77.213	30.666	1.0	1.68	PROT	H
ATOM	668	HG12	ILE	B	84	63.180	77.548	28.357	1.0	1.47	PROT	H
ATOM	669	HG13	ILE	B	84	61.676	76.731	28.826	1.0	1.31	PROT	H
ATOM	670	HG21	ILE	B	84	62.863	76.242	32.502	1.0	1.51	PROT	H
ATOM	671	HG22	ILE	B	84	61.353	76.132	31.561	1.0	1.33	PROT	H
ATOM	672	HG23	ILE	B	84	62.716	75.071	31.166	1.0	1.64	PROT	H
ATOM	673	HD11	ILE	B	84	63.112	75.305	27.387	1.0	1.39	PROT	H
ATOM	674	HD12	ILE	B	84	64.441	75.392	28.544	1.0	1.53	PROT	H
ATOM	675	HD13	ILE	B	84	62.937	74.547	28.977	1.0	1.42	PROT	H
ATOM	676	N	NME	B	85	61.770	79.251	33.003	1.0	-5.91	PROT	N
ATOM	677	H1	NME	B	85	60.819	79.300	32.668	1.0	3.30	PROT	H
ATOM	678	H2	NME	B	85	61.923	79.550	33.957	1.0	3.25	PROT	H

HETATM	679	C1	RIT	C	100	67.650	72.420	29.840	1.0	-0.13	PROT	C
HETATM	680	C2	RIT	C	100	66.300	72.270	29.720	1.0	-2.96	PROT	C
HETATM	681	S3	RIT	C	100	65.410	73.290	30.760	1.0	3.52	PROT	S
HETATM	682	C4	RIT	C	100	66.930	73.870	31.390	1.0	-0.81	PROT	C
HETATM	683	N5	RIT	C	100	68.030	73.250	30.840	1.0	-3.78	PROT	N
HETATM	684	C6	RIT	C	100	65.660	71.240	28.850	1.0	0.21	PROT	C
HETATM	685	O7	RIT	C	100	64.710	71.900	27.930	1.0	-4.49	PROT	O
HETATM	686	C10	RIT	C	100	63.950	71.010	27.220	1.0	7.59	PROT	C
HETATM	687	O24	RIT	C	100	64.210	69.850	27.140	1.0	-6.27	PROT	O
HETATM	688	N11	RIT	C	100	62.920	71.660	26.620	1.0	-5.86	PROT	N
HETATM	689	C12	RIT	C	100	61.700	70.980	26.030	1.0	0.97	PROT	C
HETATM	690	C13	RIT	C	100	60.860	71.910	25.070	1.0	1.50	PROT	C
HETATM	691	C14	RIT	C	100	61.570	72.320	23.690	1.0	-3.30	PROT	C
HETATM	692	C15	RIT	C	100	61.270	71.280	22.540	1.0	1.15	PROT	C
HETATM	693	C26	RIT	C	100	60.770	70.390	27.180	1.0	-3.24	PROT	C
HETATM	694	C28	RIT	C	100	61.420	69.240	27.990	1.0	0.55	PROT	C
HETATM	695	C31	RIT	C	100	61.830	69.350	29.310	1.0	-2.09	PROT	C
HETATM	696	C32	RIT	C	100	62.420	68.250	29.950	1.0	-1.71	PROT	C
HETATM	697	C33	RIT	C	100	62.640	67.090	29.330	1.0	-1.81	PROT	C
HETATM	698	C34	RIT	C	100	62.070	66.880	28.030	1.0	-1.16	PROT	C
HETATM	699	C35	RIT	C	100	61.420	67.950	27.400	1.0	-1.70	PROT	C
HETATM	700	O41	RIT	C	100	60.690	73.130	25.810	1.0	-5.87	PROT	O
HETATM	701	C44	RIT	C	100	61.920	71.710	21.130	1.0	-3.25	PROT	C
HETATM	702	C45	RIT	C	100	61.570	70.720	20.010	1.0	0.11	PROT	C
HETATM	703	C48	RIT	C	100	61.840	69.340	20.260	1.0	-1.89	PROT	C
HETATM	704	C49	RIT	C	100	61.740	68.400	19.300	1.0	-1.43	PROT	C
HETATM	705	C50	RIT	C	100	61.300	68.810	18.020	1.0	-1.64	PROT	C
HETATM	706	C51	RIT	C	100	61.060	70.170	17.710	1.0	-1.55	PROT	C
HETATM	707	C52	RIT	C	100	61.180	71.110	18.700	1.0	-1.92	PROT	C
HETATM	708	N58	RIT	C	100	59.840	71.150	22.400	1.0	-6.18	PROT	N
HETATM	709	N20	RIT	C	100	57.850	70.280	20.530	1.0	-6.44	PROT	N
HETATM	710	C19	RIT	C	100	57.980	69.580	21.860	1.0	-0.09	PROT	C
HETATM	711	C18	RIT	C	100	59.150	70.020	22.730	1.0	5.79	PROT	C
HETATM	712	O61	RIT	C	100	59.580	69.350	23.630	1.0	-5.44	PROT	O
HETATM	713	C62	RIT	C	100	57.900	68.000	21.850	1.0	-0.62	PROT	C
HETATM	714	C64	RIT	C	100	59.060	67.240	21.140	1.0	-4.61	PROT	C
HETATM	715	C68	RIT	C	100	56.620	67.480	21.170	1.0	-4.56	PROT	C
HETATM	716	C21	RIT	C	100	57.630	71.620	20.320	1.0	7.57	PROT	C

HETATM	717	N74	RIT	C	100	57.460	71.990	19.010	1.0	-5.31	PROT	N
HETATM	718	C75	RIT	C	100	57.390	73.410	18.680	1.0	-0.34	PROT	C
HETATM	719	O76	RIT	C	100	57.680	72.460	21.180	1.0	-6.86	PROT	O
HETATM	720	C77	RIT	C	100	58.780	73.950	18.670	1.0	0.96	PROT	C
HETATM	721	C80	RIT	C	100	59.360	74.510	19.760	1.0	-3.40	PROT	C
HETATM	722	S81	RIT	C	100	60.840	75.360	19.390	1.0	1.91	PROT	S
HETATM	723	C82	RIT	C	100	60.480	75.010	17.680	1.0	1.09	PROT	C
HETATM	724	N83	RIT	C	100	59.480	74.220	17.510	1.0	-4.49	PROT	N
HETATM	725	C85	RIT	C	100	61.340	75.330	16.510	1.0	-0.87	PROT	C
HETATM	726	C86	RIT	C	100	62.080	76.660	16.720	1.0	-4.58	PROT	C
HETATM	727	C90	RIT	C	100	62.390	74.150	16.240	1.0	-4.29	PROT	C
HETATM	728	C95	RIT	C	100	57.390	70.990	17.870	1.0	-2.11	PROT	C
HETATM	729	H1	RIT	C	100	68.419	71.909	29.262	1.0	1.82	PROT	H
HETATM	730	H4	RIT	C	100	67.046	74.613	32.169	1.0	2.07	PROT	H
HETATM	731	H61	RIT	C	100	65.088	70.496	29.442	1.0	1.55	PROT	H
HETATM	732	H62	RIT	C	100	66.395	70.707	28.214	1.0	1.54	PROT	H
HETATM	733	H11	RIT	C	100	62.782	72.656	26.789	1.0	3.39	PROT	H
HETATM	734	H12	RIT	C	100	62.074	70.115	25.423	1.0	1.58	PROT	H
HETATM	735	H13	RIT	C	100	59.876	71.441	24.880	1.0	1.55	PROT	H
HETATM	736	H141	RIT	C	100	62.655	72.402	23.847	1.0	1.64	PROT	H
HETATM	737	H142	RIT	C	100	61.198	73.314	23.407	1.0	1.53	PROT	H
HETATM	738	H15	RIT	C	100	61.720	70.298	22.842	1.0	1.52	PROT	H
HETATM	739	H261	RIT	C	100	59.835	70.020	26.716	1.0	1.80	PROT	H
HETATM	740	H262	RIT	C	100	60.476	71.216	27.850	1.0	1.69	PROT	H
HETATM	741	H31	RIT	C	100	61.756	70.303	29.829	1.0	1.49	PROT	H
HETATM	742	H32	RIT	C	100	62.770	68.398	30.982	1.0	1.50	PROT	H
HETATM	743	H33	RIT	C	100	63.175	66.263	29.796	1.0	1.52	PROT	H
HETATM	744	H34	RIT	C	100	62.144	65.910	27.555	1.0	1.50	PROT	H
HETATM	745	H35	RIT	C	100	60.976	67.810	26.416	1.0	1.64	PROT	H
HETATM	746	H41	RIT	C	100	59.909	73.610	25.458	1.0	3.37	PROT	H
HETATM	747	H441	RIT	C	100	63.018	71.751	21.247	1.0	1.77	PROT	H
HETATM	748	H442	RIT	C	100	61.583	72.724	20.874	1.0	1.55	PROT	H
HETATM	749	H48	RIT	C	100	62.163	69.060	21.269	1.0	1.66	PROT	H
HETATM	750	H49	RIT	C	100	61.960	67.349	19.477	1.0	1.56	PROT	H
HETATM	751	H50	RIT	C	100	61.179	68.065	17.237	1.0	1.54	PROT	H
HETATM	752	H51	RIT	C	100	60.771	70.447	16.698	1.0	1.58	PROT	H
HETATM	753	H52	RIT	C	100	60.991	72.162	18.495	1.0	1.60	PROT	H
HETATM	754	H58	RIT	C	100	59.427	71.803	21.743	1.0	3.20	PROT	H

```
HETATM  755  H20 RIT C 100      58.030  69.656  19.751  1.0   3.28      PROT H
HETATM  756  H19 RIT C 100      57.054  69.891  22.447  1.0   1.77      PROT H
HETATM  757  H10 RIT C 100      57.895  67.714  22.935  1.0   1.82      PROT H
HETATM  758 H641 RIT C 100      59.105  67.474  20.074  1.0   1.43      PROT H
HETATM  759 H642 RIT C 100      58.933  66.159  21.251  1.0   1.60      PROT H
HETATM  760 H643 RIT C 100      60.027  67.511  21.582  1.0   1.55      PROT H
HETATM  761 H681 RIT C 100      56.530  66.394  21.292  1.0   1.57      PROT H
HETATM  762 H682 RIT C 100      56.608  67.696  20.098  1.0   1.43      PROT H
HETATM  763 H683 RIT C 100      55.719  67.927  21.608  1.0   1.51      PROT H
HETATM  764 H751 RIT C 100      56.768  73.955  19.437  1.0   1.79      PROT H
HETATM  765 H752 RIT C 100      56.896  73.556  17.688  1.0   1.68      PROT H
HETATM  766  H80 RIT C 100      58.982  74.464  20.774  1.0   2.07      PROT H
HETATM  767  H85 RIT C 100      60.702  75.406  15.592  1.0   1.66      PROT H
HETATM  768 H861 RIT C 100      62.603  76.960  15.803  1.0   1.56      PROT H
HETATM  769 H862 RIT C 100      62.841  76.597  17.507  1.0   1.62      PROT H
HETATM  770 H863 RIT C 100      61.389  77.473  16.974  1.0   1.58      PROT H
HETATM  771 H901 RIT C 100      61.865  73.197  16.149  1.0   1.50      PROT H
HETATM  772 H902 RIT C 100      63.112  74.081  17.055  1.0   1.55      PROT H
HETATM  773 H903 RIT C 100      62.924  74.348  15.309  1.0   1.52      PROT H
HETATM  774 H951 RIT C 100      57.220  71.523  16.921  1.0   1.69      PROT H
HETATM  775 H952 RIT C 100      56.563  70.285  18.042  1.0   1.58      PROT H
HETATM  776 H953 RIT C 100      58.350  70.455  17.776  1.0   1.55      PROT H
HETATM  777  OW  SOL D3735      54.090  72.930  17.970  1.0  -7.10      PROT O
HETATM  778  HW1 SOL D3735      54.974  72.571  18.002  1.0   3.45      PROT H
HETATM  779  HW2 SOL D3735      53.934  73.177  17.056  1.0   3.62      PROT H
HETATM  780  OW  SOL D6587      57.740  74.040  23.700  1.0  -6.88      PROT O
HETATM  781  HW1 SOL D6587      58.150  73.540  22.984  1.0   3.51      PROT H
HETATM  782  HW2 SOL D6587      56.907  73.586  23.818  1.0   3.31      PROT H
HETATM  783  OW  SOL D8852      68.060  69.470  31.020  1.0  -7.16      PROT O
HETATM  784  HW1 SOL D8852      68.928  69.507  30.599  1.0   3.61      PROT H
HETATM  785  HW2 SOL D8852      67.870  70.376  31.263  1.0   3.44      PROT H
HETATM  786  OW  SOL D 765      65.840  68.200  28.550  1.0  -7.47      PROT O
HETATM  787  HW1 SOL D 765      65.212  68.751  28.062  1.0   3.78      PROT H
HETATM  788  HW2 SOL D 765      65.358  67.827  29.286  1.0   3.57      PROT H
END
```

4.6 SAQ-HIV^{Pro}

```
HEADER  data-set: HIV_SAQ_full_wH2O_OPT
REMARK  MOPAC, Version: 23.1.2
REMARK  99
REMARK  99 MOE v2014.09 (Chemical Computing Group Inc.)
ATOM      1  C   ACE A   7      68.264  50.388  27.413  1.0   4.47      PROT C
ATOM      2  O   ACE A   7      68.753  50.898  26.383  1.0  -6.38      PROT O
ATOM      3  HC  ACE A   7      68.918  49.740  28.039  1.0   1.51      PROT H
ATOM      4  N   ARG A   8      67.050  50.560  27.800  1.0  -4.94      PROT N
ATOM      5  CA  ARG A   8      66.110  51.390  27.050  1.0  -0.21      PROT C
ATOM      6  CB  ARG A   8      65.910  50.770  25.640  1.0  -2.88      PROT C
ATOM      7  CG  ARG A   8      67.160  50.680  24.800  1.0  -2.72      PROT C
ATOM      8  CD  ARG A   8      66.940  50.050  23.400  1.0  -0.77      PROT C
ATOM      9  NE  ARG A   8      67.930  50.560  22.420  1.0  -5.70      PROT N
ATOM     10  CZ  ARG A   8      68.350  49.970  21.280  1.0   6.25      PROT C
ATOM     11  NH1 ARG A   8      68.020  48.710  21.060  1.0  -6.19      PROT N
ATOM     12  NH2 ARG A   8      69.140  50.670  20.490  1.0  -6.32      PROT N
ATOM     13  C   ARG A   8      64.740  51.430  27.720  1.0   5.63      PROT C
ATOM     14  O   ARG A   8      64.390  50.410  28.330  1.0  -6.48      PROT O
ATOM     15  H   ARG A   8      66.636  50.060  28.596  1.0   3.41      PROT H
ATOM     16  HA  ARG A   8      66.559  52.421  26.941  1.0   1.93      PROT H
ATOM     17  HB2 ARG A   8      65.140  51.363  25.098  1.0   1.50      PROT H
ATOM     18  HB3 ARG A   8      65.461  49.759  25.761  1.0   1.55      PROT H
ATOM     19  HG2 ARG A   8      67.879  49.931  25.214  1.0   1.88      PROT H
ATOM     20  HG3 ARG A   8      67.666  51.667  24.713  1.0   1.97      PROT H
ATOM     21  HD2 ARG A   8      65.922  50.299  23.011  1.0   1.51      PROT H
ATOM     22  HD3 ARG A   8      66.992  48.944  23.483  1.0   1.32      PROT H
ATOM     23  HE  ARG A   8      68.437  51.445  22.730  1.0   4.00      PROT H
ATOM     24 HH11 ARG A   8      67.362  48.211  21.645  1.0   3.41      PROT H
ATOM     25 HH12 ARG A   8      68.218  48.245  20.183  1.0   3.36      PROT H
ATOM     26 HH21 ARG A   8      69.408  51.689  20.707  1.0   4.14      PROT H
ATOM     27 HH22 ARG A   8      69.628  50.261  19.705  1.0   3.41      PROT H
ATOM     28  N   NME A   9      64.068  52.552  27.579  1.0  -5.97      PROT N
ATOM     29  H1  NME A   9      64.436  53.368  27.107  1.0   3.34      PROT H
ATOM     30  H2  NME A   9      63.150  52.682  27.983  1.0   3.25      PROT H
ATOM     31  C   ACE A  10      59.289  51.376  22.618  1.0   4.87      PROT C
ATOM     32  O   ACE A  10      60.467  50.965  22.569  1.0  -6.77      PROT O
```

ATOM	33	HC	ACE	A	10	58.489	50.714	22.217	1.0	1.52	PROT	H
ATOM	34	N	LEU	A	23	58.940	52.520	23.090	1.0	-4.76	PROT	N
ATOM	35	CA	LEU	A	23	59.890	53.440	23.610	1.0	-0.37	PROT	C
ATOM	36	CB	LEU	A	23	60.990	53.690	22.540	1.0	-3.22	PROT	C
ATOM	37	CG	LEU	A	23	62.200	54.460	23.020	1.0	-0.75	PROT	C
ATOM	38	CD1	LEU	A	23	63.170	53.510	23.750	1.0	-4.55	PROT	C
ATOM	39	CD2	LEU	A	23	62.910	54.950	21.790	1.0	-4.47	PROT	C
ATOM	40	C	LEU	A	23	59.320	54.770	24.140	1.0	5.87	PROT	C
ATOM	41	O	LEU	A	23	58.870	55.550	23.310	1.0	-6.54	PROT	O
ATOM	42	H	LEU	A	23	58.006	52.909	22.895	1.0	3.35	PROT	H
ATOM	43	HA	LEU	A	23	60.419	52.924	24.480	1.0	1.67	PROT	H
ATOM	44	HB2	LEU	A	23	60.504	54.207	21.681	1.0	1.65	PROT	H
ATOM	45	HB3	LEU	A	23	61.327	52.717	22.111	1.0	2.05	PROT	H
ATOM	46	HG	LEU	A	23	61.915	55.300	23.691	1.0	1.27	PROT	H
ATOM	47	HD11	LEU	A	23	62.715	53.083	24.647	1.0	1.25	PROT	H
ATOM	48	HD12	LEU	A	23	63.467	52.673	23.107	1.0	1.56	PROT	H
ATOM	49	HD13	LEU	A	23	64.085	54.038	24.049	1.0	1.60	PROT	H
ATOM	50	HD21	LEU	A	23	63.854	55.460	22.040	1.0	1.58	PROT	H
ATOM	51	HD22	LEU	A	23	63.179	54.123	21.114	1.0	1.56	PROT	H
ATOM	52	HD23	LEU	A	23	62.301	55.650	21.202	1.0	1.38	PROT	H
ATOM	53	N	LEU	A	24	59.550	55.120	25.400	1.0	-5.44	PROT	N
ATOM	54	CA	LEU	A	24	59.230	56.530	25.820	1.0	0.38	PROT	C
ATOM	55	CB	LEU	A	24	59.030	56.540	27.290	1.0	-3.43	PROT	C
ATOM	56	CG	LEU	A	24	57.900	55.640	27.790	1.0	-0.57	PROT	C
ATOM	57	CD1	LEU	A	24	57.950	55.430	29.340	1.0	-4.59	PROT	C
ATOM	58	CD2	LEU	A	24	56.520	56.120	27.530	1.0	-4.66	PROT	C
ATOM	59	C	LEU	A	24	60.420	57.420	25.430	1.0	5.60	PROT	C
ATOM	60	O	LEU	A	24	61.490	57.380	26.070	1.0	-6.53	PROT	O
ATOM	61	H	LEU	A	24	59.949	54.514	26.107	1.0	3.43	PROT	H
ATOM	62	HA	LEU	A	24	58.293	56.850	25.275	1.0	1.91	PROT	H
ATOM	63	HB2	LEU	A	24	58.844	57.584	27.644	1.0	1.79	PROT	H
ATOM	64	HB3	LEU	A	24	59.975	56.261	27.819	1.0	1.77	PROT	H
ATOM	65	HG	LEU	A	24	58.031	54.624	27.328	1.0	1.34	PROT	H
ATOM	66	HD11	LEU	A	24	57.170	54.736	29.663	1.0	1.47	PROT	H
ATOM	67	HD12	LEU	A	24	58.913	55.017	29.653	1.0	1.43	PROT	H
ATOM	68	HD13	LEU	A	24	57.805	56.376	29.870	1.0	1.49	PROT	H
ATOM	69	HD21	LEU	A	24	56.310	57.087	28.012	1.0	1.54	PROT	H
ATOM	70	HD22	LEU	A	24	56.314	56.262	26.457	1.0	1.50	PROT	H

ATOM	71	HD23	LEU	A	24	55.756	55.417	27.893	1.0	1.50	PROT	H
ATOM	72	N	ASH	A	25	60.320	58.180	24.370	1.0	-4.84	PROT	N
ATOM	73	CA	ASH	A	25	61.360	58.960	23.770	1.0	0.02	PROT	C
ATOM	74	CB	ASH	A	25	61.390	58.610	22.280	1.0	-3.40	PROT	C
ATOM	75	CG	ASH	A	25	62.440	59.410	21.390	1.0	6.61	PROT	C
ATOM	76	OD1	ASH	A	25	63.110	60.370	21.700	1.0	-5.55	PROT	O
ATOM	77	OD2	ASH	A	25	62.600	58.870	20.180	1.0	-5.44	PROT	O
ATOM	78	C	ASH	A	25	61.280	60.500	23.940	1.0	5.36	PROT	C
ATOM	79	O	ASH	A	25	60.670	61.240	23.140	1.0	-5.62	PROT	O
ATOM	80	H	ASH	A	25	59.422	58.213	23.852	1.0	3.44	PROT	H
ATOM	81	HA	ASH	A	25	62.360	58.640	24.222	1.0	1.89	PROT	H
ATOM	82	HB2	ASH	A	25	60.396	58.813	21.811	1.0	2.09	PROT	H
ATOM	83	HB3	ASH	A	25	61.598	57.527	22.140	1.0	1.95	PROT	H
ATOM	84	HD2	ASH	A	25	63.380	59.291	19.691	1.0	3.79	PROT	H
ATOM	85	N	THR	A	26	62.000	61.060	24.920	1.0	-5.64	PROT	N
ATOM	86	CA	THR	A	26	62.090	62.530	25.260	1.0	-0.40	PROT	C
ATOM	87	CB	THR	A	26	62.940	62.840	26.410	1.0	1.69	PROT	C
ATOM	88	CG2	THR	A	26	62.280	62.240	27.730	1.0	-4.87	PROT	C
ATOM	89	OG1	THR	A	26	64.240	62.340	26.240	1.0	-6.44	PROT	O
ATOM	90	C	THR	A	26	62.440	63.400	24.050	1.0	6.01	PROT	C
ATOM	91	O	THR	A	26	61.910	64.510	24.000	1.0	-5.84	PROT	O
ATOM	92	H	THR	A	26	62.622	60.499	25.510	1.0	3.44	PROT	H
ATOM	93	HA	THR	A	26	61.009	62.827	25.515	1.0	2.11	PROT	H
ATOM	94	HB	THR	A	26	63.013	63.954	26.553	1.0	1.49	PROT	H
ATOM	95	HG21	THR	A	26	62.840	62.576	28.605	1.0	1.54	PROT	H
ATOM	96	HG22	THR	A	26	61.243	62.559	27.825	1.0	1.59	PROT	H
ATOM	97	HG23	THR	A	26	62.330	61.147	27.711	1.0	1.65	PROT	H
ATOM	98	HG1	THR	A	26	64.932	63.021	26.439	1.0	3.77	PROT	H
ATOM	99	N	GLY	A	27	63.310	62.820	23.200	1.0	-5.58	PROT	N
ATOM	100	CA	GLY	A	27	64.000	63.480	22.020	1.0	-1.43	PROT	C
ATOM	101	C	GLY	A	27	63.160	63.720	20.860	1.0	5.53	PROT	C
ATOM	102	O	GLY	A	27	63.340	64.760	20.170	1.0	-6.07	PROT	O
ATOM	103	H	GLY	A	27	63.515	61.828	23.262	1.0	3.43	PROT	H
ATOM	104	HA2	GLY	A	27	64.870	62.824	21.751	1.0	1.88	PROT	H
ATOM	105	HA3	GLY	A	27	64.424	64.456	22.385	1.0	1.91	PROT	H
ATOM	106	N	ALA	A	28	62.170	62.870	20.510	1.0	-5.47	PROT	N
ATOM	107	CA	ALA	A	28	61.180	63.010	19.460	1.0	0.33	PROT	C
ATOM	108	CB	ALA	A	28	60.830	61.700	18.880	1.0	-4.86	PROT	C

ATOM	109	C	ALA	A	28	59.930	63.700	19.960	1.0	5.44	PROT	C
ATOM	110	O	ALA	A	28	59.260	63.310	20.910	1.0	-5.84	PROT	O
ATOM	111	H	ALA	A	28	61.922	62.137	21.200	1.0	3.39	PROT	H
ATOM	112	HA	ALA	A	28	61.669	63.663	18.654	1.0	1.76	PROT	H
ATOM	113	HB1	ALA	A	28	60.320	61.793	17.903	1.0	1.84	PROT	H
ATOM	114	HB2	ALA	A	28	61.694	61.040	18.730	1.0	1.46	PROT	H
ATOM	115	HB3	ALA	A	28	60.123	61.126	19.514	1.0	1.89	PROT	H
ATOM	116	N	ASP	A	29	59.570	64.720	19.230	1.0	-5.49	PROT	N
ATOM	117	CA	ASP	A	29	58.360	65.480	19.260	1.0	0.28	PROT	C
ATOM	118	CB	ASP	A	29	58.520	66.660	18.290	1.0	-4.34	PROT	C
ATOM	119	CG	ASP	A	29	59.920	67.250	18.380	1.0	7.60	PROT	C
ATOM	120	OD1	ASP	A	29	60.220	68.060	19.240	1.0	-7.64	PROT	O
ATOM	121	OD2	ASP	A	29	60.760	66.860	17.620	1.0	-8.25	PROT	O
ATOM	122	C	ASP	A	29	57.120	64.790	18.790	1.0	5.88	PROT	C
ATOM	123	O	ASP	A	29	56.000	65.280	18.970	1.0	-6.29	PROT	O
ATOM	124	H	ASP	A	29	60.195	64.991	18.429	1.0	3.54	PROT	H
ATOM	125	HA	ASP	A	29	58.175	65.858	20.318	1.0	2.09	PROT	H
ATOM	126	HB2	ASP	A	29	57.772	67.448	18.522	1.0	1.95	PROT	H
ATOM	127	HB3	ASP	A	29	58.320	66.359	17.239	1.0	1.87	PROT	H
ATOM	128	N	ASP	A	30	57.240	63.600	18.200	1.0	-5.24	PROT	N
ATOM	129	CA	ASP	A	30	56.270	62.750	17.520	1.0	0.44	PROT	C
ATOM	130	CB	ASP	A	30	56.350	62.780	16.000	1.0	-4.53	PROT	C
ATOM	131	CG	ASP	A	30	56.940	63.970	15.330	1.0	7.88	PROT	C
ATOM	132	OD1	ASP	A	30	58.150	64.030	15.090	1.0	-8.32	PROT	O
ATOM	133	OD2	ASP	A	30	56.140	64.850	14.890	1.0	-8.05	PROT	O
ATOM	134	C	ASP	A	30	56.210	61.220	17.860	1.0	5.49	PROT	C
ATOM	135	O	ASP	A	30	57.180	60.600	18.190	1.0	-5.82	PROT	O
ATOM	136	H	ASP	A	30	58.188	63.224	18.072	1.0	3.08	PROT	H
ATOM	137	HA	ASP	A	30	55.254	63.186	17.818	1.0	1.92	PROT	H
ATOM	138	HB2	ASP	A	30	55.321	62.636	15.585	1.0	1.89	PROT	H
ATOM	139	HB3	ASP	A	30	56.931	61.896	15.632	1.0	1.74	PROT	H
ATOM	140	N	THR	A	31	55.030	60.650	17.860	1.0	-5.33	PROT	N
ATOM	141	CA	THR	A	31	54.720	59.250	17.980	1.0	-0.88	PROT	C
ATOM	142	CB	THR	A	31	53.420	59.160	18.770	1.0	1.92	PROT	C
ATOM	143	CG2	THR	A	31	52.920	57.700	18.780	1.0	-4.98	PROT	C
ATOM	144	OG1	THR	A	31	53.600	59.570	20.090	1.0	-6.07	PROT	O
ATOM	145	C	THR	A	31	54.730	58.560	16.650	1.0	5.44	PROT	C
ATOM	146	O	THR	A	31	54.040	59.040	15.690	1.0	-6.14	PROT	O

```
ATOM    147  H   THR A  31      54.222  61.181  17.476  1.0   3.28      PROT H
ATOM    148  HA  THR A  31      55.532  58.773  18.647  1.0   2.18      PROT H
ATOM    149  HB  THR A  31      52.626  59.827  18.340  1.0   1.41      PROT H
ATOM    150 HG21 THR A  31      52.088  57.579  19.481  1.0   1.58      PROT H
ATOM    151 HG22 THR A  31      52.578  57.390  17.788  1.0   1.63      PROT H
ATOM    152 HG23 THR A  31      53.712  57.010  19.104  1.0   1.72      PROT H
ATOM    153  HG1 THR A  31      54.244  60.312  20.153  1.0   3.57      PROT H
ATOM    154  N   VAL A  32      55.440  57.440  16.570  1.0  -5.31      PROT N
ATOM    155  CA  VAL A  32      55.630  56.600  15.400  1.0  -0.34      PROT C
ATOM    156  CB  VAL A  32      57.110  56.730  15.020  1.0  -0.63      PROT C
ATOM    157  CG1 VAL A  32      57.470  55.510  14.200  1.0  -4.74      PROT C
ATOM    158  CG2 VAL A  32      57.430  58.040  14.260  1.0  -4.40      PROT C
ATOM    159  C   VAL A  32      55.180  55.190  15.780  1.0   5.95      PROT C
ATOM    160  O   VAL A  32      55.420  54.770  16.900  1.0  -6.56      PROT O
ATOM    161  H   VAL A  32      55.876  57.046  17.429  1.0   3.58      PROT H
ATOM    162  HA  VAL A  32      54.991  56.993  14.546  1.0   2.01      PROT H
ATOM    163  HB  VAL A  32      57.730  56.727  15.957  1.0   1.45      PROT H
ATOM    164 HG11 VAL A  32      57.422  54.581  14.783  1.0   1.54      PROT H
ATOM    165 HG12 VAL A  32      56.829  55.391  13.318  1.0   1.50      PROT H
ATOM    166 HG13 VAL A  32      58.502  55.565  13.823  1.0   1.59      PROT H
ATOM    167 HG21 VAL A  32      58.507  58.141  14.104  1.0   1.43      PROT H
ATOM    168 HG22 VAL A  32      56.939  58.062  13.282  1.0   1.48      PROT H
ATOM    169 HG23 VAL A  32      57.081  58.912  14.827  1.0   1.59      PROT H
ATOM    170  N   NME A  33      54.550  54.519  14.841  1.0  -6.17      PROT N
ATOM    171  H1  NME A  33      54.383  54.879  13.907  1.0   3.45      PROT H
ATOM    172  H2  NME A  33      54.250  53.565  14.992  1.0   3.18      PROT H
ATOM    173  C   ACE A  34      57.509  62.527   6.214  1.0   4.38      PROT C
ATOM    174  O   ACE A  34      56.899  63.195   7.074  1.0  -6.30      PROT O
ATOM    175  HC  ACE A  34      56.966  62.224   5.291  1.0   1.41      PROT H
ATOM    176  N   ILE A  47      58.730  62.140   6.330  1.0  -5.02      PROT N
ATOM    177  CA  ILE A  47      59.540  62.460   7.520  1.0  -0.28      PROT C
ATOM    178  CB  ILE A  47      58.770  61.990   8.790  1.0  -0.88      PROT C
ATOM    179  CG2 ILE A  47      57.520  62.840   8.940  1.0  -3.97      PROT C
ATOM    180  CG1 ILE A  47      58.510  60.490   8.850  1.0  -2.88      PROT C
ATOM    181  CD1 ILE A  47      58.230  59.830  10.280  1.0  -4.23      PROT C
ATOM    182  C   ILE A  47      60.970  61.880   7.410  1.0   5.51      PROT C
ATOM    183  O   ILE A  47      61.230  60.790   6.880  1.0  -6.32      PROT O
ATOM    184  H   ILE A  47      59.186  61.507   5.666  1.0   3.38      PROT H
```

ATOM	185	HA	ILE	A	47	59.628	63.588	7.575	1.0	1.82	PROT	H
ATOM	186	HB	ILE	A	47	59.452	62.233	9.658	1.0	1.42	PROT	H
ATOM	187	HG12	ILE	A	47	59.373	59.948	8.416	1.0	1.42	PROT	H
ATOM	188	HG13	ILE	A	47	57.648	60.236	8.205	1.0	1.41	PROT	H
ATOM	189	HG21	ILE	A	47	57.642	63.886	8.626	1.0	1.66	PROT	H
ATOM	190	HG22	ILE	A	47	56.588	62.332	8.646	1.0	1.65	PROT	H
ATOM	191	HG23	ILE	A	47	57.330	62.964	10.035	1.0	1.25	PROT	H
ATOM	192	HD11	ILE	A	47	57.947	58.786	10.143	1.0	1.44	PROT	H
ATOM	193	HD12	ILE	A	47	59.127	59.876	10.896	1.0	1.37	PROT	H
ATOM	194	HD13	ILE	A	47	57.416	60.358	10.782	1.0	1.54	PROT	H
ATOM	195	N	GLY	A	48	61.990	62.510	8.030	1.0	-5.19	PROT	N
ATOM	196	CA	GLY	A	48	63.370	62.020	7.940	1.0	-1.73	PROT	C
ATOM	197	C	GLY	A	48	64.440	62.700	8.780	1.0	5.96	PROT	C
ATOM	198	O	GLY	A	48	64.890	63.790	8.470	1.0	-5.97	PROT	O
ATOM	199	H	GLY	A	48	61.879	63.425	8.464	1.0	3.34	PROT	H
ATOM	200	HA2	GLY	A	48	63.708	62.096	6.865	1.0	1.91	PROT	H
ATOM	201	HA3	GLY	A	48	63.354	60.911	8.133	1.0	1.85	PROT	H
ATOM	202	N	GLY	A	49	64.800	62.030	9.880	1.0	-5.60	PROT	N
ATOM	203	CA	GLY	A	49	65.600	62.650	10.990	1.0	-1.48	PROT	C
ATOM	204	C	GLY	A	49	67.110	62.500	10.910	1.0	5.72	PROT	C
ATOM	205	O	GLY	A	49	67.790	63.510	11.090	1.0	-6.31	PROT	O
ATOM	206	H	GLY	A	49	64.600	61.034	10.018	1.0	3.55	PROT	H
ATOM	207	HA2	GLY	A	49	65.225	62.216	11.949	1.0	1.77	PROT	H
ATOM	208	HA3	GLY	A	49	65.372	63.748	11.010	1.0	1.96	PROT	H
ATOM	209	N	ILE	A	50	67.600	61.370	10.430	1.0	-5.37	PROT	N
ATOM	210	CA	ILE	A	50	68.960	60.880	10.270	1.0	-0.20	PROT	C
ATOM	211	CB	ILE	A	50	69.250	59.770	11.310	1.0	-1.07	PROT	C
ATOM	212	CG2	ILE	A	50	70.500	58.870	10.990	1.0	-4.44	PROT	C
ATOM	213	CG1	ILE	A	50	69.430	60.310	12.710	1.0	-2.63	PROT	C
ATOM	214	CD1	ILE	A	50	70.520	61.320	12.970	1.0	-4.49	PROT	C
ATOM	215	C	ILE	A	50	69.070	60.360	8.830	1.0	5.85	PROT	C
ATOM	216	O	ILE	A	50	68.330	59.510	8.430	1.0	-6.10	PROT	O
ATOM	217	H	ILE	A	50	66.922	60.598	10.232	1.0	3.42	PROT	H
ATOM	218	HA	ILE	A	50	69.682	61.731	10.445	1.0	1.83	PROT	H
ATOM	219	HB	ILE	A	50	68.364	59.079	11.299	1.0	1.44	PROT	H
ATOM	220	HG12	ILE	A	50	69.569	59.446	13.404	1.0	1.47	PROT	H
ATOM	221	HG13	ILE	A	50	68.459	60.753	13.041	1.0	1.29	PROT	H
ATOM	222	HG21	ILE	A	50	70.376	58.350	10.035	1.0	1.51	PROT	H

ATOM	223	HG22	ILE	A	50	71.415	59.465	10.936	1.0	1.55	PROT	H
ATOM	224	HG23	ILE	A	50	70.637	58.111	11.764	1.0	1.54	PROT	H
ATOM	225	HD11	ILE	A	50	71.510	60.948	12.674	1.0	1.53	PROT	H
ATOM	226	HD12	ILE	A	50	70.359	62.255	12.415	1.0	1.53	PROT	H
ATOM	227	HD13	ILE	A	50	70.571	61.597	14.031	1.0	1.46	PROT	H
ATOM	228	N	NME	A	51	70.014	60.907	8.095	1.0	-6.27	PROT	N
ATOM	229	H1	NME	A	51	70.629	61.640	8.416	1.0	3.30	PROT	H
ATOM	230	H2	NME	A	51	70.163	60.630	7.133	1.0	3.25	PROT	H
ATOM	231	C	ACE	A	52	51.502	59.640	14.102	1.0	4.65	PROT	C
ATOM	232	O	ACE	A	52	51.666	60.740	13.536	1.0	-6.40	PROT	O
ATOM	233	HC	ACE	A	52	51.017	59.628	15.104	1.0	1.45	PROT	H
ATOM	234	N	LEU	A	76	51.870	58.510	13.610	1.0	-4.99	PROT	N
ATOM	235	CA	LEU	A	76	52.540	58.410	12.290	1.0	-0.09	PROT	C
ATOM	236	CB	LEU	A	76	53.900	59.170	12.360	1.0	-3.05	PROT	C
ATOM	237	CG	LEU	A	76	53.820	60.650	12.760	1.0	-0.20	PROT	C
ATOM	238	CD1	LEU	A	76	55.190	61.250	12.760	1.0	-4.74	PROT	C
ATOM	239	CD2	LEU	A	76	52.950	61.480	11.670	1.0	-4.24	PROT	C
ATOM	240	C	LEU	A	76	52.890	56.930	11.910	1.0	5.58	PROT	C
ATOM	241	O	LEU	A	76	53.690	56.280	12.660	1.0	-6.73	PROT	O
ATOM	242	H	LEU	A	76	51.985	57.676	14.193	1.0	3.32	PROT	H
ATOM	243	HA	LEU	A	76	51.874	58.879	11.524	1.0	1.66	PROT	H
ATOM	244	HB2	LEU	A	76	54.407	59.076	11.382	1.0	1.38	PROT	H
ATOM	245	HB3	LEU	A	76	54.543	58.645	13.104	1.0	1.70	PROT	H
ATOM	246	HG	LEU	A	76	53.467	60.783	13.805	1.0	1.76	PROT	H
ATOM	247	HD11	LEU	A	76	55.162	62.311	13.054	1.0	1.61	PROT	H
ATOM	248	HD12	LEU	A	76	55.858	60.742	13.470	1.0	1.41	PROT	H
ATOM	249	HD13	LEU	A	76	55.677	61.212	11.777	1.0	1.35	PROT	H
ATOM	250	HD21	LEU	A	76	52.664	62.436	12.105	1.0	1.42	PROT	H
ATOM	251	HD22	LEU	A	76	53.547	61.642	10.775	1.0	1.20	PROT	H
ATOM	252	HD23	LEU	A	76	52.048	60.940	11.396	1.0	1.29	PROT	H
ATOM	253	N	NME	A	77	52.315	56.469	10.821	1.0	-5.85	PROT	N
ATOM	254	H1	NME	A	77	51.638	56.991	10.282	1.0	3.33	PROT	H
ATOM	255	H2	NME	A	77	52.484	55.531	10.482	1.0	3.22	PROT	H
ATOM	256	C	ACE	A	78	58.502	52.145	10.124	1.0	4.62	PROT	C
ATOM	257	O	ACE	A	78	58.664	53.361	10.359	1.0	-6.67	PROT	O
ATOM	258	HC	ACE	A	78	57.788	51.860	9.320	1.0	1.52	PROT	H
ATOM	259	N	THR	A	80	59.110	51.200	10.750	1.0	-4.84	PROT	N
ATOM	260	CA	THR	A	80	60.060	51.470	11.810	1.0	-0.65	PROT	C

ATOM	261	CB	THR	A	80	59.320	52.150	12.980	1.0	1.61	PROT	C
ATOM	262	CG2	THR	A	80	58.220	51.360	13.680	1.0	-5.03	PROT	C
ATOM	263	OG1	THR	A	80	60.190	52.620	14.040	1.0	-6.48	PROT	O
ATOM	264	C	THR	A	80	60.660	50.150	12.410	1.0	5.77	PROT	C
ATOM	265	O	THR	A	80	60.090	49.090	12.440	1.0	-6.01	PROT	O
ATOM	266	H	THR	A	80	58.912	50.202	10.595	1.0	3.48	PROT	H
ATOM	267	HA	THR	A	80	60.862	52.156	11.426	1.0	1.89	PROT	H
ATOM	268	HB	THR	A	80	58.903	53.122	12.583	1.0	1.69	PROT	H
ATOM	269	HG21	THR	A	80	58.607	50.446	14.150	1.0	1.65	PROT	H
ATOM	270	HG22	THR	A	80	57.746	51.961	14.470	1.0	1.74	PROT	H
ATOM	271	HG23	THR	A	80	57.427	51.050	12.989	1.0	1.61	PROT	H
ATOM	272	HG1	THR	A	80	59.784	52.513	14.930	1.0	3.78	PROT	H
ATOM	273	N	PRO	A	81	61.900	50.280	12.910	1.0	-4.69	PROT	N
ATOM	274	CD	PRO	A	81	62.800	51.380	12.690	1.0	-0.77	PROT	C
ATOM	275	CG	PRO	A	81	64.160	50.850	13.150	1.0	-2.77	PROT	C
ATOM	276	CB	PRO	A	81	63.870	49.800	14.180	1.0	-2.89	PROT	C
ATOM	277	CA	PRO	A	81	62.470	49.260	13.790	1.0	0.30	PROT	C
ATOM	278	C	PRO	A	81	61.640	49.060	15.140	1.0	5.67	PROT	C
ATOM	279	O	PRO	A	81	61.390	47.950	15.560	1.0	-6.29	PROT	O
ATOM	280	HA	PRO	A	81	62.498	48.265	13.285	1.0	1.82	PROT	H
ATOM	281	HB2	PRO	A	81	63.873	50.213	15.207	1.0	1.56	PROT	H
ATOM	282	HB3	PRO	A	81	64.621	48.988	14.190	1.0	1.63	PROT	H
ATOM	283	HG2	PRO	A	81	64.799	51.664	13.544	1.0	1.58	PROT	H
ATOM	284	HG3	PRO	A	81	64.728	50.433	12.293	1.0	1.56	PROT	H
ATOM	285	HD2	PRO	A	81	62.485	52.278	13.285	1.0	1.73	PROT	H
ATOM	286	HD3	PRO	A	81	62.826	51.686	11.619	1.0	1.51	PROT	H
ATOM	287	N	VAL	A	82	61.080	50.130	15.730	1.0	-5.48	PROT	N
ATOM	288	CA	VAL	A	82	60.280	50.070	17.010	1.0	-0.27	PROT	C
ATOM	289	CB	VAL	A	82	61.170	50.140	18.310	1.0	-0.67	PROT	C
ATOM	290	CG1	VAL	A	82	62.200	49.110	18.440	1.0	-4.61	PROT	C
ATOM	291	CG2	VAL	A	82	61.880	51.510	18.450	1.0	-4.47	PROT	C
ATOM	292	C	VAL	A	82	59.190	51.100	17.130	1.0	5.70	PROT	C
ATOM	293	O	VAL	A	82	59.320	52.170	16.550	1.0	-6.19	PROT	O
ATOM	294	H	VAL	A	82	61.187	51.073	15.355	1.0	3.40	PROT	H
ATOM	295	HA	VAL	A	82	59.819	49.032	17.007	1.0	1.85	PROT	H
ATOM	296	HB	VAL	A	82	60.440	50.055	19.165	1.0	1.46	PROT	H
ATOM	297	HG11	VAL	A	82	61.808	48.087	18.307	1.0	1.51	PROT	H
ATOM	298	HG12	VAL	A	82	63.006	49.207	17.694	1.0	1.55	PROT	H

ATOM	299	HG13	VAL	A	82	62.687	49.127	19.428	1.0	1.53	PROT	H
ATOM	300	HG21	VAL	A	82	62.408	51.576	19.407	1.0	1.49	PROT	H
ATOM	301	HG22	VAL	A	82	62.601	51.674	17.643	1.0	1.49	PROT	H
ATOM	302	HG23	VAL	A	82	61.159	52.338	18.424	1.0	1.59	PROT	H
ATOM	303	N	ASN	A	83	58.110	50.830	17.840	1.0	-5.20	PROT	N
ATOM	304	CA	ASN	A	83	57.170	51.880	18.170	1.0	0.57	PROT	C
ATOM	305	CB	ASN	A	83	56.070	51.170	18.850	1.0	-4.07	PROT	C
ATOM	306	CG	ASN	A	83	55.300	50.140	18.010	1.0	6.13	PROT	C
ATOM	307	OD1	ASN	A	83	54.860	50.430	16.920	1.0	-6.35	PROT	O
ATOM	308	ND2	ASN	A	83	55.330	48.880	18.340	1.0	-6.19	PROT	N
ATOM	309	C	ASN	A	83	57.820	52.890	19.110	1.0	5.42	PROT	C
ATOM	310	O	ASN	A	83	58.520	52.630	20.140	1.0	-6.24	PROT	O
ATOM	311	H	ASN	A	83	57.998	49.942	18.333	1.0	3.33	PROT	H
ATOM	312	HA	ASN	A	83	56.827	52.395	17.214	1.0	2.10	PROT	H
ATOM	313	HB2	ASN	A	83	55.296	51.911	19.200	1.0	2.06	PROT	H
ATOM	314	HB3	ASN	A	83	56.413	50.712	19.811	1.0	1.87	PROT	H
ATOM	315	HD21	ASN	A	83	55.635	48.529	19.237	1.0	3.19	PROT	H
ATOM	316	HD22	ASN	A	83	54.876	48.179	17.763	1.0	3.24	PROT	H
ATOM	317	N	ILE	A	84	57.520	54.180	18.880	1.0	-5.41	PROT	N
ATOM	318	CA	ILE	A	84	58.170	55.310	19.670	1.0	-0.38	PROT	C
ATOM	319	CB	ILE	A	84	59.230	56.040	18.730	1.0	-0.77	PROT	C
ATOM	320	CG2	ILE	A	84	59.830	57.260	19.430	1.0	-4.54	PROT	C
ATOM	321	CG1	ILE	A	84	60.370	55.120	18.290	1.0	-2.76	PROT	C
ATOM	322	CD1	ILE	A	84	61.300	55.750	17.220	1.0	-4.26	PROT	C
ATOM	323	C	ILE	A	84	57.070	56.230	20.180	1.0	5.79	PROT	C
ATOM	324	O	ILE	A	84	56.260	56.680	19.360	1.0	-6.36	PROT	O
ATOM	325	H	ILE	A	84	56.924	54.514	18.117	1.0	3.60	PROT	H
ATOM	326	HA	ILE	A	84	58.741	54.842	20.518	1.0	1.79	PROT	H
ATOM	327	HB	ILE	A	84	58.665	56.380	17.827	1.0	1.50	PROT	H
ATOM	328	HG12	ILE	A	84	59.953	54.179	17.870	1.0	1.59	PROT	H
ATOM	329	HG13	ILE	A	84	60.973	54.809	19.163	1.0	1.35	PROT	H
ATOM	330	HG21	ILE	A	84	59.051	57.958	19.764	1.0	1.55	PROT	H
ATOM	331	HG22	ILE	A	84	60.428	56.966	20.298	1.0	1.37	PROT	H
ATOM	332	HG23	ILE	A	84	60.485	57.820	18.750	1.0	1.60	PROT	H
ATOM	333	HD11	ILE	A	84	62.047	55.016	16.893	1.0	1.48	PROT	H
ATOM	334	HD12	ILE	A	84	60.731	56.054	16.337	1.0	1.42	PROT	H
ATOM	335	HD13	ILE	A	84	61.835	56.617	17.612	1.0	1.36	PROT	H
ATOM	336	N	NME	A	85	57.079	56.469	21.473	1.0	-6.19	PROT	N

ATOM	337	H1	NME A	85	57.788	56.088	22.081	1.0	3.46	PROT	H
ATOM	338	H2	NME A	85	56.408	57.094	21.899	1.0	3.26	PROT	H
TER	339		NME A	85							
ATOM	340	C	ACE B	7	60.023	70.585	22.734	1.0	4.37	PROT	C
ATOM	341	O	ACE B	7	60.133	70.030	21.622	1.0	-6.11	PROT	O
ATOM	342	HC	ACE B	7	59.116	71.200	22.928	1.0	1.48	PROT	H
ATOM	343	N	ARG B	8	60.890	70.490	23.680	1.0	-5.19	PROT	N
ATOM	344	CA	ARG B	8	62.130	69.690	23.530	1.0	0.19	PROT	C
ATOM	345	CB	ARG B	8	63.050	70.270	22.360	1.0	-2.73	PROT	C
ATOM	346	CG	ARG B	8	62.350	70.100	21.040	1.0	-3.13	PROT	C
ATOM	347	CD	ARG B	8	63.220	70.320	19.860	1.0	-0.45	PROT	C
ATOM	348	NE	ARG B	8	62.660	69.490	18.750	1.0	-5.72	PROT	N
ATOM	349	CZ	ARG B	8	62.700	69.700	17.430	1.0	6.16	PROT	C
ATOM	350	NH1	ARG B	8	63.590	70.570	16.980	1.0	-6.45	PROT	N
ATOM	351	NH2	ARG B	8	61.880	69.150	16.610	1.0	-6.07	PROT	N
ATOM	352	C	ARG B	8	62.870	69.870	24.930	1.0	5.35	PROT	C
ATOM	353	O	ARG B	8	63.020	71.020	25.430	1.0	-6.54	PROT	O
ATOM	354	H	ARG B	8	60.846	71.057	24.534	1.0	3.41	PROT	H
ATOM	355	HA	ARG B	8	61.876	68.627	23.316	1.0	1.81	PROT	H
ATOM	356	HB2	ARG B	8	64.016	69.730	22.388	1.0	1.48	PROT	H
ATOM	357	HB3	ARG B	8	63.278	71.329	22.578	1.0	1.55	PROT	H
ATOM	358	HG2	ARG B	8	61.553	70.885	20.930	1.0	1.92	PROT	H
ATOM	359	HG3	ARG B	8	61.842	69.096	20.994	1.0	2.16	PROT	H
ATOM	360	HD2	ARG B	8	64.275	69.981	20.022	1.0	1.50	PROT	H
ATOM	361	HD3	ARG B	8	63.273	71.393	19.565	1.0	1.37	PROT	H
ATOM	362	HE	ARG B	8	61.879	68.819	19.088	1.0	4.06	PROT	H
ATOM	363	HH11	ARG B	8	64.183	71.117	17.590	1.0	3.40	PROT	H
ATOM	364	HH12	ARG B	8	63.698	70.788	15.999	1.0	3.35	PROT	H
ATOM	365	HH21	ARG B	8	61.348	68.270	16.917	1.0	4.02	PROT	H
ATOM	366	HH22	ARG B	8	62.010	69.182	15.599	1.0	3.37	PROT	H
ATOM	367	N	NME B	9	63.295	68.763	25.498	1.0	-5.86	PROT	N
ATOM	368	H1	NME B	9	63.766	68.767	26.394	1.0	3.26	PROT	H
ATOM	369	H2	NME B	9	63.137	67.841	25.114	1.0	3.30	PROT	H
ATOM	370	C	ACE B	10	70.571	69.287	23.176	1.0	4.97	PROT	C
ATOM	371	O	ACE B	10	69.652	69.849	22.545	1.0	-6.75	PROT	O
ATOM	372	HC	ACE B	10	71.598	69.704	23.082	1.0	1.51	PROT	H
ATOM	373	N	LEU B	23	70.410	68.250	23.920	1.0	-5.14	PROT	N
ATOM	374	CA	LEU B	23	69.080	67.610	24.100	1.0	-0.13	PROT	C

ATOM	375	CB	LEU	B	23	68.620	67.140	22.650	1.0	-3.20	PROT	C
ATOM	376	CG	LEU	B	23	67.240	66.600	22.500	1.0	-0.72	PROT	C
ATOM	377	CD1	LEU	B	23	66.090	67.600	22.700	1.0	-4.51	PROT	C
ATOM	378	CD2	LEU	B	23	66.950	66.070	21.080	1.0	-4.48	PROT	C
ATOM	379	C	LEU	B	23	69.320	66.420	25.050	1.0	5.75	PROT	C
ATOM	380	O	LEU	B	23	70.160	65.550	24.770	1.0	-6.43	PROT	O
ATOM	381	H	LEU	B	23	71.207	67.676	24.220	1.0	3.39	PROT	H
ATOM	382	HA	LEU	B	23	68.353	68.358	24.492	1.0	1.72	PROT	H
ATOM	383	HB2	LEU	B	23	69.372	66.404	22.292	1.0	1.66	PROT	H
ATOM	384	HB3	LEU	B	23	68.749	68.019	21.971	1.0	2.01	PROT	H
ATOM	385	HG	LEU	B	23	67.100	65.761	23.231	1.0	1.20	PROT	H
ATOM	386	HD11	LEU	B	23	65.114	67.110	22.609	1.0	1.56	PROT	H
ATOM	387	HD12	LEU	B	23	66.134	68.075	23.686	1.0	1.27	PROT	H
ATOM	388	HD13	LEU	B	23	66.134	68.408	21.959	1.0	1.53	PROT	H
ATOM	389	HD21	LEU	B	23	67.627	65.250	20.808	1.0	1.46	PROT	H
ATOM	390	HD22	LEU	B	23	65.921	65.698	20.989	1.0	1.61	PROT	H
ATOM	391	HD23	LEU	B	23	67.081	66.857	20.329	1.0	1.47	PROT	H
ATOM	392	N	LEU	B	24	68.420	66.310	26.020	1.0	-5.50	PROT	N
ATOM	393	CA	LEU	B	24	68.360	65.220	27.060	1.0	0.30	PROT	C
ATOM	394	CB	LEU	B	24	67.960	65.810	28.370	1.0	-3.28	PROT	C
ATOM	395	CG	LEU	B	24	69.130	66.410	29.200	1.0	-0.55	PROT	C
ATOM	396	CD1	LEU	B	24	68.480	66.920	30.470	1.0	-4.68	PROT	C
ATOM	397	CD2	LEU	B	24	70.270	65.530	29.540	1.0	-4.65	PROT	C
ATOM	398	C	LEU	B	24	67.450	64.100	26.450	1.0	5.74	PROT	C
ATOM	399	O	LEU	B	24	66.210	64.180	26.570	1.0	-6.36	PROT	O
ATOM	400	H	LEU	B	24	67.748	67.061	26.201	1.0	3.35	PROT	H
ATOM	401	HA	LEU	B	24	69.405	64.785	27.150	1.0	1.87	PROT	H
ATOM	402	HB2	LEU	B	24	67.457	65.025	28.986	1.0	1.78	PROT	H
ATOM	403	HB3	LEU	B	24	67.171	66.584	28.247	1.0	1.67	PROT	H
ATOM	404	HG	LEU	B	24	69.520	67.289	28.613	1.0	1.36	PROT	H
ATOM	405	HD11	LEU	B	24	67.667	67.630	30.270	1.0	1.46	PROT	H
ATOM	406	HD12	LEU	B	24	68.058	66.105	31.073	1.0	1.52	PROT	H
ATOM	407	HD13	LEU	B	24	69.199	67.446	31.113	1.0	1.49	PROT	H
ATOM	408	HD21	LEU	B	24	70.835	65.194	28.656	1.0	1.51	PROT	H
ATOM	409	HD22	LEU	B	24	70.999	66.037	30.191	1.0	1.55	PROT	H
ATOM	410	HD23	LEU	B	24	69.959	64.619	30.074	1.0	1.57	PROT	H
ATOM	411	N	ASH	B	25	68.020	63.210	25.620	1.0	-5.47	PROT	N
ATOM	412	CA	ASH	B	25	67.300	62.340	24.660	1.0	0.39	PROT	C

ATOM	413	CB	ASH	B	25	67.860	62.590	23.300	1.0	-3.79	PROT	C
ATOM	414	CG	ASH	B	25	67.170	61.850	22.190	1.0	6.73	PROT	C
ATOM	415	OD1	ASH	B	25	66.340	60.950	22.370	1.0	-5.79	PROT	O
ATOM	416	OD2	ASH	B	25	67.390	62.380	20.970	1.0	-5.29	PROT	O
ATOM	417	C	ASH	B	25	67.370	60.870	25.220	1.0	5.56	PROT	C
ATOM	418	O	ASH	B	25	68.390	60.230	24.970	1.0	-5.77	PROT	O
ATOM	419	H	ASH	B	25	69.042	63.183	25.527	1.0	3.47	PROT	H
ATOM	420	HA	ASH	B	25	66.202	62.641	24.667	1.0	1.85	PROT	H
ATOM	421	HB2	ASH	B	25	68.950	62.299	23.263	1.0	2.18	PROT	H
ATOM	422	HB3	ASH	B	25	67.862	63.688	23.068	1.0	2.07	PROT	H
ATOM	423	HD2	ASH	B	25	67.129	61.765	20.215	1.0	3.97	PROT	H
ATOM	424	N	THR	B	26	66.270	60.240	25.630	1.0	-5.63	PROT	N
ATOM	425	CA	THR	B	26	66.140	58.790	25.940	1.0	-0.27	PROT	C
ATOM	426	CB	THR	B	26	64.860	58.510	26.730	1.0	1.67	PROT	C
ATOM	427	CG2	THR	B	26	65.080	58.980	28.200	1.0	-4.86	PROT	C
ATOM	428	OG1	THR	B	26	63.630	59.110	26.360	1.0	-6.36	PROT	O
ATOM	429	C	THR	B	26	66.160	57.950	24.710	1.0	5.20	PROT	C
ATOM	430	O	THR	B	26	66.660	56.770	24.680	1.0	-5.72	PROT	O
ATOM	431	H	THR	B	26	65.408	60.760	25.852	1.0	3.50	PROT	H
ATOM	432	HA	THR	B	26	67.033	58.478	26.577	1.0	2.23	PROT	H
ATOM	433	HB	THR	B	26	64.696	57.398	26.720	1.0	1.45	PROT	H
ATOM	434	HG21	THR	B	26	65.955	58.510	28.655	1.0	1.63	PROT	H
ATOM	435	HG22	THR	B	26	65.197	60.069	28.243	1.0	1.64	PROT	H
ATOM	436	HG23	THR	B	26	64.203	58.734	28.809	1.0	1.56	PROT	H
ATOM	437	HG1	THR	B	26	62.918	58.428	26.251	1.0	3.74	PROT	H
ATOM	438	N	GLY	B	27	65.730	58.490	23.580	1.0	-5.22	PROT	N
ATOM	439	CA	GLY	B	27	65.750	57.970	22.210	1.0	-1.34	PROT	C
ATOM	440	C	GLY	B	27	67.120	58.060	21.520	1.0	6.02	PROT	C
ATOM	441	O	GLY	B	27	67.140	58.050	20.270	1.0	-7.09	PROT	O
ATOM	442	H	GLY	B	27	65.467	59.486	23.580	1.0	3.29	PROT	H
ATOM	443	HA2	GLY	B	27	65.000	58.529	21.605	1.0	1.68	PROT	H
ATOM	444	HA3	GLY	B	27	65.472	56.879	22.209	1.0	2.00	PROT	H
ATOM	445	N	ALA	B	28	68.170	58.280	22.260	1.0	-4.72	PROT	N
ATOM	446	CA	ALA	B	28	69.550	58.120	21.860	1.0	0.06	PROT	C
ATOM	447	CB	ALA	B	28	70.190	59.490	21.860	1.0	-4.69	PROT	C
ATOM	448	C	ALA	B	28	70.300	57.100	22.690	1.0	5.62	PROT	C
ATOM	449	O	ALA	B	28	70.360	57.280	23.880	1.0	-6.13	PROT	O
ATOM	450	H	ALA	B	28	68.077	58.400	23.286	1.0	3.46	PROT	H

ATOM	451	HA	ALA	B	28	69.541	57.758	20.772	1.0	1.76	PROT	H
ATOM	452	HB1	ALA	B	28	71.243	59.459	21.537	1.0	1.77	PROT	H
ATOM	453	HB2	ALA	B	28	69.663	60.188	21.197	1.0	1.54	PROT	H
ATOM	454	HB3	ALA	B	28	70.191	59.934	22.871	1.0	1.73	PROT	H
ATOM	455	N	ASP	B	29	70.910	56.140	22.120	1.0	-5.08	PROT	N
ATOM	456	CA	ASP	B	29	71.800	55.270	22.870	1.0	0.14	PROT	C
ATOM	457	CB	ASP	B	29	71.840	53.870	22.370	1.0	-4.21	PROT	C
ATOM	458	CG	ASP	B	29	70.460	53.190	22.170	1.0	7.64	PROT	C
ATOM	459	OD1	ASP	B	29	69.830	52.660	23.090	1.0	-7.76	PROT	O
ATOM	460	OD2	ASP	B	29	70.050	53.270	20.980	1.0	-8.38	PROT	O
ATOM	461	C	ASP	B	29	73.220	55.900	23.100	1.0	5.73	PROT	C
ATOM	462	O	ASP	B	29	73.870	55.530	24.110	1.0	-6.27	PROT	O
ATOM	463	H	ASP	B	29	70.742	55.848	21.145	1.0	3.47	PROT	H
ATOM	464	HA	ASP	B	29	71.389	55.220	23.949	1.0	2.12	PROT	H
ATOM	465	HB2	ASP	B	29	72.437	53.235	23.065	1.0	1.94	PROT	H
ATOM	466	HB3	ASP	B	29	72.378	53.801	21.393	1.0	1.95	PROT	H
ATOM	467	N	ASP	B	30	73.660	56.820	22.240	1.0	-5.05	PROT	N
ATOM	468	CA	ASP	B	30	74.950	57.390	22.250	1.0	0.09	PROT	C
ATOM	469	CB	ASP	B	30	75.820	56.790	21.070	1.0	-4.19	PROT	C
ATOM	470	CG	ASP	B	30	75.670	55.250	20.910	1.0	7.57	PROT	C
ATOM	471	OD1	ASP	B	30	75.010	54.770	19.940	1.0	-8.02	PROT	O
ATOM	472	OD2	ASP	B	30	76.460	54.540	21.590	1.0	-8.22	PROT	O
ATOM	473	C	ASP	B	30	74.920	58.910	22.080	1.0	5.70	PROT	C
ATOM	474	O	ASP	B	30	74.210	59.540	21.260	1.0	-6.03	PROT	O
ATOM	475	H	ASP	B	30	73.116	57.027	21.386	1.0	3.39	PROT	H
ATOM	476	HA	ASP	B	30	75.477	57.108	23.219	1.0	2.02	PROT	H
ATOM	477	HB2	ASP	B	30	76.884	57.011	21.241	1.0	1.69	PROT	H
ATOM	478	HB3	ASP	B	30	75.524	57.255	20.112	1.0	1.72	PROT	H
ATOM	479	N	THR	B	31	75.790	59.540	22.930	1.0	-5.54	PROT	N
ATOM	480	CA	THR	B	31	76.030	60.990	22.880	1.0	-0.91	PROT	C
ATOM	481	CB	THR	B	31	76.680	61.420	24.200	1.0	1.81	PROT	C
ATOM	482	CG2	THR	B	31	76.990	62.990	24.240	1.0	-4.86	PROT	C
ATOM	483	OG1	THR	B	31	75.880	61.150	25.330	1.0	-6.10	PROT	O
ATOM	484	C	THR	B	31	76.700	61.460	21.650	1.0	5.74	PROT	C
ATOM	485	O	THR	B	31	77.890	61.250	21.390	1.0	-6.58	PROT	O
ATOM	486	H	THR	B	31	76.496	59.004	23.441	1.0	3.45	PROT	H
ATOM	487	HA	THR	B	31	74.974	61.463	22.910	1.0	2.10	PROT	H
ATOM	488	HB	THR	B	31	77.635	60.870	24.384	1.0	1.44	PROT	H

ATOM	489	HG21	THR	B	31	77.820	63.222	23.572	1.0	1.59	PROT	H
ATOM	490	HG22	THR	B	31	76.109	63.556	23.924	1.0	1.68	PROT	H
ATOM	491	HG23	THR	B	31	77.245	63.282	25.258	1.0	1.52	PROT	H
ATOM	492	HG1	THR	B	31	75.160	60.514	25.123	1.0	3.54	PROT	H
ATOM	493	N	VAL	B	32	75.990	62.150	20.830	1.0	-4.91	PROT	N
ATOM	494	CA	VAL	B	32	76.490	62.780	19.570	1.0	-0.42	PROT	C
ATOM	495	CB	VAL	B	32	75.500	62.450	18.490	1.0	-0.83	PROT	C
ATOM	496	CG1	VAL	B	32	75.740	63.270	17.210	1.0	-4.57	PROT	C
ATOM	497	CG2	VAL	B	32	75.320	60.920	18.130	1.0	-4.38	PROT	C
ATOM	498	C	VAL	B	32	76.670	64.230	19.790	1.0	6.01	PROT	C
ATOM	499	O	VAL	B	32	75.690	64.880	20.120	1.0	-6.56	PROT	O
ATOM	500	H	VAL	B	32	75.033	62.473	21.063	1.0	3.50	PROT	H
ATOM	501	HA	VAL	B	32	77.484	62.273	19.321	1.0	2.00	PROT	H
ATOM	502	HB	VAL	B	32	74.481	62.769	18.868	1.0	1.48	PROT	H
ATOM	503	HG11	VAL	B	32	75.643	64.346	17.399	1.0	1.50	PROT	H
ATOM	504	HG12	VAL	B	32	76.738	63.091	16.795	1.0	1.47	PROT	H
ATOM	505	HG13	VAL	B	32	75.012	63.012	16.433	1.0	1.49	PROT	H
ATOM	506	HG21	VAL	B	32	75.134	60.337	19.042	1.0	1.73	PROT	H
ATOM	507	HG22	VAL	B	32	74.470	60.792	17.461	1.0	1.37	PROT	H
ATOM	508	HG23	VAL	B	32	76.214	60.531	17.640	1.0	1.48	PROT	H
ATOM	509	N	NME	B	33	77.889	64.689	19.606	1.0	-6.21	PROT	N
ATOM	510	H1	NME	B	33	78.113	65.664	19.748	1.0	3.14	PROT	H
ATOM	511	H2	NME	B	33	78.674	64.091	19.405	1.0	3.49	PROT	H
ATOM	512	C	ACE	B	34	77.086	55.483	12.475	1.0	4.49	PROT	C
ATOM	513	O	ACE	B	34	77.417	55.313	13.667	1.0	-6.37	PROT	O
ATOM	514	HC	ACE	B	34	77.890	55.614	11.717	1.0	1.38	PROT	H
ATOM	515	N	ILE	B	47	75.870	55.530	12.060	1.0	-5.06	PROT	N
ATOM	516	CA	ILE	B	47	74.700	55.380	13.000	1.0	-0.47	PROT	C
ATOM	517	CB	ILE	B	47	74.800	56.460	14.140	1.0	-0.85	PROT	C
ATOM	518	CG2	ILE	B	47	76.000	56.200	15.010	1.0	-4.21	PROT	C
ATOM	519	CG1	ILE	B	47	74.740	57.980	13.710	1.0	-2.67	PROT	C
ATOM	520	CD1	ILE	B	47	75.150	58.970	14.810	1.0	-4.33	PROT	C
ATOM	521	C	ILE	B	47	73.350	55.470	12.280	1.0	5.50	PROT	C
ATOM	522	O	ILE	B	47	73.270	56.330	11.420	1.0	-6.24	PROT	O
ATOM	523	H	ILE	B	47	75.600	55.787	11.110	1.0	3.32	PROT	H
ATOM	524	HA	ILE	B	47	74.829	54.372	13.489	1.0	1.79	PROT	H
ATOM	525	HB	ILE	B	47	73.876	56.297	14.763	1.0	1.38	PROT	H
ATOM	526	HG12	ILE	B	47	73.719	58.197	13.355	1.0	1.28	PROT	H

ATOM	527	HG13	ILE	B	47	75.393	58.116	12.830	1.0	1.34	PROT	H
ATOM	528	HG21	ILE	B	47	75.783	56.554	16.042	1.0	1.43	PROT	H
ATOM	529	HG22	ILE	B	47	76.219	55.133	15.159	1.0	1.69	PROT	H
ATOM	530	HG23	ILE	B	47	76.889	56.783	14.737	1.0	1.65	PROT	H
ATOM	531	HD11	ILE	B	47	76.228	58.926	15.005	1.0	1.50	PROT	H
ATOM	532	HD12	ILE	B	47	74.916	59.997	14.512	1.0	1.35	PROT	H
ATOM	533	HD13	ILE	B	47	74.633	58.759	15.754	1.0	1.57	PROT	H
ATOM	534	N	GLY	B	48	72.470	54.600	12.670	1.0	-4.83	PROT	N
ATOM	535	CA	GLY	B	48	71.140	54.390	12.160	1.0	-1.98	PROT	C
ATOM	536	C	GLY	B	48	70.110	55.070	12.940	1.0	5.91	PROT	C
ATOM	537	O	GLY	B	48	70.280	55.230	14.150	1.0	-6.21	PROT	O
ATOM	538	H	GLY	B	48	72.646	54.040	13.527	1.0	3.50	PROT	H
ATOM	539	HA2	GLY	B	48	70.934	53.277	12.148	1.0	1.96	PROT	H
ATOM	540	HA3	GLY	B	48	71.122	54.671	11.065	1.0	1.94	PROT	H
ATOM	541	N	GLY	B	49	69.100	55.510	12.260	1.0	-5.13	PROT	N
ATOM	542	CA	GLY	B	49	67.870	56.210	12.670	1.0	-1.56	PROT	C
ATOM	543	C	GLY	B	49	66.640	56.050	11.730	1.0	5.84	PROT	C
ATOM	544	O	GLY	B	49	66.630	55.240	10.820	1.0	-6.20	PROT	O
ATOM	545	H	GLY	B	49	69.019	55.266	11.253	1.0	3.32	PROT	H
ATOM	546	HA2	GLY	B	49	68.106	57.301	12.765	1.0	1.76	PROT	H
ATOM	547	HA3	GLY	B	49	67.569	55.859	13.695	1.0	1.98	PROT	H
ATOM	548	N	ILE	B	50	65.540	56.820	12.000	1.0	-5.66	PROT	N
ATOM	549	CA	ILE	B	50	64.360	56.910	11.120	1.0	0.09	PROT	C
ATOM	550	CB	ILE	B	50	63.130	57.320	11.900	1.0	-1.22	PROT	C
ATOM	551	CG2	ILE	B	50	61.970	57.360	10.960	1.0	-4.69	PROT	C
ATOM	552	CG1	ILE	B	50	62.790	56.450	13.230	1.0	-2.23	PROT	C
ATOM	553	CD1	ILE	B	50	62.620	55.020	13.000	1.0	-4.50	PROT	C
ATOM	554	C	ILE	B	50	64.680	57.930	10.000	1.0	5.68	PROT	C
ATOM	555	O	ILE	B	50	65.120	59.080	10.230	1.0	-6.61	PROT	O
ATOM	556	H	ILE	B	50	65.596	57.595	12.675	1.0	3.55	PROT	H
ATOM	557	HA	ILE	B	50	64.198	55.885	10.670	1.0	1.77	PROT	H
ATOM	558	HB	ILE	B	50	63.311	58.356	12.310	1.0	1.56	PROT	H
ATOM	559	HG12	ILE	B	50	61.880	56.911	13.663	1.0	1.34	PROT	H
ATOM	560	HG13	ILE	B	50	63.607	56.655	13.951	1.0	1.38	PROT	H
ATOM	561	HG21	ILE	B	50	61.771	56.384	10.492	1.0	1.59	PROT	H
ATOM	562	HG22	ILE	B	50	61.037	57.637	11.475	1.0	1.58	PROT	H
ATOM	563	HG23	ILE	B	50	62.097	58.091	10.149	1.0	1.51	PROT	H
ATOM	564	HD11	ILE	B	50	63.537	54.530	12.641	1.0	1.40	PROT	H

ATOM	565	HD12	ILE	B	50	62.312	54.484	13.917	1.0	1.61	PROT	H
ATOM	566	HD13	ILE	B	50	61.834	54.796	12.259	1.0	1.52	PROT	H
ATOM	567	N	NME	B	51	64.461	57.516	8.771	1.0	-5.98	PROT	N
ATOM	568	H1	NME	B	51	64.161	56.585	8.528	1.0	3.28	PROT	H
ATOM	569	H2	NME	B	51	64.696	58.100	7.976	1.0	3.26	PROT	H
ATOM	570	C	ACE	B	52	80.394	59.569	20.555	1.0	4.57	PROT	C
ATOM	571	O	ACE	B	52	80.381	58.434	20.035	1.0	-6.44	PROT	O
ATOM	572	HC	ACE	B	52	80.378	59.640	21.666	1.0	1.49	PROT	H
ATOM	573	N	LEU	B	76	80.390	60.670	19.890	1.0	-4.91	PROT	N
ATOM	574	CA	LEU	B	76	80.370	60.690	18.460	1.0	-0.35	PROT	C
ATOM	575	CB	LEU	B	76	79.040	59.950	18.110	1.0	-2.95	PROT	C
ATOM	576	CG	LEU	B	76	78.900	58.470	18.330	1.0	-0.26	PROT	C
ATOM	577	CD1	LEU	B	76	77.520	58.060	17.820	1.0	-4.82	PROT	C
ATOM	578	CD2	LEU	B	76	79.920	57.600	17.620	1.0	-4.37	PROT	C
ATOM	579	C	LEU	B	76	80.370	62.150	17.950	1.0	5.90	PROT	C
ATOM	580	O	LEU	B	76	79.690	63.010	18.500	1.0	-6.76	PROT	O
ATOM	581	H	LEU	B	76	80.118	61.558	20.344	1.0	3.43	PROT	H
ATOM	582	HA	LEU	B	76	81.242	60.117	18.044	1.0	1.73	PROT	H
ATOM	583	HB2	LEU	B	76	78.823	60.171	17.039	1.0	1.35	PROT	H
ATOM	584	HB3	LEU	B	76	78.227	60.470	18.678	1.0	1.65	PROT	H
ATOM	585	HG	LEU	B	76	78.804	58.193	19.419	1.0	1.88	PROT	H
ATOM	586	HD11	LEU	B	76	77.361	56.979	17.944	1.0	1.62	PROT	H
ATOM	587	HD12	LEU	B	76	76.715	58.560	18.371	1.0	1.44	PROT	H
ATOM	588	HD13	LEU	B	76	77.381	58.279	16.757	1.0	1.31	PROT	H
ATOM	589	HD21	LEU	B	76	79.838	57.679	16.531	1.0	1.22	PROT	H
ATOM	590	HD22	LEU	B	76	80.947	57.872	17.891	1.0	1.36	PROT	H
ATOM	591	HD23	LEU	B	76	79.794	56.545	17.891	1.0	1.49	PROT	H
ATOM	592	N	NME	B	77	81.136	62.391	16.908	1.0	-6.04	PROT	N
ATOM	593	H1	NME	B	77	81.739	61.703	16.481	1.0	3.32	PROT	H
ATOM	594	H2	NME	B	77	81.202	63.313	16.497	1.0	3.23	PROT	H
ATOM	595	C	ACE	B	78	75.140	66.033	12.484	1.0	4.65	PROT	C
ATOM	596	O	ACE	B	78	74.709	64.914	12.831	1.0	-6.78	PROT	O
ATOM	597	HC	ACE	B	78	76.077	66.068	11.884	1.0	1.53	PROT	H
ATOM	598	N	THR	B	80	74.570	67.150	12.770	1.0	-4.83	PROT	N
ATOM	599	CA	THR	B	80	73.330	67.210	13.550	1.0	-0.74	PROT	C
ATOM	600	CB	THR	B	80	73.580	66.470	14.930	1.0	1.94	PROT	C
ATOM	601	CG2	THR	B	80	74.790	66.960	15.680	1.0	-5.09	PROT	C
ATOM	602	OG1	THR	B	80	72.370	66.530	15.640	1.0	-6.31	PROT	O

ATOM	603	C	THR	B	80	72.830	68.650	13.770	1.0	5.68	PROT	C
ATOM	604	O	THR	B	80	73.670	69.590	13.790	1.0	-6.37	PROT	O
ATOM	605	H	THR	B	80	74.976	68.071	12.554	1.0	3.50	PROT	H
ATOM	606	HA	THR	B	80	72.547	66.616	12.997	1.0	1.85	PROT	H
ATOM	607	HB	THR	B	80	73.671	65.370	14.701	1.0	1.71	PROT	H
ATOM	608	HG21	THR	B	80	74.859	68.057	15.699	1.0	1.68	PROT	H
ATOM	609	HG22	THR	B	80	74.764	66.626	16.730	1.0	1.76	PROT	H
ATOM	610	HG23	THR	B	80	75.724	66.582	15.245	1.0	1.68	PROT	H
ATOM	611	HG1	THR	B	80	72.464	66.941	16.532	1.0	3.75	PROT	H
ATOM	612	N	PRO	B	81	71.500	68.910	13.940	1.0	-4.68	PROT	N
ATOM	613	CD	PRO	B	81	70.510	67.910	13.680	1.0	-0.96	PROT	C
ATOM	614	CG	PRO	B	81	69.230	68.510	14.280	1.0	-2.74	PROT	C
ATOM	615	CB	PRO	B	81	69.410	69.970	13.960	1.0	-2.92	PROT	C
ATOM	616	CA	PRO	B	81	70.890	70.230	14.230	1.0	0.36	PROT	C
ATOM	617	C	PRO	B	81	71.240	70.750	15.650	1.0	5.51	PROT	C
ATOM	618	O	PRO	B	81	71.420	72.010	15.870	1.0	-6.56	PROT	O
ATOM	619	HA	PRO	B	81	71.317	70.999	13.522	1.0	1.91	PROT	H
ATOM	620	HB2	PRO	B	81	68.757	70.630	14.563	1.0	1.65	PROT	H
ATOM	621	HB3	PRO	B	81	69.157	70.201	12.905	1.0	1.71	PROT	H
ATOM	622	HG2	PRO	B	81	69.152	68.317	15.367	1.0	1.52	PROT	H
ATOM	623	HG3	PRO	B	81	68.319	68.076	13.832	1.0	1.53	PROT	H
ATOM	624	HD2	PRO	B	81	70.756	66.930	14.165	1.0	1.83	PROT	H
ATOM	625	HD3	PRO	B	81	70.393	67.716	12.587	1.0	1.55	PROT	H
ATOM	626	N	VAL	B	82	71.290	69.860	16.650	1.0	-5.07	PROT	N
ATOM	627	CA	VAL	B	82	71.700	70.050	18.060	1.0	-0.34	PROT	C
ATOM	628	CB	VAL	B	82	70.440	70.170	19.000	1.0	-0.73	PROT	C
ATOM	629	CG1	VAL	B	82	69.400	71.300	18.760	1.0	-4.41	PROT	C
ATOM	630	CG2	VAL	B	82	69.520	68.910	19.110	1.0	-4.48	PROT	C
ATOM	631	C	VAL	B	82	72.590	68.960	18.550	1.0	5.74	PROT	C
ATOM	632	O	VAL	B	82	72.520	67.810	18.130	1.0	-6.12	PROT	O
ATOM	633	H	VAL	B	82	71.178	68.862	16.420	1.0	3.29	PROT	H
ATOM	634	HA	VAL	B	82	72.218	71.060	18.104	1.0	1.92	PROT	H
ATOM	635	HB	VAL	B	82	70.866	70.340	20.026	1.0	1.50	PROT	H
ATOM	636	HG11	VAL	B	82	69.888	72.274	18.651	1.0	1.43	PROT	H
ATOM	637	HG12	VAL	B	82	68.824	71.125	17.847	1.0	1.49	PROT	H
ATOM	638	HG13	VAL	B	82	68.709	71.362	19.606	1.0	1.51	PROT	H
ATOM	639	HG21	VAL	B	82	68.965	68.737	18.188	1.0	1.39	PROT	H
ATOM	640	HG22	VAL	B	82	70.117	68.013	19.328	1.0	1.62	PROT	H

ATOM	641	HG23	VAL	B	82	68.818	69.033	19.941	1.0	1.55	PROT	H
ATOM	642	N	ASN	B	83	73.310	69.280	19.580	1.0	-5.13	PROT	N
ATOM	643	CA	ASN	B	83	74.000	68.230	20.360	1.0	0.24	PROT	C
ATOM	644	CB	ASN	B	83	74.990	68.980	21.330	1.0	-3.66	PROT	C
ATOM	645	CG	ASN	B	83	75.840	69.910	20.400	1.0	6.17	PROT	C
ATOM	646	OD1	ASN	B	83	76.790	69.630	19.730	1.0	-6.16	PROT	O
ATOM	647	ND2	ASN	B	83	75.440	71.160	20.280	1.0	-6.48	PROT	N
ATOM	648	C	ASN	B	83	73.020	67.350	21.090	1.0	5.64	PROT	C
ATOM	649	O	ASN	B	83	71.920	67.730	21.470	1.0	-6.10	PROT	O
ATOM	650	H	ASN	B	83	73.317	70.212	19.989	1.0	3.32	PROT	H
ATOM	651	HA	ASN	B	83	74.611	67.600	19.639	1.0	2.07	PROT	H
ATOM	652	HB2	ASN	B	83	75.655	68.256	21.836	1.0	1.96	PROT	H
ATOM	653	HB3	ASN	B	83	74.463	69.537	22.116	1.0	1.77	PROT	H
ATOM	654	HD21	ASN	B	83	74.746	71.605	20.860	1.0	3.15	PROT	H
ATOM	655	HD22	ASN	B	83	75.942	71.806	19.678	1.0	3.27	PROT	H
ATOM	656	N	ILE	B	84	73.300	66.040	21.240	1.0	-5.72	PROT	N
ATOM	657	CA	ILE	B	84	72.330	65.010	21.760	1.0	-0.02	PROT	C
ATOM	658	CB	ILE	B	84	71.790	64.060	20.660	1.0	-0.92	PROT	C
ATOM	659	CG2	ILE	B	84	71.070	62.920	21.380	1.0	-4.57	PROT	C
ATOM	660	CG1	ILE	B	84	70.950	64.880	19.610	1.0	-2.63	PROT	C
ATOM	661	CD1	ILE	B	84	70.870	64.150	18.200	1.0	-4.26	PROT	C
ATOM	662	C	ILE	B	84	73.140	64.220	22.820	1.0	5.69	PROT	C
ATOM	663	O	ILE	B	84	74.140	63.530	22.500	1.0	-6.35	PROT	O
ATOM	664	H	ILE	B	84	74.140	65.597	20.844	1.0	3.64	PROT	H
ATOM	665	HA	ILE	B	84	71.465	65.553	22.227	1.0	1.69	PROT	H
ATOM	666	HB	ILE	B	84	72.664	63.634	20.103	1.0	1.59	PROT	H
ATOM	667	HG12	ILE	B	84	71.411	65.870	19.456	1.0	1.56	PROT	H
ATOM	668	HG13	ILE	B	84	69.934	65.045	19.990	1.0	1.29	PROT	H
ATOM	669	HG21	ILE	B	84	70.366	63.292	22.129	1.0	1.31	PROT	H
ATOM	670	HG22	ILE	B	84	70.492	62.320	20.662	1.0	1.64	PROT	H
ATOM	671	HG23	ILE	B	84	71.783	62.242	21.869	1.0	1.67	PROT	H
ATOM	672	HD11	ILE	B	84	71.864	64.086	17.754	1.0	1.47	PROT	H
ATOM	673	HD12	ILE	B	84	70.455	63.149	18.314	1.0	1.40	PROT	H
ATOM	674	HD13	ILE	B	84	70.232	64.725	17.527	1.0	1.39	PROT	H
ATOM	675	N	NME	B	85	72.712	64.322	24.059	1.0	-6.14	PROT	N
ATOM	676	H1	NME	B	85	71.864	64.811	24.304	1.0	3.35	PROT	H
ATOM	677	H2	NME	B	85	73.166	63.822	24.813	1.0	3.24	PROT	H
HETATM	678	C	ROC	C	100	68.360	55.190	17.600	1.0	6.17	PROT	C

HETATM	679	O	ROC	C	100	68.000	55.620	18.670	1.0	-5.54	PROT	O
HETATM	680	N1	ROC	C	100	67.980	53.570	15.780	1.0	-3.84	PROT	N
HETATM	681	C2	ROC	C	100	68.100	53.790	17.110	1.0	0.90	PROT	C
HETATM	682	C3	ROC	C	100	67.870	52.730	18.030	1.0	-1.57	PROT	C
HETATM	683	C4	ROC	C	100	67.720	51.400	17.480	1.0	-0.99	PROT	C
HETATM	684	C5	ROC	C	100	67.580	49.990	15.510	1.0	-1.48	PROT	C
HETATM	685	C6	ROC	C	100	67.620	49.820	14.150	1.0	-1.60	PROT	C
HETATM	686	C7	ROC	C	100	67.790	50.920	13.330	1.0	-1.34	PROT	C
HETATM	687	C8	ROC	C	100	67.840	52.200	13.900	1.0	-1.62	PROT	C
HETATM	688	C4A	ROC	C	100	67.650	51.240	16.090	1.0	-0.80	PROT	C
HETATM	689	C8A	ROC	C	100	67.800	52.350	15.280	1.0	1.58	PROT	C
HETATM	690	N	ROC	C	100	68.910	56.040	16.680	1.0	-5.67	PROT	N
HETATM	691	CA	ROC	C	100	69.200	57.450	17.000	1.0	0.40	PROT	C
HETATM	692	C1	ROC	C	100	68.010	58.360	16.650	1.0	6.02	PROT	C
HETATM	693	O1	ROC	C	100	68.030	59.220	15.760	1.0	-6.63	PROT	O
HETATM	694	CB	ROC	C	100	70.510	57.960	16.400	1.0	-3.82	PROT	C
HETATM	695	CG	ROC	C	100	71.030	59.250	17.050	1.0	6.76	PROT	C
HETATM	696	ND2	ROC	C	100	71.770	60.090	16.250	1.0	-6.61	PROT	N
HETATM	697	OD1	ROC	C	100	70.810	59.490	18.200	1.0	-6.33	PROT	O
HETATM	698	C9	ROC	C	100	65.600	60.280	17.850	1.0	0.77	PROT	C
HETATM	699	O2	ROC	C	100	65.490	60.240	19.310	1.0	-6.05	PROT	O
HETATM	700	CA1	ROC	C	100	65.550	58.770	17.300	1.0	0.55	PROT	C
HETATM	701	N2	ROC	C	100	66.880	58.150	17.480	1.0	-6.08	PROT	N
HETATM	702	CB1	ROC	C	100	64.530	57.790	17.890	1.0	-3.11	PROT	C
HETATM	703	CG1	ROC	C	100	64.700	56.340	17.440	1.0	0.14	PROT	C
HETATM	704	CD1	ROC	C	100	64.990	56.030	16.150	1.0	-1.85	PROT	C
HETATM	705	CD2	ROC	C	100	64.570	55.270	18.380	1.0	-1.47	PROT	C
HETATM	706	CE1	ROC	C	100	65.020	54.700	15.640	1.0	-1.21	PROT	C
HETATM	707	CE2	ROC	C	100	64.630	53.960	17.940	1.0	-1.38	PROT	C
HETATM	708	CZ	ROC	C	100	64.890	53.640	16.560	1.0	-1.64	PROT	C
HETATM	709	N11	ROC	C	100	64.790	62.470	16.740	1.0	-1.20	PROT	N
HETATM	710	CM	ROC	C	100	64.470	61.050	17.250	1.0	-2.19	PROT	C
HETATM	711	C21	ROC	C	100	63.670	63.100	15.870	1.0	-0.94	PROT	C
HETATM	712	CC	ROC	C	100	63.220	62.110	14.750	1.0	5.74	PROT	C
HETATM	713	O3	ROC	C	100	63.880	61.110	14.430	1.0	-6.67	PROT	O
HETATM	714	C31	ROC	C	100	64.060	64.510	15.310	1.0	-2.84	PROT	C
HETATM	715	C3A	ROC	C	100	64.400	65.400	16.520	1.0	-1.20	PROT	C
HETATM	716	C41	ROC	C	100	64.760	66.770	16.080	1.0	-2.83	PROT	C

HETATM	717	C51	ROC	C	100	66.310	66.990	15.790	1.0	-2.65	PROT	C
HETATM	718	C61	ROC	C	100	67.310	66.430	16.890	1.0	-2.57	PROT	C
HETATM	719	C71	ROC	C	100	66.860	65.010	17.280	1.0	-2.84	PROT	C
HETATM	720	C7A	ROC	C	100	65.360	64.810	17.580	1.0	-1.37	PROT	C
HETATM	721	C81	ROC	C	100	64.920	63.390	17.910	1.0	-1.97	PROT	C
HETATM	722	N3	ROC	C	100	62.010	62.400	14.140	1.0	-5.90	PROT	N
HETATM	723	CD	ROC	C	100	61.250	61.590	13.100	1.0	3.48	PROT	C
HETATM	724	C11	ROC	C	100	60.010	62.290	12.780	1.0	-5.31	PROT	C
HETATM	725	C22	ROC	C	100	60.930	60.190	13.790	1.0	-4.88	PROT	C
HETATM	726	C32	ROC	C	100	62.080	61.400	11.740	1.0	-4.80	PROT	C
HETATM	727	H3	ROC	C	100	67.880	52.901	19.103	1.0	2.02	PROT	H
HETATM	728	H4	ROC	C	100	67.610	50.558	18.153	1.0	1.63	PROT	H
HETATM	729	H5	ROC	C	100	67.474	49.108	16.154	1.0	1.66	PROT	H
HETATM	730	H6	ROC	C	100	67.547	48.819	13.715	1.0	1.66	PROT	H
HETATM	731	H7	ROC	C	100	67.861	50.808	12.248	1.0	1.66	PROT	H
HETATM	732	H8	ROC	C	100	67.940	53.079	13.260	1.0	1.75	PROT	H
HETATM	733	H	ROC	C	100	69.239	55.704	15.765	1.0	3.68	PROT	H
HETATM	734	HA	ROC	C	100	69.317	57.516	18.142	1.0	2.14	PROT	H
HETATM	735	HB1	ROC	C	100	70.401	58.080	15.302	1.0	1.76	PROT	H
HETATM	736	HB2	ROC	C	100	71.304	57.177	16.532	1.0	2.25	PROT	H
HETATM	737	HD22	ROC	C	100	71.956	59.892	15.289	1.0	3.15	PROT	H
HETATM	738	HD21	ROC	C	100	72.182	60.923	16.628	1.0	3.22	PROT	H
HETATM	739	H9	ROC	C	100	66.610	60.710	17.639	1.0	1.81	PROT	H
HETATM	740	H2	ROC	C	100	66.099	59.523	19.645	1.0	3.75	PROT	H
HETATM	741	HA1	ROC	C	100	65.366	58.858	16.189	1.0	1.60	PROT	H
HETATM	742	H12	ROC	C	100	66.957	57.480	18.260	1.0	3.76	PROT	H
HETATM	743	HB11	ROC	C	100	63.501	58.111	17.626	1.0	1.58	PROT	H
HETATM	744	HB12	ROC	C	100	64.592	57.821	19.003	1.0	1.70	PROT	H
HETATM	745	HD1	ROC	C	100	65.162	56.829	15.419	1.0	1.56	PROT	H
HETATM	746	HD2	ROC	C	100	64.409	55.494	19.431	1.0	1.59	PROT	H
HETATM	747	HE1	ROC	C	100	65.190	54.519	14.589	1.0	1.48	PROT	H
HETATM	748	HE2	ROC	C	100	64.515	53.138	18.643	1.0	1.56	PROT	H
HETATM	749	HZ	ROC	C	100	64.944	52.610	16.246	1.0	1.41	PROT	H
HETATM	750	H11	ROC	C	100	65.687	62.420	16.175	1.0	3.44	PROT	H
HETATM	751	HM1	ROC	C	100	63.641	61.145	18.002	1.0	1.90	PROT	H
HETATM	752	HM2	ROC	C	100	64.032	60.484	16.383	1.0	2.09	PROT	H
HETATM	753	H21	ROC	C	100	62.773	63.250	16.546	1.0	2.07	PROT	H
HETATM	754	H311	ROC	C	100	63.204	64.927	14.743	1.0	1.88	PROT	H

HETATM	755	H312	ROC	C	100	64.908	64.444	14.609	1.0	1.59	PROT	H
HETATM	756	H3A	ROC	C	100	63.407	65.509	17.081	1.0	1.74	PROT	H
HETATM	757	H411	ROC	C	100	64.476	67.509	16.858	1.0	1.41	PROT	H
HETATM	758	H412	ROC	C	100	64.217	67.068	15.161	1.0	1.52	PROT	H
HETATM	759	H511	ROC	C	100	66.486	68.066	15.665	1.0	1.25	PROT	H
HETATM	760	H512	ROC	C	100	66.543	66.491	14.836	1.0	1.33	PROT	H
HETATM	761	H611	ROC	C	100	67.296	67.099	17.764	1.0	1.37	PROT	H
HETATM	762	H612	ROC	C	100	68.333	66.418	16.490	1.0	1.39	PROT	H
HETATM	763	H711	ROC	C	100	67.447	64.684	18.164	1.0	1.44	PROT	H
HETATM	764	H712	ROC	C	100	67.157	64.319	16.462	1.0	1.49	PROT	H
HETATM	765	H7A	ROC	C	100	65.164	65.416	18.523	1.0	1.88	PROT	H
HETATM	766	H811	ROC	C	100	65.645	62.938	18.636	1.0	1.77	PROT	H
HETATM	767	H812	ROC	C	100	63.938	63.438	18.453	1.0	1.98	PROT	H
HETATM	768	H10	ROC	C	100	61.430	63.151	14.566	1.0	3.64	PROT	H
HETATM	769	H111	ROC	C	100	59.396	62.560	13.669	1.0	2.04	PROT	H
HETATM	770	H112	ROC	C	100	59.340	61.692	12.140	1.0	1.74	PROT	H
HETATM	771	H113	ROC	C	100	60.168	63.245	12.248	1.0	1.75	PROT	H
HETATM	772	H221	ROC	C	100	61.861	59.676	14.041	1.0	1.57	PROT	H
HETATM	773	H222	ROC	C	100	60.363	59.558	13.105	1.0	1.48	PROT	H
HETATM	774	H223	ROC	C	100	60.346	60.341	14.700	1.0	1.62	PROT	H
HETATM	775	H321	ROC	C	100	61.495	60.808	11.037	1.0	1.51	PROT	H
HETATM	776	H322	ROC	C	100	63.015	60.874	11.950	1.0	1.57	PROT	H
HETATM	777	H323	ROC	C	100	62.310	62.370	11.300	1.0	1.60	PROT	H
HETATM	778	OW	SOL	D6797		72.740	58.340	19.340	1.0	-7.58	PROT	O
HETATM	779	HW1	SOL	D6797		71.888	58.754	19.369	1.0	3.54	PROT	H
HETATM	780	HW2	SOL	D6797		73.319	58.841	19.923	1.0	3.71	PROT	H
HETATM	781	OW	SOL	D8066		65.170	67.280	12.640	1.0	-7.00	PROT	O
HETATM	782	HW1	SOL	D8066		65.377	66.684	11.915	1.0	3.55	PROT	H
HETATM	783	HW2	SOL	D8066		66.005	67.688	12.863	1.0	3.44	PROT	H
HETATM	784	OW	SOL	D9250		68.530	60.640	19.040	1.0	-7.45	PROT	O
HETATM	785	HW1	SOL	D9250		69.450	60.576	18.761	1.0	3.75	PROT	H
HETATM	786	HW2	SOL	D9250		68.305	59.766	19.379	1.0	3.59	PROT	H
HETATM	787	OW	SOL	D9550		67.540	54.780	21.170	1.0	-7.41	PROT	O
HETATM	788	HW1	SOL	D9550		67.691	55.109	20.289	1.0	3.59	PROT	H
HETATM	789	HW2	SOL	D9550		68.222	54.116	21.311	1.0	3.59	PROT	H
HETATM	790	OW	SOL	D	135	67.360	61.960	15.590	1.0	-7.57	PROT	O
HETATM	791	HW1	SOL	D	135	67.793	62.243	14.786	1.0	3.69	PROT	H
HETATM	792	HW2	SOL	D	135	67.713	61.081	15.799	1.0	3.90	PROT	H

```
HETATM  793  OW  SOL D 395      60.550  64.220  15.800  1.0  -8.33      PROT O
HETATM  794  HW1 SOL D 395      60.625  65.146  16.035  1.0   3.76      PROT H
HETATM  795  HW2 SOL D 395      59.589  64.063  15.599  1.0   4.20      PROT H
HETATM  796  OW  SOL D 403      65.560  59.050  13.830  1.0  -7.46      PROT O
HETATM  797  HW1 SOL D 403      64.949  59.769  13.987  1.0   3.83      PROT H
HETATM  798  HW2 SOL D 403      66.350  59.253  14.335  1.0   3.59      PROT H
END
```
